# Gene Expression Patterns Underlying the Reinstatement of Plasticity in the Adult Visual System

**DOI:** 10.1155/2013/605079

**Published:** 2013-06-26

**Authors:** Ettore Tiraboschi, Ramon Guirado, Dario Greco, Petri Auvinen, Jose Fernando Maya-Vetencourt, Lamberto Maffei, Eero Castrén

**Affiliations:** ^1^Neuroscience Centre, University of Helsinki, 00790 Helsinki, Finland; ^2^SARS Institute, University of Bergen, 5020 Bergen, Norway; ^3^Finnish Institute of Occupational Health, 00250 Helsinki, Finland; ^4^Institute of Biotechnology, University of Helsinki, 00790 Helsinki, Finland; ^5^Centre for Nanotechnology Innovation, Italian Institute of Technology, 56127 Pisa, Italy; ^6^Centre for Neuroscience and Cognitive Systems, Italian Institute of Technology, 38068 Rovereto, Italy; ^7^Neuroscience Institute, CNR, 56100 Pisa, Italy

## Abstract

The nervous system is highly sensitive to experience during early postnatal life, but this phase of heightened plasticity decreases with age. Recent studies have demonstrated that developmental-like plasticity can be reactivated in the visual cortex of adult animals through environmental or pharmacological manipulations. These findings provide a unique opportunity to study the cellular and molecular mechanisms of adult plasticity. Here we used the monocular deprivation paradigm to investigate large-scale gene expression patterns underlying the reinstatement of plasticity produced by fluoxetine in the adult rat visual cortex. We found changes, confirmed with RT-PCRs, in gene expression in different biological themes, such as chromatin structure remodelling, transcription factors, molecules involved in synaptic plasticity, extracellular matrix, and excitatory and inhibitory neurotransmission. Our findings reveal a key role for several molecules such as the metalloproteases Mmp2 and Mmp9 or the glycoprotein Reelin and open up new insights into the mechanisms underlying the reopening of the critical periods in the adult brain.

## 1. Introduction

 Use-dependent plasticity shapes neuronal networks within sensory systems during early life to optimally represent sensory stimuli [1]. Experience-dependent organization of eye-specific inputs is a major mechanism whereby refinement of synaptic connectivity is achieved in the developing visual system [2–4]. Monocular deprivation during development leads to a loss of cortical connectivity of the deprived eye resulting in a shift of the ocular dominance in the visual cortex, which will become permanent if the MD persists to adulthood [5, 6]. Although neuronal plasticity of the developing brain gradually decreases with age [7], recent findings suggest that it can be reactivated in the adult visual cortex [8] and other regions, such as the amygdala [9]. A variety of experimental manipulations, including enzymatic treatments [10, 11], environmental enrichment [12–15], food restriction [16], genetic manipulations [17, 18], and other manipulations, promote this kind of plasticity [19–21]. 

 Although the mechanisms behind the adult induced plasticity are still unclear, we are beginning to understand the key factors involved. For example, the developmental maturation of neuronal inhibition, mainly through the parvalbumin containing interneurons [22, 23], is known to be involved in both the opening and the closure of the critical period [20]. Several extracellular matrix components, such as PSA-NCAM or the perineuronal nets, have been shown to play a role in the maturation of the inhibitory circuitries and experimental manipulations removing these extracellular matrix components, can trigger an early closure [24] or a reopening of the critical period, respectively [11]. Similarly, a variety of other molecules, such as transcription factors [25] or proteins involved in chromatin structure remodeling [26], are also key factors in regulating the closure and reopening of the critical period.

The main pharmacological approaches to experimentally regulate critical period plasticity in the adulthood are those affecting the action of ascending projection systems, such as the serotoninergic or cholinergic systems [21, 27–29]. In this line, we have investigated the plastic effects of antidepressants, such as fluoxetine, that modulate serotoninergic transmission, and we have shown that these drugs, in a long-term treatment, are able to trigger critical period plasticity in the adult brain [8], through an early epigenetic modification that regulates gene expression [29]. 

 Here, we have used fluoxetine in combination with an experience-dependent paradigm of visual deprivation, to analyze the large-scale gene expression patterns, to understand the temporal-dependent changes that allow the reopening of the critical periods in the adult brain. 

## 2. Experimental Procedures

### 2.1. Animal Treatment, Fluoxetine Administration, and Surgical Procedures

**Figure 1:**
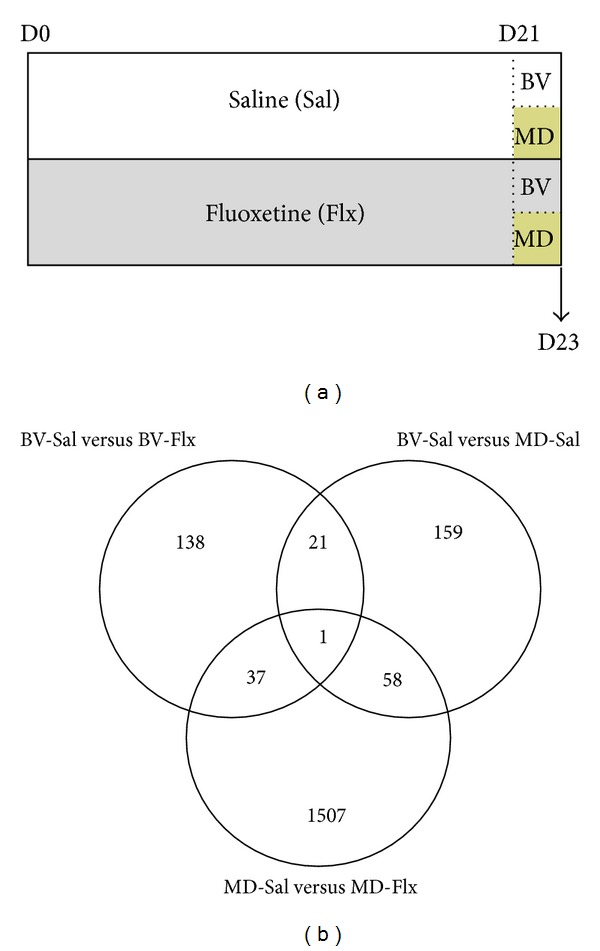
Experimental design and Venn diagram. (a) Schematic graph showing the different animal groups and experimental conditions conducted. (b) Venn diagram showing the number of genes differentially expressed when comparing the effects of monocular deprivation (BV-Sal versus MD-Sal), the effects of fluoxetine in animals with binocular vision (BV-Sal versus BV-Flx), and those of fluoxetine in animals with monocular deprivation (MD-Sal versus MD-Flx).

A total amount of 32 Long-Evans hooded rats were used in this study, equally distributed in 4 experimental groups (*n* = 8 animals per group), as explained later (Figure 1(a)). Animals were group-housed under standard conditions with food and water *ad libitum* in plexiglas cages (40 × 30 × 20 cm) and kept in a 12 : 12 light/dark cycle. Adult rats at the postnatal day 70 (P70) were systemically treated with fluoxetine (fluoxetine-hydrochloride, 0.2 mg/mL drinking water) for 23 days. Control animals were housed under the same standard conditions drinking tap water.

Three weeks after the beginning of the fluoxetine treatment, rats were anaesthetized with avertin (1 mL/100 g) and mounted on a stereotaxic apparatus to perform the eyelid suture for monocular deprivation (MD). Eyelids were inspected daily until complete cicatrisation; subjects with even minimal spontaneous reopening were excluded. Great care was taken during the first days after MD to prevent inflammation or infection of the deprived eye through topical application of antibiotic and cortisone. 

### 2.2. DNA Microarrays and Data Analysis

Two days after MD, the binocular region of the primary visual cortex was dissected. For all microarray experiments, total RNA was purified using RNA extraction kit (Macherey Nagel), and Amino Allyl cRNA labeling Kit (Ambion) was used to label cRNA according to manufacturer's standard protocols. Agilent Whole Rat Genome Microarray Kits (4 × 44 K) were hybridized following provided protocols.

Images from hybridized microarrays were segmented and the median intensity of each spot was estimated by the software GenePix v.5.0 (axon). Data was imported into the software (http://cran.r-project.org/) and preprocessed by the bioconductor package limma. The statistical analysis used was a linear model followed by *t*-test for finding the differentially expressed genes. In order to increase the reliability of the statistical analysis, we only considered significant those genes with a *P* value less than 0.01. In addition, we also increase the reliability of the analysis through validation of the results using multiple RT-PCRs. Lists of significant genes were screened by the DAVID 6.7 annotation tools in order to find overrepresented biological themes. Default DAVID parameters were used. 

### 2.3. Real-Time PCR

RNA purification was performed according to the standard Trizol procedure (Invitrogen). Purified RNA was treated with DNAse (Fermentas) and cDNA was synthesised from 1 *μ*g of RNA (Invitrogen). Real-time PCR was carried out to determine relative enrichment in the samples using the Sybr Green method according to the manufacturer instructions (SYBR Green I master, Light cycler 480, Roche Diagnostics). The comparative Ct method [30] was used to determine the normalized changes of the target gene relative to a calibrator reference; in particular, values were normalized to GAPDH levels. As calibrator reference we referred to Ct from water-treated animal samples. 

## 3. Results

### 3.1. DNA Microarrays

Previous studies have shown that 7 days period of monocular deprivation in fluoxetine-treated adult rats is sufficient to bring about a change in the ocular dominance. To reveal early transcriptional changes that precede and underlie the functional change, we analysed gene expression, using DNA microarrays, at two days after MD. Microarray analysis revealed only relatively few genes that were significantly regulated by either FLX (*n* = 197, see Supplementary Table S1 in the Supplementary Material available online at http://dx.doi.org/10.1155/2013/605079) or MD alone (*n* = 239, Table S2), treatments that themselves do not produce any changes in the ocular dominance plasticity. However, the combination of FLX and MD, the treatment that promotes changes in ocular dominance, altered the expression of a significantly larger number of genes (*n* = 1603, Table S3, Figure 1(b)). Notably 1237 out of 1603 (77%) of the genes in the group receiving both MD and FLX were downregulated, whereas in the groups receiving either MD or FLX, 111 out of 239 and 88 out of 197 genes were downregulated, respectively, comprising of roughly 50% of all the regulated genes. Hence, the combination of the treatments apparently has two major effects on gene expression; first, it increases the number of regulated genes when compared to the single treatments, and second, it has a striking effect on downregulating most of the genes, indicating that silencing of genes normally expressed during basal conditions is likely involved in the triggering of plasticity of the adult brain.

The representation of biological themes was screened using Fisher's Exact test on the lists of differentially expressed genes in each comparison. Chronic fluoxetine treatment induced a regulation of genes related to chromatin remodelling, nervous system development, and plasticity, as well as regulation of gene expression and transcription in the binocular visual cortex (Table S4). 

MD altered the expression of a significant number of genes related to cell differentiation, cell plasticity, and neurogenesis. Several genes of the ion homeostasis and regulation of transcription were also found overexpressed (Table S5). 

The combination of MD and fluoxetine treatment downregulated the majority of the differentially expressed genes, altering the expression of genes represented in a variety of functional processes, including genes related to neuronal development, plasticity, and apoptosis. In addition, genes involved in the synaptic transmission, ion and intracellular calcium homeostasis, and vesicular secretion were found differentially expressed. Blood circulation and lipid metabolism were among the most significantly overrepresented families (Table S6).

### 3.2. RT-PCR

To provide validation of the microarray data, we next examined single patterns of gene expression by means of real-time PCR, in the same experimental groups and using the same experimental paradigm (Figure 1(a)). In particular, we focused our attention on genes whose expression may alter molecular and cellular processes involved in the closure of the critical period for visual cortex plasticity, such as the balance of inhibitory and excitatory transmission [22, 31, 32], transcription factors regulating gene expression [25], extracellular matrix remodeling [11], myelination [33], and chromatin structure remodeling [26, 29], as well as genes involved in processes of synaptic plasticity, neuronal differentiation, and outgrowth (see Table 1).

#### 3.2.1. Inhibitory Neurotransmission

**Figure 2:**
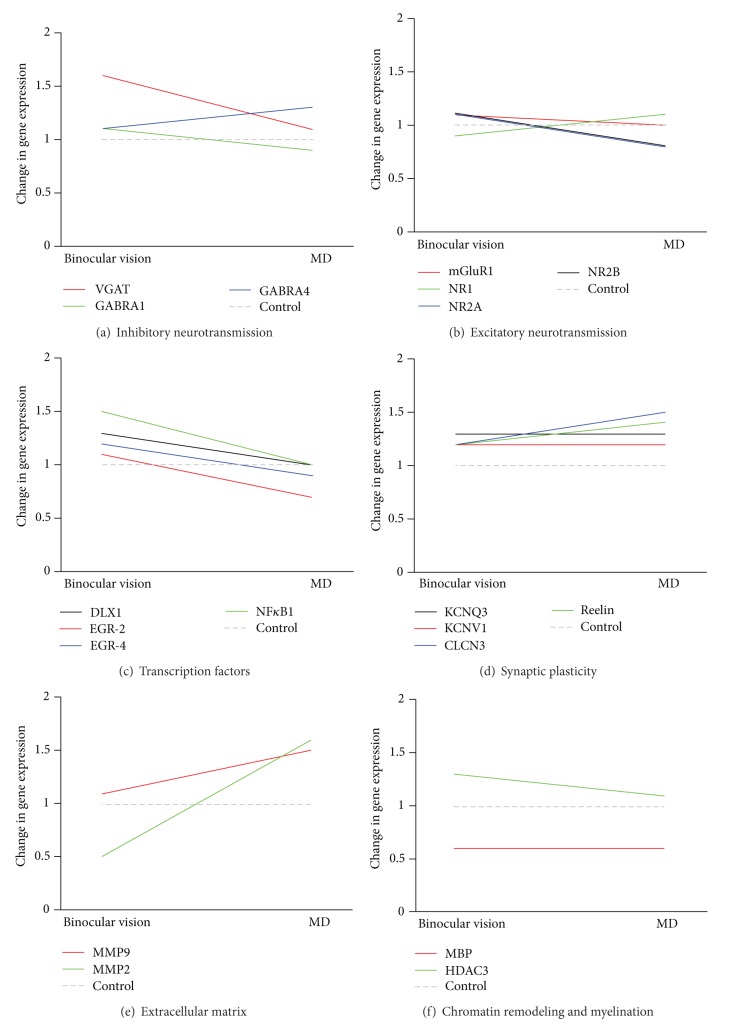
Effects of fluoxetine in the expression of genes involved in critical period plasticity. qRT-PCR mRNA fold change comparison between the effects of fluoxetine in rats with binocular vision and monocular deprivation. Statistical data is grouped by binocular vision or monocular deprivation, and in each part the position of the line represents the fold change of the fluoxetine treated group (coloured line) with respect to the saline treated group (grey dashed line) (BV-Sal versus BV-Flx and MD-Sal versus MD-Flx, resp.). All gene expression was normalized using GAPDH as a control gene.

We observed that fluoxetine produced a significant increase in the expression of genes involved in inhibitory neurotransmission when comparing both animals with binocular vision and animals with monocular deprivation with their respective controls (BV-Sal versus BV-Flx and MD-Sal versus MD-Flx; Figure 2(a)). Specifically, in animals with binocular vision, we found an increased expression of the vesicular GABA transporter (VGAT; 60% increased expression; *P* = 0.001), while in rats with monocular deprivation together with fluoxetine treatment, we observed an increase in the expression of GABRA4 (30% increased expression; *P* = 0.02).

#### 3.2.2. Excitatory Neurotransmission

We did not observe many changes in the composition of NMDA receptor subunits in either of the experimental groups (see Figure 2(b) and Table 1). The only significant change we found was a decrease in the expression of the NR2A subunit (NMDA-2A; 20% decreased expression; *P* = 0.04) in the animals with monocular deprivation treated with fluoxetine.

#### 3.2.3. Transcription Factors

We detected increases in the gene expression of transcription factors in the animals with binocular vision treated with fluoxetine (Figure 2(c)). In particular, NFKB1 and DLX1 increased their expression (50% and 30% increased expression, resp.; *P* = 0.04 and 0.03, resp.). However, in those animals with monocular deprivation, fluoxetine treatment produced a decrease in the expression of transcription factors, such as EGR-2 (*P* = 0.04).

#### 3.2.4. Synaptic Plasticity

 The expression of Reelin, transcript that encodes a glycoprotein that mediates synaptic plasticity at hippocampal level [34], was significantly increased in MD animals treated with fluoxetine (Figure 2(d); 40% increased expression; *P* = 0.001). The expression of additional transcripts that encode proteins involved in neuronal differentiation and outgrowth processes as well as synaptic plasticity increased in both groups. In animals with binocular vision, fluoxetine promoted an increase in the expression of CLCN3 (20% increased), KCNV1 (20% increased), and KCNQ3 (30% increased), which encode ion channels that mediate chloride and potassium conductance (*P* < 0.05), and in animals with monocular deprivation fluoxetine produced also an increase in the expression of CLCN3 (50% increased expression; *P* = 0.01).

#### 3.2.5. Extracellular Matrix

The expression of MMP2 and MMP9 was markedly changed between animals treated with fluoxetine and with binocular vision and those with monocular deprivation (Figure 2(e)). MMP2 and MMP9 encode for proteolytic enzymes that degrade extracellular matrix components [35–37] and play a key role in mediating synaptic plasticity at the level of the hippocampus [38, 39]. In particular, MMP2 gene expression was decreased in animals treated with fluoxetine alone (50% decrease; *P* = 0.02), while animals with combined monocular deprivation and chronic fluoxetine treatment had an increased expression of both MMP2 (60% increased; *P* = 0.01) and MMP9 (50% increased; *P* = 0.01). 

#### 3.2.6. Chromatin Remodeling and Myelination

 Changes in the expression of transcripts that encode an enzyme that regulate chromatin susceptibility to transcription were detected in animals with binocular vision after chronic fluoxetine treatment. In particular, we found that Hdac3 expression was enhanced (Figure 2(f); 30% increased *P* = 0.02). On the other hand, the expression of MBP, which encodes a basic protein of myelin, a repressive factor for visual cortex plasticity [33], was significantly reduced by fluoxetine treatment in animals with both binocular vision (40% decreased; *P* = 0.01) and monocular deprivation (40% decreased; *P* = 0.001).

## 4. Discussion

This study provides a large-scale analysis of changes in patterns of gene expression associated with the reopening of the critical period of plasticity in the adult visual system induced by the combination of fluoxetine treatment and monocular deprivation. Our findings suggest a scenario where an enhanced serotoninergic transmission induced by long-term fluoxetine treatment induces a shift of the inhibitory-excitatory balance [8, 29], which in turn promotes an alteration in the expression of genes involved in different biological themes that may underlie the functional modifications in the adult visual cortex related with the reopening of the critical period plasticity [40].

Our results reveal that the process of plasticity reactivation in adulthood involves both (i) a transient activation of neural mechanisms normally present during early stages of brain development and (ii) a removal of molecular factors that inhibit plasticity in adulthood [19]. Gene expression patterns involved in processes of synaptic plasticity, neuronal differentiation, and outgrowth were, indeed, differentially regulated by chronic fluoxetine treatment.

The increased expression of Reelin may represent an example of the first mechanism. Reelin is an extracellular glycoprotein involved in the migration and correct development of the cerebral cortex [41, 42]. Reelin is highly expressed by Cajal-Retzius neurons during development, but its expression is limited to a subpopulation of interneurons during the adulthood [43, 44]. Although the function of Reelin in adult neurons remains unclear, its overexpression has been shown to enhance plasticity and learning, affecting presynaptic transmission [34, 45]. Our results demonstrate an upregulation of Reelin after chronic fluoxetine treatment, suggesting that the overexpression of molecules involved in the juvenile plasticity plays an important role in the reopening of the critical periods during the adulthood.

 The proteolytic enzyme Mmp2, on the other hand, may drive mechanisms of synaptic plasticity by degrading extracellular matrix components that are inhibitory for plasticity, as observed in the adult hippocampus [39]. Increase of *Mmp2* expression, indeed, was paralleled by a decrease of *Mbp*: a basic component of myelin, which is a repressive factor for visual cortex plasticity [33]. Our analysis of gene expression points towards a downregulation of Mbp following long-term antidepressant treatment, supporting the hypothesis that the removal of factors that are inhibitory for plasticity may provide a permissive environment for structural and functional changes of neuronal circuitries in the adult nervous system [19]. 

Chronic fluoxetine administration has been shown to promote structural changes in both excitatory [46, 47] and inhibitory circuits [48–50]. Although there is evidence that long-term fluoxetine administration promotes a reduction of GABA-mediated inhibition in adult visual cortical circuitries [8, 29], a compensatory mechanism might explain the increase in the expression of VGAT or GABRA4 that we observe in our experiment. These results are also in agreement with previous studies, in which fluoxetine treatment in combination with monocular deprivation produces an increase in the elongation of the tips of interneuronal dendrites [50], supporting the idea that inhibitory neurotransmission plays a key role in the reopening of the critical periods [20, 22, 23]. Similarly, the change of NMDA receptor subunit composition, evidenced by the decrease in NMDA-2A gene expression following antidepressant treatment, is particularly interesting in this respect. The expression of the NR2A subunit has been correlated with a progressive decrease of NMDA receptor currents during development [51, 52]. This raises the possibility that a decrement of the NR2A/B ratio may increase NMDA receptors sensitivity thus causing the strengthening of synapses required for the potentiation of the nondeprived input [53].

 Another highly significant notion that emerges from our data is that the changes promoted by the combination of fluoxetine with monocular deprivation, regarding the expression of transcription factors and proteins of the extracellular matrix, are opposed to those promoted by fluoxetine alone. This indicates that these molecules might be underlying the structural plasticity changes driven by monocular deprivation to produce the shift in the ocular dominance and its consolidation in the visual system [54].

 Our findings support the hypothesis that the therapeutic effect of antidepressant drugs is dependent on changes in neuronal plasticity [55, 56]. Importantly, these results open up new insights into the understanding of the mechanisms underlying the reopening of the critical period in the adult brain, by providing the basis of gene expression patterns for a visual deprivation paradigm that demonstrates the ability of the nervous system to translate environmental stimuli into structural and functional changes of neural circuitries.

## Supplementary Material

Supplementary tables provide the bioinformatics analysis of the gene microarray data. S1 to S3 tables contain the fold change of expression of each particular gene when comparing different experimental groups, while supplementary tables S4 to S6 show the DAVID analysis by different biological themes in those same comparisons between the different groups.

## Figures and Tables

**Table 1:** RT-PCR analysis.

Gene symbol	Entrez gene ID	Treatment comparison	*P* value	Fold change
KCNQ3	29682	BV-Sal versus BV-Flx	0.01	1.3
KCNQ3	29682	MD-Sal versus MD-Flx	0.02	1.3

MMP9	81687	BV-Sal versus BV-Flx	0.13	1.1
MMP9	81687	MD-Sal versus MD-Flx	0.01	1.5

VGAT	83612	BV-Sal versus BV-Flx	0.001	1.6
VGAT	83612	MD-Sal versus MD-Flx	0.72	1.1

DLX1	296500	BV-Sal versus BV-Flx	0.03	1.3
DLX1	296500	MD-Sal versus MD-Flx	0.25	1.0

EGR2	114090	BV-Sal versus BV-Flx	0.86	1.1
EGR2	114090	MD-Sal versus MD-Flx	0.04	0.7

EGR4	25129	BV-Sal versus BV-Flx	0.29	1.2
EGR4	25129	MD-Sal versus MD-Flx	0.38	0.9

mGluR1	24414	BV-Sal versus BV-Flx	0.47	1.0
mGluR1	24414	MD-Sal versus MD-Flx	0.98	1.1

HDAC3	15183	BV-Sal versus BV-Flx	0.02	1.3
HDAC3	15183	MD-Sal versus MD-Flx	0.92	1.1

KCNV1	60326	BV-Sal versus BV-Flx	0.02	1.2
KCNV1	60326	MD-Sal versus MD-Flx	0.10	1.2

NFKB1	81736	BV-Sal versus BV-Flx	0.04	1.5
NFKB1	81736	MD-Sal versus MD-Flx	0.52	1.0

CLCN3	84360	BV-Sal versus BV-Flx	0.04	1.2
CLCN3	84360	MD-Sal versus MD-Flx	0.01	1.5

NR1	24408	BV-Sal versus BV-Flx	0.45	0.9
NR1	24408	MD-Sal versus MD-Flx	0.43	1.1

NR2A	14811	BV-Sal versus BV-Flx	0.63	1.1
NR2A	14811	MD-Sal versus MD-Flx	0.04	0.8

NR2B	24410	BV-Sal versus BV-Flx	0.51	1.1
NR2B	24410	MD-Sal versus MD-Flx	0.07	0.8

GABRA1	29705	BV-Sal versus BV-Flx	0.34	1.1
GABRA1	29705	MD-Sal versus MD-Flx	0.27	0.9

GABRA4	140675	BV-Sal versus BV-Flx	0.29	1.1
GABRA4	140675	MD-Sal versus MD-Flx	0.02	1.3

Reelin	24718	BV-Sal versus BV-Flx	0.15	1.2
Reelin	24718	MD-Sal versus MD-Flx	0.001	1.4

MMP2	17390	BV-Sal versus BV-Flx	0.02	0.5
MMP2	17390	MD-Sal versus MD-Flx	0.01	1.6

MBP	24547	BV-Sal versus BV-Flx	0.01	0.6
MBP	24547	MD-Sal versus MD-Flx	0.01	0.6
