# Supplementary material for: Gene Expression Patterns Underlying the Reinstatement of Plasticity in the Adult Visual System
Source: Neural Plast. 2013 Jun 26;2013:605079. doi: 10.1155/2013/605079 (PMC3710606; doi:10.1155/2013/605079)
Supplement: Supplementary file 1 — Supplementary tables provide the bioinformatics analysis of the gene microarray data. S1 to S3 tables contain the fold change of expression of each particular gene when comparing different experimental groups, while supplementary tables S4 to S6 show the DAVID analysis by different biological themes in those same comparisons between the different groups. [file 605079.f1.pdf]

Table S1

ID	logFC	AveExpr	P.Value	GenbankAccession	GeneSymbol
A_44_P505500	1.607556	11.90701	0.00122	CK358354	
A_43_P12141	1.561647	11.19161	0.001065	NM_021869	Stx7
A_44_P992854	1.324382	11.73082	0.002197	NM_057132	Rhoa
A_44_P196717	1.318426	10.03627	0.004003	XM_344970	Stk32c_predicted
A_44_P113842	1.173214	8.328451	0.000128	NM_022210	Max
A_44_P403482	1.15254	8.621625	0.000569	XM_213493	RGD1311493_predicted
A_44_P248111	1.143281	9.320377	1.87E-05	NM_001047107	Igsf4c_predicted
A_43_P16511	1.072354	9.35087	0.009672	NM_001044300	MGC112715
A_42_P522171	1.030953	9.111699	0.001285	XM_001081512	RGD1307966_predicted
A_44_P223252	1.015676	13.88146	0.009124	NM_031595	Psmc3
A_44_P239105	1.014573	8.624869	0.003369	XM_233341	Zfyve9_predicted
A_44_P563128	1.013565	7.972642	0.00074	XR_007778	
A_44_P864185	0.99365	9.886928	0.004432	AW142955	
A_44_P255954	0.985852	11.34905	0.008416	XM_573223	RGD1566063_predicted
A_44_P548507	0.976934	14.00466	0.003327	NM_032083	Chn1
A_42_P713975	0.96633	14.00655	0.002899	BC082799	LOC499779
A_43_P13247	0.945033	9.21568	0.001365	XM_001070141	Psd
A_44_P187789	0.919478	8.124942	0.001183	XM_001066818	
A_43_P16512	0.917668	12.84756	0.007548	XM_218824	RGD1311021_predicted
A_44_P769703	0.915946	8.731323	0.009651	NM_178096	Nrep
A_44_P210461	0.909428	13.77556	0.005967	NM_017025	Ldha
A_44_P176606	0.891879	7.720083	0.005843	NM_001007235	Itpr1
A_44_P480605	0.877686	13.69188	0.008763	XM_001066230	LOC291543
A_44_P1029397	0.874681	9.78541	0.006524	NM_198134	Bst2
A_44_P463858	0.858747	7.846463	0.000999	NM_133303	Bhlhb3
A_44_P189326	0.845271	12.61675	0.003442	NM_017319	Pdia3
A_43_P10162	0.837812	9.295357	0.0075	XM_224841	Odz3_predicted
A_43_P11622	0.797153	14.22093	0.002926	NM_012925	Cd59
A_44_P552865	0.794559	12.17696	0.008261	XM_221896	LOC304280
A_44_P508264	0.752347	7.640307	0.002071	NM_031762	Cdkn1b
A_44_P424570	0.739812	7.775522	0.002451	XM_235521	Poldip3_predicted
A_43_P12779	0.734704	13.88252	0.006576	NM_053346	Nrn1
A_44_P379976	0.719224	8.728674	0.009711	NM_001024782	Lrrc8
A_44_P797926	0.71717	11.582	0.009305	BG673275	
A_44_P506374	0.709429	8.661933	0.004513	XM_001073596	Nyw1
A_44_P356658	0.709393	8.23732	0.009249	XM_219716	Pdzd4_predicted
A_44_P108421	0.689004	9.564137	0.00606	XM_224350	Fndc3a_predicted
A_43_P16811	0.685518	9.346495	0.008035	NM_001014073	RGD1305486
A_44_P473322	0.680296	7.868224	0.005619	XM_224778	
A_44_P417077	0.679432	8.061647	0.001949	XM_573664	RGD1566234_predicted
A_44_P403410	0.65013	11.05502	0.004235	NM_001079895	Rbm9_predicted
A_44_P714315	0.649504	12.25655	0.006677	CO404950	
A_44_P501483	0.646159	14.38167	0.006042	XR_006667	LOC310385
A_44_P156966	0.642122	9.596286	0.007913	XM_228708	RGD1563226_predicted

A_44_P350794	0.5956	8.367077	0.009356	AA894317	
A_43_P10735	0.590686	11.8257	0.009669	BF555100	RGD1566317_predicted
A_43_P17622	0.586863	9.765676	0.005446	XM_345870	Lass5_predicted
A_42_P704370	0.57628	13.13036	0.008636	NM_023972	Arl6ip5
A_44_P448307	0.576147	14.16514	0.004325	XM_217432	Arpc2_predicted
A_44_P473472	0.569886	10.61491	0.008531	BC091233	RGD1307434_predicted
A_43_P21003	0.560543	10.93669	0.006849	NM_001033892	RGD1306841
A_44_P738831	0.55264	8.290444	0.004326	BE105152	
A_44_P189813	0.551359	9.934035	0.00539	XM_214295	Glt25d1_predicted
A_44_P161220	0.542591	14.16572	0.008859	NM_080482	Dbccr1
A_43_P19892	0.536862	10.33035	0.006277	NM_001014255	Aph1a
A_44_P108680	0.53537	8.102587	0.006747	NM_057116	Ppp2r2c
A_44_P229618	0.527464	8.367113	0.008818	XM_228065	Tmem1_predicted
A_44_P255236	0.525992	9.239359	0.009952	NM_019212	Acta1
A_44_P455065	0.518588	14.18702	0.006125	XM_001077680	RGD1564600_predicted
A_44_P208275	0.511361	8.057972	0.001406	NM_031795	Ugcg
A_43_P15848	0.510606	9.342219	0.007543	M81783	Kcnf1
A_44_P180294	0.507879	8.206421	0.006738	XM_344235	
A_44_P180676	0.507554	7.614493	0.004982	XM_341141	
A_44_P274573	0.507395	9.295892	0.007497	XM_232283	Plxnd1_predicted
A_44_P222865	0.490304	14.40565	0.009316	XR_006686	
A_44_P101023	0.487674	10.99103	0.008547	XM_239335	RGD1564778_predicted
A_44_P194863	0.470415	11.45454	0.005744	XM_220574	Spag7_predicted
A_42_P573610	0.468416	7.658664	0.003621	XM_233544	Eif2c1_predicted
A_44_P142843	0.46753	8.161509	0.009195	XM_001065442	LOC685834
A_42_P707937	0.463584	13.34077	0.006472	NM_080583	Ap2b1
A_44_P549028	0.462526	9.336237	0.005687	XM_001076082	
A_43_P17264	0.458705	8.929598	0.00814	XM_240329	RGD1565549_predicted
A_44_P720473	0.453531	8.555751	0.00795	CA512655	
A_44_P267155	0.448921	7.431128	0.008325	XM_221887	
A_44_P827752	0.443672	7.507669	0.008733		
A_43_P18570	0.433168	7.264582	0.003564	XM_236194	Mll
A_43_P21027	0.433031	8.243299	0.007216	XM_235023	RGD1560606_predicted
A_44_P195291	0.431798	12.51609	0.002861	XM_233382	RGD1560252_predicted
A_43_P23298	0.431665	8.463371	0.003823	XM_001079737	RGD1311730_predicted
A_44_P1005858	0.428954	8.971588	0.009179	CO404131	
A_44_P147980	0.42782	8.163945	0.003669	XM_576503	
A_44_P546901	0.41725	7.567971	0.007509	NM_012502	Ar
A_44_P304493	0.417047	8.166562	0.005191	NM_001024970	RGD1305572
A_43_P21762	0.387674	7.279607	0.007224	NM_001013187	Slc25a30
A_44_P333550	0.370196	7.827262	0.009141	NM_001013986	LOC305076
A_44_P119442	0.36235	11.97699	0.00757	XM_216492	Creb3
A_44_P277126	0.362131	7.823398	0.007942	XM_221369	
A_44_P133226	0.361641	7.62301	0.007063	XM_224852	Stox2
A_42_P601971	0.351093	7.762251	0.004338	XM_220328	Sox30_predicted
A_42_P504240	-0.304158	14.22476	0.007788	XM_237998	

A_44_P637629	-0.337548	12.37837	0.008706	DV727233	
A_42_P771373	-0.352053	15.04484	0.008398	NM_012614	Npy
A_44_P561760	-0.371864	10.60745	0.009932		
A_44_P479766	-0.372905	7.061162	0.008134	BM986564	
A_44_P535706	-0.375911	12.21228	0.007752	XM_216485	
A_42_P626023	-0.381003	10.46022	0.006668	XM_224232	Cenpj_predicted
A_44_P255384	-0.383536	15.24025	0.009335	XM_344225	RGD1562394_predicted
A_42_P570848	-0.388127	9.382288	0.007135	NM_017091	Pcsk1
A_42_P791872	-0.395678	15.20882	0.009108	XM_001061587	LOC682454
A_44_P212695	-0.39929	15.55995	0.00973	XM_212890	
A_42_P549321	-0.402635	15.09135	0.003742		
A_44_P473731	-0.40425	15.44158	0.00932	XM_220717	Abr_predicted
A_44_P762268	-0.405311	13.79055	0.006136		
A_44_P792063	-0.406364	12.72072	0.003616		
A_44_P593331	-0.407158	11.10025	0.00566		
A_44_P501390	-0.410068	12.88181	0.009047	XM_001055728	RGD1561042_predicted
A_44_P884739	-0.411871	14.79085	0.004969	XM_219339	Sez6l2_predicted
A_44_P1043979	-0.416159	15.46051	0.000961	NM_001030052	Ndufs5b
A_44_P161709	-0.418994	15.66453	0.005418	XM_345662	
A_42_P634674	-0.420247	15.27808	0.008207	NM_145783	Cox5a
A_43_P11268	-0.420794	12.19783	0.007261	NM_031762	Cdkn1b
A_44_P525486	-0.440114	10.56008	0.009935	XM_343841	Plxnb3_predicted
A_44_P592613	-0.444442	11.81328	0.008322	XM_576601	RGD1560766_predicted
A_42_P703403	-0.448031	15.45875	0.00121	NM_031974	Clta
A_44_P866539	-0.448651	15.46571	0.003064	AW913986	
A_44_P554867	-0.449101	13.80371	0.006241	AW917688	
A_44_P792470	-0.456522	10.01488	0.00406	DV722661	
A_43_P10505	-0.459756	15.07648	0.009444	XM_226549	Jph3_predicted
A_44_P995316	-0.460859	12.4553	0.007066	XM_343081	Sgpp1
A_44_P1055366	-0.461078	15.4244	0.005917	AW919175	
A_44_P914323	-0.461087	14.00556	0.009917	DV718684	
A_43_P16707	-0.462778	8.462194	0.007004	NM_001004202	Ccl6
A_44_P730454	-0.463051	11.17696	0.009253	XM_341399	Mapk8
A_42_P623151	-0.46981	15.46891	0.002427	NM_031026	Dncli2
A_44_P638345	-0.46998	11.36836	0.009927	NM_001034130	Wasl
A_44_P152635	-0.471578	7.525866	0.00501	NM_021857	Htr1f
A_44_P822928	-0.471616	12.19977	0.000854		
A_44_P156922	-0.473462	15.34944	0.008611	NM_001008312	Serinc3
A_44_P745579	-0.47912	11.5586	0.004111		
A_44_P1031514	-0.480469	12.12573	0.005639	NM_001014054	RGD1307778
A_44_P361202	-0.481728	13.36097	0.003147	CB569688	
A_42_P619248	-0.48445	15.32374	0.005621	XM_001053199	RGD1560511_predicted
A_44_P392886	-0.484852	15.8303	0.002919	CB567353	ND2
A_42_P614711	-0.494634	10.99742	0.003489	BF548552	
A_42_P475623	-0.495946	11.46603	0.007179	NM_053394	Klf5
A_43_P12692	-0.497221	15.00459	0.008597	NM_031814	Git1

A_44_P727480	-0.499614	12.44601	0.005736	AW142602	
A_44_P976007	-0.501242	10.5042	0.007044	DV719080	
A_44_P357478	-0.503165	7.182014	0.007098	CK481254	
A_44_P775118	-0.503898	10.08036	0.002595	NM_001014205	Tmem123
A_44_P372261	-0.505527	7.827664	0.00773	NM_053372	Slpi
A_44_P965429	-0.506297	11.15919	0.009965		
A_44_P393551	-0.507718	15.04609	0.006586	NM_012567	Gja1
A_44_P700333	-0.507758	11.50827	0.001397	CX570601	
A_43_P15460	-0.50937	11.70692	0.008183	NM_053879	Cntn4
A_44_P299247	-0.515458	14.98393	0.00915	NM_012778	Aqp1
A_44_P311334	-0.516913	8.09389	0.007592	L27060	Pde4d
A_44_P996952	-0.517441	9.262775	0.007491	NM_001039454	RGD1561028
A_44_P373681	-0.517613	11.90056	0.008317	BF563424	
A_42_P544321	-0.521129	10.1151	0.007505	AI136185	
A_44_P791696	-0.521548	11.62472	0.003333	DV725758	
A_44_P493603	-0.525791	11.8103	0.007827	DY472377	
A_44_P270700	-0.537381	15.29789	0.002379	BG663460	
A_42_P521814	-0.537861	11.63224	0.009573		
A_44_P640301	-0.54291	13.82233	0.0009	XM_001065707	RGD1566031_predicted
A_42_P525317	-0.54563	11.28367	0.006987	NM_053851	Cacnb2
A_44_P884444	-0.546077	10.18235	0.001586	XM_343782	RGD1563952_predicted
A_44_P650550	-0.558033	11.4954	0.001961	BF289404	
A_43_P11492	-0.559424	7.373786	0.002587	NM_012629	Prl
A_42_P839655	-0.561899	14.45612	0.003112	BF555716	
A_44_P443657	-0.564112	12.11021	0.008022	BI281988	
A_44_P655666	-0.576235	10.32947	0.003753		
A_42_P503281	-0.577323	11.59358	0.002812	NM_053603	Clic5
A_43_P12283	-0.579016	12.80167	0.004402	NM_022668	Mog
A_44_P884766	-0.5965	15.49362	0.000367		
A_44_P702115	-0.601856	10.62476	0.003276		
A_44_P283909	-0.609549	9.066482	0.008587	XM_216079	
A_44_P175677	-0.618299	7.45903	0.001548	XM_001062365	LOC685114
A_44_P386633	-0.630651	13.84789	0.00755	NM_145785	Hdgfrp3
A_44_P956310	-0.631047	7.415374	0.005342	AI171999	
A_42_P621736	-0.6312	15.79036	0.005605	NM_001007604	Rplp1
A_44_P469517	-0.631758	9.924125	0.005796	BF551572	
A_44_P605123	-0.644716	10.78691	0.005895	DV716579	
A_44_P770724	-0.651454	10.89887	0.002755	XM_225941	Dmxl1_predicted
A_44_P1017996	-0.663612	11.77483	0.00737	XM_341470	LOC361188
A_44_P350074	-0.666196	14.75075	0.004962	XM_001075009	LOC686892
A_44_P914438	-0.668649	10.16228	0.009157	NM_080782	Cdkn1a
A_44_P593071	-0.674446	7.774681	0.003372		
A_42_P575140	-0.683968	11.45806	0.009127	AW916021	
A_44_P652899	-0.703191	15.67359	0.004507	NM_178105	Gpm6a
A_44_P793157	-0.711691	9.441904	0.005513		
A_44_P416127	-0.713822	14.78112	0.002027	BG665128	

A_44_P123857	-0.729971	7.392116	0.00623	XM_227855	
A_44_P576019	-0.731695	9.762224	0.009106		
A_44_P556677	-0.742666	12.03146	0.004327	AW525326	
A_44_P531685	-0.774814	15.7317	0.002598	NM_212511	Rpl35
A_42_P484738	-0.777717	10.55331	0.009229	NM_022266	Ctgf
A_44_P976667	-0.778059	11.33574	0.002631		
A_44_P575265	-0.78268	7.162824	0.005089	XM_001059898	
A_44_P341298	-0.801823	10.7338	0.001651	BF396386	
A_44_P684912	-0.868223	11.62549	0.004168		
A_44_P344475	-0.871819	11.22742	0.007204	NM_053522	Rhoq
A_44_P608966	-0.898967	11.02197	0.002516		
A_42_P783113	-0.940675	15.01051	0.008372	XM_215044	
A_44_P728524	-0.948968	8.649392	0.004864	XM_218037	Zfp54_predicted
A_44_P353618	-1.598265	10.41172	0.006037	NM_053587	S100a9
A_44_P1055780	-1.799489	9.625261	0.00237	NM_053822	S100a8

Table S2

ID	logFC	AveExpr	P.Value	GenbankAccession	GeneSymbol
A_44_P255954	1.092079	11.34905	0.004114	XM_573223	RGD1566063_predicted
A_43_P16512	0.958391	12.84756	0.005599	XM_218824	RGD1311021_predicted
A_44_P199211	0.888268	13.1782	0.007	NM_001077641	Plcb1
A_44_P519174	0.865479	10.15332	0.001505	NM_001015027	Crebl2
A_44_P797926	0.827698	11.582	0.003393	BG673275	
A_44_P638014	0.786066	9.362983	0.001642	XM_001067431	Irf6_predicted
A_44_P253196	0.72852	10.6956	0.000407	XM_228114	Arid5b_predicted
A_44_P817120	0.705276	10.23002	0.000413	BF563826	
A_44_P764308	0.70371	12.43124	0.00571		
A_44_P515211	0.702895	9.195753	0.000603	NM_145765	Tnfsf15
A_44_P405203	0.692478	10.26765	0.001054	XM_218664	Hebp2_predicted
A_44_P314492	0.692285	10.13554	0.004549	XM_221672	Tiam1
A_44_P108421	0.689739	9.564137	0.006015	XM_224350	Fndc3a_predicted
A_44_P274573	0.681768	9.295892	0.000701	XM_232283	Plxnd1_predicted
A_44_P525431	0.678326	10.94687	0.001897		
A_43_P11622	0.670731	14.22093	0.009935	NM_012925	Cd59
A_44_P1050433	0.669816	10.15768	0.008041	XM_001078552	LOC691501
A_43_P19994	0.651395	9.365389	0.004094	XM_233485	Rlf_predicted
A_44_P473472	0.649492	10.61491	0.003372	BC091233	RGD1307434_predicted
A_44_P168514	0.64246	9.344473	0.001894	XM_226964	Zfhx4_predicted
A_44_P377645	0.63785	8.537705	0.000763	XM_342774	
A_44_P300612	0.625149	10.59645	0.008106	NM_022850	Dpp6
A_43_P17175	0.624011	8.564087	0.001089	NM_001031642	Serpinb1a
A_43_P17180	0.623162	11.83235	0.002449	NM_001007696	mrpl9
A_44_P323430	0.620476	9.88312	0.008344	NM_138532	Nme7
A_44_P422271	0.600331	10.07162	0.00451	XM_217019	Cerk_predicted
A_44_P525645	0.592939	8.785316	0.008531	XM_220442	
A_44_P175495	0.59217	7.297441	0.003832	NM_182952	Cxcl11
A_44_P163242	0.587393	8.543951	0.00271	NM_138541	Tacstd1
A_44_P102291	0.58478	9.037239	0.001128	XM_341619	Adamts19_predicted
A_43_P10020	0.579306	12.71223	0.009383	NM_012771	Lyz
A_44_P522492	0.577851	8.866765	0.002137	NM_133414	Clcc1
A_44_P348973	0.574847	8.505955	0.006247	NM_001004277	Lypla3
A_44_P160846	0.574796	8.886562	0.000923	NM_182673	Rnasel
A_44_P1027904	0.574744	12.94124	0.003016	XM_213794	Pop5_predicted
A_44_P269425	0.57387	10.3993	0.005824	NM_133415	Efcbp2
A_43_P17880	0.572611	8.703786	0.003833	NM_182953	Nek6
A_44_P440633	0.569351	10.4556	0.0078	NM_031639	Dlgh3
A_44_P255236	0.561685	9.239359	0.006445	NM_019212	Acta1
A_44_P469113	0.561279	9.121905	0.004531	XM_220779	Slfn2_predicted
A_42_P744464	0.559015	8.758601	0.006845	XM_340740	RGD1565449_predicted
A_44_P247219	0.558874	9.764186	0.001307	NM_053699	Cited4
A_42_P453976	0.554018	9.751298	0.000729	XM_219476	Ifitm6_predicted
A_44_P191022	0.553331	11.25682	0.006487	NM_053674	Phyh
A_44_P492629	0.552641	11.97851	0.003115	DV727624	LOC497975
A_43_P10824	0.546754	11.88143	0.00131	NM_001015010	Cib2
A_43_P18186	0.539919	10.43356	0.008235	XM_232226	Pdzrn3_predicted

A_44_P408520	0.539577	10.59062	0.006601	NM_031569	Oprl1
A_44_P189813	0.539428	9.934035	0.006284	XM_214295	Glt25d1_predicted
A_44_P150471	0.535326	12.78451	0.00827	NM_031696	Gpr88
A_43_P12802	0.5329	12.46216	0.00132	NM_053418	Ufd1l
A_44_P445369	0.532457	8.410379	0.004245	XM_001080476	LOC360824
A_44_P114184	0.525917	10.4511	0.003452	NM_022543	Ssg1
A_44_P134634	0.519467	9.390154	0.008789	CB576476	RGD1562272_predicted
A_44_P309523	0.516512	10.28453	0.007552	XM_001076269	LOC501230
A_44_P273449	0.514881	8.7635	0.008173	XM_214953	Perp_predicted
A_44_P823303	0.512983	7.675391	0.004082		
A_44_P729266	0.503142	7.240329	0.001277	XR_005718	LOC300506
A_43_P18294	0.500507	7.598985	0.003877	BC099835	
A_44_P314353	0.499854	9.286162	0.008369	NM_001037765	RGD1306649
A_44_P288241	0.496253	8.737087	0.006619	XM_341029	RGD1307396_predicted
A_44_P167408	0.492468	9.154808	0.007935	AW144014	
A_44_P229051	0.492248	7.852318	0.00518	NM_012748	Epim
A_44_P668388	0.490809	12.07127	0.004134	BC098022	RGD1561494_predicted
A_44_P408108	0.475979	9.272041	0.008532	XM_236649	Nbeal2_predicted
A_44_P189953	0.471873	9.605232	0.008348	NM_031769	Pdxk
A_44_P549028	0.471493	9.336237	0.004959	XM_001076082	
A_42_P839541	0.466727	8.176077	0.008615	NM_001007671	Cyb5d2
A_44_P252756	0.466033	9.749369	0.008365	XM_221874	
A_44_P655158	0.462016	10.96723	0.003263	CO567276	
A_44_P426624	0.46039	9.187297	0.002826	XM_344380	
A_43_P23215	0.456773	10.24316	0.001849	NM_001025737	Smpdl3b
A_44_P299123	0.452931	7.997765	0.006893	XM_215847	Nphp1_predicted
A_44_P456747	0.438792	8.694571	0.005099	AB012231	Nfib
A_44_P237220	0.438141	9.322389	0.003965	XM_214720	
A_44_P148280	0.437942	12.38004	0.007917	NM_053670	Crcp
A_43_P10534	0.434336	11.85153	0.00493	NM_001034108	Mar.02
A_44_P332817	0.430724	12.22793	0.008347	NM_053527	Cdc5l
A_43_P20794	0.418891	8.354658	0.007973	BC091326	Osbpl5
A_44_P483177	0.416901	11.34909	0.006728	NM_001009344	Tbrg1
A_43_P19369	0.414985	8.634819	0.008199	XM_219805	Prkg1_predicted
A_44_P545649	0.410999	7.755706	0.001615	NM_001014093	Parp16
A_44_P111825	0.410741	8.817684	0.006841	NM_001014144	RGD1305679
A_44_P1051765	0.405347	11.08866	0.005446	XM_342984	Thap3_predicted
A_44_P109834	0.401496	7.550092	0.006214	XM_341783	Rdh13_predicted
A_44_P809514	0.398808	7.858837	0.00334		
A_44_P1038400	0.394146	12.34258	0.005052	DV727624	LOC497975
A_43_P10315	0.393843	8.182339	0.009911	XM_341626	Gnal
A_44_P289173	0.390852	9.276699	0.007259	BC097985	Grtp1
A_44_P187300	0.390448	8.451293	0.008284	XM_344202	
A_44_P228903	0.389952	7.646097	0.001145	NM_078621	Ccbp2
A_44_P532500	0.387401	9.399204	0.008349	XM_217354	
A_43_P12267	0.380794	7.482954	0.00323	NM_022623	Fzd4
A_44_P424069	0.380306	7.490027	0.006055	XM_218463	Zfp84_predicted
A_44_P370082	0.379629	12.25524	0.005576	XM_340856	Timm22
A_43_P15129	0.377676	7.558528	0.009304	BF558849	

A_44_P349817	0.373434	7.119817	0.006636	XM_237215	LOC301455
A_44_P1010416	0.365499	8.425505	0.003415	NM_001002853	P2ry13
A_44_P447927	0.362439	7.278709	0.004074		
A_44_P356482	0.359146	7.30241	0.008106		
A_44_P119442	0.353803	11.97699	0.00886	XM_216492	Creb3
A_44_P409539	0.350217	7.44798	0.004442	NM_175869	Plod2
A_44_P407800	0.350096	7.710196	0.001764	XM_225607	
A_43_P17282	0.349379	7.370769	0.00758	XM_219879	Cnnm1_predicted
A_42_P809101	0.345238	10.43971	0.005251	XM_342873	Tctex1d1_predicted
A_44_P468816	0.340427	7.136901	0.008588	XM_001057103	
A_42_P601971	0.333952	7.762251	0.006207	XM_220328	Sox30_predicted
A_44_P398433	0.328459	7.746764	0.00909	XM_224923	Tnks_predicted
A_44_P156846	0.326852	7.465644	0.009923	XM_231139	Lamc3_predicted
A_44_P370774	0.321406	7.045656	0.007011	XM_235183	Irak3_predicted
A_44_P260384	0.313235	7.263999	0.00804	XM_240915	RGD1307915_predicted
A_44_P388709	0.279068	7.125181	0.007876	NM_145767	Prrxl1
A_44_P591036	-0.312213	7.029821	0.008327	XM_001069503	LOC685839
A_43_P11492	-0.341474	7.590819	0.006493	NM_012629	Prl
A_44_P229557	-0.344053	10.42845	0.008754	XM_230513	RGD1561554_predicted
A_44_P518050	-0.364194	11.07642	0.004205	XM_223566	Sf3a1_predicted
A_44_P184865	-0.377312	14.20673	0.007668	NM_031579	Ptp4a1
A_44_P1002407	-0.383112	12.21029	0.009369	AI137378	
A_44_P334736	-0.388138	8.79879	0.002976	NM_012548	Edn1
A_44_P839802	-0.389477	7.467368	0.008537		
A_44_P307646	-0.395441	7.041288	0.007608	BI289723	
A_44_P420046	-0.409126	8.398432	0.009059	NM_021669	Ghrl
A_42_P736812	-0.412467	13.07906	0.004888	NM_134449	Prkcdbp
A_44_P777113	-0.4134	11.74969	0.008401	XM_001068152	LOC684233
A_42_P840867	-0.416389	11.51094	0.005105	XR_008037	RGD1561455_predicted
A_44_P597013	-0.422945	9.936548	0.006715		
A_44_P288443	-0.423627	10.61669	0.00444	XM_344542	Thsd1_predicted
A_43_P13326	-0.425342	9.86345	0.003719	NM_138894	Grasp
A_44_P110334	-0.427016	7.55124	0.004078	XM_236387	RGD1560796_predicted
A_43_P23304	-0.429392	11.02713	0.004137	NM_199397	Panx1
A_44_P428338	-0.437758	7.45371	0.000983	AA817984	
A_44_P1050510	-0.440611	8.275122	0.007818	XM_001075804	LOC686809
A_44_P127962	-0.442185	9.386424	0.006015	NM_181771	Gpr135
A_44_P379645	-0.450221	7.565198	0.004867	AF030090	
A_44_P578593	-0.4523	9.756066	0.004554		
A_43_P18201	-0.452482	11.10478	0.007172	XM_230639	Zfp339_predicted
A_44_P283372	-0.452635	15.04796	0.00478	BC062235	Hnrpa1
A_44_P960490	-0.453901	13.07605	0.005162	XM_573497	
A_44_P171321	-0.460493	12.38677	0.003954	NM_001009671	RGD1359509
A_42_P737455	-0.463557	12.01383	0.00428	AI071866	
A_44_P182739	-0.466768	8.675444	0.009307	XM_217651	Pdzk8_predicted
A_44_P853624	-0.468433	10.94544	0.006035	XM_237855	RGD1308321_predicted
A_44_P650550	-0.475469	11.4954	0.00659	BF289404	
A_44_P745585	-0.483808	11.0584	0.00908	NM_001024309	LOC499749
A_44_P376411	-0.489603	13.76028	0.007865	BF548067	

A_44_P885461	-0.491508	11.36357	0.002118		
A_44_P976153	-0.492456	13.56608	0.002154		
A_44_P792746	-0.494639	12.61698	0.003912		
A_43_P14760	-0.495239	8.143939	0.006653		
A_44_P578847	-0.497309	11.57353	0.005959		
A_44_P1031380	-0.49908	7.921169	0.009759	XM_001077462	
A_44_P808218	-0.499845	10.42192	0.006617	XM_233470	RGD1311937_predicted
A_44_P278659	-0.509675	11.62496	0.001616	AJ517196	
A_44_P988767	-0.510231	9.065142	0.004772	BF564195	
A_42_P475623	-0.519306	11.46603	0.005213	NM_053394	Klf5
A_44_P653818	-0.5195	11.96479	0.002595		
A_44_P254238	-0.519538	12.83616	0.005937	NM_001033670	Bcl2l1
A_44_P635089	-0.520099	14.54726	0.000703	AI030078	
A_44_P724632	-0.522737	10.62241	0.006501	AI176008	
A_44_P1026631	-0.523563	12.89669	0.008387	XM_573823	Akap11
A_44_P840348	-0.530728	11.49232	0.00838		
A_44_P559242	-0.532673	9.055327	0.004592	XM_001065920	
A_42_P570848	-0.544448	9.382288	0.000431	NM_017091	Pcsk1
A_44_P175677	-0.555101	7.45903	0.00369	XM_001062365	LOC685114
A_42_P788810	-0.562441	11.15106	0.008966	AW918709	
A_44_P837846	-0.574638	11.59704	0.005446	DV723320	
A_44_P895064	-0.576006	8.930311	0.00887	XM_341290	RGD1562692_predicted
A_44_P570046	-0.577647	10.78454	0.007289	XM_001067059	LOC314964
A_42_P502759	-0.58777	14.49019	0.00567	XM_343513	Aplp2
A_44_P249774	-0.594975	8.836396	0.009915	BG668003	LOC688621
A_44_P731912	-0.601613	10.58883	0.006816		
A_42_P638620	-0.608517	7.700495	0.003204	NM_130741	Lcn2
A_44_P181718	-0.61556	8.066556	0.003116	BI285476	
A_44_P521587	-0.616852	13.53308	0.00098	XM_224588	Arhgef3_predicted
A_44_P374708	-0.619325	11.886	0.0085	NM_198750	Cry1
A_44_P962074	-0.632751	9.587462	0.006478		
A_44_P551872	-0.637831	8.523936	0.009835	AI555745	
A_42_P556083	-0.641374	11.39813	0.009144	NM_013165	Cckbr
A_44_P492624	-0.643134	9.667534	0.000409	XM_340867	
A_44_P472989	-0.643421	10.67878	0.003461	NM_017232	Ptgs2
A_44_P199107	-0.65155	7.152713	0.003241	XM_345241	RGD1562545_predicted
A_44_P341298	-0.654523	10.7338	0.007667	BF396386	
A_43_P12535	-0.655373	13.32748	0.009243	NM_031135	Klf10
A_43_P14919	-0.660635	12.98223	0.002117	NM_019372	Ppm2c
A_43_P12283	-0.667059	12.80167	0.001419	NM_022668	Mog
A_44_P776423	-0.671757	12.77633	0.001579	NM_001017386	Tmem10
A_44_P559239	-0.692896	8.905563	0.004793	AW921223	
A_44_P1034541	-0.694564	12.09181	0.001515	NM_022602	Pim3
A_44_P254130	-0.701323	10.04192	0.000593	AA875032	
A_44_P337351	-0.707538	13.17046	0.00034	NM_001033883	Cxcl12
A_44_P593071	-0.709574	7.774681	0.002257		
A_44_P221885	-0.71164	11.43124	0.005104	BF565038	
A_44_P589346	-0.723727	11.5605	0.003934	DY471743	
A_42_P762508	-0.757283	9.97503	0.000872	NM_001034199	Nptx2

A_44_P670185	-0.758857	10.81712	0.00219		
A_44_P823528	-0.760627	10.71223	0.000555		
A_42_P681722	-0.767592	10.37324	0.00117	BF554576	
A_44_P870183	-0.771142	10.51029	0.007791		
A_42_P739860	-0.772039	11.50895	0.005963	NM_133578	Dusp5
A_44_P914438	-0.778529	10.16228	0.003109	NM_080782	Cdkn1a
A_44_P219026	-0.785466	10.94052	0.002205	NM_012513	Bdnf
A_44_P732510	-0.804474	8.797511	0.008231	DV722942	
A_44_P307971	-0.824418	12.49927	0.004772	L04739	
A_44_P807565	-0.830688	10.52499	0.002158		
A_44_P1029253	-0.914823	10.58408	0.002969	NM_012757	Mas1
A_44_P378959	-0.933023	11.30108	5.98E-06	AA859348	
A_44_P763434	-0.945217	10.5241	0.005537	XM_576579	RGD1560005_predicted
A_44_P437896	-0.953692	10.65054	1.17E-05	NM_012513	Bdnf
A_44_P303430	-0.964636	12.90879	5.44E-06	AW142654	
A_44_P916210	-0.990373	15.19466	0.001296		
A_44_P522524	-0.9989	9.086332	0.000509	NM_019328	Nr4a2
A_44_P931389	-1.006772	11.03869	0.002907	CA509267	
A_44_P1032732	-1.010129	9.622861	0.006732	NM_031740	B4galt6
A_42_P839593	-1.017323	11.81139	0.002422	AW142999	
A_44_P424723	-1.05196	11.14587	0.005727	NM_021693	Snf1lk
A_42_P484738	-1.143309	10.55331	0.000399	NM_022266	Ctgf
A_44_P512806	-1.18274	7.98503	0.000782	NM_153626	Npas4
A_44_P654444	-1.185412	12.51873	8.56E-06	DQ268830	Nr4a3
A_44_P365379	-1.251433	11.97297	0.000967	NM_001077640	Gadd45g
A_42_P594613	-1.298603	11.44392	0.000322	NM_053883	Dusp6
A_42_P540950	-1.311262	12.17803	0.004526	NM_001009541	Ier2
A_44_P233080	-1.323167	14.34415	9.76E-05	NM_012551	Egr1
A_44_P701809	-1.392299	9.465179	0.000511		
A_44_P591857	-1.393798	11.97394	0.000557		
A_42_P708068	-1.414561	13.68879	5.27E-05	NM_019137	Egr4
A_44_P560710	-1.449866	9.965458	0.000353		
A_44_P495480	-1.508251	10.05032	0.001411	NM_022197	Fos
A_43_P12619	-1.588268	12.07253	0.000459	NM_031628	Nr4a3
A_44_P212803	-1.612143	13.06959	0.002631	AF050659	
A_43_P12125	-1.615114	11.49742	0.003416	NM_021836	Junb
A_44_P218896	-1.65862	9.205401	0.006471	NM_031327	Cyr61
A_44_P398142	-1.690248	12.12949	0.002178	NM_017259	Btg2
A_44_P380835	-1.72043	11.95836	1.00E-05	AB003726	Homer1
A_44_P839581	-1.759771	11.1114	0.000537		
A_42_P682589	-1.881937	13.8678	0.000267	NM_024388	Nr4a1
A_43_P11932	-2.061968	9.928836	0.000533	NM_017352	Nr4a3
A_42_P750683	-2.096022	9.035922	0.000742	NM_031327	Cyr61
A_44_P118724	-2.159492	14.27586	4.76E-05	NM_019361	Arc
A_42_P473594	-2.43313	11.62043	3.64E-06	NM_053633	Egr2

Table S3

ID	logFC	AveExpr	P.Value	GenbankAccession	GeneSymbol
A_44_P839581	1.478474	11.1114	0.002504		
A_44_P157527	1.190031	9.92948	0.000759	BF285469	
A_44_P535706	1.064651	12.24392	0.008651	XM_216485	
A_42_P525886	1.056686	10.01648	0.004039	XM_236253	Crabp1
A_44_P262625	0.984094	10.4371	0.002967	XM_344424	
A_44_P217250	0.951383	10.90076	0.00608	NM_012949	Eno3
A_44_P478637	0.946374	8.477595	0.000206	BI274236	
A_42_P679251	0.920972	10.74394	0.00304	NM_031503	Ascl2
A_44_P931389	0.919886	11.03869	0.005691	CA509267	
A_44_P913689	0.915771	12.44902	0.003316		
A_43_P16887	0.914468	10.50225	0.006547	XM_215059	Eraf_predicted
A_44_P479890	0.912017	9.666256	0.000903	NM_153821	Prrx1
A_42_P793008	0.908714	15.02889	0.007719	NM_130432	Rps13
A_44_P231657	0.907535	10.83999	0.000947	XM_341779	Zfp628_predicted
A_43_P14752	0.898158	10.27264	0.000507	XM_001073940	LOC690274
A_44_P236566	0.897017	9.874991	0.000944	XM_230848	Neurl2_predicted
A_44_P671992	0.894022	11.10745	3.85E-05	XM_575387	RGD1566201_predicted
A_43_P16949	0.846619	9.382546	0.006384	XM_220884	Sgca_predicted
A_43_P11563	0.835843	9.280827	0.00022	NM_012784	Cnr1
A_44_P685508	0.821372	10.87674	0.005479		
A_44_P743576	0.820139	10.21146	0.001612	AW917533	
A_44_P1009948	0.806853	12.19187	0.002096	XM_220532	RGD1304587
A_44_P252855	0.803766	10.96382	0.000453		
A_44_P853254	0.800247	10.52673	0.005443		
A_44_P197290	0.792545	9.481667	0.001253	NM_021760	Col5a3
A_44_P588759	0.792232	10.76386	0.000334	BF563670	
A_44_P543813	0.776085	10.17043	0.005401	XM_228345	Ascc3_predicted
A_44_P974651	0.774859	10.43947	0.000564	XM_575260	RGD1564048_predicted
A_42_P635881	0.773147	11.75922	0.000466	NM_031782	Slc32a1
A_44_P558838	0.769348	9.104969	0.00724	BF417038	
A_44_P413324	0.763004	10.31502	0.00565	BF558477	
A_44_P307308	0.761818	9.460592	0.008579	XM_237568	LOC301725
A_43_P11044	0.760943	11.9497	0.003488	NM_001009632	G0s2
A_44_P409913	0.750951	8.847053	0.008251	XM_341043	
A_44_P685354	0.747361	8.734705	0.001885		
A_42_P596942	0.746022	8.598174	8.19E-05	NM_145682	Filip1
A_42_P568943	0.738116	8.151025	0.007983	XM_217167	RGD1311874_predicted
A_44_P592800	0.732669	10.93803	0.001284		
A_44_P233786	0.73062	14.31421	0.002362	NM_030997	Vgf
A_44_P461587	0.727787	10.34052	0.000607	XM_341847	RGD1311142_predicted
A_44_P808072	0.727337	10.73465	0.007333		
A_43_P11082	0.723487	10.05847	0.003035		
A_44_P304759	0.722884	9.750024	0.003913	XR_007322	RGD1563459_predicted
A_44_P670185	0.715761	10.81712	0.003476		
A_42_P814235	0.715392	9.823875	0.00957	NM_057124	P2ry6
A_44_P848951	0.708819	10.57781	0.002262	AW915522	
A_44_P222447	0.706019	14.60212	0.004094	CF979076	RGD1560394_predicted

A_44_P319238	0.705051	9.700374	0.003076	XM_345977	Cpne4_predicted
A_44_P589674	0.702823	9.794569	0.002936	AI406628	
A_44_P394279	0.701716	14.61949	0.008634	XM_001081777	RGD1560070_predicted
A_43_P19039	0.699688	11.21215	0.001006	XM_342487	RGD1560583_predicted
A_44_P1014527	0.698386	11.80935	0.007188	XM_216189	Alms1_predicted
A_44_P1000100	0.69182	10.41259	0.000131	XM_215666	Trim45_predicted
A_44_P117216	0.691555	9.702736	0.004375	NM_001025007	RGD1310852
A_44_P559239	0.69093	8.905563	0.004893	AW921223	
A_44_P620208	0.688642	11.0855	0.001347	BF549419	
A_44_P868418	0.683253	11.31543	0.002557	AI043758	
A_44_P748361	0.683244	9.811364	0.009237		
A_42_P797638	0.677852	11.19226	0.001471	XM_341839	Wtip_predicted
A_43_P12401	0.677561	9.473038	0.006205	NM_024142	Mepe
A_42_P596050	0.674306	9.845698	0.004973	NM_053572	Pcdh21
A_44_P435158	0.674159	8.895149	0.004429	AW917664	
A_44_P930681	0.673171	11.51947	0.003812	DV723298	
A_44_P882551	0.671501	11.07155	0.007595	XM_001072622	RGD1560871_predicted
A_42_P782490	0.669773	9.828156	0.005585	XM_226464	RGD1560268_predicted
A_44_P640931	0.668623	11.37124	0.001121		
A_44_P1049275	0.666814	10.60548	0.009591	DQ889715	
A_44_P272253	0.66654	9.507764	0.001977	XM_213864	LOC288978
A_44_P507773	0.661387	10.38264	0.009943	BG663014	RGD1565037_predicted
A_44_P971665	0.659826	11.41268	0.003752	AW916050	
A_43_P11476	0.656787	9.86656	0.003993	NM_012590	Inha
A_44_P668572	0.65317	10.08484	0.003757		
A_44_P900576	0.653048	9.196065	0.006286		
A_44_P996952	0.653002	9.262775	0.001239	NM_001039454	RGD1561028
A_44_P376112	0.652891	10.44574	0.00588	X72758	
A_44_P178503	0.652686	9.392082	0.007859	XM_221452	
A_44_P1034937	0.650992	12.38865	0.006503	NM_001011966	Smarcd3
A_44_P296473	0.648189	8.12823	0.003827	AI013718	
A_44_P623694	0.648094	9.491483	0.003318	XM_001067023	LOC688455
A_44_P358028	0.646793	10.30608	0.009916	CO569648	
A_44_P179338	0.646712	8.706902	0.007715	AI603168	Rps15
A_42_P740209	0.643911	10.52559	0.000482	NM_012601	Mpg
A_44_P669323	0.641396	9.487703	0.003317		
A_44_P500295	0.639834	8.53055	0.005152	BI274118	
A_42_P619403	0.638018	10.81318	0.00178	XM_341720	RGD1309879
A_44_P316898	0.636016	11.33567	0.002103	XM_213488	Higd1b_predicted
A_44_P324906	0.635202	11.11406	0.004427	AW142380	
A_43_P11634	0.633438	9.769668	0.004831	NM_012959	Gfra1
A_44_P753353	0.633317	8.537939	0.008049	CO401859	
A_44_P464065	0.632653	9.778917	0.008309	NM_001033687	Ushbp1
A_44_P729931	0.632187	10.18263	0.001685	XM_001061367	LOC682404
A_44_P808566	0.631451	9.344853	0.003337		
A_43_P11350	0.629225	9.91316	0.006281	NM_001009271	Nt5dc2
A_44_P637675	0.627415	11.75785	0.004746		
A_44_P1024315	0.625645	10.69359	0.006978	NM_031321	Slit3
A_42_P560984	0.625403	13.64446	0.000459	XM_574891	RGD1565641_predicted

A_44_P1048140	0.620488	10.50182	0.000329	AI072036	
A_43_P12205	0.619793	10.56946	0.007281	NM_022294	Eltd1
A_42_P558206	0.618407	11.05513	0.005686	AW144330	
A_44_P513232	0.618325	12.10898	0.003182	BC099181	
A_44_P747073	0.618093	8.869727	0.002865		
A_43_P11654	0.616996	11.29558	0.003532	NM_013007	Pnoc
A_44_P1007449	0.6148	11.20815	0.004405	XM_222190	RGD1310159_predicted
A_44_P1026706	0.612765	10.75157	0.003673	XM_001061644	RGD1560433_predicted
A_44_P900390	0.611891	10.72012	0.000772		
A_42_P735417	0.610726	9.364027	0.003617	NM_175756	Fcgr2b
A_44_P284079	0.610166	8.530775	0.005062		
A_44_P839432	0.609938	9.475047	0.003499		
A_44_P747182	0.60653	11.36896	0.005169		
A_44_P376411	0.606227	13.76028	0.001558	BF548067	
A_44_P559242	0.605856	9.055327	0.001664	XM_001065920	
A_42_P694208	0.602707	9.982905	0.00706	XM_216483	RGD1311126_predicted
A_43_P17094	0.602556	11.6708	0.005941	XM_575803	RGD1561916_predicted
A_44_P977637	0.599374	9.003673	0.001018		
A_43_P10036	0.598834	12.94357	0.004326		
A_43_P11441	0.598154	9.32848	0.006699	NM_012510	Atp4b
A_44_P113907	0.59688	10.14031	0.007975	NM_170787	Cmbp
A_42_P620042	0.594377	11.38184	0.009277	NM_032080	Gsk3b
A_43_P14264	0.593121	10.59142	0.003642	NM_053653	Vegfc
A_44_P311963	0.588438	10.50042	0.001069	XM_575939	RGD1560468_predicted
A_44_P420046	0.586908	8.398432	0.000493	NM_021669	Ghrl
A_44_P653264	0.586072	11.61992	0.004981		
A_44_P408050	0.585517	11.32287	0.007527	XM_343189	Ckap4_predicted
A_44_P200522	0.584708	8.435389	0.006355	AI171232	
A_44_P404737	0.584359	8.349892	0.006292	BQ208553	
A_44_P1031380	0.584111	7.921169	0.003226	XM_001077462	
A_43_P22042	0.583503	10.78119	0.009341	NM_001009353	Pla2g7
A_43_P17061	0.582069	10.14337	0.00096	XM_223729	Rnf3_predicted
A_44_P746478	0.581686	10.85794	0.009795		
A_42_P829349	0.580869	12.14343	0.001062	XM_223785	Comtd1_predicted
A_44_P1017035	0.580629	9.27318	0.003502	NM_019214	Slc26a4
A_44_P250575	0.580384	9.39954	0.009085	XM_214236	Nudt15_predicted
A_43_P13381	0.577117	10.26594	0.001837	NM_139326	Pomc
A_44_P1026631	0.577082	12.89669	0.004254	XM_573823	Akap11
A_44_P255837	0.576706	15.01845	0.004168	XR_007349	RGD1561996_predicted
A_44_P215917	0.573202	8.78396	0.009015	XM_344018	RGD1563504_predicted
A_44_P686665	0.566384	9.497307	0.007581		
A_44_P1036170	0.565807	10.90934	0.006795	XM_220770	Nle1_predicted
A_44_P434118	0.565666	8.471229	0.007235	XM_213408	Rhbdl4_predicted
A_42_P695770	0.565462	13.0292	0.000787	XM_214288	
A_42_P686756	0.564589	11.6427	0.008268	BC087706	
A_42_P781585	0.56446	10.69382	0.006588	NM_001037554	Bex4
A_44_P1027971	0.564132	10.09748	0.002595	XM_001068081	LOC688730
A_43_P15659	0.563997	11.5428	0.000662	XM_342062	
A_44_P457599	0.563541	9.073987	0.003797	NM_053832	Foxj1

A_43_P17945	0.56207	9.8905	0.006736	NM_001014006	F12
A_43_P18226	0.561037	9.822439	0.009187	XM_234942	Myo1f_predicted
A_44_P956958	0.560997	10.34486	0.00161	AW143890	
A_42_P747311	0.560893	10.13027	0.008442	NM_057204	Ptpn23
A_44_P533531	0.560063	7.749707	0.003019	AI406723	
A_44_P302265	0.558124	8.517348	0.00574	XM_001065833	
A_44_P1050510	0.55727	8.275122	0.001286	XM_001075804	LOC686809
A_44_P302383	0.557114	15.03616	0.006088	XR_006725	LOC288777
A_44_P273734	0.556905	11.77642	0.00382	BC090336	Man1a_predicted
A_44_P983188	0.555079	9.746672	0.005676	AA818818	
A_44_P128820	0.554639	9.493733	0.000674		
A_42_P454907	0.554121	8.79808	0.001381	NM_053644	Cdh23
A_43_P14870	0.553953	10.69843	0.008927	NM_001013224	Nmnat3
A_42_P771373	0.553627	15.02783	0.001801	NM_012614	Npy
A_44_P454065	0.552253	10.04517	0.00631	NM_019147	Jag1
A_44_P527154	0.552208	10.96466	0.002312	NM_001024282	RGD1564767_predicted
A_42_P550172	0.55146	10.77524	0.008243	XM_001072438	
A_44_P356679	0.550647	10.6521	0.002296	XM_342932	Atpbd1b_predicted
A_44_P186883	0.550626	10.1592	0.004351	NM_173103	Clcnkb
A_44_P731439	0.550347	9.554146	0.002267		
A_44_P1020748	0.549987	11.98345	0.002468	XM_343405	RGD1309779_predicted
A_43_P19791	0.549438	10.00081	0.007721	XM_343063	
A_43_P13996	0.548706	10.46704	0.007236		
A_42_P515028	0.548351	8.802646	0.007597	XM_574162	RGD1564431_predicted
A_44_P527089	0.5474	8.896843	0.004266	M11597	Calca
A_44_P117015	0.546888	14.60943	0.009864	XM_342664	Ndufb2_predicted
A_44_P454847	0.546823	12.30266	0.002103	XM_342460	Ptpmt1
A_42_P502768	0.543328	11.25355	0.009264	XM_001065468	LOC685841
A_44_P974137	0.541887	7.395577	0.0006	AI716115	
A_44_P354018	0.540368	13.14388	0.00538	XM_224779	RGD1559708_predicted
A_43_P16588	0.540157	12.47542	0.0032	XM_342414	Sh2d3c_predicted
A_44_P993027	0.538938	11.5777	0.00983	NM_017060	Hrasls3
A_44_P186860	0.537831	10.31388	0.007414	NM_030867	Nfkbib
A_44_P779143	0.537821	8.487288	0.00418		
A_44_P468661	0.536414	10.48671	0.003267	XM_001067949	LOC688966
A_42_P797965	0.535476	11.00195	0.00189	XM_341902	RGD1306959_predicted
A_44_P728951	0.534878	15.17816	0.006581	NM_031838	Rps2
A_44_P960935	0.5334	11.41158	0.002319		
A_44_P525649	0.533214	11.41392	0.003291		
A_44_P1020608	0.533079	9.273392	0.007964	XM_227821	Ifi44
A_44_P115509	0.532461	8.32335	0.003018	CN542475	Mrpl34
A_44_P901878	0.531861	8.805109	0.001812		
A_44_P389590	0.530125	8.795146	0.006664	XM_236348	Cilp_predicted
A_44_P1034346	0.527902	15.16222	0.002687	NM_001013922	RGD1307752
A_44_P996729	0.524978	9.473439	0.003588	NM_031054	Mmp2
A_44_P792646	0.524628	8.148467	0.006338		
A_44_P187641	0.524616	10.19557	0.009149	XM_342871	RGD1307610_predicted
A_42_P700089	0.52346	11.57458	0.009959	NM_001034157	Bat4
A_44_P791860	0.521772	9.427981	0.001731		

A_42_P501233	0.521734	12.36386	0.003728	NM_024483	Adra1d
A_44_P592844	0.521615	9.250689	0.000598		
A_44_P159613	0.521193	9.600495	0.006964	XM_344763	RGD1306894_predicted
A_43_P14871	0.520827	9.257777	0.002226	NM_021587	Ltbp1
A_44_P792446	0.520423	9.211865	0.009129	XM_235529	Scube1
A_44_P189859	0.517844	8.457089	0.006586	NM_001012094	Zfp297b
A_43_P22700	0.517808	8.510855	0.00613		
A_42_P578953	0.517652	9.465361	0.009175	XM_342416	Ptrh1_predicted
A_44_P210736	0.51718	10.06647	0.009054	XR_007818	RGD1563527_predicted
A_44_P668620	0.516418	10.83056	0.003444	XM_340915	Ccdc43
A_42_P837214	0.516128	9.812712	0.005553	NM_012861	Mgmt
A_44_P164848	0.514671	10.69432	0.007166	AW142951	
A_43_P10498	0.514272	11.03042	0.001276	XM_237039	
A_44_P421333	0.513769	8.574784	0.007005	NM_080778	Nr2f2
A_44_P122206	0.513365	15.15379	0.003959	NM_022541	Timm8b
A_44_P133485	0.512323	12.43585	0.003145	XM_342883	LOC362564
A_44_P337311	0.511511	7.975955	0.001121	XM_342172	Thbs4
A_44_P670043	0.510834	10.45987	0.007806		
A_44_P1026123	0.509573	12.01201	0.008494	XM_341806	
A_44_P978083	0.507157	10.46215	0.00409		
A_42_P737455	0.506634	12.01383	0.002142	AI071866	
A_44_P607632	0.505455	10.97934	0.004712	XM_001079243	LOC691678
A_44_P412236	0.505256	10.44247	0.004168	NM_053730	Stag3
A_44_P976153	0.504629	13.56608	0.001759		
A_44_P853624	0.503794	10.94544	0.003551	XM_237855	RGD1308321_predicted
A_44_P370645	0.503213	13.74896	0.007767	XM_216400	Sec61b_predicted
A_44_P561643	0.502716	8.03752	0.008948		
A_44_P274083	0.502042	11.44706	0.007485	NM_173105	Aqp11
A_44_P263854	0.500949	7.556899	0.002011	AA819299	
A_44_P997787	0.499178	11.68636	0.001984	XM_213799	RGD1562579_predicted
A_44_P215056	0.49916	15.24759	0.00311	NM_001007235	Itpr1
A_42_P621832	0.498006	13.25698	0.002695	XM_215083	RGD1306636_predicted
A_43_P12437	0.496721	9.648658	0.008789	NM_024385	Hhex
A_44_P513360	0.494845	8.485518	0.000889	XM_214152	Cdkn3_predicted
A_43_P14954	0.493343	11.66088	0.004046	XM_233820	Sos1
A_44_P447701	0.492105	10.46227	0.008867		
A_43_P19306	0.491696	10.80629	0.0087	XM_223660	RGD1309501_predicted
A_43_P19265	0.490977	10.98343	0.009847	XM_342507	RGD1562705_predicted
A_44_P330109	0.490805	10.01774	0.00451	NM_040669	Hps1
A_43_P10072	0.490183	10.93163	0.005158	NM_213564	Zbtb9
A_44_P241190	0.48927	10.80101	0.003973	NM_001013199	Sdccag10
A_42_P601961	0.487725	10.03823	0.00844	XM_225203	Susd3_predicted
A_44_P932158	0.487385	9.506271	0.005037		
A_42_P512838	0.487372	12.00659	0.004471	XM_217335	Trem2_predicted
A_44_P562712	0.486028	9.158063	0.005034		
A_44_P409232	0.485898	7.385279	0.003633	NM_031543	Cyp2e1
A_44_P370335	0.48549	11.42783	0.006794	NM_001011911	Lyar
A_44_P597013	0.484512	9.936548	0.002444		
A_44_P713589	0.482311	9.954375	0.008636		

A_44_P591963	0.481999	11.282	0.009105	DV728639	
A_44_P822852	0.480143	13.07186	0.008598	XM_001073919	LOC686393
A_43_P10505	0.479408	15.07648	0.007169	XM_226549	Jph3_predicted
A_44_P428922	0.479017	10.93142	0.009585	XM_341979	
A_42_P824657	0.47757	10.6343	0.002811	XM_214678	RGD1307357_predicted
A_44_P306626	0.476762	11.74221	0.007671	XM_577573	
A_44_P277669	0.47676	9.356253	0.008363	XM_573168	RGD1559961_predicted
A_42_P770330	0.47665	12.22638	0.008628	XM_001062632	LOC684934
A_44_P125145	0.476294	9.924844	0.006945	CF109884	
A_44_P387314	0.476035	8.528691	0.007087		
A_42_P738337	0.475807	13.2805	0.005505	NM_012575	Grin2c
A_43_P16816	0.47557	11.83034	0.009131	NM_001044290	LOC686883
A_44_P1022403	0.474294	13.72272	0.002583	NM_001004204	MGC94190
A_42_P506956	0.473183	10.23541	0.006511	NM_133559	Pcsk4
A_43_P17196	0.473149	10.88791	0.007969	XM_576184	RGD1562114_predicted
A_44_P1058112	0.473068	10.42333	0.002332	NM_001009627	Yars2
A_44_P578250	0.472654	10.84042	0.007706	DV714571	
A_44_P290327	0.471468	10.33899	0.008942	NM_001004234	MGC94881
A_44_P669746	0.471266	9.35851	0.001943	XM_229131	Cova1_predicted
A_44_P776556	0.470685	10.6081	0.007348	XM_342497	Chac1_predicted
A_44_P239176	0.468474	12.48785	0.009179	NM_001008380	RGD1308959
A_43_P14144	0.468252	12.01082	0.002547	XM_222855	Umpk_predicted
A_42_P700150	0.467273	11.80679	0.000671	BP483355	
A_44_P240414	0.466987	11.48117	0.005379	NM_001017537	Tex261
A_42_P515931	0.466948	11.1748	0.009094	XM_220256	
A_44_P225353	0.465362	10.46115	0.008862	NM_001009678	RGD1311458
A_44_P492624	0.465045	9.667534	0.006243	XM_340867	
A_44_P930534	0.464955	9.366294	0.00883	BC092660	RGD1563167_predicted
A_43_P11236	0.463992	12.00416	0.001889	NM_053538	Laptm5
A_44_P281457	0.463175	15.21279	0.006222	XM_001054883	LOC679899
A_44_P792746	0.462911	12.61698	0.006308		
A_43_P10692	0.461194	10.27534	0.009132		
A_44_P859124	0.460771	9.379251	0.006061	CB328239	LOC689165
A_44_P437346	0.460583	8.365901	0.002927	AW435442	
A_44_P368513	0.459819	9.534264	0.003137	BF282682	
A_44_P816687	0.457365	15.08238	0.006713		
A_44_P1008245	0.457175	10.86121	0.004652	XM_216310	Casc1_predicted
A_44_P997737	0.457138	10.4096	0.009265	AA942848	
A_44_P288443	0.456325	10.61669	0.002509	XM_344542	Thsd1_predicted
A_44_P472635	0.454909	7.530146	0.002444	AA900458	
A_44_P938669	0.454871	10.45325	0.007816	BC127463	Ng3
A_44_P496788	0.454063	11.58357	0.008877	NM_001008295	Fip1l1
A_42_P459306	0.453602	15.23919	0.009502	CB314148	RGD1305687_predicted
A_44_P1030801	0.453237	11.43176	0.005632	NM_031749	Gcs1
A_44_P557994	0.451938	15.05147	0.00241	XR_006555	LOC298125
A_44_P142472	0.451815	13.74517	0.002097	XM_214293	RGD1308064_predicted
A_44_P1027866	0.45081	10.85902	0.004562	NM_001011925	Nup93
A_44_P292419	0.449724	7.803599	0.006188	AA944408	
A_44_P1045652	0.449511	10.26325	0.009308	XM_001057185	LOC680266

A_44_P843798	0.448234	10.13852	0.00458	AW144049	
A_42_P792497	0.446787	8.957712	0.00776	NM_023025	Cyp2j4
A_44_P747708	0.445849	8.532038	0.006265		
A_42_P809733	0.445214	10.10656	0.009371	XM_347094	RGD1309410_predicted
A_44_P281216	0.444489	11.03937	0.00685	XM_341975	
A_42_P666843	0.44252	12.14138	0.000614	XM_214296	Pgls_predicted
A_44_P838483	0.439995	12.02493	0.003033		
A_44_P980178	0.439693	9.848248	0.007696		
A_42_P838051	0.439586	10.38087	0.002926	XM_216446	Oma1_predicted
A_44_P211997	0.436673	8.390802	0.00677	BF282863	
A_44_P381177	0.433194	8.439087	0.00677	AA925047	
A_44_P1058421	0.432785	11.85759	0.007566	BC091438	Phf12
A_44_P884119	0.432377	11.39608	0.007537	NM_001014061	LOC312863
A_44_P303883	0.431123	8.470756	0.007266	AI602844	
A_43_P17192	0.430405	11.86591	0.00643		
A_44_P274785	0.427234	11.39347	0.009019	XM_213260	RGD1310922_predicted
A_44_P1004260	0.426859	11.65237	0.009919	XM_231121	Tmem15_predicted
A_44_P147888	0.424227	7.149848	0.009323	AA925444	
A_43_P11580	0.423705	9.696657	0.007139	NM_012833	Abcc2
A_44_P402559	0.422607	7.597691	0.002504	NM_001000910	Olr1598_predicted
A_44_P307646	0.422312	7.041288	0.004813	BI289723	
A_44_P591370	0.421966	13.85891	0.006538		
A_44_P993577	0.42188	10.7227	0.005385	XM_214633	RGD1560538_predicted
A_44_P710192	0.419518	7.395678	0.007925	NM_001033067	Tmem45b
A_44_P251542	0.418155	7.329535	0.00136	NM_001008864	Tessp5
A_44_P1045612	0.416475	9.352711	0.005762	NM_172317	Fxyd3
A_44_P156317	0.413928	15.16467	0.005538	NM_138910	Dad1
A_42_P705083	0.413592	9.636382	0.00501	XM_341621	Il17b
A_44_P638411	0.411681	8.943508	0.009697		
A_44_P1030648	0.41131	11.38167	0.009437	XM_001079792	LOC687694
A_43_P23304	0.410921	11.02713	0.005687	NM_199397	Panx1
A_44_P476237	0.409138	7.158337	0.005359	BF406637	
A_44_P822839	0.408016	12.31907	0.007651		
A_44_P890053	0.407782	7.351377	0.005303	CA505010	
A_44_P899264	0.405945	11.22946	0.007897		
A_44_P421534	0.405341	12.30185	0.00563	NM_017196	Aif1
A_44_P981783	0.402686	9.048275	0.007562		
A_44_P558411	0.39837	12.48958	0.005443	NM_033499	Scrg1
A_42_P802520	0.396592	12.61219	0.008982	NM_172063	Pex14
A_44_P340498	0.396466	12.96412	0.007304	XM_213445	RGD1559720_predicted
A_44_P1050444	0.396311	10.22641	0.007523	NM_001007749	Atp5s
A_44_P229769	0.395256	15.30845	0.006174	NM_031838	Rps2
A_44_P140266	0.39507	8.238868	0.007651	XM_227523	Slc22a15_predicted
A_44_P562685	0.393179	8.369911	0.003369	XM_575917	RGD1564163_predicted
A_44_P810151	0.392701	11.82465	0.003282		
A_43_P14262	0.391785	13.5887	0.006879	XM_001057346	RGD1565310_predicted
A_44_P422602	0.390835	7.556121	0.007543	AI177360	
A_44_P667745	0.387442	13.15201	0.007663	DY311022	
A_44_P475617	0.386967	7.385824	0.004279	XM_238082	RGD1565487_predicted

A_43_P21364	0.383104	8.531841	0.007566	XM_343342	LOC363009
A_44_P399999	0.379395	15.41955	0.009292	NM_053752	Suclg1
A_44_P1002320	0.378477	13.9765	0.006397	BF289377	
A_44_P505188	0.378105	7.591275	0.009527	AI454498	
A_44_P378322	0.375598	9.393646	0.00534	AA858768	
A_44_P290016	0.375006	13.07897	0.009351	BG666843	
A_43_P22494	0.37441	8.369659	0.007185		
A_43_P16530	0.374106	13.06302	0.009245	XM_213733	Pop7_predicted
A_44_P1043668	0.372798	7.238479	0.006893	NM_001014003	RGD1307172
A_44_P1051894	0.368997	9.204886	0.007275	XR_009243	LOC363915
A_44_P1047992	0.367006	7.214531	0.008066	NM_012803	Proc
A_42_P685138	0.366955	11.97115	0.009234	NM_153621	Dab1
A_44_P351239	0.366576	7.624133	0.008737	NM_001000949	Olr179_predicted
A_43_P16804	0.363022	11.4628	0.007236	NM_001007652	Pole3
A_42_P626023	0.358798	10.46022	0.00991	XM_224232	Cenpj_predicted
A_44_P134465	0.350702	8.410751	0.0084	AA963226	
A_44_P257522	0.348981	8.174514	0.007883	XM_574228	Agrp
A_44_P178014	0.346895	7.08938	0.007953	BC079202	RGD1560814_predicted
A_44_P273515	0.339925	12.94672	0.0055	DV723169	
A_44_P108993	0.338519	7.542753	0.006345	BF420718	
A_43_P14576	0.33491	13.26406	0.006177		
A_44_P794680	0.332151	8.579136	0.009291		
A_44_P505488	0.329268	7.348394	0.008895	BI288152	
A_44_P591036	0.30765	7.029821	0.009164	XM_001069503	LOC685839
A_44_P388709	-0.277114	7.125181	0.00825	NM_145767	Prrxl1
A_44_P163408	-0.301364	7.336079	0.007371	NM_031798	Slc12a2
A_44_P370774	-0.30644	7.045656	0.009599	XM_235183	Irak3_predicted
A_44_P300110	-0.31546	8.120294	0.00754	XM_234335	Zfyve26_predicted
A_44_P515461	-0.317648	12.48507	0.00948	NM_171990	Bpnt1
A_44_P228903	-0.319342	7.646097	0.005616	NM_078621	Ccbp2
A_44_P349720	-0.326164	7.517157	0.006523	NM_001039174	LOC654482
A_44_P374795	-0.328038	7.277528	0.009295	XM_232197	Fbln2
A_44_P242252	-0.328341	13.95643	0.008558	NM_031237	Ube2d3
A_44_P557376	-0.334624	7.479056	0.009316	NM_012546	Drd1a
A_43_P18979	-0.337979	8.103409	0.009229	XM_340992	Rabl3_predicted
A_43_P16060	-0.338348	7.375756	0.005639	XM_001065759	Mitf
A_44_P332429	-0.34648	8.812568	0.004876	NM_138900	C1s
A_44_P274526	-0.347421	7.964663	0.002915	XM_230889	RGD1311378_predicted
A_44_P865552	-0.352072	7.495648	0.007668	AW143038	
A_44_P291081	-0.354249	10.2845	0.008309	XM_001054316	Mon2
A_44_P372555	-0.355726	10.97908	0.008972	NM_175764	Jmjd1a
A_44_P604860	-0.355913	8.288109	0.008249	BC098774	
A_44_P150432	-0.356004	7.38676	0.000845	D38557	
A_43_P15352	-0.360573	7.803403	0.006078	NM_080577	Nploc4
A_43_P18495	-0.362158	8.081096	0.009698	NM_001011952	Slc39a8
A_44_P304374	-0.363852	8.110134	0.007795	NM_001012745	Zfp422_predicted
A_44_P187388	-0.371358	7.568589	0.008114	XR_006961	LOC295077
A_44_P464428	-0.371729	7.097096	0.001069	XM_343279	Zfp647_predicted
A_43_P21231	-0.372047	8.054747	0.008386	XM_217594	Suv39h1_predicted

A_42_P703664	-0.372616	8.338728	0.00858	CV118897	Srxn1
A_44_P1010416	-0.372721	8.425505	0.002935	NM_001002853	P2ry13
A_44_P187524	-0.376345	13.29484	0.008423	XM_214690	Aars
A_44_P527635	-0.377348	7.727722	0.0087	NM_001014055	RGD1309682
A_44_P874814	-0.377382	8.871871	0.007677		
A_44_P437973	-0.377777	7.563555	0.005686	NM_020090	Slc24a1
A_44_P412418	-0.37849	7.41379	0.004835	XM_001063618	
A_43_P17282	-0.380701	7.370769	0.004136	XM_219879	Cnnm1_predicted
A_44_P180645	-0.382578	7.562432	0.006521	BC098851	Usp24_predicted
A_44_P333452	-0.385514	7.947414	0.004676	XM_347258	RGD1563309_predicted
A_44_P1000561	-0.386045	8.849037	0.007385	XM_235378	RGD1305781
A_44_P136911	-0.386216	13.38161	0.006216	BC078917	Rnpc2
A_43_P16885	-0.386816	8.04462	0.007478	XM_235169	Mdm2_predicted
A_44_P536125	-0.388142	9.259137	0.006564	NM_053557	Hrmt1l3
A_44_P656668	-0.388242	7.348888	0.009361	CK476135	
A_44_P154721	-0.389698	9.396214	0.006498	XM_220217	Zfp263_predicted
A_44_P116606	-0.392618	7.486812	0.007196	XM_342337	Ank2
A_43_P12267	-0.392704	7.482954	0.002535	NM_022623	Fzd4
A_44_P829047	-0.393483	8.964901	0.006918	CO402576	
A_44_P541431	-0.395296	8.013577	0.009828	NM_001024246	RGD1310794
A_44_P925410	-0.396807	9.196403	0.00307	BF386740	
A_43_P22351	-0.398613	8.784806	0.007162	NM_001024968	RGD1307594
A_43_P19646	-0.399039	7.740829	0.002969	XM_341833	
A_44_P984158	-0.40054	7.913112	0.002939	BI291259	LOC691759
A_43_P11729	-0.401196	7.849259	0.005334	NM_013134	Hmgcr
A_44_P184955	-0.402159	9.922687	0.009914	XM_215006	RGD1307366_predicted
A_44_P374341	-0.402413	11.9804	0.009791	NM_001029897	Strn3
A_44_P395572	-0.402616	7.391226	0.004891	NM_147210	Nr1d2
A_43_P18148	-0.402674	8.9781	0.008113	XM_218963	Arhgef17_predicted
A_44_P333374	-0.402932	7.958977	0.004709	XM_001064905	RGD1560736_predicted
A_44_P998042	-0.405032	7.829167	0.005607	XM_229579	RGD1309621
A_44_P128986	-0.406634	10.13576	0.006003	NM_001034959	LOC619574
A_44_P893828	-0.408401	8.447218	0.009765		
A_42_P584710	-0.409763	7.911257	0.005578	XM_213677	
A_44_P545649	-0.411763	7.755706	0.00159	NM_001014093	Parp16
A_44_P147980	-0.41267	8.163945	0.004793	XM_576503	
A_44_P126027	-0.414711	8.320375	0.002833	NM_019333	Pfkfb4
A_44_P149599	-0.415456	8.959757	0.008602	NM_001079705	RGD1311558_predicted
A_43_P22795	-0.416161	8.343222	0.009128	NM_001037204	Taf1a
A_44_P303716	-0.417343	9.839435	0.004084	XM_345266	Trim33_predicted
A_44_P100609	-0.418309	11.7365	0.008571	XM_001064153	
A_44_P655158	-0.418598	10.96723	0.00669	CO567276	
A_44_P337579	-0.41874	7.77436	0.007229	NM_001008346	RGD1308302
A_43_P16847	-0.419123	7.615653	0.004217	XM_233686	Spsb1_predicted
A_44_P523190	-0.420052	7.857785	0.004025	XM_343095	RGD1311756_predicted
A_44_P683082	-0.422342	8.705595	0.008957		
A_44_P473314	-0.424177	7.958854	0.0092	NM_001013997	RGD1309522
A_44_P729266	-0.425062	7.240329	0.00491	XR_005718	LOC300506
A_44_P357870	-0.425064	10.4228	0.00681	NM_031559	Cpt1a

A_43_P15840	-0.425124	8.202072	0.006167	XM_221874	
A_44_P166464	-0.425388	7.355895	0.005999	XM_235050	Scyl2_predicted
A_43_P15468	-0.426087	7.748036	0.004805	NM_012585	Htr1a
A_44_P549198	-0.426763	7.242243	0.007579	XM_224165	Ift88_predicted
A_44_P520700	-0.426846	10.3449	0.007187	XM_341377	RGD1561742_predicted
A_44_P986848	-0.427928	13.98555	0.004016	XM_341882	Sytl2_predicted
A_44_P206258	-0.430841	10.53316	0.008065	NM_001034146	Usp10
A_44_P123813	-0.43159	7.738666	0.005008	XM_223597	Cobl_predicted
A_44_P258277	-0.432043	11.92776	0.007726	NM_001008507	Dhrs7b
A_44_P304493	-0.432466	8.166562	0.00398	NM_001024970	RGD1305572
A_44_P668388	-0.433567	12.07127	0.009722	BC098022	RGD1561494_predicted
A_44_P1004392	-0.435274	12.93181	0.003053	XM_221455	LOC288109
A_44_P328654	-0.436421	11.70857	0.007688	XM_576882	LOC501474
A_43_P19234	-0.437806	11.46931	0.009135	NM_001034926	LOC310946
A_44_P1026191	-0.438058	10.88975	0.004056	NM_001039338	LOC362156
A_44_P116809	-0.439177	7.470634	0.000991	NM_001014136	Ngly1
A_44_P501242	-0.439216	8.149434	0.006859	XM_215947	Ncoa3
A_44_P327515	-0.440596	8.048777	0.003946	XM_001060307	LOC501515
A_44_P206154	-0.4407	7.880564	0.005502	XM_345231	RGD1307244_predicted
A_43_P10608	-0.442394	12.19876	0.002867	XR_006750	RGD1310444_predicted
A_44_P188869	-0.443051	7.32103	0.003535	AW915311	
A_44_P333550	-0.443265	7.827262	0.002488	NM_001013986	LOC305076
A_44_P360563	-0.443912	7.683515	0.003859	NM_017012	Grm5
A_44_P456747	-0.445211	8.694571	0.00459	AB012231	Nfib
A_43_P12243	-0.445373	13.92915	0.006547	NM_022540	Prdx3
A_44_P134186	-0.445505	12.47747	0.00625	NM_145678	Vps4a
A_44_P377722	-0.446034	12.68135	0.009816	NM_001007651	Peflin
A_44_P183227	-0.446446	9.436279	0.008026	CO394022	
A_44_P545867	-0.446549	8.205647	0.001163	XM_222229	RGD1309359_predicted
A_42_P574960	-0.447813	11.39518	0.001401	XR_009643	
A_44_P682849	-0.448246	8.37393	0.00533	XM_001081465	Rundc1_predicted
A_44_P462285	-0.450382	7.83441	0.00322	XM_342391	Inpp5e
A_44_P170864	-0.450423	10.55487	0.006427	XM_223651	RGD1311920_predicted
A_44_P729342	-0.451904	12.94046	0.007958	XR_009643	
A_44_P124027	-0.452323	8.094798	0.001562	XM_226458	RGD1309394_predicted
A_44_P193644	-0.452453	9.664843	0.00925	XM_001062296	RGD1561065_predicted
A_44_P466608	-0.454116	10.16095	0.008012	XM_238380	Usp5_predicted
A_43_P22288	-0.45513	8.782478	0.008509	XM_001068814	
A_44_P437721	-0.455167	9.310782	0.004106	CO566833	
A_44_P449819	-0.455646	8.385985	0.006492	NM_053851	Cacnb2
A_44_P443493	-0.45642	9.271581	0.007848	NM_001025754	Neil1
A_43_P17816	-0.456669	9.180063	0.00936	NM_199120	Cdv1
A_44_P544297	-0.457127	7.92615	0.004401	NM_001009661	Wbp11
A_44_P114184	-0.457582	10.4511	0.009192	NM_022543	Ssg1
A_44_P485592	-0.457681	12.21972	0.009729	XM_217388	Mrps9
A_44_P257832	-0.458486	8.387939	0.007793	XM_224535	Farp1_predicted
A_44_P174982	-0.458794	10.12484	0.004693	NM_022601	Pnpo
A_43_P10332	-0.460476	7.918233	0.004285	XR_009629	
A_44_P759334	-0.461876	12.22712	0.009711	NM_001011914	Dr1

A_44_P424069	-0.463971	7.490027	0.001269	XM_218463	Zfp84_predicted
A_44_P324034	-0.465472	8.71878	0.006468	XM_235942	RGD1308472_predicted
A_43_P19519	-0.465646	8.376732	0.001694	XM_223781	Vcl_predicted
A_43_P22080	-0.465665	8.304411	0.004401	NM_001012044	Lcp1
A_43_P15916	-0.465861	8.580545	0.006233	XM_342491	
A_44_P249717	-0.465922	7.787938	0.004592	AW144114	
A_43_P10414	-0.466482	9.127477	0.001963	NM_001017445	Ubxd8
A_44_P515792	-0.466818	8.010703	0.008513	XM_341532	Elmo1_predicted
A_44_P260475	-0.467015	10.55382	0.007242	XM_238362	Hdac11_predicted
A_44_P492629	-0.469771	11.97851	0.009798	DV727624	LOC497975
A_44_P412710	-0.469921	10.97259	0.005681	NM_001025423	Adhfe1
A_44_P211111	-0.470244	8.929899	0.007857	XM_236367	RGD1565416_predicted
A_44_P445521	-0.470433	13.10279	0.000946	XM_226843	Rnasen
A_44_P267501	-0.47192	7.687429	0.007344	NM_001017448	RGD1311358
A_44_P520557	-0.472441	9.851848	0.009165	XM_230284	Arhgap1_predicted
A_43_P21150	-0.472475	9.124359	0.007372	XM_345825	Tbc1d15
A_44_P484877	-0.472528	11.40675	0.009604	AF475093	
A_44_P283772	-0.47292	8.297396	0.005855	XM_573876	RGD1561342_predicted
A_44_P345136	-0.473156	8.837558	0.00698	XM_340879	Spag9_predicted
A_44_P625829	-0.474148	8.485355	0.009303		
A_44_P427067	-0.474709	11.6164	0.0082	XM_001076752	
A_43_P13424	-0.474807	9.092693	0.004906	NM_173290	Yy1
A_44_P231655	-0.476392	7.596331	0.007901	XM_222147	Glt1d1_predicted
A_44_P414704	-0.47772	8.234356	0.008052		
A_44_P397851	-0.477779	9.15503	0.007182	NM_053518	Lyst
A_44_P730048	-0.477978	10.56896	0.008411		
A_43_P21938	-0.478192	8.638288	0.008101	XM_220541	Ulk2_predicted
A_44_P381842	-0.479346	8.259924	0.009669	XM_343640	Ptprm
A_44_P1058502	-0.479398	8.486831	0.004314	XM_238162	Ppfia1_predicted
A_44_P463973	-0.479937	10.936	0.006261	NM_001079889	Sephs2
A_44_P126498	-0.479954	8.848829	0.009172	XM_234272	Klhdc1_predicted
A_43_P15409	-0.480053	8.148761	0.006225	NM_023971	Drp2
A_44_P384833	-0.480229	7.833966	0.007021	XM_343166	Mum1_predicted
A_44_P324124	-0.480593	10.55514	0.009972	XM_220595	RGD1563106_predicted
A_44_P350098	-0.480639	8.884507	0.006151	XM_345938	Alg9_predicted
A_44_P107177	-0.481244	7.566365	0.008674	NM_017018	Hrh1
A_42_P645142	-0.483451	11.32202	0.008261	NM_019375	Sep.03
A_44_P356445	-0.483861	7.658623	0.002629	XM_227547	Lrig2_predicted
A_44_P376905	-0.484469	12.72706	0.003147	XM_214994	Spcs2_predicted
A_44_P532020	-0.484935	8.716298	0.008881	XM_219414	RGD1306820_predicted
A_44_P501220	-0.485339	7.802926	0.003192	NM_053951	Mcf2l
A_44_P331109	-0.485528	8.957026	0.006251	XM_220175	
A_44_P105229	-0.485553	10.16727	0.004021	NM_001013157	Nnt
A_44_P664802	-0.485909	12.15639	0.007649	DV728182	
A_44_P136845	-0.486006	12.40366	0.00595	XM_340879	Spag9_predicted
A_44_P397495	-0.487804	12.14965	0.00402	NM_024359	Hif1a
A_44_P311955	-0.488411	8.390355	0.004453	NM_017171	Prkce
A_42_P765031	-0.48852	8.84223	0.007803	XM_346083	
A_44_P480830	-0.489345	8.330588	0.005378	NM_001079943	Zfp426

A_44_P661516	-0.490505	7.975856	0.002516		
A_43_P18324	-0.490957	7.896779	0.0057	NM_207165	Arl10
A_44_P215931	-0.491066	7.225432	0.004447	AI232088	
A_44_P501992	-0.491468	12.71414	0.003475	BC071175	Ero1l
A_44_P693682	-0.49161	10.28309	0.008944		
A_43_P12802	-0.491629	12.46216	0.002589	NM_053418	Ufd1l
A_43_P18343	-0.492117	10.27197	0.005068	XM_001072617	Gga2
A_44_P299123	-0.492405	7.997765	0.003786	XM_215847	Nphp1_predicted
A_44_P301512	-0.492541	11.62567	0.003021	NM_176077	G6pc3
A_42_P612592	-0.495016	7.834775	0.002747	XM_001067905	Saps3_predicted
A_44_P583834	-0.495725	11.18116	0.005538	XM_223597	Cobl_predicted
A_44_P534113	-0.496145	8.15518	0.008216	NM_053866	Plaa
A_44_P409977	-0.496805	8.586221	0.003235	NM_001012742	Wee1
A_44_P403816	-0.497337	10.19456	0.002825	AW142588	
A_44_P1057763	-0.497353	14.89762	0.00726	NM_053576	Prdx6
A_44_P768438	-0.498157	15.05002	0.00177	XM_225314	Vmp_predicted
A_44_P539167	-0.498327	7.569368	0.001016	XR_009059	LOC317276
A_44_P729229	-0.498505	8.042134	0.00752	XR_009322	LOC366863
A_44_P210753	-0.499432	9.573971	0.006112	M22631	Pcca
A_44_P935947	-0.499675	9.102805	0.004718	XM_342684	Hnrpa2b1_predicted
A_44_P303044	-0.499955	8.694465	0.006585	XM_239258	Supt6h
A_44_P555760	-0.50031	7.670131	0.002044	XM_001064688	LOC257646
A_44_P286467	-0.50037	9.413313	0.009875	XM_237056	RGD1309266_predicted
A_44_P388973	-0.501101	14.59061	0.004797	NM_012571	Got1
A_44_P123102	-0.501148	8.015902	0.00321	NM_001039607	LOC294154
A_44_P293438	-0.502716	12.33671	0.00823	XM_340949	RGD1311925_predicted
A_44_P993382	-0.503389	10.06768	0.009282	NM_031121	Ssrp1
A_44_P351490	-0.504828	10.77395	0.000536	NM_031797	Cd82
A_44_P370213	-0.505052	14.71043	0.007415	NM_012749	Ncl
A_43_P16583	-0.505052	11.68119	0.002588	XM_214539	Snx2_predicted
A_44_P400520	-0.505512	11.05271	0.004681	NM_012866	Nfyc
A_44_P133464	-0.511164	8.223828	0.008518		
A_43_P23298	-0.511238	8.463371	0.000938	XM_001079737	RGD1311730_predicted
A_44_P281750	-0.511574	12.76908	0.004393	XM_237790	Poldip2_predicted
A_44_P533572	-0.511838	8.138074	0.003513	AI639343	
A_44_P292900	-0.512225	7.828978	0.007812	XM_219377	LOC309016
A_44_P340180	-0.51227	9.585392	0.00796	XM_341668	Anapc10_predicted
A_44_P1048296	-0.512554	13.82163	0.005698	NM_172023	Osbpl1a
A_44_P238019	-0.513936	9.708293	0.005888	AW143671	
A_43_P21191	-0.513978	8.21006	0.001345	XM_234988	Btbd11_predicted
A_44_P1034030	-0.514586	8.979437	0.008022	XM_220420	Aff4_predicted
A_44_P290424	-0.51475	10.67252	0.004908	NM_080905	Siah1a
A_44_P461678	-0.515057	8.811745	0.002391	XM_574677	RGD1564887_predicted
A_44_P363522	-0.515107	10.42892	0.005067	NM_001031661	B4galt7
A_43_P23210	-0.515252	11.99024	0.00817	NM_001012153	Fmip
A_44_P477633	-0.516537	9.815629	0.007468	NM_174864	Ncstn
A_44_P226906	-0.517049	7.231693	0.004884	NM_147205	St6gal1
A_44_P540279	-0.517501	11.62786	0.00369	AW144704	
A_44_P377698	-0.518017	8.032468	0.004629	XR_006438	LOC303083

A_44_P157009	-0.518179	7.915851	0.008623	XM_232995	Rnf20_predicted
A_44_P445369	-0.518198	8.410379	0.005173	XM_001080476	LOC360824
A_44_P520929	-0.51833	8.270723	0.001288	NM_001007675	C1qtnf1
A_44_P208566	-0.520218	8.194993	0.000909	XM_341567	Plxdc2_predicted
A_44_P301875	-0.521017	8.537786	0.005085	XM_343761	Wdr44
A_43_P10744	-0.521209	11.8158	0.00767	NM_001014212	RGD1309784
A_43_P16724	-0.521403	8.927668	0.008353	NM_001014188	RGD1307688
A_44_P974892	-0.521521	8.218009	0.000906		RGD1560103_predicted
A_43_P18451	-0.521667	10.50442	0.007196	NM_080579	Gpr19
A_44_P342730	-0.521799	9.506206	0.005248	NM_001012275	Arih2_predicted
A_44_P362498	-0.523592	9.057442	0.008193	AW143788	
A_44_P255678	-0.523681	10.04796	0.004349	XM_228777	RGD1564628_predicted
A_43_P13675	-0.524202	9.004544	0.003831	CO570281	
A_44_P150877	-0.525453	9.650493	0.008311	XM_001080836	RGD1560454_predicted
A_44_P109834	-0.526057	7.550092	0.000686	XM_341783	Rdh13_predicted
A_42_P644929	-0.527818	13.23495	0.009106	NM_021579	Nxf1
A_44_P171155	-0.527957	12.38644	0.002943	NM_001014221	LOC363337
A_44_P142843	-0.528222	8.161509	0.003931	XM_001065442	LOC685834
A_43_P17871	-0.529071	9.583896	0.008044	NM_001013186	Bin3
A_44_P940553	-0.529771	14.44724	0.005633	AW143148	
A_43_P21003	-0.529928	10.93669	0.009913	NM_001033892	RGD1306841
A_44_P651694	-0.530167	7.349954	0.003359		
A_44_P776937	-0.530279	8.932346	0.00542	CR754170	
A_44_P227201	-0.530711	8.205059	0.002803	XM_225013	Tubgcp3
A_43_P10797	-0.532261	13.40048	0.001705	BG666794	
A_44_P471691	-0.532338	8.448807	0.000262	XM_223781	Vcl_predicted
A_44_P378225	-0.532628	11.11796	0.003763	NM_001011993	Pycrl
A_44_P315714	-0.535232	9.634824	0.009022	NM_021577	Asl
A_44_P683068	-0.535246	8.540939	0.005211	NM_017032	Pde4d
A_44_P260055	-0.53652	10.82252	0.004399	NM_031043	Gyg1
A_42_P750029	-0.537118	10.17965	0.001934	BF559198	
A_44_P490460	-0.537239	8.583732	0.001709	XM_224518	Tgds_predicted
A_44_P230960	-0.5381	12.36711	0.005794	AW144271	
A_44_P415459	-0.538541	9.588119	0.007499	CB547990	
A_44_P228874	-0.538818	9.251096	0.007028	NM_012815	Gclc
A_44_P516073	-0.539135	9.313478	0.001599	XM_217194	RGD1310552_predicted
A_44_P223808	-0.540022	11.92907	0.003404	XM_225711	
A_44_P170338	-0.540274	7.955337	0.009628	NM_001002815	Ece2
A_44_P485614	-0.540413	8.787416	0.00262		
A_44_P241371	-0.54044	9.019803	0.007357	NM_001013161	Cstf1
A_44_P175543	-0.541018	8.534275	0.005918	NM_001033699	Cox15
A_44_P579630	-0.541041	9.037196	0.009716		
A_44_P515211	-0.541293	9.195753	0.005306	NM_145765	Tnfsf15
A_44_P466183	-0.541305	8.38538	0.006606	NM_133410	Rap2b
A_44_P168778	-0.541812	8.029785	0.008427	XM_233220	LOC313391
A_44_P201601	-0.542679	9.320256	0.006957	XM_216096	
A_44_P180080	-0.54313	13.22102	0.002966	NM_022867	Map1lc3b
A_44_P353927	-0.543168	8.204865	0.002782	XM_341931	RGD1564195_predicted
A_44_P344340	-0.543214	8.425997	0.006536	U78135	Zfoc1

A_44_P310949	-0.543425	11.20006	0.004972	NM_001004283	Eif3s7
A_43_P12473	-0.543679	8.782044	0.000727	NM_031006	Adar
A_44_P207215	-0.543992	8.44599	0.001323	XM_237115	Nck2_predicted
A_44_P281155	-0.544066	7.616002	0.008359	XM_341127	Nek7_predicted
A_44_P323729	-0.544773	7.638288	0.004219	NM_001042621	Pip5k1a_predicted
A_43_P10248	-0.544913	13.09991	0.007402	NM_001014117	Ublcp1
A_44_P147622	-0.545175	10.55648	0.001064	XM_343462	Dnajc13_predicted
A_44_P386375	-0.545382	10.05504	0.004797	NM_053842	Mapk1
A_44_P333232	-0.545453	7.603713	0.001496	XM_342803	
A_44_P265326	-0.545734	8.11399	0.003644	BC087674	Pus3_predicted
A_43_P19260	-0.546023	9.074997	0.004115	XM_001053368	RGD1559803_predicted
A_44_P525101	-0.546161	7.790761	0.001773	XR_006056	
A_44_P534508	-0.546585	8.026102	0.003988	XM_231749	Zfp212
A_44_P447514	-0.546908	9.609403	0.003516	NM_017155	Adora1
A_44_P493688	-0.547009	12.20581	0.001709	NM_001008371	RGD1309216
A_44_P587322	-0.547841	13.30776	0.002382	AW917250	
A_43_P15866	-0.548306	9.016571	0.002132	XM_341686	Ap1g1
A_43_P21374	-0.548726	8.795816	0.003865	NM_001008353	Mkks
A_44_P557474	-0.549682	9.075636	0.006461	NM_053742	Pitpnb
A_44_P191022	-0.549841	11.25682	0.006775	NM_053674	Phyh
A_44_P338068	-0.550111	9.971705	0.002162	NM_001024355	LOC500909
A_44_P331003	-0.551713	8.367306	0.002003	XM_234281	
A_44_P222603	-0.551891	8.122971	0.004433	XM_341580	
A_44_P995544	-0.552105	10.31184	0.006155	NM_001039034	Wdr5
A_44_P241781	-0.552115	9.261364	0.004623	BF558420	
A_44_P228715	-0.552514	9.397023	0.000278	NM_019275	Smad4
A_44_P803129	-0.552556	8.623957	0.002243	AW918772	
A_44_P352104	-0.552676	11.45537	0.00664	XM_216993	Slc25a17_predicted
A_44_P168083	-0.553081	12.95714	0.005519	NM_019381	Tegt
A_44_P443228	-0.554142	10.05594	0.005989	XM_341622	Fech_predicted
A_43_P21031	-0.554515	8.256688	0.002089	XM_232343	Pex5_predicted
A_44_P171128	-0.55544	10.96284	0.00987	XM_232987	Alg2
A_44_P160550	-0.555742	12.55467	0.008317	NM_017116	Capn2
A_44_P349689	-0.555915	13.50737	0.009107	NM_001009831	Spg3a
A_44_P550670	-0.556316	11.92568	0.005809	XM_223611	Tmed4_predicted
A_44_P454426	-0.556338	8.18265	0.002798	AF133906	
A_43_P15280	-0.556496	8.112504	0.001725	NM_019194	Tef
A_43_P21715	-0.557461	8.881928	0.008605	BC091390	RGD1305062
A_44_P147582	-0.558181	7.806964	0.004217	NM_001079936	Rab5b_predicted
A_44_P173345	-0.558596	7.899522	0.002954	XM_342595	Stx16_predicted
A_44_P381483	-0.558686	11.69825	0.003748	XM_213930	
A_44_P167403	-0.558725	9.628788	0.006902	AA875633	
A_44_P191924	-0.559349	10.83352	0.006013	NM_019906	Frap1
A_43_P20321	-0.559792	8.491448	0.000581	XM_228770	Ccdc22_predicted
A_44_P744309	-0.560586	10.48679	0.005371	XM_222534	LOC683847
A_44_P289630	-0.562386	13.30648	0.005131	NM_017261	Gria2
A_44_P1019326	-0.562708	14.64606	0.007105	NM_199372	Eif4a1
A_44_P292479	-0.563031	12.45133	0.007171	NM_053880	Dncic2
A_44_P146833	-0.563782	11.62293	0.007793	M92076	Grm3

A_44_P284462	-0.563948	12.82861	0.003027	NM_031728	Snap91
A_44_P529934	-0.56395	8.644282	0.007049	XM_225661	RGD1566269_predicted
A_43_P16274	-0.564297	11.44811	0.004857	S53987	Chrna7
A_43_P16927	-0.564312	8.495799	0.003682	XM_228073	Pofut2_predicted
A_43_P19892	-0.564842	10.33035	0.004367	NM_001014255	Aph1a
A_44_P147062	-0.564927	9.477962	0.008194	XM_220095	Moxd1
A_42_P627394	-0.564957	13.05839	0.000589	BC089998	
A_44_P299699	-0.565034	9.391809	0.005983	NM_001013896	RGD1309948
A_44_P309513	-0.566123	8.088215	0.003308	NM_001024367	LOC501619
A_44_P207634	-0.566195	14.25058	0.008942	NM_001014148	LOC361309
A_44_P618177	-0.566587	12.8748	0.003863	CF110664	
A_44_P156805	-0.566739	8.099318	0.008471	XM_227475	Pogz_predicted
A_44_P280759	-0.568056	14.37983	0.007818	NM_012992	Npm1
A_44_P212355	-0.56835	9.738948	0.002238	XM_232466	Lrp6_predicted
A_44_P468544	-0.568435	9.993473	0.004454	NM_001013126	Cyb5r1
A_44_P738643	-0.568634	9.591004	0.003566		
A_44_P348973	-0.568895	8.505955	0.006712	NM_001004277	Lypla3
A_44_P261883	-0.569063	11.145	0.008299	NM_134402	Bzw2
A_44_P650648	-0.570226	10.93273	0.000434	BF561546	
A_44_P1060064	-0.570935	9.82548	0.005238	NM_001024883	RGD1310810
A_44_P409866	-0.572035	8.822675	0.003034	NM_013221	Hbp1
A_44_P347771	-0.572246	12.42882	0.002978	CB579735	
A_44_P384688	-0.572632	13.66295	0.002533		
A_44_P356027	-0.572935	12.00952	0.008344	NM_053642	Sc5d
A_44_P249606	-0.573078	7.971547	0.008649	NM_001025718	MGC112830
A_43_P15129	-0.573778	7.558528	0.000289	BF558849	
A_44_P137356	-0.574199	13.12717	0.009568	NM_053523	Herpud1
A_44_P550497	-0.57495	8.939273	0.002301	NM_022684	Bid
A_44_P365765	-0.575656	8.050544	0.005348	XM_224579	Slmap_predicted
A_44_P190115	-0.576073	9.257267	0.002651	XM_235940	Snx19_predicted
A_43_P19005	-0.576291	8.928856	0.008555	XM_574285	RGD1566426_predicted
A_44_P229352	-0.577022	8.451554	0.005411	XM_222717	Rgl1_predicted
A_44_P729514	-0.577376	9.689644	0.009895	XM_573199	RGD1560049_predicted
A_44_P130849	-0.577743	8.230187	0.008975	XM_224627	
A_44_P534249	-0.578016	14.39865	0.009912	NM_001013874	Ociad1
A_44_P707011	-0.578591	8.762253	0.003167	CO393635	
A_42_P747877	-0.57873	10.76747	0.00304	NM_172029	Eif2b1
A_44_P101223	-0.579038	10.35117	0.001145	XM_344744	Gcdh_predicted
A_44_P246311	-0.580416	10.15275	0.006615	NM_001017473	MGC112682
A_44_P548450	-0.580709	10.99874	0.007588	XM_225160	
A_44_P356596	-0.581185	10.9493	0.008387	XM_342783	RGD1304592_predicted
A_44_P368170	-0.581794	8.855102	0.001661	XM_342563	Actr5_predicted
A_44_P987846	-0.581855	11.16986	0.006395	XM_001075075	Dpp8_predicted
A_44_P246554	-0.582001	8.905353	0.003826	CO566833	
A_44_P463557	-0.582634	14.19713	0.004298	NM_013067	Rpn1
A_44_P231583	-0.583034	10.75149	0.007279	NM_032462	Csen
A_44_P130732	-0.583815	8.713516	0.003228	XM_001064066	LOC682999
A_43_P15355	-0.584685	13.54511	0.007016	NM_130755	Cs
A_44_P235090	-0.586284	8.863998	0.005913	XM_234988	Btbd11_predicted

A_44_P271989	-0.586374	11.34059	0.0094	XM_345169	Zfr
A_44_P170070	-0.586507	9.614184	0.004003	XM_236380	Rnf111_predicted
A_44_P810365	-0.586805	9.726458	0.009332	CV104092	
A_44_P382611	-0.587121	10.96644	0.00759	NM_001031651	RGD1304758
A_44_P444039	-0.58729	8.409482	0.006954	XM_215897	
A_44_P637334	-0.587827	10.97686	0.005035		
A_44_P262569	-0.58791	8.076643	0.001623	XM_341961	Tollip_predicted
A_44_P344730	-0.588025	7.880086	0.001613	NM_001009405	Arhgap29
A_44_P103692	-0.589224	10.07171	0.008032	XM_234484	RGD1309059_predicted
A_44_P698466	-0.58941	8.937917	0.008468	XM_001062636	
A_44_P504298	-0.589507	8.821758	0.007981	XM_221077	Cdc42ep4_predicted
A_44_P509129	-0.589766	8.808838	0.009171	BC083806	Tex2
A_42_P744464	-0.590185	8.758601	0.004669	XM_340740	RGD1565449_predicted
A_43_P17175	-0.590299	8.564087	0.001764	NM_001031642	Serpinb1a
A_43_P21386	-0.59044	8.419719	0.004713	CB546740	
A_43_P11905	-0.590826	9.270507	0.006217	NM_017303	Kcnab1
A_44_P471386	-0.591807	13.18609	0.008934	NM_001013107	Dazap2
A_44_P530041	-0.592177	9.105207	0.007566	XM_343804	Hdac8_predicted
A_44_P529718	-0.592568	7.591056	0.00106	XM_341914	RGD1310509_predicted
A_44_P237220	-0.592591	9.322389	0.000271	XM_214720	
A_44_P398587	-0.592826	11.00259	0.006479	XM_343766	Usp9x_predicted
A_44_P292327	-0.593308	11.46625	0.006377	NM_017006	G6pdx
A_44_P782486	-0.593503	7.65762	0.00356	XM_575380	RGD1563612_predicted
A_44_P508462	-0.593522	11.23446	0.009071	NM_131911	Anp32b
A_44_P222011	-0.593914	13.04026	0.005138	NM_017343	Mrlcb
A_44_P312371	-0.594586	12.02887	0.0022	NM_172066	Slc30a4
A_43_P21821	-0.595729	8.545106	0.004644	CB547114	
A_44_P222353	-0.596088	8.508683	0.001984	NM_001040128	RGD1309546_predicted
A_44_P217462	-0.596111	8.18643	0.002077	U78138	
A_43_P13557	-0.597345	13.14417	0.002976	CF110620	
A_44_P226353	-0.598015	11.01006	0.004433	NM_031672	Slc15a2
A_44_P438269	-0.598109	9.857028	0.000911	NM_022585	Azin1
A_44_P1009692	-0.598298	13.31779	0.004267	NM_001025624	Hmgn2
A_43_P12191	-0.598708	12.60738	0.00218	NM_022265	Pdcd4
A_44_P302996	-0.599223	11.48023	0.002285	XM_342171	RGD1562968_predicted
A_44_P461489	-0.599266	8.962531	0.00937	XM_231528	RGD1559440_predicted
A_44_P686863	-0.599609	11.85384	0.001206		
A_43_P18281	-0.599651	8.903045	0.000566	XM_001057305	
A_44_P703930	-0.600012	8.125062	0.002394		
A_44_P955997	-0.600108	11.43887	0.002913	DN932367	
A_44_P327535	-0.6004	8.105457	0.001482	XR_007094	LOC691397
A_44_P1049827	-0.600752	13.66577	0.008886	NM_024128	Nsg1
A_44_P516958	-0.601174	11.62942	0.004807	EF076766	
A_43_P10835	-0.601231	11.4609	0.008636	BP482727	RGD1308816_predicted
A_44_P823303	-0.601517	7.675391	0.001111		
A_44_P186813	-0.602333	8.109993	0.004622	XM_213276	Rars_predicted
A_44_P128711	-0.602335	7.768201	0.008573	XM_217566	Sh3bgrl_predicted
A_43_P18294	-0.60239	7.598985	0.000823	BC099835	
A_44_P326176	-0.602682	9.917051	0.005607	XM_222244	RGD1563628_predicted

A_44_P217909	-0.602949	8.417672	0.002581	XM_228644	Mtmr1_predicted
A_44_P851961	-0.603248	8.122635	0.00924	NM_001009504	Fbxl16
A_44_P325189	-0.603659	13.30424	0.000582	NM_175578	Dscr1l1
A_44_P789739	-0.60378	10.19794	0.002178	NM_001030024	Slc19a2
A_42_P688027	-0.606511	9.303765	0.005323	NM_030999	Crhr1
A_44_P114298	-0.606513	8.87185	0.002852	XM_215693	Hiat1_predicted
A_44_P344096	-0.606913	9.736673	0.009398	NM_138890	Ehd3
A_43_P10536	-0.607958	8.086298	0.007905	XR_007603	
A_44_P917935	-0.607991	8.678929	0.007689		
A_44_P377653	-0.608356	9.62169	0.001543		
A_43_P17872	-0.608383	10.81552	0.006759	CB545618	
A_44_P142318	-0.608411	10.34125	0.006816	XM_222171	Anapc7_predicted
A_44_P400936	-0.608664	12.47592	0.006637	XR_006350	LOC314123
A_44_P735345	-0.6091	7.725892	0.001624	XM_576015	RGD1560812_predicted
A_44_P495832	-0.610195	7.98717	0.001613	XM_343442	Pgm3_predicted
A_44_P356711	-0.610563	7.862104	0.006197	XM_343094	Ttc7b_predicted
A_44_P702685	-0.61115	11.2384	0.006725	CO402564	
A_43_P18909	-0.611317	9.101784	0.006293	XM_236648	Kif9_predicted
A_44_P809536	-0.611648	9.018063	0.002691	AW144200	
A_44_P475932	-0.612343	9.506439	0.008437	NM_001037496	Otud5
A_44_P465188	-0.613837	12.99403	0.006046	NM_053445	Fads1
A_44_P192018	-0.613883	7.703809	0.000494		
A_44_P354806	-0.614498	11.63019	0.005472	NM_172072	Vps45
A_44_P477135	-0.615226	8.68826	0.002682	NM_052801	Vhl
A_44_P513246	-0.615322	8.481193	0.004191	XM_341091	Fbxo21_predicted
A_44_P348983	-0.615971	8.261497	0.007893	XM_340889	Npepps
A_44_P671422	-0.616393	10.93854	0.004166		
A_43_P18993	-0.616672	10.24685	0.007665	XM_216679	Lamb1_predicted
A_44_P261179	-0.616735	9.356162	0.001222	XM_343177	LOC362845
A_44_P384223	-0.617224	12.50624	0.00345	NM_024151	Arf4
A_44_P358664	-0.617255	10.2275	0.009024	XM_223454	Stim2_predicted
A_44_P418824	-0.617423	9.363052	0.001671	NM_053430	Fen1
A_44_P253196	-0.617866	10.6956	0.001838	XM_228114	Arid5b_predicted
A_43_P18795	-0.618039	10.72011	0.008233	NM_001047849	Agpat6
A_44_P108680	-0.618465	8.102587	0.002297	NM_057116	Ppp2r2c
A_44_P396772	-0.619342	10.46003	0.001217	NM_001037363	Lrrn1
A_44_P457129	-0.619549	8.743385	0.006961	XM_227809	LOC310958
A_44_P301812	-0.619841	11.17309	0.004013	NM_001005765	Rap1a
A_44_P183048	-0.620055	10.77672	0.002498	XM_233467	
A_44_P199358	-0.620299	10.4968	0.007816	XM_229162	RGD1561327_predicted
A_43_P12127	-0.620811	14.8234	0.006163	NM_021842	Ensa
A_44_P526729	-0.62244	9.842671	0.006342	NM_031135	Klf10
A_44_P240973	-0.622495	7.998083	0.00086	NM_022264	Kit
A_44_P370761	-0.622587	8.463284	0.003614	XM_236009	Nfrkb_predicted
A_44_P135944	-0.62263	11.26265	0.008567	XM_220178	Adcy9_predicted
A_44_P396198	-0.623268	8.938957	0.00662	NM_001025421	Cugbp1
A_44_P400884	-0.62356	11.45829	0.004356	XM_345073	
A_44_P552986	-0.623801	9.054984	0.002394	NM_001007693	Ttc9c
A_44_P523016	-0.623912	12.37282	0.009372	NM_001024314	LOC499913

A_44_P1024729	-0.623962	9.026463	0.001142	XR_006336	RGD1311429_predicted
A_44_P303777	-0.624011	13.52164	0.00533	AW143533	
A_44_P271625	-0.624662	14.84276	0.002663	NM_053291	Pgk1
A_43_P17160	-0.62594	9.958981	0.00768	NM_001017383	Ppil2
A_44_P469040	-0.62652	8.165212	0.001126	XM_235547	RGD1560783_predicted
A_44_P917437	-0.626641	9.558865	0.002512		
A_44_P548203	-0.626707	12.49294	0.005956	BC087677	Rbm3
A_44_P293124	-0.628186	7.935044	0.009121	NM_001044243	Tasp1_predicted
A_44_P321359	-0.628274	10.01661	0.006258	XM_226455	Zfp612_predicted
A_44_P779609	-0.628795	8.320908	0.001183	NM_001037652	Zdhhc6
A_44_P171183	-0.629303	8.474922	0.00293	NM_001044267	MGC112775
A_43_P13046	-0.629635	9.752248	0.001519	NM_057147	Sec22l2
A_44_P318999	-0.629726	8.872416	0.008478		
A_43_P10386	-0.630223	12.97329	0.000518	CA511607	
A_44_P192548	-0.630339	9.351752	0.001843	XM_343535	Gtpbp2
A_44_P494723	-0.630505	11.4003	0.009436	XM_343716	
A_44_P262192	-0.631249	12.84926	0.009614	NM_182814	Cct4
A_44_P240248	-0.632188	8.634159	0.003151	AB012232	Nfib
A_44_P492268	-0.63219	10.8392	0.006459	XM_212982	
A_44_P403084	-0.632293	9.823149	0.001151	NM_001039044	Cdc42se1
A_44_P461807	-0.632695	8.095919	0.000504	XM_229225	Baz2b_predicted
A_44_P348825	-0.632978	9.691787	0.003731	NM_024150	Arf2
A_44_P398014	-0.633132	12.4155	0.0051	NM_053448	Hdac3
A_44_P527134	-0.63402	14.00775	0.007491	BC081744	Oxr1
A_44_P191962	-0.634064	9.541643	0.005569	NM_001007734	Echdc1
A_43_P19178	-0.634171	8.223944	0.001053	NM_031574	Rasa3
A_44_P400857	-0.634452	12.21639	0.006584	NM_001011926	Fts
A_43_P13362	-0.634717	10.72983	0.006961	NM_139094	Rbm16
A_44_P908874	-0.634786	8.955233	0.003401		
A_44_P112488	-0.635472	8.404066	0.0068	XM_343087	Galntl1
A_44_P454180	-0.635515	8.272513	0.003393	NM_182675	Rab40c
A_44_P288548	-0.63558	9.253557	0.004623	XR_008866	RGD1306534_predicted
A_44_P139291	-0.635986	9.326605	0.001776	XM_001058939	
A_44_P791631	-0.636117	8.807255	0.003104	XM_227139	RGD1565414_predicted
A_44_P473472	-0.636139	10.61491	0.003947	BC091233	RGD1307434_predicted
A_44_P760606	-0.637549	12.68986	0.002205	XR_007237	
A_42_P521902	-0.637726	8.609116	0.000822	NM_001079941	Gtf3c5_predicted
A_44_P345101	-0.637911	10.42344	0.005728	XM_217367	Bai3_predicted
A_44_P178179	-0.638409	11.32229	0.008534	XM_226561	Pgbd5_predicted
A_44_P508750	-0.638435	8.171009	0.004833	XM_001056358	
A_42_P707937	-0.639077	13.34077	0.000445	NM_080583	Ap2b1
A_44_P1007105	-0.63928	13.26468	0.00435	NM_138905	Ppap2b
A_43_P22124	-0.639677	13.337	0.004781	NM_213625	Dnajc7
A_44_P859113	-0.640213	8.953787	0.004534	NM_199463	Ripk5
A_44_P148695	-0.640551	9.640543	0.000911	XM_240915	RGD1307915_predicted
A_43_P12462	-0.641286	8.041492	0.002222	NM_030863	Msn
A_44_P383199	-0.641381	9.791592	0.002582	XM_214649	RGD1308706_predicted
A_44_P267595	-0.641725	10.04405	0.007378	XM_233839	Eml4_predicted
A_44_P323882	-0.641998	9.658085	0.003262	NM_001013073	Btbd9

A_44_P477889	-0.642086	13.29279	0.004444	NM_031783	Nefl
A_44_P523112	-0.643135	11.37171	0.003507	XM_232937	RGD1306148_predicted
A_44_P840751	-0.643651	8.0978	0.00193		
A_43_P11938	-0.643668	7.909169	0.0072	NM_017364	Zfp260
A_44_P340839	-0.643741	8.838773	0.004557	XM_001055446	
A_43_P17983	-0.644983	12.6799	0.006621	XM_342679	Cul1_predicted
A_44_P1054864	-0.645182	11.12162	0.002305	NM_001007662	Arcn1
A_44_P825644	-0.64519	9.057609	0.003705		
A_44_P369701	-0.646083	11.87503	0.003244	NM_001025660	Ei24
A_44_P152395	-0.646432	12.2429	0.008762	NM_001013222	Rnd1
A_44_P365882	-0.646949	10.84827	0.004004	NM_001014046	RGD1308697
A_44_P177678	-0.647221	8.401784	0.007057	CB547239	
A_44_P375613	-0.647271	10.40813	0.006682	NM_001013183	Narfl
A_44_P235336	-0.648526	9.743373	0.001962	NM_001083966	Rev3l
A_44_P438478	-0.648574	14.64021	0.009817	XR_006258	
A_44_P523924	-0.648626	9.63477	0.008841	NM_001007689	Ciapin1
A_44_P449969	-0.648891	8.622949	0.001171	NM_001009258	Chfr
A_43_P17172	-0.648993	7.549533	0.000382	XM_213385	Prpf8
A_44_P514539	-0.649428	12.17488	0.005961	NM_001008324	Eif4b
A_44_P442941	-0.649561	9.201323	0.00355	NM_032067	Ralbp1
A_43_P18744	-0.650106	11.15677	0.002633	NM_001012744	Enpp5
A_44_P443343	-0.650592	7.957523	0.001555	XM_216091	Pdk3
A_44_P137262	-0.650642	10.46178	0.003983	NM_148891	Nmt1
A_44_P366056	-0.6509	9.137319	0.001343	XM_233418	Mmachc_predicted
A_44_P395709	-0.651297	12.14363	0.005458	NM_134366	Rac1
A_44_P422063	-0.651673	8.392712	0.006469	XM_228173	Gopc_predicted
A_43_P17344	-0.652063	8.310589	0.001639	XM_232252	Bcl2l13_predicted
A_44_P555261	-0.652078	8.380619	0.008749	NM_031057	Aldh6a1
A_43_P12591	-0.65239	7.777536	0.000543	NM_031569	Oprl1
A_44_P441434	-0.6525	9.111109	0.003536	NM_131914	Cav2
A_44_P248111	-0.652911	9.320377	0.004791	NM_001047107	Igsf4c_predicted
A_44_P794608	-0.657157	9.089318	0.00904	AI112975	
A_44_P594969	-0.657411	7.785626	0.0006		
A_43_P18497	-0.657706	8.96092	0.001825	NM_001007609	Mfap3
A_43_P13344	-0.657852	8.783735	0.008338	NM_138976	Mfn1
A_44_P272294	-0.658024	7.434132	0.000969	XM_233937	Asxl2_predicted
A_44_P255341	-0.658029	10.7227	0.003693	XM_222661	Ipo9_predicted
A_44_P342314	-0.658159	13.2173	0.009311	NM_001014232	RGD1308373
A_43_P18271	-0.658212	11.17964	0.005555	XM_576264	RGD1564335_predicted
A_44_P466827	-0.658495	9.300238	0.007269	NM_001034835	RGD1309220
A_44_P233867	-0.659003	9.728503	0.003555	NM_172067	Spon1
A_44_P1007438	-0.659371	9.813387	0.004121	NM_001044238	Gdpd1_predicted
A_44_P215210	-0.659582	9.528286	0.00427	XM_342568	Ptprt_predicted
A_44_P166216	-0.659636	14.18189	0.004388	NM_001013059	Ndfip1
A_44_P886861	-0.65992	11.82148	0.002477	NM_001024245	Ccdc32
A_44_P170710	-0.660042	13.38092	0.007866	NM_013060	Id2
A_44_P344397	-0.661307	11.05304	0.007497	NM_001024233	Bles03
A_43_P17012	-0.661735	13.29255	0.003819	NM_001014227	RGD1307010
A_44_P667977	-0.663111	9.045672	0.004604	XR_007894	LOC367705

A_44_P902742	-0.663318	11.53225	0.001062	XM_343576	Als2cr13_predicted
A_42_P826202	-0.663566	8.962978	0.006901	NM_153732	Zfp597
A_44_P147702	-0.663636	8.978381	0.003573	XM_340853	RGD1311017_predicted
A_43_P12487	-0.664083	9.881514	0.001847	NM_031040	Grm7
A_44_P324042	-0.664103	9.089293	0.004246	NM_001034150	Srpr
A_44_P477927	-0.665178	9.91646	0.003267	NM_013225	Cntn6
A_44_P1026688	-0.665491	11.02828	0.006486	NM_001014263	Sypl
A_44_P552977	-0.665722	9.640369	0.003625	NM_001007693	Ttc9c
A_44_P175751	-0.66592	8.42582	0.006218	XR_009314	LOC690211
A_44_P352268	-0.665997	13.87135	0.000486	NM_031821	Plk2
A_44_P241439	-0.666017	12.2453	0.003634	NM_001024789	Nap1l2
A_44_P288691	-0.66628	12.61409	0.008512		
A_44_P475371	-0.666669	9.786368	0.004316	XM_342812	Ccnc
A_44_P334195	-0.666683	11.19342	0.003964	NM_001007624	Qars
A_44_P175461	-0.667045	8.767074	0.001832	XM_239761	RGD1565602_predicted
A_43_P12936	-0.66754	11.16029	0.009331	NM_053801	Sec14l2
A_44_P485453	-0.667632	10.32878	0.008859	XM_001063164	RGD1561943_predicted
A_44_P744519	-0.667916	13.73968	0.00043	NM_012992	Npm1
A_42_P654739	-0.668259	9.014156	0.003873	XM_221888	Rnf6_predicted
A_44_P208880	-0.668399	10.47121	0.006465	NM_001038595	Dnaja3
A_44_P476107	-0.668587	11.31869	0.003496	XM_217278	
A_44_P217590	-0.668667	9.059417	0.000726	NM_053794	Prkwnk1
A_44_P199661	-0.669436	8.687349	0.006699	XM_341464	RGD1563853_predicted
A_44_P632544	-0.669509	7.896681	0.005783	AA944304	
A_44_P231556	-0.669772	7.869283	0.00065	NM_001077589	Rgs16
A_44_P658594	-0.670139	9.191758	0.000253	CO575464	
A_44_P269596	-0.671296	13.26019	0.006176	XM_344976	Psmd13_predicted
A_44_P328302	-0.672591	8.777259	0.002665	XM_213992	Glmn
A_44_P473322	-0.672656	7.868224	0.006082	XM_224778	
A_44_P293944	-0.672671	13.8667	0.007733	NM_001005908	Ghitm
A_44_P775787	-0.673339	11.32534	0.00098	XM_575783	LOC500420
A_44_P281959	-0.673402	8.081822	0.000983	XM_218006	RGD1310304_predicted
A_44_P421391	-0.673725	8.967514	0.008583	NM_198763	Slc1a4
A_44_P414757	-0.674502	9.692538	0.00543	NM_001024770	Sec3l1
A_44_P521893	-0.674632	8.832269	0.00481	BG669130	
A_43_P11734	-0.674874	9.655395	0.008583	NM_013145	Gnai1
A_42_P814410	-0.67517	11.87156	0.007169	NM_212500	Gnl1
A_44_P175681	-0.675396	10.52681	0.00529	NM_001014011	Gramd3
A_44_P492635	-0.67561	14.04256	0.005462	NM_001008281	Psmd3
A_44_P243102	-0.677044	8.408617	0.008294	NM_017007	Gad1
A_44_P172941	-0.67789	9.432247	0.006219	NM_182821	Pxk
A_43_P20530	-0.678095	8.871127	0.006082	XM_231400	Steap2_predicted
A_44_P482200	-0.678514	12.40565	0.003411	XM_222785	Astn1
A_44_P112546	-0.679215	9.419917	0.009026	CO400633	RGD1309873_predicted
A_44_P182555	-0.681208	11.999	0.008571	NM_138509	Mapre1
A_44_P375322	-0.681511	10.1816	0.00845	XM_001074519	Trpc4ap
A_44_P338095	-0.681595	9.584036	0.00481	NM_001013112	Hibch
A_44_P525482	-0.68227	8.987538	0.005143	CB546704	
A_44_P300612	-0.683667	10.59645	0.004338	NM_022850	Dpp6

A_42_P712718	-0.683836	10.9304	0.005702	NM_001004280	RGD1303272
A_43_P19662	-0.683865	8.366867	0.004069	CB547048	
A_44_P121457	-0.683919	12.36108	0.004367	NM_001037778	Dctn5
A_44_P340490	-0.684095	8.01205	0.005384	XM_221232	RGD1310429_predicted
A_44_P104679	-0.684853	9.308212	0.005798	NM_053481	Pik3cb
A_43_P18706	-0.684899	8.990666	0.003995	NM_001012103	Trim32
A_44_P686038	-0.686229	9.953226	0.000504	NM_022198	Clcn4-2
A_44_P509099	-0.686288	11.11681	0.003834	NM_001008382	Cnot8
A_44_P248598	-0.68654	9.140249	4.10E-05	NM_001017459	LOC314859
A_44_P332041	-0.686639	11.95608	0.009963	NM_022385	Arl1
A_44_P525827	-0.686772	12.63864	0.003991	NM_199091	Cct3
A_44_P324066	-0.687064	8.571865	0.004003	XM_001072853	LOC689997
A_44_P149734	-0.687945	9.954353	0.005321	NM_001009657	Hat1
A_44_P466041	-0.688036	10.14526	0.001573	NM_001007682	Thtpa
A_43_P11763	-0.689954	8.225533	0.001545	Z49081	Add1
A_44_P466565	-0.690363	10.13551	0.005187	XM_001058018	RGD1565759_predicted
A_44_P959355	-0.690506	14.41659	0.00172	XR_008469	LOC361963
A_44_P636077	-0.691064	9.295773	0.002041	XM_225093	RGD1308377_predicted
A_44_P192568	-0.691346	9.9763	0.005102	NM_172335	Gm2a
A_44_P321605	-0.691528	14.38762	0.00993	XM_576504	LOC501089
A_44_P715184	-0.692584	8.486267	0.003501	XM_215883	Kif3b_predicted
A_44_P557979	-0.692677	8.67592	0.002177	NM_001012190	Ubap1
A_44_P285741	-0.692747	9.249087	0.009985	NM_031556	Cav
A_44_P128629	-0.693207	10.22845	0.006852	XM_344744	Gcdh_predicted
A_43_P18261	-0.693296	11.92018	0.002116	NM_001008345	Mesdc2
A_44_P356538	-0.693746	10.46584	0.002295	XM_341666	Zfp330_predicted
A_43_P16735	-0.694316	11.01645	0.007446	XM_340886	Nfe2l1_predicted
A_44_P426707	-0.694392	12.88563	0.009882	XM_001053199	RGD1560511_predicted
A_44_P370502	-0.695424	8.655955	0.009894	XM_342445	RGD1561141_predicted
A_44_P516017	-0.695966	10.87721	0.006661	NM_001031648	Efhd2
A_44_P234362	-0.696237	7.659072	0.000406	NM_001007745	Tmem43
A_44_P781559	-0.696443	12.18886	0.006077	XM_233944	Ncoa1_predicted
A_44_P543350	-0.696783	8.474691	0.00037	NM_017063	Kpnb1
A_44_P371823	-0.697464	9.537366	0.006001	XM_242940	
A_44_P471818	-0.697741	9.200884	0.003551	XM_001071384	LOC689616
A_44_P405679	-0.697777	9.039271	0.009317	XM_233609	Rap1ga1
A_43_P16946	-0.697811	9.672708	0.007204	XM_213649	Ifnar1_predicted
A_44_P509473	-0.698504	10.44605	0.005045	NM_139060	Csnk1d
A_44_P503673	-0.699146	9.366485	0.006058	XM_341100	Ulk1
A_44_P386748	-0.702079	13.34473	0.009233	NM_001007616	Wsb2
A_43_P20777	-0.702381	9.632501	0.009165	XM_341474	Nsun2_predicted
A_43_P15781	-0.702694	9.424702	0.009951	BC090029	Jak1
A_44_P345009	-0.704027	10.85685	0.006687	NM_001008526	Bag5
A_43_P16640	-0.704181	11.01546	0.006406	XM_343298	RGD1311547
A_44_P822082	-0.704323	13.25393	0.004761	XR_009104	
A_44_P648671	-0.706766	11.58223	0.006472	XM_001076507	RGD1559498_predicted
A_44_P928907	-0.706901	9.190431	0.000626	XM_574241	RGD1565049_predicted
A_44_P229079	-0.707063	11.94041	0.00343	XM_344434	Rb1
A_44_P510470	-0.707064	8.08123	0.008377	NM_053487	Pex11a

A_44_P278923	-0.707738	10.52196	6.05E-05	XM_224623	Oxnad1_predicted
A_44_P132822	-0.707764	8.916867	0.001035	NM_001005265	Crry
A_44_P110098	-0.708323	10.09779	0.001122	NM_001080783	RGD1311552_predicted
A_44_P260850	-0.708435	9.12765	0.0044	CO394432	
A_44_P206520	-0.709319	10.11567	0.004135	XM_217385	Lman2l_predicted
A_44_P429364	-0.710105	10.76198	0.000908	NM_001012113	Ptdss1
A_44_P135339	-0.710133	14.74068	0.002906	NM_175761	Hspca
A_44_P459851	-0.710349	9.658631	0.001275	XM_340974	Dscr3_predicted
A_44_P442873	-0.710445	13.18986	0.000449	NM_053739	Becn1
A_44_P535400	-0.710756	10.29758	0.00891	AW916612	
A_44_P463996	-0.710903	8.168438	0.000841	XM_344971	Inpp5a_predicted
A_44_P637889	-0.711137	9.731263	0.001241		
A_44_P271368	-0.711859	13.0028	0.00484	AW917142	
A_44_P494644	-0.712484	11.96627	0.006901	NM_001012075	Tspyl4
A_44_P365653	-0.713637	11.76364	0.005634	NM_001014161	LOC361596
A_44_P222779	-0.713943	8.50309	0.000525	XM_342937	RGD1305162_predicted
A_44_P543288	-0.714546	10.03262	0.003176	NM_031704	Stx5a
A_42_P724600	-0.71565	10.02345	0.003976	NM_001006976	Tars
A_44_P281540	-0.715686	8.615028	0.000588	XM_215428	LOC294560
A_44_P329139	-0.715693	8.394615	0.006545	XM_224588	Arhgef3_predicted
A_43_P17974	-0.716595	9.515081	0.005591	XM_341688	Cog4_predicted
A_44_P295151	-0.716847	9.093263	0.000871	XM_224561	Flnb_predicted
A_44_P403209	-0.717596	9.277081	0.005136	NM_001013073	Btbd9
A_43_P19299	-0.718378	7.772278	0.0002	XM_235571	RGD1309983_predicted
A_44_P323430	-0.718496	9.88312	0.002903	NM_138532	Nme7
A_44_P128447	-0.719167	11.31551	0.003131	NM_001007680	Abhd6
A_44_P229618	-0.71918	8.367113	0.000769	XM_228065	Tmem1_predicted
A_44_P187789	-0.719962	8.124942	0.007832	XM_001066818	
A_44_P474509	-0.720164	12.50656	0.001046	CO394478	
A_44_P389212	-0.720654	11.51459	0.007951	NM_001024975	RGD1307336
A_44_P887061	-0.720696	10.08852	0.007313	BC107438	
A_44_P260644	-0.72127	8.407463	0.001474	XM_576264	RGD1564335_predicted
A_44_P437641	-0.721334	9.637998	0.001922	NM_001007702	RGD1359108
A_44_P366177	-0.72167	9.880374	0.004393	DN933141	LOC681352
A_43_P17527	-0.722108	8.781197	0.00568	NM_001024261	Trnt1
A_44_P250983	-0.722291	10.81962	0.000881	XM_001057073	LOC680451
A_44_P124294	-0.722674	9.650509	0.004216	NM_001012003	Orc2l
A_44_P538531	-0.722867	10.08705	0.002979	NM_053742	Pitpnb
A_44_P424092	-0.722882	8.510474	0.006779	NM_001025669	RGD1307161
A_44_P189909	-0.723969	10.66671	0.002177	NM_001025020	RGD1310753
A_44_P607982	-0.724047	8.726039	0.009631		
A_44_P210461	-0.724218	14.11055	0.002096	NM_017025	Ldha
A_44_P227121	-0.725426	9.930826	0.000234	XM_341301	RGD1306755_predicted
A_44_P356322	-0.725778	10.89637	0.001673	XM_219482	
A_44_P126131	-0.726175	8.173707	0.001792	XM_341215	Tbc1d1_predicted
A_44_P827919	-0.726469	9.333749	0.00967		
A_42_P521609	-0.726957	9.608308	0.002166	XM_221635	Morc3_predicted
A_43_P12890	-0.727081	8.909091	0.003676	NM_053665	Akap1
A_44_P126310	-0.72742	12.22158	0.00529	NM_001005537	Ik

A_44_P438442	-0.728064	8.337862	0.006042	XM_001069431	
A_44_P197041	-0.728079	10.51144	0.001425	NM_001035221	Ypel5
A_44_P320894	-0.728496	10.37575	0.003358	NM_013090	Vamp1
A_44_P854406	-0.729	8.456598	0.007527		
A_44_P400112	-0.729177	12.19754	0.006955	CO384551	
A_44_P326124	-0.729686	15.26894	0.002926	XM_001056105	LOC679385
A_44_P154367	-0.730342	9.281083	0.004187	NM_001024744	Cdc16
A_44_P530424	-0.732079	12.73546	0.004615	BC085931	RGD1309459
A_44_P116846	-0.732713	10.5152	8.84E-05	XM_341384	LOC361098
A_44_P250412	-0.733285	9.320603	0.003192	NM_001007713	Tmbim1
A_43_P13047	-0.735128	8.592086	0.003122	NM_057148	Sep.02
A_44_P103160	-0.735662	10.16767	0.001835	XM_342909	Inpp5b
A_43_P11514	-0.736388	11.08872	0.002482	NM_012678	Tpm4
A_44_P119007	-0.736654	11.29753	0.002083	NM_172321	Slc6a15
A_44_P729866	-0.737709	10.44694	0.001265	CA511309	
A_44_P300506	-0.738032	13.37647	0.000788	NM_022399	Calr
A_44_P656856	-0.738458	11.02773	0.008158	CK596682	
A_44_P557962	-0.73918	14.79963	0.00234	XM_232602	
A_44_P520190	-0.739506	8.711355	0.007267	NM_134414	Exoc2
A_44_P443030	-0.739533	10.324	0.005014	XM_223227	Sept6_predicted
A_44_P525645	-0.739723	8.785316	0.001623	XM_220442	
A_44_P550581	-0.739757	8.369567	0.001509	XM_218411	Dmpk_predicted
A_44_P434216	-0.740314	10.10546	0.004603	NM_001012193	Rdh11
A_43_P12932	-0.740654	12.20032	0.002856	NM_053788	Stx1a
A_44_P233736	-0.74102	11.56517	0.004468	NM_001004090	Tspan5
A_44_P489120	-0.741475	10.20944	0.008299	NM_001007701	Tram1
A_44_P440633	-0.741539	10.4556	0.000987	NM_031639	Dlgh3
A_44_P415412	-0.742562	8.633771	0.005222	NM_001004252	Farslb
A_44_P445318	-0.742956	10.76738	0.002692	XM_215985	
A_43_P15432	-0.742965	8.174573	0.003091	NM_053910	Pscd1
A_44_P1015001	-0.743467	8.405712	0.008775	U50842	Nedd4a
A_44_P1014311	-0.743779	8.744107	0.003717	XM_343619	Atg16l1_predicted
A_44_P248484	-0.743898	13.35195	0.003782	XM_001069080	LOC367617
A_44_P312484	-0.743971	10.08945	0.002171	XM_213426	Mtmr4_predicted
A_44_P405793	-0.745803	9.317497	0.000835	XM_244089	Npas2_predicted
A_44_P473754	-0.746888	9.118205	0.001714	XM_221656	Son
A_43_P18072	-0.746961	8.805348	0.001287	NM_001013413	Als2
A_44_P714315	-0.747239	12.25655	0.002346	CO404950	
A_43_P18263	-0.750304	8.603148	0.005633	NM_001025421	Cugbp1
A_44_P760403	-0.750365	11.83512	0.000948		
A_44_P454177	-0.750546	9.564492	0.005748	NM_182675	Rab40c
A_43_P20387	-0.750553	8.592192	0.001522	XM_232614	Arfgef1_predicted
A_44_P853268	-0.751009	8.775023	0.000223	CO393484	
A_43_P16469	-0.751323	10.77797	0.006331	NM_001083313	Chmp1a
A_43_P10226	-0.751437	10.42195	0.009819	XM_236333	Lrrc49_predicted
A_44_P159569	-0.75145	9.190013	0.000607	NM_001039587	Wdr45l_predicted
A_44_P330332	-0.751549	8.728765	0.003646	BC129128	
A_44_P229800	-0.754324	9.060456	0.009386	NM_001011991	Ndrg1
A_43_P15423	-0.754442	9.635179	0.0005	NM_019239	Mgat3

A_44_P806641	-0.754958	11.49879	0.005591	XM_236024	
A_44_P187293	-0.755579	11.71979	0.0067	XM_344175	
A_44_P286719	-0.756576	9.510973	0.004347	XM_224863	Snx25
A_44_P321686	-0.756834	8.771057	0.006453	XM_001060299	
A_44_P270510	-0.757372	8.717218	0.005432	XM_220420	Aff4_predicted
A_44_P203842	-0.757977	8.823596	0.00582	BP485454	RGD1559751_predicted
A_44_P145416	-0.758544	8.271167	0.003876	NM_001007646	Fkbp9
A_44_P775952	-0.759294	12.15302	0.001784		
A_44_P482512	-0.759667	10.47499	0.000794	XM_343513	Aplp2
A_44_P548303	-0.760193	8.571865	0.004889	NM_001009688	Thumpd1
A_44_P375252	-0.760435	9.392698	0.00157	XM_342379	Ehmt1_predicted
A_44_P838313	-0.760683	13.05444	0.001		
A_43_P17880	-0.762673	8.703786	0.000302	NM_182953	Nek6
A_44_P545635	-0.762675	8.296813	0.00081	AY325140	RGD1560766_predicted
A_44_P541378	-0.762945	7.579984	0.000277	NM_001014242	Isoc1
A_44_P475159	-0.762947	13.19762	0.001813	NM_012673	Thy1
A_44_P261818	-0.76305	9.329422	0.004341	BC087666	
A_44_P806713	-0.763825	10.53177	0.0004		
A_44_P1020219	-0.764497	8.876013	0.003207	CO574509	Rundc1_predicted
A_44_P439573	-0.764536	13.61199	0.000165	DV728042	
A_43_P12453	-0.764651	9.151736	0.005921	NM_030839	Syt13
A_44_P253601	-0.765994	8.243451	0.001336	NM_001025009	Josd1
A_44_P203793	-0.766671	11.35587	0.002354	NM_001013912	Mllt11
A_44_P172936	-0.767375	9.619187	0.009972	NM_138613	Zfp179
A_44_P229670	-0.767526	12.75535	0.008309	XM_001075438	LOC686934
A_44_P451014	-0.768482	10.82196	0.006677	NM_017322	Mapk9
A_44_P203665	-0.768518	8.505706	0.004547	XM_223270	Ccng2_predicted
A_44_P550454	-0.768846	7.804654	0.000548		
A_44_P213320	-0.768863	14.2796	0.00708	XM_236873	LOC688956
A_44_P852634	-0.769854	8.744858	0.001617	XR_007953	LOC501135
A_44_P961330	-0.770326	10.5835	0.00473		
A_44_P351789	-0.770822	9.291229	0.005812	XM_341509	Cd83_predicted
A_44_P134405	-0.770887	9.108849	0.004683	NM_031798	Slc12a2
A_44_P545727	-0.771223	10.49515	0.001557	XM_341086	Fln29
A_44_P236656	-0.771271	9.238519	0.001101	XM_343817	Gla
A_44_P832837	-0.771519	9.96242	0.007956	AW143397	
A_44_P230410	-0.771562	12.57756	0.007994	NM_031600	Ptprn2
A_44_P363204	-0.77167	8.892924	0.006836	NM_053824	Csnk2a1
A_44_P274397	-0.771797	10.44541	0.004003	NM_001033700	Lass2
A_43_P17671	-0.772099	8.388576	0.001237	NM_001014186	LOC362304
A_44_P150089	-0.7723	8.616038	0.001656	NM_001013229	Eral1
A_44_P671470	-0.772302	9.103882	0.004433		
A_44_P459301	-0.77262	9.149703	0.007149	XM_219377	LOC309016
A_44_P377645	-0.773449	8.537705	0.000106	XM_342774	
A_44_P287250	-0.773756	11.2841	0.008399	NM_022521	Oat
A_44_P538538	-0.774107	11.86528	0.008686	NM_017357	Hpcal4
A_44_P250030	-0.775557	8.910662	0.009305	NM_013107	Bmp6
A_44_P142562	-0.776339	13.17596	0.003051	NM_001007643	Tmx2
A_44_P215689	-0.77949	12.56723	0.007158	XM_343744	LOC686934

A_44_P725617	-0.779699	12.15481	0.001587	BF548107	
A_44_P138363	-0.779752	11.52163	0.008934	XM_344057	LOC363849
A_44_P184279	-0.78011	11.55638	0.008311	NM_012504	Atp1a1
A_43_P17443	-0.780406	8.247825	9.77E-05	XM_230647	Rin2_predicted
A_44_P157118	-0.781188	9.479825	0.004446	BC089114	Foxred1_predicted
A_44_P159455	-0.782713	8.994728	0.002178	XM_001062352	Ibtk_predicted
A_44_P208711	-0.782766	13.39	0.009113	XM_001075438	
A_44_P290703	-0.782788	15.64819	0.002914	XM_226680	
A_44_P322500	-0.782907	8.670947	0.001796	CK473706	
A_44_P100740	-0.783096	12.38389	0.005403	NM_001024243	Nudt3
A_44_P386515	-0.783243	9.980967	0.006867	NM_001008771	Wdr77
A_44_P322960	-0.783753	12.49027	0.000134	NM_022532	Araf
A_44_P759589	-0.783856	12.06586	0.002218	CF110322	
A_44_P452668	-0.783988	8.976903	0.000605	XM_342731	Sumf1_predicted
A_44_P356468	-0.784919	10.14415	0.002477	XR_007829	RGD1561940_predicted
A_44_P283971	-0.785012	9.049607	0.000234	XM_232972	Zfp189_predicted
A_43_P19994	-0.785178	9.365389	0.000868	XM_233485	Rlf_predicted
A_43_P20118	-0.785793	10.4862	0.008255	BC099746	Suclg2
A_43_P21464	-0.78587	9.177944	0.006099	XM_233220	LOC313391
A_44_P382523	-0.786011	10.81879	0.009434	XM_236376	RGD1311958_predicted
A_44_P344425	-0.786114	9.781851	0.004082	XM_224538	Dock9
A_44_P1039444	-0.786386	8.248205	0.007048	NM_032613	Lasp1
A_44_P326090	-0.786522	11.3645	0.001936	XM_342312	Ahcyl1_predicted
A_44_P425380	-0.786802	12.00819	2.62E-05	NM_001011991	Ndrg1
A_44_P480240	-0.786957	9.61964	0.007872	NM_019182	Rnf4
A_44_P831218	-0.788899	12.51332	0.008455	CK596627	
A_43_P23188	-0.789235	8.984787	0.006612	XM_001053087	LOC678766
A_44_P422271	-0.789537	10.07162	0.000428	XM_217019	Cerk_predicted
A_44_P825629	-0.789986	8.743667	0.004918		
A_44_P966141	-0.790387	10.67786	0.004458	CO399145	
A_44_P501082	-0.790871	10.12628	0.008074	NM_134346	Rap1b
A_44_P367698	-0.791355	8.877036	0.005434	NM_031720	Dio2
A_43_P10511	-0.792255	11.50748	0.00296	XM_342289	
A_44_P340393	-0.792382	10.64778	5.48E-05	XR_009391	RGD1565753_predicted
A_44_P246128	-0.792787	10.07023	0.006945	XM_576696	LOC501281
A_44_P168102	-0.792962	11.70241	0.009798	NM_031729	Ppp5c
A_43_P17837	-0.792995	10.48533	0.004349	XM_215423	Sesn1_predicted
A_43_P18051	-0.793458	9.698695	0.001821	XM_001054856	LOC679203
A_44_P536676	-0.793756	10.54809	0.005142	NM_001013158	B3galt3
A_44_P506374	-0.794243	8.661933	0.001863	XM_001073596	Nyw1
A_44_P553939	-0.794537	10.15677	0.003392	BG667845	
A_44_P149954	-0.794596	9.399586	0.00696	XM_576917	RGD1562289_predicted
A_44_P324452	-0.794638	10.7937	0.004997	NM_001048184	Rragc_predicted
A_44_P1029770	-0.795192	11.5724	0.002588	NM_001025732	Nol5a
A_44_P485246	-0.795505	11.15076	0.000209	XR_006015	LOC296593
A_43_P21256	-0.79594	9.616947	0.007425	NM_001083966	Rev3l
A_44_P764308	-0.796777	12.43124	0.002224		
A_44_P1008284	-0.797081	11.05187	0.000467		
A_44_P500250	-0.797368	10.23157	0.007857	XM_343404	RGD1565253_predicted

A_44_P380157	-0.797475	13.15193	0.004485	XM_001074009	LOC690298
A_44_P539738	-0.798034	8.898049	0.007117	NM_022862	Unc13b
A_44_P268837	-0.800875	14.91321	0.002766	NM_001013923	LOC296318
A_44_P536282	-0.801147	14.54553	0.000242	NM_001013130	Spnb2
A_44_P506464	-0.802365	11.35145	0.003007	XR_005489	LOC316483
A_42_P505280	-0.80242	11.65719	0.002254	NM_001012063	Uble1a
A_43_P16672	-0.802949	11.21939	0.002994	NM_001024800	Txndc1
A_44_P331164	-0.804514	11.57423	0.004321	NM_001013974	RGD1308813
A_44_P218229	-0.805107	12.3132	0.007585	XR_006724	LOC363208
A_44_P806917	-0.805151	8.473763	0.001051		
A_44_P366005	-0.805172	11.03052	0.003555	XR_008061	RGD1560954_predicted
A_44_P387374	-0.805344	9.379108	0.003578	XM_221315	RGD1309443_predicted
A_44_P944991	-0.805734	11.55252	0.000681		
A_44_P335898	-0.805981	9.912098	0.003737	BC097982	
A_44_P871871	-0.807236	9.084835	0.000161		
A_44_P987960	-0.80876	11.55567	0.002189	CO398009	
A_44_P365155	-0.809646	13.55149	0.004859	NM_012583	Hprt
A_44_P410080	-0.810974	9.846895	0.007343	XM_001061719	RGD1563620_predicted
A_43_P13247	-0.810995	9.21568	0.004632	XM_001070141	Psd
A_43_P10441	-0.81137	10.65016	0.002046	XM_217149	RGD1310937_predicted
A_44_P558119	-0.811375	8.498026	0.004495	XM_236476	Elovl4_predicted
A_44_P612824	-0.811468	12.84505	0.006458	NM_183326	Gabra1
A_44_P524981	-0.811837	8.832762	0.005526	NM_024373	Galnt1
A_43_P13175	-0.812151	9.510029	0.003163	NM_133405	Cry2
A_44_P177793	-0.812473	13.2334	0.008377		
A_44_P165692	-0.813438	10.70566	0.00093	NM_001034003	RGD1308874
A_44_P344421	-0.814478	11.30413	0.001612	XM_224538	Dock9
A_44_P590197	-0.815765	12.77303	0.001655	AW915353	
A_44_P419565	-0.817066	8.767756	0.001182	XM_231128	RGD1310887_predicted
A_43_P10318	-0.817311	9.623956	0.009787	XM_001062954	Amfr_predicted
A_44_P375593	-0.817459	8.904718	0.004004	XM_001053401	RGD1305001_predicted
A_42_P658005	-0.817751	10.96039	0.000808	XM_573442	R3hdm1
A_44_P821396	-0.81801	11.14901	0.005183	XM_220013	RGD1564625_predicted
A_44_P297114	-0.818198	9.289671	0.000997	NM_001014226	RGD1308915
A_43_P10712	-0.818306	11.54703	0.000346	CO385963	
A_44_P135148	-0.818866	9.836715	0.005412	NM_130894	Mfn2
A_43_P20941	-0.818953	10.36852	0.003289	NM_001009685	Drg1
A_44_P114774	-0.819684	8.742619	0.003905	XM_343190	RGD1563028_predicted
A_44_P231724	-0.8197	11.55686	0.008186	XR_009038	LOC308950
A_44_P287286	-0.821021	9.32241	0.004277	XM_216910	
A_44_P589989	-0.821357	11.40158	0.006218	CA339579	
A_42_P544887	-0.823142	10.02887	0.004302	NM_031665	Stx6
A_44_P509872	-0.82419	9.224376	0.006955	XM_238280	Stard7_predicted
A_43_P23313	-0.824556	10.73031	0.006603	NM_001006960	MGC94604
A_44_P637687	-0.825702	12.07551	0.004244	XM_001079537	RGD1310754_predicted
A_44_P476598	-0.826362	13.11167	0.00335	XM_575808	
A_44_P471848	-0.82645	9.127431	0.002307	XM_225257	
A_44_P560788	-0.826837	14.22944	0.001736	CO566111	
A_43_P18579	-0.827324	11.47094	0.004409	XM_231162	Fbxw2_predicted

A_44_P507881	-0.827462	10.73166	0.005981	AI502676	
A_44_P550412	-0.827778	8.910014	0.001253	AF375874	Trpm7
A_44_P455174	-0.830207	10.55528	0.000725	NM_001014116	RGD1307381
A_44_P203682	-0.830529	10.79381	0.001988	XM_215113	Ptdss2_predicted
A_44_P671835	-0.830567	8.357442	0.003606		
A_44_P259574	-0.830641	14.92661	0.004778	NM_053290	Pgam1
A_44_P494510	-0.83076	8.47185	0.002851	XM_001068808	
A_44_P166777	-0.831154	8.870917	0.006837	XM_574528	LOC499235
A_44_P920120	-0.831683	10.40844	0.002672	XM_001066536	LOC686098
A_44_P606516	-0.83203	11.17502	0.000829		
A_44_P209817	-0.832416	9.940716	0.000341	BC078744	Cav
A_43_P21088	-0.83259	8.417264	0.002447	XM_229993	RGD1306214_predicted
A_44_P274491	-0.832886	11.54702	0.004151	XM_341645	Large_predicted
A_44_P141513	-0.833535	11.4231	0.003774	NM_001014175	LOC361990
A_44_P123908	-0.833932	9.166325	0.008822	XM_342338	RGD1311435
A_43_P18107	-0.834743	10.78105	0.002496	XR_008372	RGD1307034_predicted
A_44_P432432	-0.835275	9.693417	0.002327	NM_013151	Plat
A_43_P10854	-0.8356	9.558071	0.007977	NM_001011934	Hbs1l
A_44_P124225	-0.835726	11.15481	0.002671	NM_001025701	MGC116373
A_44_P852513	-0.835811	13.01714	0.00087	XR_005712	LOC366544
A_44_P883146	-0.835885	9.733681	0.001575	NM_001007682	Thtpa
A_43_P10831	-0.836254	9.547403	0.00254	NM_001013207	Rnpc2
A_44_P662685	-0.836691	8.319404	0.00219	NM_001025046	LOC500102
A_44_P540542	-0.836969	8.683687	0.004265	XM_001054154	Myef2
A_44_P720404	-0.837213	9.667063	0.004071	XM_231564	RGD1564895_predicted
A_44_P963185	-0.838192	12.09844	0.001448	AB014879	
A_44_P226881	-0.839542	12.48466	0.00757	NM_031079	Pde2a
A_44_P177807	-0.839994	8.88638	0.000103	NM_023090	Epas1
A_44_P511049	-0.840182	10.80784	0.001527	XM_224947	Prosc_predicted
A_44_P974681	-0.840279	10.35346	0.001809	XM_575362	RGD1562992_predicted
A_43_P17622	-0.84034	9.765676	0.000235	XM_345870	Lass5_predicted
A_44_P794598	-0.840375	12.69006	0.003716	NM_198132	Hnrpa3
A_44_P621980	-0.840874	11.64749	0.007824	M31178	
A_44_P163645	-0.841165	12.14695	0.000501	NM_001014135	Wdr1
A_43_P10843	-0.842047	9.483842	0.005797	XM_342312	Ahcyl1_predicted
A_44_P856158	-0.842088	8.219854	0.000446	XM_001058383	RGD1559576_predicted
A_44_P680369	-0.842217	8.724026	0.000689	BF545930	
A_44_P487562	-0.843181	7.793448	0.001154	NM_001039023	Net1
A_43_P15854	-0.843798	9.295432	0.002712	XM_227134	
A_44_P241862	-0.844188	9.094338	0.002739	NM_022615	Top1
A_44_P403415	-0.84445	8.996061	0.001818	XM_343301	Mpped1_predicted
A_44_P543548	-0.844926	9.422296	0.002666	NM_057119	Sfrs10
A_44_P1039760	-0.844948	9.985154	0.009284	NM_001013228	Tcte1l
A_44_P249164	-0.845962	13.33993	0.004086	NM_031028	Gabbr1
A_43_P15265	-0.847874	13.38868	0.000515	NM_017042	Ppp3cb
A_44_P242781	-0.848486	9.029121	0.001246	DY308836	
A_44_P501483	-0.850384	14.38167	0.000633	XR_006667	LOC310385
A_44_P475744	-0.850696	11.65921	0.005254	XM_215524	Tpd52_predicted
A_44_P151399	-0.851778	10.08971	0.003203	NM_198737	Arl6ip1

A_44_P112788	-0.852687	8.668467	0.000613	NM_021850	Bcl2l2
A_42_P693042	-0.85293	10.92209	0.001492	XM_214555	RGD1312005_predicted
A_44_P932876	-0.85312	10.2437	0.005947	NM_001014239	LOC364637
A_42_P713975	-0.853185	14.00655	0.007192	BC082799	LOC499779
A_43_P10213	-0.854676	11.58924	0.003286	XM_232515	Etnk1_predicted
A_44_P323496	-0.857383	9.456808	0.001818	NM_019311	Inpp5d
A_44_P322590	-0.858927	11.83874	0.004295	NM_057211	Klf9
A_44_P243701	-0.859383	12.2999	0.007041	XM_578871	
A_44_P753384	-0.860601	10.44877	0.001563		
A_44_P319042	-0.861633	10.52504	0.000626	XM_231798	Kbtbd2_predicted
A_44_P342586	-0.862131	9.340877	0.002361	NM_001014019	LOC308650
A_44_P425367	-0.86303	13.09488	0.005032	NM_001024332	LOC500282
A_44_P759588	-0.864571	12.31002	0.000475	CF110322	
A_44_P667768	-0.86515	10.03546	0.002647		
A_44_P520324	-0.865344	14.543	0.001973	XM_213911	
A_44_P342657	-0.867773	9.773441	0.0038	XM_233792	Vps13d_predicted
A_44_P534374	-0.867883	9.892686	0.00205	XM_227614	Extl2
A_44_P199420	-0.86915	10.28286	0.002966	XM_235924	RGD1309188_predicted
A_42_P522171	-0.869716	9.111699	0.004987	XM_001081512	RGD1307966_predicted
A_44_P930764	-0.870367	8.618998	0.001655	NM_001013125	Bfar
A_44_P145549	-0.870394	8.732414	0.001413	NM_019182	Rnf4
A_44_P230875	-0.870405	12.33114	0.004182	BF567478	
A_42_P545928	-0.871064	9.551304	0.007959	NM_153730	Klhl12
A_44_P233569	-0.871688	11.89088	0.000435	NM_031770	Gnb5
A_44_P198535	-0.874525	8.467903	0.000164	NM_057103	Akap12
A_44_P975286	-0.875152	9.924327	0.000359	CO403766	
A_44_P944912	-0.876799	10.35114	0.002205		
A_44_P362541	-0.87682	10.72609	0.00118	NM_001013235	Scoc
A_44_P283715	-0.876952	9.429911	0.006233	XM_223664	Ehbp1_predicted
A_44_P219796	-0.879236	13.31127	0.003461	XM_230038	Nckap1
A_44_P102628	-0.880199	12.04884	0.00607	NM_021688	Kcnk1
A_44_P548507	-0.882136	14.00466	0.006965	NM_032083	Chn1
A_44_P471440	-0.882876	9.075809	0.004845	NM_001024765	Nbr1
A_44_P476663	-0.88324	9.22413	0.001022	XM_344277	Asb3_predicted
A_44_P465329	-0.885403	13.18839	0.00018	NM_012798	Mal
A_44_P157167	-0.88618	11.39183	0.001068	NM_001008324	Eif4b
A_43_P17496	-0.887473	9.19712	0.008464	XM_231176	Rbm18_predicted
A_44_P137247	-0.891594	10.70451	0.002241	NM_001025142	Trim35
A_44_P438744	-0.892502	9.47676	0.001538	XM_233718	
A_44_P112050	-0.893598	8.93452	0.002972	XM_573428	RGD1560523_predicted
A_44_P895660	-0.893805	9.179857	0.009931	AW916954	
A_43_P17964	-0.893859	9.14457	0.001186	NM_001007662	Arcn1
A_44_P393929	-0.894308	9.800587	0.002936	NM_001039713	RGD1305121_predicted
A_44_P207721	-0.894337	8.940166	0.006695	XM_222946	Adss_predicted
A_44_P267724	-0.895152	8.893436	0.008978	XM_217022	Kif21a_predicted
A_44_P512255	-0.895159	12.15255	0.006149	NM_017214	Rgs4
A_44_P354208	-0.896039	10.41378	0.007776	XM_232315	Rasgef1a_predicted
A_44_P860400	-0.896072	10.18175	0.002993	CK839443	LOC680003
A_44_P344273	-0.897967	11.44876	0.002233	NM_001034129	Tloc1

A_44_P948202	-0.899553	11.85151	8.66E-05		
A_44_P463759	-0.899619	8.247009	6.28E-05	XM_235609	Prickle1
A_44_P667548	-0.89963	13.34538	0.004697		
A_44_P918128	-0.901601	9.504957	0.002334		
A_44_P233742	-0.902752	10.44578	0.000809	NM_001004090	Tspan5
A_44_P409694	-0.903718	11.87361	0.002481	XM_343604	Serpine2
A_44_P232642	-0.905716	10.50494	0.006499	NM_001008301	Usp14
A_44_P121401	-0.909039	9.597854	0.000594	XM_237998	
A_44_P356078	-0.912182	10.7963	0.000877	NM_017033	Pgm1
A_44_P157604	-0.912277	9.631781	0.005396	DY315019	RGD1565556_predicted
A_44_P745191	-0.913003	11.38851	0.002981	CF978988	Prkacb
A_43_P11416	-0.913741	11.3789	0.004315	XM_225625	Gpr158_predicted
A_44_P158973	-0.914102	9.52898	0.001232	NM_001013046	Rab35
A_43_P21122	-0.916898	11.25123	0.000669	XM_341882	Sytl2_predicted
A_44_P552865	-0.917112	12.17696	0.002942	XM_221896	LOC304280
A_44_P147509	-0.91732	11.89346	0.000271	NM_199412	Cbara1
A_43_P13702	-0.919716	10.92545	0.003644	NM_001025136	Clp1
A_44_P492153	-0.91998	8.651813	0.004016	XM_224271	Mtmr6_predicted
A_43_P10791	-0.921154	10.07704	0.001537	CB547648	
A_43_P12189	-0.922647	11.87634	0.007109	NM_022262	Tsnax
A_44_P526933	-0.922673	11.59775	0.001857	NM_053357	Ctnnb1
A_43_P10856	-0.922774	9.374146	0.006399	XM_001077761	
A_44_P415115	-0.923459	11.48848	0.003641	XM_345660	
A_44_P221077	-0.92647	11.11792	0.007184	NM_001017479	MGC108778
A_44_P505932	-0.927578	9.684475	0.000221	NM_021868	Cttn
A_44_P469394	-0.927674	10.95975	0.000659	NM_022217	Amph1
A_44_P409339	-0.931009	8.033903	0.005822	NM_053357	Ctnnb1
A_44_P621820	-0.933715	11.95108	0.002175	XR_005838	LOC501532
A_44_P349309	-0.936479	10.88574	0.007826	XM_223327	
A_44_P269752	-0.937257	12.53318	0.005391	XM_341581	Rnf125_predicted
A_44_P261326	-0.937683	9.315438	0.003697	XM_342782	Golt1b_predicted
A_44_P278639	-0.938965	9.436573	0.003089	NM_053693	Dmtf1
A_44_P210563	-0.939523	11.39399	0.003864	NM_012578	H1f0
A_44_P443976	-0.939799	8.653178	0.003824	NM_031697	St3gal3
A_44_P868060	-0.940038	9.085655	0.000327	XR_005433	
A_44_P143974	-0.940953	12.80713	0.001732	NM_001013235	Scoc
A_43_P21660	-0.941041	10.4107	0.000164	NM_001007147	Unc84a
A_43_P18787	-0.9414	9.262463	0.002095	XM_342552	Ncoa6
A_44_P115461	-0.942682	9.245119	0.007063	NM_017007	Gad1
A_44_P410062	-0.943971	12.47318	0.001655	XR_005977	LOC290503
A_43_P17406	-0.945546	9.715528	0.001908	NM_001005541	Vps16
A_44_P794770	-0.94696	10.27504	0.004338	NM_133560	Trak2
A_44_P973796	-0.947016	11.59966	0.004459	DY319575	
A_43_P10574	-0.947419	9.88833	0.000371	NM_001012012	Snx11
A_44_P164287	-0.947676	8.473386	0.000485	NM_001013925	Nub1
A_44_P427089	-0.948337	11.95941	0.000863	XM_341008	
A_44_P606344	-0.948971	10.54105	0.003253	XM_343550	RGD1310392_predicted
A_44_P356876	-0.950631	8.529601	8.67E-05	NM_001039002	RGD1304790
A_44_P113842	-0.951642	8.328451	0.001042	NM_022210	Max

A_44_P365516	-0.952073	14.32874	0.00153	BC059146	Tgfb1i4
A_43_P10725	-0.952393	10.57854	0.006304	NM_001012468	Rab18
A_43_P16458	-0.956172	13.35345	0.001088	NM_001011992	Atp6v1c1
A_44_P433334	-0.957339	9.778173	0.001503	NM_001012197	Tra1_predicted
A_44_P466489	-0.957393	10.38779	0.005784	NM_001007642	MGC93920
A_44_P413294	-0.958499	10.65194	0.004919	NM_022864	Cplx1
A_44_P288796	-0.958543	9.77281	0.001562	NM_001013125	Bfar
A_44_P609842	-0.959053	9.079842	0.006878		
A_44_P822078	-0.959623	10.3268	0.002286	XM_576832	LOC501422
A_44_P984248	-0.959633	8.922284	0.000801	XM_222785	Astn1
A_42_P832417	-0.959872	14.31019	0.001339	NM_012686	Vsnl1
A_44_P317520	-0.960089	11.88639	0.000868	BC079339	Atp6ap2
A_44_P286573	-0.96102	9.659496	0.000623	AA955833	
A_43_P11752	-0.964338	10.8451	0.0015	NM_013198	Maob
A_44_P342908	-0.965246	10.98916	0.000306	NM_001012076	Phyhipl
A_43_P17784	-0.966917	10.40971	0.000967	NM_001025722	Cdk10
A_44_P487021	-0.967089	11.75454	0.004192	NM_001002830	Rasl11b
A_44_P698487	-0.967251	11.0701	0.003175	XR_007600	LOC501341
A_44_P776000	-0.967895	12.74713	0.001375	XM_001061754	
A_44_P196757	-0.968211	10.26993	0.002441	XM_224256	Sacs_predicted
A_44_P764653	-0.968498	9.566406	0.000695	CO559017	
A_44_P166396	-0.969982	9.102654	0.000579	XM_216348	RGD1311622_predicted
A_44_P111368	-0.970396	10.00032	0.005783	NM_001037356	MGC125015
A_44_P191903	-0.971317	9.005184	0.000774	NM_053693	Dmtf1
A_44_P625415	-0.971557	14.28528	0.000403	XM_220333	Cyfip2_predicted
A_44_P237886	-0.97168	14.67145	0.009105	NM_031353	Vdac1
A_44_P318891	-0.973557	14.13979	0.004502	XR_007422	RGD1561111_predicted
A_44_P248541	-0.974703	10.35557	0.004283	NM_001014263	Sypl
A_44_P111959	-0.974894	8.187997	3.11E-06	BC087709	Slc15a4
A_44_P187827	-0.975297	10.99753	0.00531	XM_213437	Spop
A_44_P156439	-0.976221	9.753425	0.00196	XM_344861	Trim28
A_44_P193210	-0.979059	10.75687	0.005774	AY724483	LOC690163
A_44_P114023	-0.979827	9.12418	0.002735	NM_001047972	LOC501706
A_44_P501593	-0.981285	9.661811	0.001256	XM_230773	Acss2_predicted
A_44_P555525	-0.983285	11.03448	0.008015	NM_001007625	Epdr2
A_44_P347007	-0.985732	9.112875	0.005147	XM_001058095	
A_44_P290231	-0.991312	15.26656	0.005277	NM_013013	Psap
A_44_P201511	-0.991781	8.903813	0.000151	NM_001012087	Mar.07
A_44_P386840	-0.992813	13.26252	0.003197	NM_001013209	Dnajb6
A_44_P401170	-0.994067	10.95804	0.005615	NM_001025014	Tmem50b
A_44_P328311	-0.995783	13.29344	0.004944	XR_007180	LOC499261
A_44_P149161	-0.99613	10.26854	0.007722	NM_198738	Psat1
A_44_P238710	-0.997814	8.468174	0.000807	XM_218747	Tjp1_predicted
A_43_P10867	-1.00068	9.238465	0.001962	CB547678	
A_44_P239095	-1.001923	11.38682	0.000538	XM_223440	RGD1564407_predicted
A_43_P15490	-1.002544	11.39528	0.000103	NM_134377	Clstn2
A_44_P123898	-1.002823	10.07004	0.003245	XM_341452	
A_44_P214054	-1.004151	11.25551	0.008879	NM_031722	Tmed2
A_44_P162099	-1.004188	9.388554	0.002809	NM_053296	Glrb

A_43_P15623	-1.004336	10.74371	0.001195	NM_053414	Ddx1
A_44_P479520	-1.00546	10.10051	0.003832	XM_341907	Eif4g2_predicted
A_44_P905187	-1.005469	11.47995	0.002067	XM_233231	Dnajc6_predicted
A_44_P170309	-1.006565	8.625744	0.000217	XM_001064372	RGD1562407_predicted
A_44_P950583	-1.008831	9.836371	0.002687	CO396335	
A_44_P500906	-1.010881	9.936269	0.004815	NM_012788	Dlgh1
A_44_P912711	-1.012212	11.21529	0.004315	XM_224429	Pcdh9_predicted
A_44_P929345	-1.012916	10.90634	0.001278	XM_576703	LOC681644
A_44_P426919	-1.013338	12.66231	0.005288	XM_345601	
A_44_P328121	-1.016958	10.3368	0.006076	NM_057207	Sv2b
A_44_P978901	-1.017263	10.12303	0.00224	XM_218747	Tjp1_predicted
A_44_P296084	-1.020161	9.707363	0.000465	CB544459	
A_44_P159296	-1.020721	10.10136	0.007064	NM_001013929	Atp6v1h
A_44_P100991	-1.020722	10.01432	0.001454	XM_220404	RGD1310352
A_44_P519174	-1.021111	10.15332	0.000319	NM_001015027	Crebl2
A_44_P320955	-1.023289	9.428457	0.003607	NM_001012213	Sfxn1
A_44_P231994	-1.023305	9.89084	0.000809	XM_231626	Slc37a3_predicted
A_44_P183241	-1.023585	9.310267	0.005435	XM_221333	Bcl6_predicted
A_43_P16889	-1.025905	10.09083	0.002697	XM_220636	RGD1307929
A_44_P131031	-1.026384	15.044	0.002092	XR_006475	LOC499479
A_44_P428872	-1.027036	8.76117	0.007642	XM_341137	
A_44_P769703	-1.029805	8.731323	0.004312	NM_178096	Nrep
A_42_P843366	-1.030639	9.593706	0.001143	NM_022681	Adnp
A_44_P394401	-1.035642	9.837967	8.60E-05	XM_230531	Disp2_predicted
A_44_P269675	-1.039368	9.460818	0.006258	XM_001065667	LOC685894
A_44_P251692	-1.042788	12.91922	0.00797	NM_019257	Sfrs5
A_43_P15601	-1.043059	9.276099	0.00032	NM_134351	Mat2a
A_44_P118993	-1.045783	9.558989	0.00415	NM_134351	Mat2a
A_44_P108728	-1.052343	9.228743	0.005081	CA512164	
A_44_P356658	-1.052781	8.23732	0.000364	XM_219716	Pdzd4_predicted
A_44_P433568	-1.055139	10.05306	0.008337	XM_341882	Sytl2_predicted
A_44_P420005	-1.060142	10.64892	0.001587	NM_001047897	LOC360618
A_44_P110392	-1.062312	10.29743	0.009783		
A_44_P917564	-1.062494	13.42654	0.000786	NM_001044282	MGC94725
A_44_P790861	-1.062642	8.940687	0.001409		
A_44_P189326	-1.066924	12.61675	0.00045	NM_017319	Pdia3
A_44_P215393	-1.067533	11.4316	0.006142	NM_001025129	RGD1306327
A_44_P109887	-1.069138	12.16077	0.005229	XM_214007	Ccni_predicted
A_42_P509365	-1.069294	12.67278	0.005048	NM_031140	Vim
A_44_P374848	-1.069632	10.04428	0.000273	NM_024139	Chp
A_44_P354008	-1.072481	9.573272	0.001294	XM_224429	Pcdh9_predicted
A_44_P482267	-1.073559	9.237502	0.005217	NM_134382	Elovl5
A_43_P16591	-1.075943	9.78436	0.001714	AY724483	LOC690163
A_44_P461470	-1.077919	9.689354	0.000273	NM_053714	Ank
A_44_P699787	-1.077996	9.30303	0.000781	XM_221369	Senp5_predicted
A_44_P443138	-1.080637	14.64915	0.002534	XM_001054273	RGD1561628_predicted
A_44_P116904	-1.081159	12.77852	0.000733	XM_225314	Vmp_predicted
A_43_P11707	-1.081189	10.4956	0.000904	NM_013090	Vamp1
A_44_P484826	-1.081737	10.2743	0.002837	NM_017276	Gdi2

A_44_P856083	-1.082793	9.886582	0.001193		
A_44_P732939	-1.088751	9.200086	0.008222	XM_220269	
A_44_P828123	-1.088815	11.76102	0.002066	CO393245	
A_44_P687063	-1.094545	8.473641	0.009149		
A_43_P16511	-1.100214	9.35087	0.008189	NM_001044300	MGC112715
A_44_P651866	-1.100887	9.020218	0.000955	XR_009004	RGD1560288_predicted
A_44_P846589	-1.104643	10.63952	0.002532	NM_022229	Hspd1
A_44_P109862	-1.106694	10.03475	2.72E-05	NM_001047085	Ivns1abp_predicted
A_44_P141351	-1.119344	15.11579	0.0025	AY035551	
A_44_P855981	-1.121351	8.017247	0.002263		
A_44_P110158	-1.123021	9.375679	0.000752	XM_232077	Ctnna2_predicted
A_44_P433542	-1.126	9.254163	0.007077	NM_198132	Hnrpa3
A_44_P112641	-1.127383	10.04735	7.45E-05	XM_221214	RGD1304570_predicted
A_44_P108982	-1.131107	11.66852	0.004792	XM_341354	Sucla2_predicted
A_44_P283366	-1.131551	9.4448	0.000311	NM_181081	Myst2
A_44_P379420	-1.132617	14.026	9.91E-05	NM_017088	Gdi1
A_44_P903850	-1.134347	12.62073	0.009738	NM_001042621	Pip5k1a_predicted
A_44_P419757	-1.136875	11.96629	0.000772	XM_346210	
A_44_P187144	-1.144151	10.41503	0.001052	NM_080896	Hnrph1
A_44_P205301	-1.144216	11.84258	0.000678	NM_183402	Grinl1a
A_44_P281435	-1.148942	12.47708	0.000275	NM_001013923	LOC296318
A_44_P206284	-1.150263	8.977071	0.000717	XM_217496	RGD1566215_predicted
A_44_P458704	-1.158889	15.178	0.000227	NM_017073	Glul
A_44_P426808	-1.161242	10.50081	0.000721	NM_001013033	Tspyl
A_42_P828898	-1.169638	9.093417	0.000622	XM_219958	RGD1305793
A_43_P21284	-1.177773	9.659302	0.00667	NM_001012072	Ppp1r3c
A_44_P365598	-1.181633	10.34801	0.000439	NM_001012060	Rnf146
A_44_P554271	-1.182362	9.892054	0.003047	NM_001012235	Impact
A_43_P22174	-1.189049	9.629683	7.01E-05	NM_001014135	Wdr1
A_44_P549445	-1.189998	10.69697	0.001182	NM_130755	Cs
A_44_P149114	-1.190204	13.43588	0.007867	NM_057213	Atp6v1b2
A_43_P12430	-1.191741	9.018875	0.001834	NM_024374	Mtpn
A_43_P11881	-1.195272	14.19091	1.92E-05	NM_017253	Bcat1
A_44_P112597	-1.196669	10.69711	0.001391	XM_220423	Sept8_predicted
A_44_P196717	-1.210552	10.03627	0.007336	XM_344970	Stk32c_predicted
A_44_P496496	-1.216847	9.512697	0.001327	XM_342857	Sh3gl2
A_44_P852695	-1.230847	12.96167	0.002981	XR_007221	LOC367738
A_44_P594455	-1.232739	10.56054	0.002363	XM_341907	Eif4g2_predicted
A_43_P10894	-1.237647	10.40303	0.000796	NM_001025635	Nars
A_44_P409336	-1.242365	13.4477	0.002121	U56261	
A_44_P555389	-1.243916	11.1337	0.005838	NM_138839	Tmem49
A_43_P12816	-1.246149	9.601757	0.006035	U45479	Synj1
A_44_P550364	-1.247556	13.63787	0.00729	NM_080697	Dynll2
A_44_P538779	-1.250005	12.36209	0.003068	NM_001017385	Kdelr1
A_44_P625348	-1.263318	12.04507	0.004999		
A_43_P11613	-1.272921	11.1712	0.007455	NM_012903	Anp32a
A_44_P992854	-1.285168	11.73082	0.002797	NM_057132	Rhoa
A_44_P480605	-1.285189	13.4398	0.003088	XM_001066230	LOC291543
A_44_P243145	-1.291999	10.89005	0.001388	NM_021748	Nsf

A_44_P1040736	-1.294283	14.45449	0.000396	NM_199384	Laptm4a
A_44_P196625	-1.300095	11.39929	0.003723	XM_222253	
A_43_P18282	-1.304967	9.017291	0.000401	XM_341483	RGD1311345
A_44_P858481	-1.31791	9.906794	0.000748		
A_44_P442161	-1.319289	10.07827	0.003586	XM_342405	Ppp2r4_predicted
A_44_P964633	-1.334951	9.974101	0.001176	CO393123	
A_44_P104976	-1.341226	10.14277	0.000192	NM_080583	Ap2b1
A_44_P515029	-1.347381	11.9698	0.004702	XM_215469	Map1b
A_44_P293750	-1.357619	10.95115	0.001441	XM_001066493	Rnf14
A_44_P133044	-1.366986	11.71581	0.008077		
A_44_P338059	-1.370934	9.325555	0.000154	XM_343264	RGD1562665_predicted
A_44_P127156	-1.375902	10.39584	0.00155	M55017	
A_44_P480533	-1.376007	10.07984	0.000659		
A_44_P544023	-1.37978	15.59272	0.00558	NM_001011995	Tuba6
A_44_P902244	-1.382804	10.55446	0.002626	XM_001066493	Rnf14
A_43_P12839	-1.392875	12.20892	0.003602	NM_053531	Sybl1
A_44_P198396	-1.397774	12.74067	0.004725	NM_031841	Scd2
A_44_P958966	-1.414575	12.88172	1.04E-05	XR_005722	LOC360902
A_42_P618436	-1.425871	10.82035	0.00057	XM_235689	RGD1305138_predicted
A_44_P213415	-1.441176	12.31949	0.001576	NM_031353	Vdac1
A_44_P529743	-1.451843	10.57053	0.002358	XM_574587	RGD1562983_predicted
A_44_P107219	-1.469824	12.54211	0.003115	NM_207591	Gltscr2
A_44_P238314	-1.485104	9.641584	0.009513	NM_019621	Dlgh4
A_44_P918103	-1.509247	10.93965	0.002717		
A_44_P177434	-1.512322	12.02379	0.000964	NM_178105	Gpm6a
A_44_P488637	-1.526402	13.0635	0.004916	NM_019351	Timm17a
A_44_P180786	-1.563483	10.31511	0.00123	XM_217372	Plekhb2_predicted
A_44_P217648	-1.66614	11.36677	0.00039	NM_001037217	Mmd2_predicted
A_44_P488022	-1.667428	9.204686	1.12E-05	XM_576451	LOC501039
A_44_P238312	-1.676628	9.563557	0.005752	NM_019621	Dlgh4
A_44_P168842	-1.683993	10.19831	0.000402	XR_006947	LOC300429
A_44_P477322	-1.696866	13.70206	0.000621	NM_031831	Rtn4
A_43_P12141	-1.79599	11.19161	0.000277	NM_021869	Stx7
A_44_P448181	-1.799041	11.6104	0.000186	AY569014	Clstn1
A_43_P18735	-1.800031	10.71494	0.001217	NM_001030030	Cpsf3
A_43_P15367	-1.835326	11.87294	0.001591	NM_139189	Lmbrd1
A_44_P149148	-1.923989	10.84637	0.000345	NM_017309	Ppp3r1

Table S4

Median: 0.00991979436682584 Geo: 0.013098146982064234

Term	Count	%	PValue
GO:0032502~developmental process	29	24.37%	0.0019038
GO:0048513~organ development	17	14.29%	0.00264471
GO:0048468~cell development	16	13.45%	0.00365645
GO:0030154~cell differentiation	19	15.97%	0.00467809
GO:0048869~cellular developmental process	19	15.97%	0.00467809
GO:0048731~system development	19	15.97%	0.00999198
GO:0007275~multicellular organismal development	21	17.65%	0.0146788
GO:0048856~anatomical structure development	20	16.81%	0.02188862
GO:0007399~nervous system development	10	8.40%	0.03763158
GO:0009653~anatomical structure morphogenesis	11	9.24%	0.09039635
GO:0032501~multicellular organismal process	27	22.69%	0.44243405

Median: 0.007011150321556307 Geo: 0.01656665519371505

Term	Count	%	PValue
GO:0065007~biological regulation	36	30.25%	0.00358475
GO:0050789~regulation of biological process	32	26.89%	0.00624207
GO:0050794~regulation of cellular process	29	24.37%	0.00778023
GO:0019222~regulation of metabolic process	13	10.92%	0.43267287

Median: 0.05358450190602306 Geo: 0.03213539348414222

Term	Count	%	PValue
GO:0048468~cell development	16	13.45%	0.00365645
GO:0030154~cell differentiation	19	15.97%	0.00467809
GO:0048869~cellular developmental process	19	15.97%	0.00467809
GO:0016265~death	11	9.24%	0.00950467
GO:0008219~cell death	11	9.24%	0.00950467
GO:0008283~cell proliferation	11	9.24%	0.01140801
GO:0006915~apoptosis	10	8.40%	0.01943025
GO:0012501~programmed cell death	10	8.40%	0.02096655
GO:0048518~positive regulation of biological proce	12	10.08%	0.04580325
GO:0048523~negative regulation of cellular process	11	9.24%	0.05139072
GO:0048522~positive regulation of cellular process	11	9.24%	0.05577828
GO:0008284~positive regulation of cell proliferation	5	4.20%	0.05685355
GO:0043065~positive regulation of apoptosis	5	4.20%	0.06626229
GO:0043068~positive regulation of programmed ce	5	4.20%	0.06854287
GO:0048519~negative regulation of biological proce	11	9.24%	0.07541499
GO:0042127~regulation of cell proliferation	7	5.88%	0.08055805
GO:0042981~regulation of apoptosis	7	5.88%	0.09226092
GO:0043067~regulation of programmed cell death	7	5.88%	0.09745399
GO:0043066~negative regulation of apoptosis	4	3.36%	0.147503
GO:0043069~negative regulation of programmed c	4	3.36%	0.15030364

Median: 0.029321235494187657	Geo: 0.038754254726694026		
Term	Count	%	PValue
GO:0051179~localization	30	25.21%	7.56E-04
GO:0006810~transport	25	21.01%	0.00354746
GO:0051234~establishment of localization	25	21.01%	0.00574139
GO:0022892~substrate-specific transporter activity	14	11.76%	0.00815607
GO:0022857~transmembrane transporter activity	13	10.92%	0.00963755
GO:0006812~cation transport	9	7.56%	0.00979958
GO:0030001~metal ion transport	8	6.72%	0.01000551
GO:0022891~substrate-specific transmembrane tra	12	10.08%	0.01211268
GO:0015674~di-, tri-valent inorganic cation transpo	5	4.20%	0.01456401
GO:0005215~transporter activity	15	12.61%	0.02445628
GO:0022803~passive transmembrane transporter a	7	5.88%	0.02559288
GO:0015267~channel activity	7	5.88%	0.02559288
GO:0015075~ion transmembrane transporter activi	10	8.40%	0.02773796
GO:0006811~ion transport	10	8.40%	0.02932124
GO:0006816~calcium ion transport	4	3.36%	0.03470812
GO:0046873~metal ion transmembrane transporter	6	5.04%	0.04073964
GO:0008324~cation transmembrane transporter ac	8	6.72%	0.04694332
GO:0022838~substrate specific channel activity	6	5.04%	0.06781793
GO:0022890~inorganic cation transmembrane trans	4	3.36%	0.09697663
GO:0005216~ion channel activity	5	4.20%	0.16194302
GO:0015077~monovalent inorganic cation transme	3	2.52%	0.19009553
GO:0005261~cation channel activity	4	3.36%	0.20972592
GO:0015672~monovalent inorganic cation transpor	4	3.36%	0.27459217
GO:0005244~voltage-gated ion channel activity	3	2.52%	0.27903633
GO:0022832~voltage-gated channel activity	3	2.52%	0.27903633
GO:0022836~gated channel activity	3	2.52%	0.50244347
transport	4	3.36%	0.92879381

Median: 0.05103870428246421	Geo: 0.04066100997140925		
Term	Count	%	PValue
gpi-anchor	4	3.36%	0.00498683
GO:0048503~GPI anchor binding	4	3.36%	0.00526782
GO:0035091~phosphoinositide binding	4	3.36%	0.03295023
GO:0008289~lipid binding	6	5.04%	0.0510387
lipoprotein	6	5.04%	0.0603392
GO:0005543~phospholipid binding	4	3.36%	0.08847203
glycoprotein	10	8.40%	0.77916217

Median: 0.06375143382925563	Geo: 0.055121287609943094		
Term	Count	%	PValue
GO:0000902~cell morphogenesis	9	7.56%	0.00654198
GO:0032989~cellular structure morphogenesis	9	7.56%	0.00654198
GO:0051674~localization of cell	8	6.72%	0.00843971

GO:0006928~cell motility	8	6.72%	0.00843971
GO:0045664~regulation of neuron differentiation	3	2.52%	0.02142502
GO:0030182~neuron differentiation	6	5.04%	0.03435246
GO:0007399~nervous system development	10	8.40%	0.03763158
GO:0009966~regulation of signal transduction	7	5.88%	0.0550051
GO:0048699~generation of neurons	6	5.04%	0.0551754
GO:0009888~tissue development	6	5.04%	0.06087848
GO:0030030~cell projection organization and bioge	5	4.20%	0.06255205
GO:0032990~cell part morphogenesis	5	4.20%	0.06255205
GO:0048858~cell projection morphogenesis	5	4.20%	0.06255205
GO:0009611~response to wounding	7	5.88%	0.06495082
GO:0022008~neurogenesis	6	5.04%	0.07722863
GO:0048667~neuron morphogenesis during differei	4	3.36%	0.08301481
GO:0048812~neurite morphogenesis	4	3.36%	0.08301481
GO:0050793~regulation of developmental process	5	4.20%	0.0890998
GO:0009653~anatomical structure morphogenesis	11	9.24%	0.09039635
GO:0016477~cell migration	5	4.20%	0.09174819
GO:0040007~growth	5	4.20%	0.09533805
GO:0000904~cellular morphogenesis during differe	4	3.36%	0.10288219
GO:0031175~neurite development	4	3.36%	0.11410047
GO:0048666~neuron development	4	3.36%	0.14890113
GO:0045595~regulation of cell differentiation	3	2.52%	0.29754846
GO:0007243~protein kinase cascade	4	3.36%	0.38887732

Median: 0.06568497554225985 Geo: 0.07576685350125058

Term	Count	%	PValue
GO:0006665~sphingolipid metabolic process	4	3.36%	0.00289589
GO:0046519~sphingoid metabolic process	3	2.52%	0.01366877
GO:0006643~membrane lipid metabolic process	4	3.36%	0.06305875
GO:0005783~endoplasmic reticulum	8	6.72%	0.06568498
GO:0044255~cellular lipid metabolic process	5	4.20%	0.38170379
GO:0008610~lipid biosynthetic process	3	2.52%	0.43645072
GO:0006629~lipid metabolic process	5	4.20%	0.52477172

Median: 0.12698373523485457 Geo: 0.1085747713659542

Term	Count	%	PValue
GO:0030659~cytoplasmic vesicle membrane	4	3.36%	0.00995806
GO:0044433~cytoplasmic vesicle part	4	3.36%	0.01786045
GO:0012506~vesicle membrane	4	3.36%	0.02250049
GO:0006886~intracellular protein transport	7	5.88%	0.03008529
GO:0030662~coated vesicle membrane	3	2.52%	0.03867681
GO:0046907~intracellular transport	9	7.56%	0.04142686
GO:0031982~vesicle	6	5.04%	0.05214339
calcium	6	5.04%	0.0881864
GO:0016023~cytoplasmic membrane-bound vesicle	5	4.20%	0.10152223

GO:0031988~membrane-bound vesicle	5	4.20%	0.10963595
GO:0015031~protein transport	7	5.88%	0.11611726
GO:0031410~cytoplasmic vesicle	5	4.20%	0.12233789
GO:0051649~establishment of cellular localization	9	7.56%	0.12639205
GO:0031090~organelle membrane	8	6.72%	0.12757542
GO:0051641~cellular localization	9	7.56%	0.13266272
GO:0045184~establishment of protein localization	7	5.88%	0.1517592
GO:0022607~cellular component assembly	6	5.04%	0.16807623
GO:0008104~protein localization	7	5.88%	0.18813107
GO:0006461~protein complex assembly	4	3.36%	0.20488479
GO:0033036~macromolecule localization	7	5.88%	0.21509493
GO:0030135~coated vesicle	3	2.52%	0.24447744
GO:0016192~vesicle-mediated transport	5	4.20%	0.27460159
GO:0065003~macromolecular complex assembly	5	4.20%	0.27726166
alternative splicing	7	5.88%	0.28705867
GO:0012505~endomembrane system	4	3.36%	0.49566323
splice variant	6	5.04%	0.50529718

Median: 0.09566593468640966 Geo: 0.12641338012275546

Term	Count	%	PValue
GO:0048514~blood vessel morphogenesis	4	3.36%	0.06705093
GO:0009653~anatomical structure morphogenesis	11	9.24%	0.09039635
GO:0001568~blood vessel development	4	3.36%	0.09097701
GO:0001944~vasculature development	4	3.36%	0.09566593
GO:0001525~angiogenesis	3	2.52%	0.17276244
GO:0009887~organ morphogenesis	5	4.20%	0.23150988
GO:0048646~anatomical structure formation	3	2.52%	0.244505

Median: 0.4181424454299303 Geo: 0.12708631750829097

Term	Count	%	PValue
GO:0051258~protein polymerization	5	4.20%	3.37E-04
GO:0046785~microtubule polymerization	3	2.52%	0.00122634
propeptide:Removed in mature form	5	4.20%	0.00483429
GO:0007010~cytoskeleton organization and biogen	9	7.56%	0.00554424
GO:0051128~regulation of cellular component orga	4	3.36%	0.00739916
GO:0031109~microtubule polymerization or depoly	3	2.52%	0.00960909
GO:0030036~actin cytoskeleton organization and b	5	4.20%	0.0231797
Methylation	4	3.36%	0.02721095
GO:0030029~actin filament-based process	5	4.20%	0.02752056
GO:0006996~organelle organization and biogenesis	11	9.24%	0.04869729
GO:0000226~microtubule cytoskeleton organizatio	3	2.52%	0.07868213
GO:0050793~regulation of developmental process	5	4.20%	0.0890998
GO:0007017~microtubule-based process	4	3.36%	0.11923121
rno04810:Regulation of actin cytoskeleton	4	3.36%	0.30419871

cytoplasm	9	7.56%	0.40296436
GO:0017111~nucleoside-triphosphatase activity	5	4.20%	0.41814245
GO:0016462~pyrophosphatase activity	5	4.20%	0.44739985
GO:0016818~hydrolase activity, acting on acid anh	5	4.20%	0.45292712
GO:0016817~hydrolase activity, acting on acid anh	5	4.20%	0.4598126
GO:0042623~ATPase activity, coupled	3	2.52%	0.51221187
nucleotide-binding	6	5.04%	0.55462111
GO:0016787~hydrolase activity	13	10.92%	0.58971636
GO:0016887~ATPase activity	3	2.52%	0.6078906
atp-binding	4	3.36%	0.72056333
GO:0032553~ribonucleotide binding	6	5.04%	0.96851476
GO:0032555~purine ribonucleotide binding	6	5.04%	0.96851476
GO:0017076~purine nucleotide binding	6	5.04%	0.97692857
GO:0000166~nucleotide binding	7	5.88%	0.97699087
GO:0005524~ATP binding	4	3.36%	0.9859679
GO:0032559~adenyl ribonucleotide binding	4	3.36%	0.98743697
GO:0030554~adenyl nucleotide binding	4	3.36%	0.99120971

Median: 0.22688672366390947 Geo: 0.17317491533194845

Term	Count	%	PValue
GO:0040007~growth	5	4.20%	0.09533805
GO:0016049~cell growth	3	2.52%	0.22688672
GO:0008361~regulation of cell size	3	2.52%	0.24009306

Median: 0.2165902008057497 Geo: 0.19738681531543417

Term	Count	%	PValue
GO:0009611~response to wounding	7	5.88%	0.06495082
GO:0006950~response to stress	10	8.40%	0.16020793
GO:0006954~inflammatory response	4	3.36%	0.27297247
GO:0006952~defense response	4	3.36%	0.53442293

Median: 0.21605817549213288 Geo: 0.19865088907109454

Term	Count	%	PValue
GO:0009888~tissue development	6	5.04%	0.06087848
GO:0001503~ossification	3	2.52%	0.1920233
GO:0031214~biomineral formation	3	2.52%	0.1920233
GO:0046849~bone remodeling	3	2.52%	0.24009306
GO:0048771~tissue remodeling	3	2.52%	0.26881863
GO:0001501~skeletal development	3	2.52%	0.42416269

Median: 0.27889043603661545 Geo: 0.2011624834289807

Term	Count	%	PValue
GO:0044444~cytoplasmic part	27	22.69%	0.03720137
GO:0005737~cytoplasm	33	27.73%	0.05258976
GO:0005622~intracellular	51	42.86%	0.13093863

GO:0043229~intracellular organelle	41	34.45%	0.1348883
GO:0043226~organelle	41	34.45%	0.13808623
GO:0044424~intracellular part	45	37.82%	0.27889044
GO:0043231~intracellular membrane-bound organe	33	27.73%	0.30426919
GO:0043227~membrane-bound organelle	33	27.73%	0.30690129
GO:0005623~cell	75	63.03%	0.37068418
GO:0044464~cell part	74	62.18%	0.54899273
GO:0009987~cellular process	64	53.78%	0.863184

Median: 0.15196001235368436 Geo: 0.20457578861941178

Term	Count	%	PValue
GO:0005783~endoplasmic reticulum	8	6.72%	0.06568498
endoplasmic reticulum	5	4.20%	0.15196001
transmembrane region	10	8.40%	0.85776306

Median: 0.13366383848907484 Geo: 0.22905797793122154

Term	Count	%	PValue
GO:0006935~chemotaxis	3	2.52%	0.13340296
GO:0042330~taxis	3	2.52%	0.13340296
GO:0007610~behavior	5	4.20%	0.13366384
GO:0007626~locomotory behavior	3	2.52%	0.26660663
GO:0042221~response to chemical stimulus	6	5.04%	0.99428592

Median: 0.21635803992904926 Geo: 0.2376488035116084

Term	Count	%	PValue
GO:0005096~GTPase activator activity	3	2.52%	0.18339936
GO:0030234~enzyme regulator activity	7	5.88%	0.20844452
GO:0030695~GTPase regulator activity	4	3.36%	0.22427156
GO:0008047~enzyme activator activity	3	2.52%	0.37203176

Median: 0.22736533800174696 Geo: 0.2824205975609152

Term	Count	%	PValue
GO:0048545~response to steroid hormone stimulus	3	2.52%	0.12540959
GO:0009719~response to endogenous stimulus	5	4.20%	0.20139135
GO:0009725~response to hormone stimulus	3	2.52%	0.25333932
GO:0042221~response to chemical stimulus	6	5.04%	0.99428592

Median: 0.2986929605585694 Geo: 0.2956774482192189

Term	Count	%	PValue
GO:0050790~regulation of catalytic activity	5	4.20%	0.23023173
GO:0065009~regulation of a molecular function	5	4.20%	0.29869296
GO:0051338~regulation of transferase activity	3	2.52%	0.37589317

Median: 0.48319041257918804 Geo: 0.2994837815815022

Term	Count	%	PValue

GO:0007050~cell cycle arrest	3	2.52%	0.03831729
GO:0045786~negative regulation of progression thr	3	2.52%	0.13139359
GO:0000074~regulation of progression through cell	3	2.52%	0.48028099
GO:0051726~regulation of cell cycle	3	2.52%	0.48609983
GO:0022402~cell cycle process	3	2.52%	0.74850836
GO:0007049~cell cycle	3	2.52%	0.82007454

Median: 0.36012413372890034 Geo: 0.34114833908466563

Term	Count	%	PValue
GO:0005615~extracellular space	16	13.45%	0.10473089
GO:0044421~extracellular region part	16	13.45%	0.14825301
GO:0005576~extracellular region	16	13.45%	0.28147749
signal	11	9.24%	0.36012413
disulfide bond	10	8.40%	0.49379321
signal peptide	9	7.56%	0.82725323
Secreted	4	3.36%	0.83645974

Median: 0.41578343518711314 Geo: 0.3415495270682285

Term	Count	%	PValue
GO:0031090~organelle membrane	8	6.72%	0.12757542
GO:0031966~mitochondrial membrane	4	3.36%	0.21886529
GO:0005740~mitochondrial envelope	4	3.36%	0.26119565
GO:0044429~mitochondrial part	4	3.36%	0.38474803
GO:0005743~mitochondrial inner membrane	3	2.52%	0.41578344
GO:0019866~organelle inner membrane	3	2.52%	0.44419225
GO:0031975~envelope	4	3.36%	0.47119996
GO:0031967~organelle envelope	4	3.36%	0.47119996
GO:0005739~mitochondrion	6	5.04%	0.54972855

Median: 0.6692685796256796 Geo: 0.34206884401432364

Term	Count	%	PValue
GO:0005886~plasma membrane	21	17.65%	0.00824633
membrane	23	19.33%	0.04504188
GO:0044459~plasma membrane part	15	12.61%	0.07477456
GO:0005887~integral to plasma membrane	8	6.72%	0.35720236
GO:0016020~membrane	42	35.29%	0.37195217
GO:0031226~intrinsic to plasma membrane	8	6.72%	0.3720425
transmembrane	13	10.92%	0.66010746
GO:0044425~membrane part	32	26.89%	0.6784297
transmembrane protein	3	2.52%	0.77150498
topological domain:Cytoplasmic	8	6.72%	0.85461623
transmembrane region	10	8.40%	0.85776306
GO:0016021~integral to membrane	24	20.17%	0.94918057
GO:0031224~intrinsic to membrane	24	20.17%	0.95202413
topological domain:Extracellular	5	4.20%	0.95580871

Median: 0.6697869754096847 Geo: 0.3551016719532347

Term	Count	%	PValue
GO:0007242~intracellular signaling cascade	14	11.76%	0.0380455
GO:0007154~cell communication	27	22.69%	0.54613563
GO:0007165~signal transduction	22	18.49%	0.79343832
GO:0007166~cell surface receptor linked signal tra	11	9.24%	0.9644815

Median: 0.4580394280214789 Geo: 0.3817041963201787

Term	Count	%	PValue
GO:0003714~transcription corepressor activity	3	2.52%	0.10708232
GO:0016564~transcription repressor activity	3	2.52%	0.33159958
GO:0003712~transcription cofactor activity	3	2.52%	0.45803943
GO:0008134~transcription factor binding	3	2.52%	0.65248975
GO:0030528~transcription regulator activity	7	5.88%	0.76353048

Median: 0.9948670647412782 Geo: 0.38869644747229287

Term	Count	%	PValue
GO:0007267~cell-cell signaling	10	8.40%	0.02010251
GO:0007268~synaptic transmission	6	5.04%	0.08269739
GO:0019226~transmission of nerve impulse	6	5.04%	0.1264984
GO:0003008~system process	9	7.56%	0.96937986
GO:0050877~neurological system process	6	5.04%	0.99486706
GO:0007186~G-protein coupled receptor protein si	5	4.20%	0.99888011
GO:0001584~rhodopsin-like receptor activity	3	2.52%	0.99985866
GO:0004930~G-protein coupled receptor activity	3	2.52%	0.99994731
GO:0004888~transmembrane receptor activity	4	3.36%	0.99995356

Median: 0.7490033588699851 Geo: 0.4160549356873782

Term	Count	%	PValue
GO:0031090~organelle membrane	8	6.72%	0.12757542
GO:0044446~intracellular organelle part	13	10.92%	0.74900336
GO:0044422~organelle part	13	10.92%	0.75370476

Median: 0.4748806082394614 Geo: 0.45696154314135434

Term	Count	%	PValue
GO:0005509~calcium ion binding	7	5.88%	0.30641968
GO:0043167~ion binding	20	16.81%	0.35301025
GO:0046872~metal ion binding	19	15.97%	0.41200894
GO:0043169~cation binding	17	14.29%	0.53775228
GO:0008270~zinc ion binding	9	7.56%	0.58962396
GO:0046914~transition metal ion binding	11	9.24%	0.64433232

Median: 0.6108548056123693 Geo: 0.46366787538666515

Term	Count	%	PValue

GO:0009055~electron carrier activity	4	3.36%	0.16010272
rno00190:Oxidative phosphorylation	3	2.52%	0.29465413
GO:0005739~mitochondrion	6	5.04%	0.54972855
GO:0006091~generation of precursor metabolites a	4	3.36%	0.67198106
GO:0006118~electron transport	3	2.52%	0.72145424
GO:0016491~oxidoreductase activity	5	4.20%	0.79034494

Median: 0.5781882914454173 Geo: 0.4860358594698407

Term	Count	%	PValue
GO:0005856~cytoskeleton	8	6.72%	0.18425912
GO:0043232~intracellular non-membrane-bound or	10	8.40%	0.57818829
GO:0043228~non-membrane-bound organelle	10	8.40%	0.57818829
GO:0044430~cytoskeletal part	4	3.36%	0.64268789
GO:0005198~structural molecule activity	5	4.20%	0.68513139

Median: 0.9423352055185812 Geo: 0.5612926576195539

Term	Count	%	PValue
GO:0006119~oxidative phosphorylation	3	2.52%	0.09294528
GO:0006091~generation of precursor metabolites a	4	3.36%	0.67198106
GO:0016310~phosphorylation	3	2.52%	0.94233521
GO:0006793~phosphorus metabolic process	3	2.52%	0.97292307
GO:0006796~phosphate metabolic process	3	2.52%	0.97292307

Median: 0.9706858737440671 Geo: 0.6755664698446382

Term	Count	%	PValue
rno04012:ErbB signaling pathway	3	2.52%	0.19756883
kinase	3	2.52%	0.74437278
GO:0016301~kinase activity	3	2.52%	0.97068587
GO:0016772~transferase activity, transferring phos	3	2.52%	0.98728428
GO:0016740~transferase activity	4	3.36%	0.99841383

Median: 0.7330494427919996 Geo: 0.689925413309536

Term	Count	%	PValue
Ionic channel	3	2.52%	0.48234063
ion transport	3	2.52%	0.73304944
transport	4	3.36%	0.92879381

Median: 0.7762607275814594 Geo: 0.7381337367173091

Term	Count	%	PValue
GO:0019538~protein metabolic process	20	16.81%	0.51096511
GO:0043170~macromolecule metabolic process	32	26.89%	0.65084492
GO:0044260~cellular macromolecule metabolic pro	17	14.29%	0.72895769
GO:0044238~primary metabolic process	36	30.25%	0.76037186
GO:0044267~cellular protein metabolic process	16	13.45%	0.79214959
GO:0044237~cellular metabolic process	35	29.41%	0.81029799

GO:0008152~metabolic process	39	32.77%	0.85052368
GO:0043283~biopolymer metabolic process	19	15.97%	0.87567903

Median: 0.796723032466517 Geo: 0.7558166759021745

Term	Count	%	PValue
GO:0019222~regulation of metabolic process	13	10.92%	0.43267287
GO:0010468~regulation of gene expression	11	9.24%	0.53827834
GO:0031323~regulation of cellular metabolic proce:	11	9.24%	0.62702663
GO:0045449~regulation of transcription	9	7.56%	0.71296839
GO:0006355~regulation of transcription, DNA-depe	8	6.72%	0.74680584
GO:0019219~regulation of nucleobase, nucleoside,	9	7.56%	0.74927528
GO:0030528~transcription regulator activity	7	5.88%	0.76353048
GO:0006350~transcription	9	7.56%	0.77827361
GO:0006351~transcription, DNA-dependent	8	6.72%	0.79672303
GO:0032774~RNA biosynthetic process	8	6.72%	0.79846712
GO:0010467~gene expression	13	10.92%	0.81665599
GO:0006139~nucleobase, nucleoside, nucleotide ar	14	11.76%	0.82572682
GO:0016070~RNA metabolic process	9	7.56%	0.8732745
GO:0043283~biopolymer metabolic process	19	15.97%	0.87567903
GO:0003700~transcription factor activity	4	3.36%	0.89256127
GO:0003677~DNA binding	7	5.88%	0.91650031
GO:0003676~nucleic acid binding	12	10.08%	0.92266281

Median: 0.7346365499807694 Geo: 0.7567949594906387

Term	Count	%	PValue
GO:0006412~translation	4	3.36%	0.6856941
GO:0009058~biosynthetic process	8	6.72%	0.73223591
GO:0009059~macromolecule biosynthetic process	5	4.20%	0.73703719
GO:0044249~cellular biosynthetic process	5	4.20%	0.88642519

Median: 0.8973573679866338 Geo: 0.8617550111058944

Term	Count	%	PValue
zinc-finger	3	2.52%	0.77262736
metal-binding	5	4.20%	0.89735737
zinc	3	2.52%	0.92303026

Median: 0.9736314715800498 Geo: 0.9595490109139895

Term	Count	%	PValue
GO:0043687~post-translational protein modificatior	5	4.20%	0.92717983
GO:0006464~protein modification process	5	4.20%	0.97363147
GO:0043412~biopolymer modification	5	4.20%	0.97868494

Median: 0.9468890540524483 Geo: 0.9630591152749226

Term	Count	%	PValue
GO:0004871~signal transducer activity	14	11.76%	0.94688905

GO:0060089~molecular transducer activity	14	11.76%	0.94688905
GO:0004872~receptor activity	9	7.56%	0.99623236

Table S5

Median: 6.748340700062148E-4	Geo: 6.426805056597486E-4		
Term	Count	%	PValue
GO:0032502~developmental process	45	29.61%	4.78E-06
GO:0048869~cellular developmental process	30	19.74%	2.99E-05
GO:0030154~cell differentiation	30	19.74%	2.99E-05
GO:0007275~multicellular organismal development	32	21.05%	5.16E-04
GO:0048731~system development	28	18.42%	6.75E-04
GO:0048856~anatomical structure development	30	19.74%	0.00152982
GO:0048513~organ development	22	14.47%	0.00159909
GO:0009653~anatomical structure morphogenesis	15	9.87%	0.05367044
GO:0032501~multicellular organismal process	43	28.29%	0.09576885

Median: 0.0036852104896518256	Geo: 0.00177884271287579		
Term	Count	%	PValue
GO:0042127~regulation of cell proliferation	15	9.87%	8.15E-05
GO:0008283~cell proliferation	18	11.84%	1.21E-04
GO:0008285~negative regulation of cell proliferatior	8	5.26%	0.00304683
GO:0008284~positive regulation of cell proliferation	8	5.26%	0.00432359
GO:0048523~negative regulation of cellular process	16	10.53%	0.01161529
GO:0048519~negative regulation of biological proce	16	10.53%	0.02091638

Median: 0.011553468381934585	Geo: 0.003868567826100308		
Term	Count	%	PValue
GO:0032502~developmental process	45	29.61%	4.78E-06
GO:0048869~cellular developmental process	30	19.74%	2.99E-05
GO:0030154~cell differentiation	30	19.74%	2.99E-05
GO:0048468~cell development	23	15.13%	2.67E-04
GO:0008219~cell death	14	9.21%	0.0066416
GO:0016265~death	14	9.21%	0.0066416
GO:0006915~apoptosis	13	8.55%	0.01155347
GO:0012501~programmed cell death	13	8.55%	0.01272079
GO:0042981~regulation of apoptosis	10	6.58%	0.03207116
GO:0043067~regulation of programmed cell death	10	6.58%	0.03482955
GO:0043066~negative regulation of apoptosis	5	3.29%	0.11766584
GO:0043069~negative regulation of programmed ce	5	3.29%	0.12049239
GO:0006916~anti-apoptosis	3	1.97%	0.37109276

Median: 0.011139084952939873	Geo: 0.004790451489712513		
Term	Count	%	PValue
GO:0048518~positive regulation of biological proces	26	17.11%	1.34E-06
GO:0032502~developmental process	45	29.61%	4.78E-06
GO:0048522~positive regulation of cellular process	23	15.13%	1.10E-05
GO:0065007~biological regulation	55	36.18%	1.57E-05
GO:0050794~regulation of cellular process	46	30.26%	2.45E-05
Transcription	15	9.87%	1.83E-04
GO:0050789~regulation of biological process	47	30.92%	2.50E-04
Transcription regulation	14	9.21%	5.62E-04
GO:0045941~positive regulation of transcription	10	6.58%	7.67E-04

GO:0045935~positive regulation of nucleobase, nucl	10	6.58%	0.00109242
GO:0031325~positive regulation of cellular metaboli	10	6.58%	0.00394122
dna-binding	13	8.55%	0.00417032
GO:0043283~biopolymer metabolic process	45	29.61%	0.00463287
GO:0006355~regulation of transcription, DNA-depen	22	14.47%	0.00601692
GO:0009893~positive regulation of metabolic proces	10	6.58%	0.00619934
GO:0003677~DNA binding	23	15.13%	0.00621432
nucleus	22	14.47%	0.00675776
GO:0016070~RNA metabolic process	26	17.11%	0.00861611
GO:0006351~transcription, DNA-dependent	22	14.47%	0.01101814
GO:0032774~RNA biosynthetic process	22	14.47%	0.01126003
GO:0003676~nucleic acid binding	32	21.05%	0.01151421
GO:0019219~regulation of nucleobase, nucleoside, r	23	15.13%	0.01208922
GO:0045449~regulation of transcription	22	14.47%	0.01618277
GO:0019222~regulation of metabolic process	25	16.45%	0.01911386
GO:0003700~transcription factor activity	14	9.21%	0.01981277
GO:0030528~transcription regulator activity	18	11.84%	0.02085853
GO:0005634~nucleus	35	23.03%	0.02523932
GO:0006139~nucleobase, nucleoside, nucleotide an	32	21.05%	0.02781943
GO:0006350~transcription	22	14.47%	0.03053968
GO:0010468~regulation of gene expression	22	14.47%	0.03448082
GO:0031323~regulation of cellular metabolic proces	23	15.13%	0.03705001
GO:0045893~positive regulation of transcription, DN	6	3.95%	0.05128254
GO:0045944~positive regulation of transcription fror	5	3.29%	0.06361487
GO:0010467~gene expression	27	17.76%	0.11393444
GO:0006357~regulation of transcription from RNA po	7	4.61%	0.14555294
GO:0016563~transcription activator activity	5	3.29%	0.20323926
GO:0006366~transcription from RNA polymerase II p	7	4.61%	0.30991157
GO:0046983~protein dimerization activity	4	2.63%	0.5722885

Median: 0.00888381322916805 Geo: 0.005713472237800778

Term	Count	%	PValue
GO:0046872~metal ion binding	38	25.00%	9.66E-04
GO:0043167~ion binding	38	25.00%	0.00152226
GO:0046914~transition metal ion binding	26	17.11%	0.00400902
GO:0008270~zinc ion binding	20	13.16%	0.0137586
GO:0043169~cation binding	32	21.05%	0.01799826
metal-binding	18	11.84%	0.02383083

Median: 0.004267893538795904 Geo: 0.005825031646840147

Term	Count	%	PValue
zinc finger	6	3.95%	2.17E-05
DNA binding	7	4.61%	5.82E-04
zinc finger region:C2H2-type 3	4	2.63%	0.00360219
zinc finger region:C2H2-type 2	4	2.63%	0.00403583
zinc finger region:C2H2-type 1	4	2.63%	0.00449996
zinc-finger	9	5.92%	0.07610532
IPR007087:Zinc finger, C2H2-type	5	3.29%	0.10183541
zinc	10	6.58%	0.20726519

Median: 0.007826510251834073 Geo: 0.007611786276558587

Term	Count	%	PValue
GO:0001569~patterning of blood vessels	4	2.63%	2.96E-04
GO:0007389~pattern specification process	7	4.61%	0.00204985
GO:0048646~anatomical structure formation	7	4.61%	0.00218479
GO:0009880~embryonic pattern specification	4	2.63%	0.00361935
GO:0001525~angiogenesis	6	3.95%	0.00412935
GO:0048754~branching morphogenesis of a tube	4	2.63%	0.00484675
GO:0001763~morphogenesis of a branching structu	4	2.63%	0.00669642
GO:0009887~organ morphogenesis	10	6.58%	0.00782651
GO:0048514~blood vessel morphogenesis	6	3.95%	0.01028697
GO:0001568~blood vessel development	6	3.95%	0.01730714
GO:0035295~tube development	6	3.95%	0.0176862
GO:0001944~vasculature development	6	3.95%	0.01885633
GO:0035239~tube morphogenesis	5	3.29%	0.0231404
GO:0009790~embryonic development	7	4.61%	0.044897
GO:0009653~anatomical structure morphogenesis	15	9.87%	0.05367044

Median: 0.05457297922436276 Geo: 0.014620536960131459

Term	Count	%	PValue
zinc finger	6	3.95%	2.17E-05
IPR003070:Orphan nuclear receptor	3	1.97%	2.97E-04
PIRSF002524:probable hormone receptor N10, nucle	3	1.97%	3.95E-04
DNA binding	7	4.61%	5.82E-04
region of interest:Ligand-binding	3	1.97%	0.02103787
IPR001723:Steroid hormone receptor	3	1.97%	0.05028855
zinc finger region:NR C4-type	3	1.97%	0.05111912
DNA-binding region:Nuclear receptor	3	1.97%	0.05111912
IPR001628:Zinc finger, nuclear hormone receptor-ty	3	1.97%	0.05802684
IPR000536:Nuclear hormone receptor, ligand-bindin	3	1.97%	0.05802684
IPR008946:Nuclear hormone receptor, ligand-bindin	3	1.97%	0.05802684
GO:0003707~steroid hormone receptor activity	3	1.97%	0.06048219
SM00430:HOLI	3	1.97%	0.07872766
SM00399:ZnF_C4	3	1.97%	0.07872766
GO:0043565~sequence-specific DNA binding	7	4.61%	0.11289371
GO:0004879~ligand-dependent nuclear receptor act	3	1.97%	0.12910237

Median: 0.027819433619917497 Geo: 0.0306394706702835

Term	Count	%	PValue
GO:0043283~biopolymer metabolic process	45	29.61%	0.00463287
GO:0009987~cellular process	101	66.45%	0.01801886
GO:0044237~cellular metabolic process	64	42.11%	0.02644205
GO:0006139~nucleobase, nucleoside, nucleotide an	32	21.05%	0.02781943
GO:0044238~primary metabolic process	62	40.79%	0.07000976
GO:0008152~metabolic process	68	44.74%	0.0740469
GO:0043170~macromolecule metabolic process	54	35.53%	0.07963106

Median: 0.043534823014015345 Geo: 0.042953132282386765

Term	Count	%	PValue
GO:0019932~second-messenger-mediated signaling	7	4.61%	0.0298774
GO:0007200~G-protein signaling, coupled to IP3 sec	4	2.63%	0.03872718
GO:0043085~positive regulation of catalytic activity	6	3.95%	0.04834246
GO:0048015~phosphoinositide-mediated signaling	4	2.63%	0.06085438

Median: 0.04555127796655499 Geo: 0.04839470743609883

Term	Count	%	PValue
GO:0030335~positive regulation of cell migration	3	1.97%	0.01085114
GO:0040017~positive regulation of locomotion	3	1.97%	0.01842662
GO:0051272~positive regulation of cell motility	3	1.97%	0.01842662
GO:0016477~cell migration	7	4.61%	0.03362988
GO:0006928~cell motility	8	5.26%	0.04555128
GO:0051674~localization of cell	8	5.26%	0.04555128
GO:0030334~regulation of cell migration	3	1.97%	0.10050712
GO:0051270~regulation of cell motility	3	1.97%	0.12495972
GO:0040012~regulation of locomotion	3	1.97%	0.14485459
GO:0040011~locomotion	3	1.97%	0.15065598

Median: 0.04022831394862833 Geo: 0.04844946335952177

Term	Count	%	PValue
GO:0005622~intracellular	77	50.66%	0.00474
GO:0043231~intracellular membrane-bound organel	54	35.53%	0.01200889
GO:0043227~membrane-bound organelle	54	35.53%	0.01229198
GO:0005634~nucleus	35	23.03%	0.02523932
GO:0044424~intracellular part	68	44.74%	0.03572386
GO:0043229~intracellular organelle	59	38.82%	0.04022831
GO:0043226~organelle	59	38.82%	0.0417487
GO:0005737~cytoplasm	42	27.63%	0.11320826
GO:0044464~cell part	105	69.08%	0.23012183
GO:0005623~cell	105	69.08%	0.23111091
GO:0044444~cytoplasmic part	27	17.76%	0.54126325

Median: 0.06545635625828403 Geo: 0.04962889637919206

Term	Count	%	PValue
GO:0050790~regulation of catalytic activity	10	6.58%	0.0077088
GO:0032147~activation of protein kinase activity	4	2.63%	0.0132609
GO:0065009~regulation of a molecular function	10	6.58%	0.01587624
GO:0045859~regulation of protein kinase activity	6	3.95%	0.02954193
GO:0043549~regulation of kinase activity	6	3.95%	0.03284997
GO:0051338~regulation of transferase activity	6	3.95%	0.0369918
GO:0007243~protein kinase cascade	8	5.26%	0.0386475
GO:0043085~positive regulation of catalytic activity	6	3.95%	0.04834246
GO:0006469~negative regulation of protein kinase a	3	1.97%	0.08257025
GO:0033673~negative regulation of kinase activity	3	1.97%	0.08257025
GO:0051348~negative regulation of transferase acti	3	1.97%	0.08758647
GO:0045860~positive regulation of protein kinase ac	4	2.63%	0.0941953
GO:0033674~positive regulation of kinase activity	4	2.63%	0.10092596
GO:0051347~positive regulation of transferase activ	4	2.63%	0.10960265

GO:0051336~regulation of hydrolase activity	4	2.63%	0.12777791
GO:0043086~negative regulation of catalytic activity	3	1.97%	0.15650345

Median: 0.14206229159700978 Geo: 0.07125983280189296

Term	Count	%	PValue
GO:0007242~intracellular signaling cascade	22	14.47%	0.00196264
GO:0007154~cell communication	45	29.61%	0.08178314
GO:0007165~signal transduction	39	25.66%	0.20234145
GO:0007166~cell surface receptor linked signal tran	20	13.16%	0.79394355

Median: 0.10637149401814736 Geo: 0.0849474832216158

Term	Count	%	PValue
GO:0000902~cell morphogenesis	10	6.58%	0.01669842
GO:0032989~cellular structure morphogenesis	10	6.58%	0.01669842
GO:0016477~cell migration	7	4.61%	0.03362988
GO:0006928~cell motility	8	5.26%	0.04555128
GO:0051674~localization of cell	8	5.26%	0.04555128
GO:0030030~cell projection organization and bioger	6	3.95%	0.05990029
GO:0048858~cell projection morphogenesis	6	3.95%	0.05990029
GO:0032990~cell part morphogenesis	6	3.95%	0.05990029
GO:0022008~neurogenesis	7	4.61%	0.10172001
GO:0030182~neuron differentiation	6	3.95%	0.11102298
GO:0016043~cellular component organization and b	26	17.11%	0.11236985
GO:0007409~axonogenesis	4	2.63%	0.13916871
GO:0048699~generation of neurons	6	3.95%	0.1651285
GO:0048812~neurite morphogenesis	4	2.63%	0.1752294
GO:0048667~neuron morphogenesis during differen	4	2.63%	0.1752294
GO:0000904~cellular morphogenesis during differen	4	2.63%	0.21139848
GO:0031175~neurite development	4	2.63%	0.23117678
GO:0048666~neuron development	4	2.63%	0.28990286

Median: 0.09104503852992898 Geo: 0.0908244416758216

Term	Count	%	PValue
GO:0046649~lymphocyte activation	5	3.29%	0.03889336
GO:0042110~T cell activation	4	2.63%	0.05815535
GO:0045321~leukocyte activation	5	3.29%	0.08376054
GO:0002376~immune system process	11	7.24%	0.0842084
GO:0046651~lymphocyte proliferation	3	1.97%	0.09788168
GO:0032943~mononuclear cell proliferation	3	1.97%	0.09788168
GO:0001775~cell activation	5	3.29%	0.1162637
GO:0006955~immune response	7	4.61%	0.26056428

Median: 0.10263827467169256 Geo: 0.1099454504314445

Term	Count	%	PValue
protein phosphatase	4	2.63%	0.01933435
domain:Tyrosine-protein phosphatase	3	1.97%	0.03341908
active site:Phosphocysteine intermediate	3	1.97%	0.04191161
GO:0006470~protein amino acid dephosphorylation	4	2.63%	0.08289082
IPR000387:Protein-tyrosine phosphatase, Tyr-specific	3	1.97%	0.08945855

GO:0042578~phosphoric ester hydrolase activity	6	3.95%	0.09679904
GO:0016311~dephosphorylation	4	2.63%	0.10263827
GO:0016788~hydrolase activity, acting on ester bon	9	5.92%	0.10830484
GO:0004721~phosphoprotein phosphatase activity	4	2.63%	0.11146389
GO:0004725~protein tyrosine phosphatase activity	3	1.97%	0.12631583
GO:0016791~phosphoric monoester hydrolase activi	4	2.63%	0.28627894
GO:0016787~hydrolase activity	18	11.84%	0.49881661
hydrolase	7	4.61%	0.78952711

Median: 0.07802447275373386 Geo: 0.11298829618483724

Term	Count	%	PValue
GO:0048168~regulation of neuronal synaptic plastic	3	1.97%	0.03105792
GO:0007268~synaptic transmission	8	5.26%	0.04555128
GO:0007267~cell-cell signaling	11	7.24%	0.06121429
GO:0048167~regulation of synaptic plasticity	3	1.97%	0.07522256
GO:0019226~transmission of nerve impulse	8	5.26%	0.08082638
GO:0050803~regulation of synapse structure and ac	3	1.97%	0.08758647
GO:0003008~system process	18	11.84%	0.71987716
GO:0050877~neurological system process	15	9.87%	0.80010184

Median: 0.07946974656261849 Geo: 0.12741174769041366

Term	Count	%	PValue
GO:0007049~cell cycle	9	5.92%	0.06925923
cell cycle	5	3.29%	0.07650756
GO:0000074~regulation of progression through cell (	6	3.95%	0.07661957
GO:0051726~regulation of cell cycle	6	3.95%	0.07946975
GO:0022402~cell cycle process	8	5.26%	0.08008684
GO:0000278~mitotic cell cycle	4	2.63%	0.32204552
GO:0022403~cell cycle phase	3	1.97%	0.65503475

Median: 0.12910236754218637 Geo: 0.1315662955643494

Term	Count	%	PValue
GO:0004620~phospholipase activity	3	1.97%	0.09416653
GO:0016298~lipase activity	3	1.97%	0.12910237
GO:0004091~carboxylesterase activity	3	1.97%	0.18732821

Median: 0.23450793810192835 Geo: 0.1421944033974187

Term	Count	%	PValue
GO:0042981~regulation of apoptosis	10	6.58%	0.03207116
GO:0043067~regulation of programmed cell death	10	6.58%	0.03482955
GO:0006917~induction of apoptosis	4	2.63%	0.23339623
GO:0012502~induction of programmed cell death	4	2.63%	0.23561965
GO:0043065~positive regulation of apoptosis	4	2.63%	0.36341037
GO:0043068~positive regulation of programmed cel	4	2.63%	0.37027961

Median: 0.21298981099252623 Geo: 0.14655099926417334

Term	Count	%	PValue
GO:0043687~post-translational protein modification	17	11.18%	0.03447465
GO:0043412~biopolymer modification	19	12.50%	0.05642001

GO:0006464~protein modification process	18	11.84%	0.07583392
GO:0043170~macromolecule metabolic process	54	35.53%	0.07963106
GO:0006793~phosphorus metabolic process	11	7.24%	0.21298981
GO:0006796~phosphate metabolic process	11	7.24%	0.21298981
GO:0044260~cellular macromolecule metabolic proc	28	18.42%	0.36408905
GO:0019538~protein metabolic process	29	19.08%	0.38759877
GO:0044267~cellular protein metabolic process	27	17.76%	0.41467928

Median: 0.14499032954436994 Geo: 0.15202648386524137

Term	Count	%	PValue
GO:0040007~growth	6	3.95%	0.09774778
GO:0016049~cell growth	4	2.63%	0.13343014
GO:0008361~regulation of cell size	4	2.63%	0.14499033
GO:0040008~regulation of growth	4	2.63%	0.16699377
GO:0001558~regulation of cell growth	3	1.97%	0.25715566

Median: 0.28092879778048224 Geo: 0.15998774749004374

Term	Count	%	PValue
GO:0035295~tube development	6	3.95%	0.0176862
GO:0045934~negative regulation of nucleobase, nuc	5	3.29%	0.17765547
GO:0031324~negative regulation of cellular metabo	5	3.29%	0.2809288
GO:0016481~negative regulation of transcription	4	2.63%	0.33814868
GO:0009892~negative regulation of metabolic proce	5	3.29%	0.35116929

Median: 0.21679396770072448 Geo: 0.17254682489492937

Term	Count	%	PValue
GO:0007631~feeding behavior	4	2.63%	0.0132609
GO:0019932~second-messenger-mediated signaling	7	4.61%	0.0298774
GO:0006873~cellular ion homeostasis	5	3.29%	0.14107926
GO:0055082~cellular chemical homeostasis	5	3.29%	0.14107926
GO:0007204~elevation of cytosolic calcium ion conc	3	1.97%	0.14485459
GO:0051480~cytosolic calcium ion homeostasis	3	1.97%	0.15357415
GO:0050801~ion homeostasis	5	3.29%	0.17931515
GO:0030005~cellular di-, tri-valent inorganic cation l	4	2.63%	0.18779203
GO:0055066~di-, tri-valent inorganic cation homeost	4	2.63%	0.18779203
GO:0048878~chemical homeostasis	5	3.29%	0.24579591
GO:0030003~cellular cation homeostasis	4	2.63%	0.24679262
GO:0055080~cation homeostasis	4	2.63%	0.24679262
GO:0019725~cellular homeostasis	5	3.29%	0.2753221
GO:0042592~homeostatic process	6	3.95%	0.3060075
GO:0006874~cellular calcium ion homeostasis	3	1.97%	0.31923838
GO:0055074~calcium ion homeostasis	3	1.97%	0.31923838
GO:0006875~cellular metal ion homeostasis	3	1.97%	0.3590126
GO:0055065~metal ion homeostasis	3	1.97%	0.3590126

Median: 0.21687007947550468 Geo: 0.18081047887105914

Term	Count	%	PValue
GO:0002376~immune system process	11	7.24%	0.0842084
GO:0030097~hemopoiesis	4	2.63%	0.19629554

GO:0002521~leukocyte differentiation	3	1.97%	0.21687008
GO:0048534~hemopoietic or lymphoid organ develo	4	2.63%	0.22454334
GO:0002520~immune system development	4	2.63%	0.24007799

Median: 0.1515089135837126 Geo: 0.18575887357629942

Term	Count	%	PValue
GO:0006665~sphingolipid metabolic process	3	1.97%	0.0612205
GO:0006643~membrane lipid metabolic process	4	2.63%	0.13724646
GO:0044255~cellular lipid metabolic process	8	5.26%	0.15150891
GO:0006629~lipid metabolic process	8	5.26%	0.28070626
GO:0008610~lipid biosynthetic process	3	1.97%	0.61895567

Median: 0.31306330276550215 Geo: 0.20213993292493965

Term	Count	%	PValue
GO:0009888~tissue development	8	5.26%	0.03004377
GO:0001501~skeletal development	5	3.29%	0.14566491
GO:0001503~ossification	3	1.97%	0.3130633
GO:0031214~biomineral formation	3	1.97%	0.3130633
GO:0046849~bone remodeling	3	1.97%	0.38009492
GO:0048771~tissue remodeling	3	1.97%	0.41845332

Median: 0.22612678300387035 Geo: 0.20522610538248465

Term	Count	%	PValue
GO:0007626~locomotory behavior	4	2.63%	0.16904168
GO:0006935~chemotaxis	3	1.97%	0.22612678
GO:0042330~taxis	3	1.97%	0.22612678

Median: 0.18718910532733748 Geo: 0.2140013630480883

Term	Count	%	PValue
disulfide bond	18	11.84%	0.09644361
GO:0005576~extracellular region	24	15.79%	0.10978214
GO:0044421~extracellular region part	21	13.82%	0.1520038
GO:0005615~extracellular space	20	13.16%	0.15518646
signal	18	11.84%	0.21919175
glycoprotein	20	13.16%	0.36803111
Secreted	9	5.92%	0.43196977
signal peptide	16	10.53%	0.50542507

Median: 0.28744567816976296 Geo: 0.24435719127448727

Term	Count	%	PValue
IPR007087:Zinc finger, C2H2-type	5	3.29%	0.10183541
IPR013087:Zinc finger, C2H2-type/integrase, DNA-bi	3	1.97%	0.22072902
IPR015880:Zinc finger, C2H2-like	3	1.97%	0.35416233
SM00355:ZnF_C2H2	3	1.97%	0.44785751

Median: 0.35873012641214763 Geo: 0.2739158732596765

Term	Count	%	PValue
GO:0004857~enzyme inhibitor activity	5	3.29%	0.1570748
GO:0004866~endopeptidase inhibitor activity	3	1.97%	0.35873013

GO:0030414~protease inhibitor activity	3	1.97%	0.36473484

Median: 0.25732520791296887	Geo: 0.3070428593235986		
Term	Count	%	PValue
GO:0006397~mRNA processing	4	2.63%	0.13533357
GO:0005681~spliceosome	3	1.97%	0.14319607
GO:0016071~mRNA metabolic process	4	2.63%	0.19416055
GO:0006396~RNA processing	5	3.29%	0.21707126
GO:0008380~RNA splicing	3	1.97%	0.29757916
GO:0044428~nuclear part	7	4.61%	0.61839088
GO:0030529~ribonucleoprotein complex	5	3.29%	0.6720952
GO:0003723~RNA binding	4	2.63%	0.78198005

Median: 0.3776840545803085	Geo: 0.35546088644285123		
Term	Count	%	PValue
Postsynaptic cell membrane	3	1.97%	0.24967477
cell junction	4	2.63%	0.37768405
synapse	3	1.97%	0.47629076

Median: 0.4350696695030194	Geo: 0.3726779361542909		
Term	Count	%	PValue
lipoprotein	6	3.95%	0.25929439
Palmitate	3	1.97%	0.43506967
lipid moiety-binding region:S-palmitoyl cysteine	3	1.97%	0.45882716

Median: 0.6550347493280828	Geo: 0.3930222011751995		
Term	Count	%	PValue
GO:0051704~multi-organism process	5	3.29%	0.11907542
GO:0022414~reproductive process	3	1.97%	0.65503475
GO:0000003~reproduction	4	2.63%	0.77833184

Median: 0.5887866746955883	Geo: 0.40006057508686044		
Term	Count	%	PValue
GO:0000287~magnesium ion binding	8	5.26%	0.00212904
GO:0016301~kinase activity	12	7.89%	0.09401846
magnesium	5	3.29%	0.11028258
GO:0016772~transferase activity, transferring phosp	12	7.89%	0.20462216
GO:0006793~phosphorus metabolic process	11	7.24%	0.21298981
GO:0006796~phosphate metabolic process	11	7.24%	0.21298981
kinase	7	4.61%	0.24441827
GO:0016773~phosphotransferase activity, alcohol gı	9	5.92%	0.27451616
SM00220:S_TKc	6	3.95%	0.28396519
GO:0016740~transferase activity	17	11.18%	0.2851711
IPR002290:Serine/threonine protein kinase	3	1.97%	0.40439199
GO:0004672~protein kinase activity	7	4.61%	0.44285187
binding site:ATP	4	2.63%	0.45273372
IPR000719:Protein kinase, core	4	2.63%	0.5317224
IPR008271:Serine/threonine protein kinase, active si	3	1.97%	0.55142859
Serine/threonine-protein kinase	3	1.97%	0.57400518

GO:0016310~phosphorylation	7	4.61%	0.60356817
GO:0004674~protein serine/threonine kinase activity	5	3.29%	0.61344031
GO:0006468~protein amino acid phosphorylation	6	3.95%	0.62083328
active site:Proton acceptor	4	2.63%	0.66904821
domain:Protein kinase	3	1.97%	0.68221601
nucleotide phosphate-binding region:ATP	4	2.63%	0.68868595
nucleotide-binding	8	5.26%	0.69231599
atp-binding	6	3.95%	0.71410301
GO:0005524~ATP binding	9	5.92%	0.82230441
GO:0032559~adenyl ribonucleotide binding	9	5.92%	0.83617534
GO:0000166~nucleotide binding	13	8.55%	0.84942789
transferase	6	3.95%	0.87435008
GO:0030554~adenyl nucleotide binding	9	5.92%	0.87489505
GO:0032555~purine ribonucleotide binding	10	6.58%	0.90630926
GO:0032553~ribonucleotide binding	10	6.58%	0.90630926
GO:0017076~purine nucleotide binding	10	6.58%	0.93066493

Median: 0.38568296458186957	Geo: 0.4004411400930015		
Term	Count	%	PValue
GO:0005764~lysosome	3	1.97%	0.38252226
GO:0000323~lytic vacuole	3	1.97%	0.38568296
GO:0005773~vacuole	3	1.97%	0.43524009

Median: 0.6351311617719959	Geo: 0.4636426277900228		
Term	Count	%	PValue
transducer	10	6.58%	0.02538471
g-protein coupled receptor	8	5.26%	0.09168277
GO:0001653~peptide receptor activity	4	2.63%	0.09275957
GO:0042277~peptide binding	5	3.29%	0.09441662
rno04080:Neuroactive ligand-receptor interaction	5	3.29%	0.23679494
membrane	30	19.74%	0.2394147
IPR000276:Rhodopsin-like GPCR superfamily	7	4.61%	0.24170315
G protein-coupled receptor	3	1.97%	0.24587694
GO:0005886~plasma membrane	20	13.16%	0.25253032
glycoprotein	20	13.16%	0.36803111
receptor	12	7.89%	0.40674756
GO:0044459~plasma membrane part	14	9.21%	0.54022955
GO:0005887~integral to plasma membrane	9	5.92%	0.56466453
GO:0031226~intrinsic to plasma membrane	9	5.92%	0.58241564
glycosylation site:N-linked (GlcNAc...)	17	11.18%	0.61271877
transmembrane	21	13.82%	0.6131457
transmembrane protein	5	3.29%	0.65711662
topological domain:Extracellular	11	7.24%	0.6586014
topological domain:Cytoplasmic	13	8.55%	0.72610211
GO:0007186~G-protein coupled receptor protein sigı	16	10.53%	0.74139492
GO:0007166~cell surface receptor linked signal tran	20	13.16%	0.79394355
transmembrane region	15	9.87%	0.8266659
GO:0004871~signal transducer activity	20	13.16%	0.92876504
GO:0060089~molecular transducer activity	20	13.16%	0.92876504

GO:0004930~G-protein coupled receptor activity	11	7.24%	0.96003914
GO:0001584~rhodopsin-like receptor activity	9	5.92%	0.9810981
GO:0004888~transmembrane receptor activity	12	7.89%	0.9839537
GO:0004872~receptor activity	15	9.87%	0.98458069
GO:0016020~membrane	41	26.97%	0.9982432
GO:0044425~membrane part	31	20.39%	0.99928109
GO:0016021~integral to membrane	26	17.11%	0.99967323
GO:0031224~intrinsic to membrane	26	17.11%	0.99970986

Median: 0.4708182436304623 Geo: 0.4660174692927841

Term	Count	%	PValue
GO:0030135~coated vesicle	3	1.97%	0.38252226
GO:0016023~cytoplasmic membrane-bound vesicle	4	2.63%	0.45233669
GO:0031988~membrane-bound vesicle	4	2.63%	0.47081824
GO:0031410~cytoplasmic vesicle	4	2.63%	0.49801369
GO:0031982~vesicle	4	2.63%	0.54174871

Median: 0.5977868973943758 Geo: 0.5188412456329615

Term	Count	%	PValue
GO:0006396~RNA processing	5	3.29%	0.21707126
GO:0043233~organelle lumen	8	5.26%	0.25090659
GO:0031974~membrane-enclosed lumen	8	5.26%	0.25090659
rna-binding	3	1.97%	0.45932768
GO:0031981~nuclear lumen	5	3.29%	0.54864712
GO:0005654~nucleoplasm	4	2.63%	0.57718292
GO:0044428~nuclear part	7	4.61%	0.61839088
GO:0030529~ribonucleoprotein complex	5	3.29%	0.6720952
GO:0044451~nucleoplasm part	3	1.97%	0.72030841
GO:0044446~intracellular organelle part	17	11.18%	0.83082855
GO:0044422~organelle part	17	11.18%	0.83519455
GO:0032991~macromolecular complex	15	9.87%	0.92160933

Median: 0.5316807174174902 Geo: 0.5256863931597372

Term	Count	%	PValue
GO:0006954~inflammatory response	4	2.63%	0.47305709
GO:0006950~response to stress	10	6.58%	0.50424836
GO:0006952~defense response	5	3.29%	0.55911307
GO:0009611~response to wounding	5	3.29%	0.57259724

Median: 0.6050691824575416 Geo: 0.5538417602513612

Term	Count	%	PValue
GO:0005739~mitochondrion	9	5.92%	0.38018834
GO:0044429~mitochondrial part	4	2.63%	0.60506918
Mitochondrion	4	2.63%	0.7385047

Median: 0.6203505593051134 Geo: 0.5574080784731396

Term	Count	%	PValue
GO:0005578~proteinaceous extracellular matrix	4	2.63%	0.34465906
GO:0031012~extracellular matrix	4	2.63%	0.36653473

GO:0022610~biological adhesion	4	2.63%	0.87416638
GO:0007155~cell adhesion	4	2.63%	0.87416638

Median: 0.6077307778369707 Geo: 0.6401225395288845

Term	Count	%	PValue
GO:0051179~localization	25	16.45%	0.45835904
GO:0046907~intracellular transport	7	4.61%	0.47664479
GO:0033036~macromolecule localization	7	4.61%	0.50456307
GO:0006605~protein targeting	3	1.97%	0.55097123
GO:0051649~establishment of cellular localization	8	5.26%	0.56384228
GO:0051641~cellular localization	8	5.26%	0.57663052
GO:0008104~protein localization	6	3.95%	0.63883103
GO:0006886~intracellular protein transport	4	2.63%	0.64815115
GO:0045184~establishment of protein localization	5	3.29%	0.7541813
GO:0015031~protein transport	4	2.63%	0.85437133
GO:0051234~establishment of localization	16	10.53%	0.92870858
GO:0006810~transport	14	9.21%	0.96737074

Median: 0.7180410061501701 Geo: 0.6532110505514424

Term	Count	%	PValue
GO:0005506~iron ion binding	4	2.63%	0.40215727
iron	3	1.97%	0.58274416
GO:0016491~oxidoreductase activity	6	3.95%	0.85333785
oxidoreductase	3	1.97%	0.9103727

Median: 0.7898650649158409 Geo: 0.8021733696316998

Term	Count	%	PValue
GO:0006508~proteolysis	6	3.95%	0.73437339
GO:0008233~peptidase activity	5	3.29%	0.78986506
GO:0004175~endopeptidase activity	3	1.97%	0.88988704

Median: 0.970335217397613 Geo: 0.9247462277976668

Term	Count	%	PValue
GO:0008092~cytoskeletal protein binding	3	1.97%	0.79393513
GO:0005856~cytoskeleton	4	2.63%	0.9623272
GO:0043228~non-membrane-bound organelle	8	5.26%	0.97834324
GO:0043232~intracellular non-membrane-bound org	8	5.26%	0.97834324

Median: 0.9839158915997125 Geo: 0.9253029306672969

Term	Count	%	PValue
GO:0015267~channel activity	3	1.97%	0.81163057
GO:0022803~passive transmembrane transporter ac	3	1.97%	0.81163057
GO:0022857~transmembrane transporter activity	4	2.63%	0.9832207
GO:0005215~transporter activity	6	3.95%	0.98461108
GO:0022892~substrate-specific transporter activity	4	2.63%	0.99122499
GO:0022891~substrate-specific transmembrane trar	3	1.97%	0.9928848

Median: 0.9998834069831795 Geo: 0.9976789972437883

Term	Count	%	PValue

GO:0007600~sensory perception	7	4.61%	0.99325095
GO:0007608~sensory perception of smell	3	1.97%	0.99988341
GO:0007606~sensory perception of chemical stimulu	3	1.97%	0.99991743

Table S6

Median: 7.044309624431602E-9 Geo: 1.1045055549475803E-8

Term	Count	%	PValue
GO:0005622~intracellular	501	44.81%	1.21E-19
GO:0044424~intracellular part	453	40.52%	3.90E-14
GO:0005737~cytoplasm	291	26.03%	8.02E-10
GO:0043227~membrane-bound organelle	333	29.79%	1.05E-09
GO:0043231~intracellular membrane-bound o	332	29.70%	1.48E-09
GO:0043226~organelle	377	33.72%	7.04E-09
GO:0043229~intracellular organelle	376	33.63%	8.62E-09
GO:0044444~cytoplasmic part	215	19.23%	2.79E-06
GO:0005634~nucleus	184	16.46%	0.005189
GO:0005623~cell	652	58.32%	0.006045
GO:0044464~cell part	651	58.23%	0.009543

Median: 3.542056092261983E-5 Geo: 3.775890795601803E-5

Term	Count	%	PValue
GO:0016192~vesicle-mediated transport	55	4.92%	1.25E-07
GO:0015031~protein transport	64	5.72%	1.64E-07
GO:0045184~establishment of protein localiz	67	5.99%	2.77E-07
GO:0008104~protein localization	69	6.17%	7.17E-07
GO:0033036~macromolecule localization	70	6.26%	1.71E-06
GO:0006886~intracellular protein transport	47	4.20%	3.20E-06
GO:0016043~cellular component organization	181	16.19%	6.81E-06
GO:0046907~intracellular transport	64	5.72%	3.54E-05
GO:0051649~establishment of cellular localiz	74	6.62%	2.04E-04
GO:0051641~cellular localization	74	6.62%	2.87E-04
GO:0006810~transport	169	15.12%	5.43E-04
GO:0051234~establishment of localization	174	15.56%	6.17E-04
GO:0051179~localization	194	17.35%	8.66E-04
GO:0032940~secretion by cell	26	2.33%	0.044288
GO:0005215~transporter activity	81	7.25%	0.112579

Median: 0.0011776838916593433 Geo: 6.354060560924282E-4

Term	Count	%	PValue
GO:0031090~organelle membrane	61	5.46%	1.14E-04
GO:0044422~organelle part	153	13.69%	0.001178
GO:0044446~intracellular organelle part	151	13.51%	0.001906

Median: 0.00409552834425737 Geo: 0.0011073699870049756

Term	Count	%	PValue
GO:0016791~phosphoric monoester hydrolas	31	2.77%	5.27E-06
GO:0042578~phosphoric ester hydrolase acti	35	3.13%	2.72E-05
GO:0016788~hydrolase activity, acting on est	56	5.01%	4.15E-05
GO:0016311~dephosphorylation	17	1.52%	0.004096
GO:0004721~phosphoprotein phosphatase ac	16	1.43%	0.009877
GO:0006470~protein amino acid dephosphory	14	1.25%	0.022158
GO:0004725~protein tyrosine phosphatase ac	6	0.54%	0.383037

Median: 0.003228915351500921	Geo: 0.0014419553823479551		
Term	Count	%	PValue
GO:0044238~primary metabolic process	417	37.30%	1.56E-05
GO:0008152~metabolic process	458	40.97%	2.05E-05
GO:0044237~cellular metabolic process	411	36.76%	4.95E-05
GO:0019538~protein metabolic process	218	19.50%	6.51E-04
GO:0043170~macromolecule metabolic proce	348	31.13%	0.002058
GO:0009987~cellular process	658	58.86%	0.002091
GO:0044267~cellular protein metabolic proce	199	17.80%	0.004366
GO:0044260~cellular macromolecule metabo	202	18.07%	0.004398
GO:0043412~biopolymer modification	104	9.30%	0.010652
GO:0006464~protein modification process	101	9.03%	0.011152
GO:0043283~biopolymer metabolic process	239	21.38%	0.015262
GO:0043687~post-translational protein modif	81	7.25%	0.052405

Median: 0.0014458326006580122	Geo: 0.0018374799149451773		
Term	Count	%	PValue
GO:0004437~inositol or phosphatidylinositol p	9	0.81%	2.56E-04
GO:0042577~lipid phosphatase activity	5	0.45%	0.001446
GO:0004439~phosphoinositide 5-phosphatase	3	0.27%	0.016744

Median: 0.023947488057755185	Geo: 0.0019884476899413527		
Term	Count	%	PValue
GO:0016192~vesicle-mediated transport	55	4.92%	1.25E-07
GO:0048193~Golgi vesicle transport	13	1.16%	0.007169
GO:0045045~secretory pathway	24	2.15%	0.023947
GO:0046903~secretion	33	2.95%	0.032828
GO:0032940~secretion by cell	26	2.33%	0.044288

Median: 0.0016357492368776236	Geo: 0.0032548360076113886		
Term	Count	%	PValue
GO:0014069~postsynaptic density	8	0.72%	5.29E-04
GO:0042734~presynaptic membrane	7	0.63%	0.001636
GO:0032279~asymmetric synapse	3	0.27%	0.039821

Median: 0.004086253621505005	Geo: 0.003681953265631834		
Term	Count	%	PValue
GO:0006807~nitrogen compound metabolic p	44	3.94%	3.81E-04
GO:0006519~amino acid and derivative meta	38	3.40%	5.58E-04
GO:0009308~amine metabolic process	41	3.67%	7.89E-04
GO:0006520~amino acid metabolic process	27	2.42%	0.007384
GO:0019752~carboxylic acid metabolic proce	41	3.67%	0.044166
GO:0006082~organic acid metabolic process	41	3.67%	0.04559

Median: 0.0024659668666173417	Geo: 0.0038986907006064455		
Term	Count	%	PValue
GO:0031988~membrane-bound vesicle	32	2.86%	2.95E-04
GO:0016023~cytoplasmic membrane-bound v	30	2.68%	8.53E-04

GO:0031982~vesicle	32	2.86%	0.001726
GO:0031410~cytoplasmic vesicle	30	2.68%	0.002466
GO:0030135~coated vesicle	17	1.52%	0.008603
GO:0030136~clathrin-coated vesicle	15	1.34%	0.011561
GO:0008021~synaptic vesicle	8	0.72%	0.128535

Median: 0.005697269569420361	Geo: 0.003905295818069365		
Term	Count	%	PValue
GO:0012505~endomembrane system	41	3.67%	4.89E-04
GO:0005789~endoplasmic reticulum membra	15	1.34%	0.004224
GO:0042175~nuclear envelope-endoplasmic r	15	1.34%	0.007171
GO:0044432~endoplasmic reticulum part	16	1.43%	0.01569

Median: 0.005800083031536231	Geo: 0.0045423103766470746		
Term	Count	%	PValue
GO:0065003~macromolecular complex assen	42	3.76%	0.00193
GO:0022607~cellular component assembly	43	3.85%	0.0058
GO:0006461~protein complex assembly	26	2.33%	0.008372

Median: 0.003102987338819654	Geo: 0.005413943633266698		
Term	Count	%	PValue
GO:0000166~nucleotide binding	139	12.43%	4.33E-05
GO:0032553~ribonucleotide binding	114	10.20%	6.94E-04
GO:0032555~purine ribonucleotide binding	114	10.20%	6.94E-04
GO:0017076~purine nucleotide binding	116	10.38%	0.001642
nucleotide-binding	68	6.08%	0.004564
GO:0032559~adenyl ribonucleotide binding	79	7.07%	0.140823
GO:0005524~ATP binding	77	6.89%	0.165516
GO:0030554~adenyl nucleotide binding	81	7.25%	0.202837

Median: 0.004872413994231359	Geo: 0.006380150986284573		
Term	Count	%	PValue
GO:0004437~inositol or phosphatidylinositol p	9	0.81%	2.56E-04
SM00128:IPPc	5	0.45%	0.002727
GO:0046030~inositol trisphosphate phosphat	4	0.36%	0.004872
GO:0004445~inositol-polyphosphate 5-phospl	4	0.36%	0.004872
IPR000300:Inositol polyphosphate related pho	4	0.36%	0.011463
IPR005135:Endonuclease/exonuclease/phosph	4	0.36%	0.354689

Median: 0.01031240595268623	Geo: 0.00687033963953285		
Term	Count	%	PValue
GO:0007049~cell cycle	50	4.47%	3.48E-04
GO:0007050~cell cycle arrest	10	0.89%	0.002501
GO:0045786~negative regulation of progress	15	1.34%	0.002807
GO:0022402~cell cycle process	39	3.49%	0.008623
GO:0000074~regulation of progression throug	26	2.33%	0.012002
GO:0051726~regulation of cell cycle	26	2.33%	0.013531
GO:0000278~mitotic cell cycle	23	2.06%	0.017947

GO:0022403~cell cycle phase	22	1.97%	0.080816

Median: 0.0028084324789411762	Geo: 0.008228285605659864		
Term	Count	%	PValue
GO:0048519~negative regulation of biologica	80	7.16%	0.00239
GO:0048523~negative regulation of cellular p	75	6.71%	0.002808
GO:0009892~negative regulation of metaboli	28	2.50%	0.082998

Median: 0.012960373553618398	Geo: 0.008546649490151655		
Term	Count	%	PValue
GO:0006457~protein folding	23	2.06%	0.001451
chaperone	10	0.89%	0.011169
GO:0051082~unfolded protein binding	12	1.07%	0.014752
IPR002423:Chaperonin Cpn60/TCP-1	4	0.36%	0.022313

Median: 0.9967247590148626	Geo: 0.01098528701294567		
Term	Count	%	PValue
GO:0007242~intracellular signaling cascade	114	10.20%	1.33E-06
GO:0007154~cell communication	218	19.50%	0.996725
GO:0007165~signal transduction	196	17.53%	0.997839

Median: 0.040660299161142804	Geo: 0.012755242088997738		
Term	Count	%	PValue
GO:0016044~membrane organization and bic	29	2.59%	0.001255
GO:0010324~membrane invagination	16	1.43%	0.04066
GO:0006897~endocytosis	16	1.43%	0.04066

Median: 0.10964998236347706	Geo: 0.014021621729689819		
Term	Count	%	PValue
GO:0005525~GTP binding	37	3.31%	7.53E-06
GO:0032561~guanyl ribonucleotide binding	37	3.31%	1.26E-05
GO:0019001~guanyl nucleotide binding	37	3.31%	1.46E-05
GTP-binding	26	2.33%	2.21E-04
GO:0003924~GTPase activity	21	1.88%	4.06E-04
IPR005225:Small GTP-binding protein domain	15	1.34%	0.007434
IPR001806:Ras GTPase	15	1.34%	0.007434
nucleotide phosphate-binding region:GTP	14	1.25%	0.023175
lipid moiety-binding region:S-geranylgeranyl c	7	0.63%	0.041332
short sequence motif:Effector region	7	0.63%	0.062708
GO:0017111~nucleoside-triphosphatase activ	37	3.31%	0.10965
GO:0016462~pyrophosphatase activity	38	3.40%	0.122742
GO:0016818~hydrolase activity, acting on aci	38	3.40%	0.12873
GO:0016817~hydrolase activity, acting on aci	38	3.40%	0.139562
prenylation	8	0.72%	0.152535
IPR013753:Ras	9	0.81%	0.152954
PIRSF037165:ras protein	3	0.27%	0.159725
propeptide:Removed in mature form	9	0.81%	0.175615
Methylation	10	0.89%	0.203346

IPR003577:Ras small GTPase, Ras type	3	0.27%	0.297638
SM00173:RAS	3	0.27%	0.305554

Median: 0.01276382284052376 Geo: 0.01838815303424093

Term	Count	%	PValue
GO:0004774~succinate-CoA ligase activity	3	0.27%	0.008688
GO:0016405~CoA-ligase activity	4	0.36%	0.010767
GO:0016878~acid-thiol ligase activity	4	0.36%	0.014761
GO:0016877~ligase activity, forming carbon-s	4	0.36%	0.082806

Median: 0.017439820365672112 Geo: 0.024768054688680787

Term	Count	%	PValue
IPR000504:RNA recognition motif, RNP-1	14	1.25%	0.012923
rna-binding	18	1.61%	0.014434
IPR012677:Nucleotide-binding, alpha-beta pla	14	1.25%	0.01744
SM00360:RRM	16	1.43%	0.042508
GO:0003723~RNA binding	36	3.22%	0.067407

Median: 0.035923506232415525 Geo: 0.0260307834995732

Term	Count	%	PValue
GO:0044271~nitrogen compound biosynthetic	13	1.16%	0.010719
GO:0009309~amine biosynthetic process	10	0.89%	0.035924
GO:0008652~amino acid biosynthetic process	7	0.63%	0.045807

Median: 0.020676498351106368 Geo: 0.02792059126257564

Term	Count	%	PValue
GO:0005794~Golgi apparatus	31	2.77%	0.015073
GO:0044431~Golgi apparatus part	19	1.70%	0.020676
GO:0000139~Golgi membrane	12	1.07%	0.069837

Median: 0.04179945450168271 Geo: 0.02866270308739085

Term	Count	%	PValue
GO:0051020~GTPase binding	11	0.98%	0.005704
GO:0031267~small GTPase binding	9	0.81%	0.0147
GO:0017016~Ras GTPase binding	7	0.63%	0.068898
GO:0017048~Rho GTPase binding	4	0.36%	0.116835

Median: 0.05672383173871821 Geo: 0.03746482697220645

Term	Count	%	PValue
GO:0007264~small GTPase mediated signal t	43	3.85%	9.56E-06
GO:0005083~small GTPase regulator activity	20	1.79%	7.72E-04
GO:0030695~GTPase regulator activity	27	2.42%	0.002797
GO:0005085~guanyl-nucleotide exchange fac	15	1.34%	0.007996
GO:0051056~regulation of small GTPase med	17	1.52%	0.017757
GO:0008047~enzyme activator activity	20	1.79%	0.02122
GO:0005088~Ras guanyl-nucleotide exchange	9	0.81%	0.025163
GO:0007265~Ras protein signal transduction	17	1.52%	0.031174
GO:0030234~enzyme regulator activity	47	4.20%	0.038539

GO:0046578~regulation of Ras protein signal	12	1.07%	0.039714
GO:0007266~Rho protein signal transduction	10	0.89%	0.056724
GO:0009966~regulation of signal transductio	35	3.13%	0.071205
GO:0005089~Rho guanyl-nucleotide exchang	7	0.63%	0.074098
GO:0005096~GTPase activator activity	12	1.07%	0.085486
GO:0035023~regulation of Rho protein signal	7	0.63%	0.112689
IPR011993:Pleckstrin homology-type	12	1.07%	0.182677
IPR001849:Pleckstrin-like	9	0.81%	0.407613
IPR000219:DH	3	0.27%	0.415812
SM00325:RhoGEF	3	0.27%	0.425544
SM00233:PH	9	0.81%	0.488891
domain:PH	3	0.27%	0.801822

Median: 0.05790452203551337 Geo: 0.03831891115558686

Term	Count	%	PValue
GO:0044463~cell projection part	14	1.25%	0.004533
GO:0043198~dendritic shaft	5	0.45%	0.015482
GO:0042995~cell projection	33	2.95%	0.033403
GO:0043197~dendritic spine	5	0.45%	0.082407
GO:0043005~neuron projection	18	1.61%	0.125864
GO:0030425~dendrite	11	0.98%	0.130214

Median: 0.029417122574276287 Geo: 0.03899252756753423

Term	Count	%	PValue
aminoacyl-tRNA synthetase	6	0.54%	0.018385
GO:0043039~tRNA aminoacylation	9	0.81%	0.024262
GO:0006418~tRNA aminoacylation for protein	9	0.81%	0.024262
GO:0043038~amino acid activation	9	0.81%	0.024262
GO:0006399~tRNA metabolic process	12	1.07%	0.029253
GO:0016875~ligase activity, forming carbon-c	9	0.81%	0.029581
GO:0016876~ligase activity, forming aminoac	9	0.81%	0.029581
GO:0004812~aminoacyl-tRNA ligase activity	9	0.81%	0.029581
rno00970:Aminoacyl-tRNA biosynthesis	4	0.36%	0.116794
protein biosynthesis	11	0.98%	0.349878

Median: 0.07501409807774695 Geo: 0.04573781156503287

Term	Count	%	PValue
GO:0048193~Golgi vesicle transport	13	1.16%	0.007169
GO:0006888~ER to Golgi vesicle-mediated tra	7	0.63%	0.075014
er-golgi transport	5	0.45%	0.177915

Median: 0.06899050268680867 Geo: 0.046416713865919654

Term	Count	%	PValue
outer membrane	6	0.54%	0.010652
GO:0019867~outer membrane	9	0.81%	0.020773
GO:0005741~mitochondrial outer membrane	6	0.54%	0.068991
GO:0031968~organelle outer membrane	6	0.54%	0.118051
topological domain:Mitochondrial intermembra	4	0.36%	0.119556

Median: 0.06513242583564044	Geo: 0.05036987687278405		
Term	Count	%	PValue
GO:0016874~ligase activity	34	3.04%	1.59E-04
GO:0016881~acid-amino acid ligase activity	15	1.34%	0.04563
GO:0019787~small conjugating protein ligase	14	1.25%	0.046039
GO:0016879~ligase activity, forming carbon-n	17	1.52%	0.047902
GO:0004842~ubiquitin-protein ligase activity	12	1.07%	0.050809
GO:0008639~small protein conjugating enzyr	12	1.07%	0.065132
GO:0006512~ubiquitin cycle	21	1.88%	0.111332
GO:0016567~protein ubiquitination	8	0.72%	0.143279
GO:0000209~protein polyubiquitination	3	0.27%	0.168192
rno04120:Ubiquitin mediated proteolysis	12	1.07%	0.18278
GO:0032446~protein modification by small pr	8	0.72%	0.204258

Median: 0.0316532809502792	Geo: 0.05575989202701649		
Term	Count	%	PValue
GO:0048468~cell development	89	7.96%	0.005336
GO:0042981~regulation of apoptosis	44	3.94%	0.011114
GO:0043067~regulation of programmed cell d	44	3.94%	0.01401
GO:0008219~cell death	56	5.01%	0.014591
GO:0016265~death	56	5.01%	0.014591
GO:0048869~cellular developmental process	110	9.84%	0.021203
GO:0030154~cell differentiation	110	9.84%	0.021203
GO:0006915~apoptosis	52	4.65%	0.028182
GO:0012501~programmed cell death	52	4.65%	0.035124
GO:0043066~negative regulation of apoptosis	18	1.61%	0.138967
GO:0043069~negative regulation of programm	18	1.61%	0.146863
GO:0006916~anti-apoptosis	12	1.07%	0.266561
GO:0043065~positive regulation of apoptosis	18	1.61%	0.29451
GO:0043068~positive regulation of programm	18	1.61%	0.311435
GO:0006917~induction of apoptosis	13	1.16%	0.467993
GO:0012502~induction of programmed cell de	13	1.16%	0.475026

Median: 0.1562812317333729	Geo: 0.05770769390815544		
Term	Count	%	PValue
GO:0005856~cytoskeleton	60	5.37%	0.002472
GO:0044430~cytoskeletal part	37	3.31%	0.093461
GO:0043232~intracellular non-membrane-bou	88	7.87%	0.219101
GO:0043228~non-membrane-bound organelle	88	7.87%	0.219101

Median: 0.06825579194341394	Geo: 0.06084916171997176		
Term	Count	%	PValue
IPR006689:ARF/SAR superfamily	5	0.45%	0.041646
IPR006688:ADP-ribosylation factor	4	0.36%	0.066748
SM00177:ARF	4	0.36%	0.069764
PIRSF001711:ADP-ribosylation factor	4	0.36%	0.070693

Median: 0.142736866623424	Geo: 0.0615377040316669		
Term	Count	%	PValue
GO:0065007~biological regulation	277	24.78%	1.82E-05
GO:0050789~regulation of biological process	237	21.20%	6.06E-04
GO:0050794~regulation of cellular process	210	18.78%	0.001453
GO:0005634~nucleus	184	16.46%	0.005189
GO:0003676~nucleic acid binding	160	14.31%	0.018851
GO:0019222~regulation of metabolic process	125	11.18%	0.037235
GO:0006139~nucleobase, nucleoside, nucleot	171	15.30%	0.051081
GO:0003677~DNA binding	96	8.59%	0.081483
GO:0031323~regulation of cellular metabolic	115	10.29%	0.098182
GO:0010468~regulation of gene expression	107	9.57%	0.117897
GO:0010467~gene expression	153	13.69%	0.133621
GO:0016070~RNA metabolic process	116	10.38%	0.151853
Transcription regulation	35	3.13%	0.152907
Transcription	35	3.13%	0.176114
GO:0006350~transcription	100	8.94%	0.289473
GO:0045449~regulation of transcription	94	8.41%	0.302651
GO:0019219~regulation of nucleobase, nucle	95	8.50%	0.390213
GO:0006355~regulation of transcription, DNA	84	7.51%	0.393237
GO:0006351~transcription, DNA-dependent	86	7.69%	0.51837
GO:0032774~RNA biosynthetic process	86	7.69%	0.526486
GO:0030528~transcription regulator activity	66	5.90%	0.563567
GO:0003700~transcription factor activity	43	3.85%	0.721366

Median: 0.2068132443128243	Geo: 0.06384945590624247		
Term	Count	%	PValue
lipoprotein	35	3.13%	0.004159
lipid moiety-binding region:S-palmitoyl cystei	12	1.07%	0.206813
Palmitate	12	1.07%	0.302654

Median: 0.05902448559063275	Geo: 0.06952667627562897		
Term	Count	%	PValue
GO:0008270~zinc ion binding	90	8.05%	0.027333
GO:0043167~ion binding	172	15.38%	0.047907
GO:0046872~metal ion binding	167	14.94%	0.059024
GO:0046914~transition metal ion binding	109	9.75%	0.084958
GO:0043169~cation binding	150	13.42%	0.247418

Median: 0.08069942792163615	Geo: 0.07022645291284828		
Term	Count	%	PValue
lipid moiety-binding region:N-myristoyl glycin	9	0.81%	0.01454
myristate	9	0.81%	0.017341
myristylation	6	0.54%	0.018385
blocked amino end	6	0.54%	0.143013
nucleotide binding	6	0.54%	0.406721
P-loop	6	0.54%	0.444845

Median: 0.052526849789520304 Geo: 0.07343970394205135

Term	Count	%	PValue
GO:0006584~catecholamine metabolic proce	6	0.54%	0.019824
GO:0018958~phenol metabolic process	6	0.54%	0.02286
GO:0006576~biogenic amine metabolic proce	10	0.89%	0.022861
GO:0006575~amino acid derivative metabolic	12	1.07%	0.052527
GO:0042133~neurotransmitter metabolic pro	5	0.45%	0.181854
GO:0042417~dopamine metabolic process	3	0.27%	0.297004
GO:0006725~aromatic compound metabolic p	10	0.89%	0.392012

Median: 0.08857971092806124 Geo: 0.07428537664101921

Term	Count	%	PValue
rno04130:SNARE interactions in vesicular trar	7	0.63%	0.027406
IPR006012:Syntaxin/epimorphin coiled-coil	4	0.36%	0.046104
domain:t-SNARE coiled-coil homology	4	0.36%	0.053705
IPR006011:Syntaxin, N-terminal	3	0.27%	0.08858
SM00503:SynN	3	0.27%	0.091493
IPR000727:Target SNARE coiled-coil region	4	0.36%	0.147697
SM00397:t_SNARE	4	0.36%	0.153687

Median: 0.062429438292995174 Geo: 0.07578489960391194

Term	Count	%	PValue
lipid moiety-binding region:N-myristoyl glycine	9	0.81%	0.01454
myristate	9	0.81%	0.017341
calcium-binding region:1	8	0.72%	0.019181
domain:EF-hand 3	7	0.63%	0.02202
calcium-binding region:2	7	0.63%	0.046167
IPR002048:Calcium-binding EF-hand	14	1.25%	0.056269
domain:EF-hand 2	9	0.81%	0.057434
domain:EF-hand 4	5	0.45%	0.058266
IPR011992:EF-Hand type	14	1.25%	0.062354
SM00054:EFh	14	1.25%	0.062505
PIRSF002350:calmodulin	4	0.36%	0.070693
domain:EF-hand 1	8	0.72%	0.090052
calcium	30	2.68%	0.152543
calcium-binding region:3	3	0.27%	0.246277
IPR001125:Recoverin	3	0.27%	0.273309
Signal transduction mechanisms / Cytoskeleto	6	0.54%	0.282244
EF hand	4	0.36%	0.294715
calcium binding	5	0.45%	0.346603

Median: 0.08419109283311178 Geo: 0.07865101152463738

Term	Count	%	PValue
GO:0008289~lipid binding	27	2.42%	0.034511
GO:0005543~phospholipid binding	15	1.34%	0.084191
GO:0035091~phosphoinositide binding	10	0.89%	0.167453

Median: 0.06749757640491225 Geo: 0.08163632183901466

Term	Count	%	PValue
GO:0006396~RNA processing	26	2.33%	0.021741
GO:0006397~mRNA processing	16	1.43%	0.027483
mrna processing	10	0.89%	0.03976
GO:0016071~mRNA metabolic process	16	1.43%	0.095236
mrna splicing	6	0.54%	0.279462
GO:0008380~RNA splicing	9	0.81%	0.468157

Median: 0.08367445242528601 Geo: 0.09325118835443283

Term	Count	%	PValue
GO:0019226~transmission of nerve impulse	39	3.49%	0.008986
GO:0007268~synaptic transmission	35	3.13%	0.009379
GO:0007267~cell-cell signaling	49	4.38%	0.083674
GO:0003008~system process	69	6.17%	1
GO:0050877~neurological system process	57	5.10%	1

Median: 0.10197451032252433 Geo: 0.09443930654308128

Term	Count	%	PValue
GO:0008285~negative regulation of cell prolif	22	1.97%	0.021171
GO:0042127~regulation of cell proliferation	37	3.31%	0.101975
GO:0008283~cell proliferation	45	4.03%	0.390141

Median: 0.07380752782690993 Geo: 0.09919375989807463

Term	Count	%	PValue
GO:0006536~glutamate metabolic process	3	0.27%	0.072716
rno00251:Glutamate metabolism	5	0.45%	0.073808
GO:0009064~glutamine family amino acid me	5	0.45%	0.181854

Median: 0.15290711979150115 Geo: 0.10087618409325069

Term	Count	%	PValue
repressor	12	1.07%	0.02792
nucleus	91	8.14%	0.033644
Transcription regulation	35	3.13%	0.152907
Transcription	35	3.13%	0.176114
dna-binding	34	3.04%	0.412953

Median: 0.1309296131274359 Geo: 0.10616709332351958

Term	Count	%	PValue
domain:BAR	3	0.27%	0.057758
IPR004148:BAR	3	0.27%	0.13093
SM00721:BAR	3	0.27%	0.158241

Median: 0.10584057003619658 Geo: 0.10679216096906936

Term	Count	%	PValue
GO:0005100~Rho GTPase activator activity	4	0.36%	0.053969
GO:0005096~GTPase activator activity	12	1.07%	0.085486
GO:0043087~regulation of GTPase activity	7	0.63%	0.105841
gtpase activation	5	0.45%	0.141009

GO:0005099~Ras GTPase activator activity	6	0.54%	0.201724

Median: 0.10189181414834529	Geo: 0.10949812218825965		
Term	Count	%	PValue
GO:0019725~cellular homeostasis	29	2.59%	0.014671
GO:0042592~homeostatic process	36	3.22%	0.02805
GO:0006875~cellular metal ion homeostasis	14	1.25%	0.088302
GO:0055065~metal ion homeostasis	14	1.25%	0.088302
GO:0006873~cellular ion homeostasis	20	1.79%	0.091552
GO:0055082~cellular chemical homeostasis	20	1.79%	0.091552
GO:0006874~cellular calcium ion homeostasi	13	1.16%	0.093013
GO:0055074~calcium ion homeostasis	13	1.16%	0.093013
GO:0065008~regulation of biological quality	59	5.28%	0.101892
GO:0050801~ion homeostasis	21	1.88%	0.117543
GO:0030003~cellular cation homeostasis	17	1.52%	0.128115
GO:0055080~cation homeostasis	17	1.52%	0.128115
GO:0048878~chemical homeostasis	22	1.97%	0.204232
GO:0030005~cellular di-, tri-valent inorganic	14	1.25%	0.219321
GO:0055066~di-, tri-valent inorganic cation h	14	1.25%	0.219321
GO:0007204~elevation of cytosolic calcium ic	7	0.63%	0.307615
GO:0051480~cytosolic calcium ion homeosta:	7	0.63%	0.338314

Median: 0.10842151729013733	Geo: 0.11186370049581089		
Term	Count	%	PValue
GO:0032012~regulation of ARF protein signal	3	0.27%	0.108422
GO:0032011~ARF protein signal transduction	3	0.27%	0.108422
GO:0005086~ARF guanyl-nucleotide exchang	3	0.27%	0.119079

Median: 0.1076855020720717	Geo: 0.11547631425780384		
Term	Count	%	PValue
rno00510:N-Glycan biosynthesis	8	0.72%	0.013869
GO:0009100~glycoprotein metabolic process	13	1.16%	0.054018
rno01030:Glycan structures - biosynthesis 1	12	1.07%	0.088684
GO:0016757~transferase activity, transferrin	17	1.52%	0.098527
GO:0006486~protein amino acid glycosylatio	10	0.89%	0.107686
GO:0043413~biopolymer glycosylation	10	0.89%	0.118084
GO:0009101~glycoprotein biosynthetic proce	10	0.89%	0.152395
GO:0016758~transferase activity, transferrin	10	0.89%	0.369148
glycosyltransferase	7	0.63%	0.779679

Median: 0.1258641009200506	Geo: 0.11806714660258627		
Term	Count	%	PValue
GO:0030424~axon	10	0.89%	0.076941
GO:0043005~neuron projection	18	1.61%	0.125864
GO:0043025~cell soma	10	0.89%	0.169952

Median: 0.1677959048829996	Geo: 0.11965887011097136		
Term	Count	%	PValue

GO:0007010~cytoskeleton organization and b	37	3.31%	0.029817
GO:0007015~actin filament organization	7	0.63%	0.092817
GO:0030036~actin cytoskeleton organization	14	1.25%	0.242775
GO:0030029~actin filament-based process	14	1.25%	0.305131

Median: 0.18185380918816363 Geo: 0.12058492068067901

Term	Count	%	PValue
GO:0008652~amino acid biosynthetic process	7	0.63%	0.045807
GO:0009064~glutamine family amino acid me	5	0.45%	0.181854
GO:0009084~glutamine family amino acid bio	3	0.27%	0.210485

Median: 0.13346020508450196 Geo: 0.1210625157076649

Term	Count	%	PValue
GO:0051246~regulation of protein metabolic	22	1.97%	0.040026
GO:0022618~protein-RNA complex assembly	10	0.89%	0.053362
GO:0009889~regulation of biosynthetic proce	16	1.43%	0.060281
GO:0006413~translational initiation	7	0.63%	0.092817
GO:0008135~translation factor activity, nucle	9	0.81%	0.104059
GO:0003743~translation initiation factor activ	6	0.54%	0.129377
GO:0006446~regulation of translational initia	5	0.45%	0.137543
GO:0022613~ribonucleoprotein complex biog	12	1.07%	0.150133
GO:0045182~translation regulator activity	9	0.81%	0.175896
GO:0031326~regulation of cellular biosynthet	12	1.07%	0.207834
initiation factor	4	0.36%	0.277076
GO:0006417~regulation of translation	10	0.89%	0.294521

Median: 0.1803019793844538 Geo: 0.12215092015596596

Term	Count	%	PValue
GO:0051301~cell division	16	1.43%	0.002944
GO:0000278~mitotic cell cycle	23	2.06%	0.017947
GO:0022403~cell cycle phase	22	1.97%	0.080816
cell cycle	15	1.34%	0.121315
cell division	9	0.81%	0.125236
GO:0051329~interphase of mitotic cell cycle	8	0.72%	0.172594
GO:0051325~interphase	8	0.72%	0.180302
GO:0051318~G1 phase	3	0.27%	0.210485
GO:0000279~M phase	15	1.34%	0.249235
GO:0007067~mitosis	11	0.98%	0.302128
mitosis	5	0.45%	0.302678
GO:0000087~M phase of mitotic cell cycle	11	0.98%	0.332362
GO:0000082~G1/S transition of mitotic cell cy	4	0.36%	0.418699

Median: 0.11267688765907659 Geo: 0.1268010744333647

Term	Count	%	PValue
GO:0035258~steroid hormone receptor bindi	5	0.45%	0.025487
GO:0030518~steroid hormone receptor signa	6	0.54%	0.047282
GO:0050681~androgen receptor binding	4	0.36%	0.053969
GO:0003713~transcription coactivator activity	14	1.25%	0.060449

GO:0030522~intracellular receptor-mediated	6	0.54%	0.097158
GO:0030521~androgen receptor signaling pa	4	0.36%	0.128196
GO:0035257~nuclear hormone receptor bindi	6	0.54%	0.211598
GO:0051427~hormone receptor binding	6	0.54%	0.242086
GO:0046966~thyroid hormone receptor bindi	3	0.27%	0.610061
GO:0045944~positive regulation of transcript	10	0.89%	0.702208

Median: 0.10065090511468879 Geo: 0.1276364001100667

Term	Count	%	PValue
pyridoxal phosphate	7	0.63%	0.047012
GO:0008483~transaminase activity	5	0.45%	0.051374
aminotransferase	4	0.36%	0.076229
GO:0016769~transferase activity, transferrin	5	0.45%	0.095855
IPR015421:Pyridoxal phosphate-dependent tr	5	0.45%	0.100651
GO:0019842~vitamin binding	10	0.89%	0.155451
GO:0030170~pyridoxal phosphate binding	5	0.45%	0.256409
binding site:Pyridoxal phosphate (covalent)	4	0.36%	0.258983
GO:0048037~cofactor binding	14	1.25%	0.490306

Median: 0.09004221777709542 Geo: 0.13237772337733444

Term	Count	%	PValue
GO:0048583~regulation of response to stimul	9	0.81%	0.01677
GO:0032107~regulation of response to nutrie	3	0.27%	0.072716
GO:0032104~regulation of response to extrac	3	0.27%	0.072716
GO:0032095~regulation of response to food	3	0.27%	0.072716
GO:0032101~regulation of response to exter	3	0.27%	0.090042
GO:0032094~response to food	3	0.27%	0.127677
GO:0031667~response to nutrient levels	8	0.72%	0.507172
GO:0009991~response to extracellular stimul	8	0.72%	0.552089
GO:0007584~response to nutrient	6	0.54%	0.601449

Median: 0.24146442504751328 Geo: 0.1327707699357756

Term	Count	%	PValue
GO:0032502~developmental process	184	16.46%	0.015368
GO:0048869~cellular developmental process	110	9.84%	0.021203
GO:0030154~cell differentiation	110	9.84%	0.021203
GO:0007275~multicellular organismal develo	131	11.72%	0.100626
GO:0048856~anatomical structure developm	118	10.55%	0.382303
GO:0048731~system development	101	9.03%	0.421206
GO:0048513~organ development	65	5.81%	0.862571
GO:0032501~multicellular organismal proces	185	16.55%	0.999998

Median: 0.17519858930582505 Geo: 0.1329722840960057

Term	Count	%	PValue
GO:0006626~protein targeting to mitochondr	5	0.45%	0.017079
GO:0006839~mitochondrial transport	6	0.54%	0.082855
GO:0007005~mitochondrion organization and	7	0.63%	0.267542
GO:0044455~mitochondrial membrane part	3	0.27%	0.825805

Median: 0.10305094387637022 Geo: 0.1330202062600308

Term	Count	%	PValue
GO:0001937~negative regulation of endothel	3	0.27%	0.090042
GO:0001935~endothelial cell proliferation	4	0.36%	0.103051
GO:0001936~regulation of endothelial cell pr	3	0.27%	0.253662

Median: 0.11990710670990666 Geo: 0.13685778237651414

Term	Count	%	PValue
GO:0007187~G-protein signaling, coupled to	14	1.25%	0.031366
GO:0019935~cyclic-nucleotide-mediated sign	14	1.25%	0.065869
GO:0007188~G-protein signaling, coupled to	11	0.98%	0.066354
GO:0045761~regulation of adenylate cyclase	6	0.54%	0.097158
GO:0051339~regulation of lyase activity	6	0.54%	0.11262
GO:0031279~regulation of cyclase activity	6	0.54%	0.11262
GO:0019933~cAMP-mediated signaling	11	0.98%	0.127194
GO:0007190~adenylate cyclase activation	4	0.36%	0.213759
GO:0051349~positive regulation of lyase acti	4	0.36%	0.244569
GO:0045762~positive regulation of adenylate	4	0.36%	0.244569
GO:0031281~positive regulation of cyclase ac	4	0.36%	0.260231
GO:0007189~G-protein signaling, adenylate c	4	0.36%	0.603889

Median: 0.0908459699550602 Geo: 0.13704169444018185

Term	Count	%	PValue
IPR003958:Transcription factor CBF/NF-Y/arch	3	0.27%	0.035936
IPR009072:Histone-fold	4	0.36%	0.090846
Transcription	3	0.27%	0.788366

Median: 0.13734646667265776 Geo: 0.14002507969953631

Term	Count	%	PValue
GO:0005798~Golgi-associated vesicle	8	0.72%	0.034523
GO:0012506~vesicle membrane	11	0.98%	0.037788
IPR008152:Clathrin adaptor, alpha/beta/gamn	3	0.27%	0.051649
SM00809:Alpha_adaptinC2	3	0.27%	0.053424
GO:0030659~cytoplasmic vesicle membrane	8	0.72%	0.092881
GO:0030133~transport vesicle	6	0.54%	0.095371
GO:0044433~cytoplasmic vesicle part	9	0.81%	0.109754
GO:0048475~coated membrane	6	0.54%	0.134454
GO:0030117~membrane coat	6	0.54%	0.134454
GO:0030118~clathrin coat	5	0.45%	0.137346
GO:0030140~trans-Golgi network transport ve	4	0.36%	0.159338
GO:0030130~clathrin coat of trans-Golgi netw	3	0.27%	0.201342
GO:0030120~vesicle coat	5	0.45%	0.239078
GO:0012510~trans-Golgi network transport ve	3	0.27%	0.243116
GO:0030125~clathrin vesicle coat	4	0.36%	0.276901
GO:0030662~coated vesicle membrane	5	0.45%	0.288866
GO:0030665~clathrin coated vesicle membra	4	0.36%	0.307779
GO:0030658~transport vesicle membrane	3	0.27%	0.368185

GO:0030660~Golgi-associated vesicle membr	3	0.27%	0.408313

Median: 0.1361326064824238 Geo: 0.1440097981822845

Term	Count	%	PValue
GO:0002761~regulation of myeloid leukocyte	6	0.54%	0.008587
GO:0002762~negative regulation of myeloid l	4	0.36%	0.016525
GO:0045637~regulation of myeloid cell differe	7	0.63%	0.024981
GO:0002573~myeloid leukocyte differentiatio	6	0.54%	0.063669
GO:0045638~negative regulation of myeloid c	4	0.36%	0.069637
GO:0045670~regulation of osteoclast differer	4	0.36%	0.069637
GO:0030316~osteoclast differentiation	4	0.36%	0.091299
GO:0048534~hemopoietic or lymphoid organ	17	1.52%	0.093025
GO:0030097~hemopoiesis	16	1.43%	0.098623
GO:0002520~immune system development	17	1.52%	0.116874
GO:0051093~negative regulation of developr	9	0.81%	0.136133
GO:0030099~myeloid cell differentiation	9	0.81%	0.155467
GO:0045639~positive regulation of myeloid c	3	0.27%	0.253662
GO:0050793~regulation of developmental prc	20	1.79%	0.270145
GO:0051094~positive regulation of developm	8	0.72%	0.319107
GO:0045596~negative regulation of cell differ	6	0.54%	0.33732
GO:0002521~leukocyte differentiation	8	0.72%	0.385125
GO:0045595~regulation of cell differentiation	12	1.07%	0.477352
GO:0045597~positive regulation of cell differe	4	0.36%	0.754627
GO:0045165~cell fate commitment	3	0.27%	0.955029
GO:0002376~immune system process	33	2.95%	0.956468

Median: 0.3508793236375377 Geo: 0.14658367233857106

Term	Count	%	PValue
GO:0048489~synaptic vesicle transport	9	0.81%	0.004764
GO:0001505~regulation of neurotransmitter l	13	1.16%	0.089241
GO:0016079~synaptic vesicle exocytosis	4	0.36%	0.16939
GO:0003001~generation of a signal involved	10	0.89%	0.350879
GO:0045055~regulated secretory pathway	8	0.72%	0.356681
GO:0007269~neurotransmitter secretion	7	0.63%	0.390062
GO:0006887~exocytosis	8	0.72%	0.413622

Median: 0.43415643114745134 Geo: 0.14675904481537738

Term	Count	%	PValue
GO:0048583~regulation of response to stimul	9	0.81%	0.01677
GO:0031347~regulation of defense response	4	0.36%	0.434156
GO:0050727~regulation of inflammatory resp	4	0.36%	0.434156

Median: 0.24998858310372712 Geo: 0.15290988756460436

Term	Count	%	PValue
wd repeat	13	1.16%	0.025128
IPR001680:WD40 repeat	13	1.16%	0.035564
SM00320:WD40	13	1.16%	0.053411
repeat:WD 6	5	0.45%	0.170135

repeat:WD 5	5	0.45%	0.242372
repeat:WD 4	5	0.45%	0.257606
repeat:WD 3	5	0.45%	0.288595
repeat:WD 2	5	0.45%	0.304286
repeat:WD 1	5	0.45%	0.304286
repeat:WD 7	3	0.27%	0.5158

Median: 0.15565765283845925 Geo: 0.1529854602605251

Term	Count	%	PValue
GO:0051186~cofactor metabolic process	20	1.79%	0.055485
GO:0006732~coenzyme metabolic process	17	1.52%	0.067256
GO:0051188~cofactor biosynthetic process	9	0.81%	0.244059
GO:0009108~coenzyme biosynthetic process	6	0.54%	0.601449

Median: 0.15899314901573366 Geo: 0.15637827340600607

Term	Count	%	PValue
GO:0000302~response to reactive oxygen sp	6	0.54%	0.069757
GO:0009725~response to hormone stimulus	15	1.34%	0.083759
GO:0043627~response to estrogen stimulus	6	0.54%	0.146779
GO:0006979~response to oxidative stress	10	0.89%	0.171207
GO:0042542~response to hydrogen peroxide	4	0.36%	0.183882
GO:0048545~response to steroid hormone sti	7	0.63%	0.541646

Median: 0.3508793236375377 Geo: 0.1679204348599385

Term	Count	%	PValue
GO:0065009~regulation of a molecular functi	40	3.58%	0.01017
GO:0050790~regulation of catalytic activity	36	3.22%	0.012368
GO:0043085~positive regulation of catalytic a	22	1.97%	0.029565
GO:0043549~regulation of kinase activity	17	1.52%	0.156675
GO:0051338~regulation of transferase activit	17	1.52%	0.188353
GO:0045859~regulation of protein kinase acti	16	1.43%	0.201339
GO:0033674~positive regulation of kinase act	10	0.89%	0.350879
GO:0051347~positive regulation of transferas	10	0.89%	0.392012
GO:0007200~G-protein signaling, coupled to	7	0.63%	0.400431
GO:0032147~activation of protein kinase acti	5	0.45%	0.438703
GO:0045860~positive regulation of protein ki	9	0.81%	0.459439
GO:0007205~protein kinase C activation	3	0.27%	0.553633
GO:0000165~MAPKKK cascade	10	0.89%	0.621474

Median: 0.200554335921077 Geo: 0.16822064139055293

Term	Count	%	PValue
PIRSF001741:membrane-associated guanylate	3	0.27%	0.023567
SM00072:GuKc	5	0.45%	0.026247
GO:0008022~protein C-terminus binding	8	0.72%	0.029859
domain:Guanylate kinase-like	3	0.27%	0.057758
domain:SH3	7	0.63%	0.075448
IPR008145:Guanylate kinase/L-type calcium c	4	0.36%	0.07839
domain:PDZ 3	3	0.27%	0.121188

GO:0030165~PDZ domain binding	6	0.54%	0.17311
IPR008144:Guanylate kinase	3	0.27%	0.200554
domain:PDZ 1	3	0.27%	0.29823
domain:PDZ 2	3	0.27%	0.29823
IPR000108:Neutrophil cytosol factor 2	3	0.27%	0.345817
IPR001452:Src homology-3	9	0.81%	0.397693
SM00326:SH3	9	0.81%	0.46892
SH3 domain	8	0.72%	0.47937
IPR001478:PDZ/DHR/GLGF	3	0.27%	0.971386
SM00228:PDZ	3	0.27%	0.975655

Median: 0.10305094387637022 Geo: 0.1746413970914796

Term	Count	%	PValue
GO:0010003~gastrulation (sensu Mammalia)	4	0.36%	0.042285
GO:0001702~gastrulation with mouth forming	4	0.36%	0.103051
GO:0048276~gastrulation (sensu Vertebrata)	4	0.36%	0.103051
GO:0007369~gastrulation	4	0.36%	0.479537
GO:0003002~regionalization	6	0.54%	0.754437

Median: 0.146778845974888 Geo: 0.175730085063464

Term	Count	%	PValue
GO:0014070~response to organic cyclic subst	6	0.54%	0.076153
GO:0043627~response to estrogen stimulus	6	0.54%	0.146779
GO:0042493~response to drug	9	0.81%	0.485498

Median: 0.2310913261201935 Geo: 0.17766634724831248

Term	Count	%	PValue
GO:0008270~zinc ion binding	90	8.05%	0.027333
zinc-finger	34	3.04%	0.155905
zinc	45	4.03%	0.306277
metal-binding	60	5.37%	0.763395

Median: 0.21535379537979749 Geo: 0.17768583122794948

Term	Count	%	PValue
GO:0016323~basolateral plasma membrane	13	1.16%	0.010827
GO:0005912~adherens junction	9	0.81%	0.055736
GO:0005925~focal adhesion	5	0.45%	0.158264
GO:0005924~cell-substrate adherens junction	5	0.45%	0.191629
GO:0030055~cell-matrix junction	5	0.45%	0.239078
GO:0005913~cell-cell adherens junction	3	0.27%	0.447158
GO:0030054~cell junction	11	0.98%	0.58202
GO:0005911~intercellular junction	6	0.54%	0.872536

Median: 0.15546695149387 Geo: 0.17829203345072625

Term	Count	%	PValue
GO:0005184~neuropeptide hormone activity	5	0.45%	0.057794
GO:0007218~neuropeptide signaling pathway	9	0.81%	0.155467
GO:0005179~hormone activity	8	0.72%	0.630778

Median: 0.1719070097875019	Geo: 0.17861075696769138		
Term	Count	%	PValue
serine/threonine-specific protein kinase	6	0.54%	0.054413
PIRSF000564:kinase-related transforming prot	4	0.36%	0.171907
rno04340:Hedgehog signaling pathway	4	0.36%	0.609158

Median: 0.48112562038876727	Geo: 0.1787057436525346		
Term	Count	%	PValue
GO:0044456~synapse part	19	1.70%	2.22E-04
GO:0045202~synapse	27	2.42%	3.62E-04
GO:0045211~postsynaptic membrane	12	1.07%	0.018502
GO:0008144~drug binding	4	0.36%	0.240467
synapse	13	1.16%	0.283011
Postsynaptic cell membrane	8	0.72%	0.384888
GO:0030534~adult behavior	4	0.36%	0.403104
neurotransmitter receptor	5	0.45%	0.462802
GO:0005230~extracellular ligand-gated ion cl	5	0.45%	0.481126
GO:0030594~neurotransmitter receptor activ	7	0.63%	0.565097
cell junction	14	1.25%	0.609355
GO:0042165~neurotransmitter binding	7	0.63%	0.642168
IPR006202:Neurotransmitter-gated ion-chann	3	0.27%	0.748186
IPR006029:Neurotransmitter-gated ion-chann	3	0.27%	0.748186
IPR006201:Neurotransmitter-gated ion-chann	3	0.27%	0.748186
GO:0015276~ligand-gated ion channel activit	5	0.45%	0.772342
GO:0022834~ligand-gated channel activity	5	0.45%	0.772342

Median: 0.1803019793844538	Geo: 0.18582633481789906		
Term	Count	%	PValue
GO:0007270~nerve-nerve synaptic transmiss	6	0.54%	0.097158
GO:0007612~learning	5	0.45%	0.107637
GO:0048169~regulation of long-term neurona	3	0.27%	0.147649
GO:0007611~learning and/or memory	8	0.72%	0.180302
GO:0048167~regulation of synaptic plasticity	6	0.54%	0.194764
GO:0050803~regulation of synapse structure	6	0.54%	0.247342
GO:0048168~regulation of neuronal synaptic	3	0.27%	0.57052

Median: 0.21008051367698122	Geo: 0.18658182674770896		
Term	Count	%	PValue
GO:0006605~protein targeting	20	1.79%	0.046078
GO:0017038~protein import	9	0.81%	0.142436
GO:0006913~nucleocytoplasmic transport	11	0.98%	0.184392
GO:0051169~nuclear transport	11	0.98%	0.210081
GO:0006606~protein import into nucleus	7	0.63%	0.267542
GO:0051170~nuclear import	7	0.63%	0.287429
GO:0000060~protein import into nucleus, trai	3	0.27%	0.402649

Median: 0.19868478281630433	Geo: 0.1885086380593916

Term	Count	%	PValue
GO:0006665~sphingolipid metabolic process	6	0.54%	0.137856
GO:0006672~ceramide metabolic process	4	0.36%	0.198685
GO:0046519~sphingoid metabolic process	4	0.36%	0.244569

Median: 0.22585340278900806 Geo: 0.18852812202274055

Term	Count	%	PValue
GO:0015833~peptide transport	8	0.72%	0.039498
GO:0046883~regulation of hormone secretior	5	0.45%	0.089544
GO:0002790~peptide secretion	5	0.45%	0.107637
GO:0046888~negative regulation of hormone	3	0.27%	0.189177
GO:0030073~insulin secretion	4	0.36%	0.198685
GO:0046879~hormone secretion	6	0.54%	0.225853
GO:0050796~regulation of insulin secretion	3	0.27%	0.232012
GO:0030072~peptide hormone secretion	4	0.36%	0.260231
GO:0003001~generation of a signal involved	10	0.89%	0.350879
GO:0051046~regulation of secretion	7	0.63%	0.369321
GO:0051048~negative regulation of secretior	3	0.27%	0.422933

Median: 0.20076639204212415 Geo: 0.19510207483180358

Term	Count	%	PValue
IPR001841:Zinc finger, RING-type	11	0.98%	0.152621
SM00184:RING	11	0.98%	0.200766
zinc finger region:RING-type	5	0.45%	0.242372

Median: 0.17259375798038148 Geo: 0.19665995517085125

Term	Count	%	PValue
GO:0040011~locomotion	9	0.81%	0.096192
GO:0030334~regulation of cell migration	7	0.63%	0.1582
GO:0040013~negative regulation of locomoti	4	0.36%	0.16939
GO:0040012~regulation of locomotion	8	0.72%	0.172594
GO:0051270~regulation of cell motility	7	0.63%	0.238419
GO:0030336~negative regulation of cell migr	3	0.27%	0.297004
GO:0051271~negative regulation of cell motil	3	0.27%	0.361117

Median: 0.1927075177175326 Geo: 0.19770529455139862

Term	Count	%	PValue
GO:0009056~catabolic process	46	4.11%	0.060783
GO:0044248~cellular catabolic process	39	3.49%	0.068095
GO:0030163~protein catabolic process	16	1.43%	0.12049
GO:0006511~ubiquitin-dependent protein cat	12	1.07%	0.134647
GO:0043632~modification-dependent macror	12	1.07%	0.144872
GO:0019941~modification-dependent protein	12	1.07%	0.144872
GO:0051603~proteolysis involved in cellular p	12	1.07%	0.166495
GO:0044257~cellular protein catabolic proces	12	1.07%	0.183691
GO:0004843~ubiquitin-specific protease activ	6	0.54%	0.201724
GO:0019783~small conjugating protein-speci	6	0.54%	0.211598
GO:0016790~thiolester hydrolase activity	7	0.63%	0.228439

GO:0004221~ubiquitin thiolesterase activity	5	0.45%	0.318674
GO:0043285~biopolymer catabolic process	16	1.43%	0.421516
GO:0044265~cellular macromolecule cataboli	19	1.70%	0.425693
GO:0009057~macromolecule catabolic proce:	23	2.06%	0.437564
GO:0008234~cysteine-type peptidase activity	10	0.89%	0.518148

Median: 0.3402131629373112 Geo: 0.20091348973280643

Term	Count	%	PValue
GO:0009058~biosynthetic process	98	8.77%	0.01976
GO:0044249~cellular biosynthetic process	67	5.99%	0.249013
GO:0009059~macromolecule biosynthetic pro	50	4.47%	0.431413
GO:0006412~translation	31	2.77%	0.767586

Median: 0.15219034199850132 Geo: 0.20388043471441814

Term	Count	%	PValue
GO:0009892~negative regulation of metaboli	28	2.50%	0.082998
GO:0000122~negative regulation of transcrip	12	1.07%	0.106427
GO:0016481~negative regulation of transcrip	20	1.79%	0.108749
GO:0045892~negative regulation of transcrip	15	1.34%	0.145618
GO:0031324~negative regulation of cellular r	24	2.15%	0.15219
GO:0045934~negative regulation of nucleoba	20	1.79%	0.171513
GO:0016564~transcription repressor activity	14	1.25%	0.250078
GO:0006357~regulation of transcription from	23	2.06%	0.714292
GO:0006366~transcription from RNA polymer	25	2.24%	0.933218

Median: 0.20425774245182154 Geo: 0.2051856545880777

Term	Count	%	PValue
GO:0043549~regulation of kinase activity	17	1.52%	0.156675
GO:0051338~regulation of transferase activit	17	1.52%	0.188353
GO:0045859~regulation of protein kinase acti	16	1.43%	0.201339
GO:0043086~negative regulation of catalytic	8	0.72%	0.204258
GO:0006469~negative regulation of protein k	6	0.54%	0.225853
GO:0033673~negative regulation of kinase ac	6	0.54%	0.225853
GO:0051348~negative regulation of transfera	6	0.54%	0.247342

Median: 0.24894442110152143 Geo: 0.21358808151445924

Term	Count	%	PValue
IPR013753:Ras	9	0.81%	0.152954
IPR003578:Ras small GTPase, Rho type	3	0.27%	0.248944
SM00174:RHO	3	0.27%	0.255899

Median: 0.23201158734523689 Geo: 0.21679040728369364

Term	Count	%	PValue
GO:0006665~sphingolipid metabolic process	6	0.54%	0.137856
GO:0006687~glycosphingolipid metabolic pro	3	0.27%	0.232012
GO:0006664~glycolipid metabolic process	3	0.27%	0.318554

Median: 0.2103440663828989 Geo: 0.21685062964976048

Term	Count	%	PValue
GO:0050804~regulation of synaptic transmiss	6	0.54%	0.204959
GO:0031644~regulation of neurological proce	7	0.63%	0.210344
GO:0051969~regulation of transmission of ne	6	0.54%	0.236529

Median: 0.23477854425515898 Geo: 0.22626350310823068

Term	Count	%	PValue
GO:0016477~cell migration	21	1.88%	0.210149
GO:0051674~localization of cell	27	2.42%	0.234779
GO:0006928~cell motility	27	2.42%	0.234779

Median: 0.213759410366611 Geo: 0.22973313871178894

Term	Count	%	PValue
GO:0006099~tricarboxylic acid cycle	4	0.36%	0.155251
GO:0046356~acetyl-CoA catabolic process	4	0.36%	0.16939
GO:0006084~acetyl-CoA metabolic process	5	0.45%	0.193598
GO:0009109~coenzyme catabolic process	4	0.36%	0.213759
GO:0009060~aerobic respiration	4	0.36%	0.260231
GO:0051187~cofactor catabolic process	4	0.36%	0.307828
GO:0045333~cellular respiration	4	0.36%	0.387391

Median: 0.44053360278966125 Geo: 0.22979315384796073

Term	Count	%	PValue
GO:0060090~molecular adaptor activity	6	0.54%	0.04997
GO:0005070~SH3/SH2 adaptor activity	3	0.27%	0.440534
GO:0030674~protein binding, bridging	5	0.45%	0.551213

Median: 0.3185542039557815 Geo: 0.2348694799133672

Term	Count	%	PValue
GO:0045428~regulation of nitric oxide biosyn	3	0.27%	0.127677
GO:0006809~nitric oxide biosynthetic process	3	0.27%	0.318554
GO:0046209~nitric oxide metabolic process	3	0.27%	0.318554

Median: 0.2640537134097305 Geo: 0.24588531886167345

Term	Count	%	PValue
GO:0016868~intramolecular transferase activ	3	0.27%	0.100971
GO:0016866~intramolecular transferase activ	4	0.36%	0.182498
GO:0016853~isomerase activity	9	0.81%	0.345609
Carbohydrate transport and metabolism	7	0.63%	0.573973

Median: 0.21316646918908094 Geo: 0.24597617148673978

Term	Count	%	PValue
GO:0004468~lysine N-acetyltransferase activ	3	0.27%	0.137908
GO:0004402~histone acetyltransferase activi	3	0.27%	0.137908
GO:0016746~transferase activity, transferrin	12	1.07%	0.162026
GO:0008415~acyltransferase activity	11	0.98%	0.206846
GO:0016747~transferase activity, transferrin	11	0.98%	0.213166
GO:0016410~N-acyltransferase activity	5	0.45%	0.244245

GO:0008080~N-acetyltransferase activity	4	0.36%	0.362031
acyltransferase	6	0.54%	0.457428
GO:0016407~acetyltransferase activity	4	0.36%	0.599807

Median: 0.3846404082940681 Geo: 0.24846768359367194

Term	Count	%	PValue
GO:0006643~membrane lipid metabolic proc	14	1.25%	0.099218
GO:0044255~cellular lipid metabolic process	33	2.95%	0.38464
GO:0006629~lipid metabolic process	39	3.49%	0.401942

Median: 0.34773517538872534 Geo: 0.24956754803083364

Term	Count	%	PValue
rno04662:B cell receptor signaling pathway	9	0.81%	0.050096
rno04360:Axon guidance	11	0.98%	0.220624
rno04650:Natural killer cell mediated cytotoxi	9	0.81%	0.288715
rno04210:Apoptosis	7	0.63%	0.406755
rno04660:T cell receptor signaling pathway	8	0.72%	0.42779
rno04370:VEGF signaling pathway	6	0.54%	0.435149

Median: 0.32482771592771276 Geo: 0.24979656724149604

Term	Count	%	PValue
GO:0035258~steroid hormone receptor bindi	5	0.45%	0.025487
GO:0003713~transcription coactivator activit	14	1.25%	0.060449
GO:0048518~positive regulation of biological	70	6.26%	0.121497
activator	14	1.25%	0.123257
GO:0008134~transcription factor binding	26	2.33%	0.124756
GO:0003712~transcription cofactor activity	19	1.70%	0.14302
GO:0009893~positive regulation of metabolic	28	2.50%	0.201809
GO:0016563~transcription activator activity	19	1.70%	0.273589
GO:0031325~positive regulation of cellular m	24	2.15%	0.376066
GO:0048522~positive regulation of cellular pr	58	5.19%	0.380492
GO:0045935~positive regulation of nucleobas	19	1.70%	0.487533
GO:0045893~positive regulation of transcript	15	1.34%	0.509516
GO:0045941~positive regulation of transcript	17	1.52%	0.607714
GO:0045944~positive regulation of transcript	10	0.89%	0.702208
GO:0006357~regulation of transcription from	23	2.06%	0.714292
GO:0006366~transcription from RNA polymer	25	2.24%	0.933218

Median: 0.261572969683048 Geo: 0.2505067926553862

Term	Count	%	PValue
GO:0005778~peroxisomal membrane	4	0.36%	0.187301
GO:0031903~microbody membrane	4	0.36%	0.187301
GO:0044439~peroxisomal part	4	0.36%	0.261573
GO:0044438~microbody part	4	0.36%	0.261573
GO:0005777~peroxisome	7	0.63%	0.28454
GO:0042579~microbody	7	0.63%	0.28454
GO:0007031~peroxisome organization and bi	3	0.27%	0.318554

Median: 0.18151085579993848	Geo: 0.25534676871162876		
Term	Count	%	PValue
GO:0015630~microtubule cytoskeleton	22	1.97%	0.125925
GO:0007017~microtubule-based process	16	1.43%	0.181511
GO:0005874~microtubule	8	0.72%	0.728409

Median: 0.36267773691844213	Geo: 0.25565972459880804		
Term	Count	%	PValue
GO:0006643~membrane lipid metabolic proc	14	1.25%	0.099218
GO:0006644~phospholipid metabolic process	9	0.81%	0.362678
GO:0006650~glycerophospholipid metabolic p	5	0.45%	0.464381

Median: 0.25291158153882726	Geo: 0.2570069622144935		
Term	Count	%	PValue
GO:0001565~phorbol ester receptor activity	3	0.27%	0.137908
IPR002219:Protein kinase C, phorbol ester/dia	5	0.45%	0.237707
zinc finger region:Phorbol-ester/DAG-type	3	0.27%	0.246277
Phorbol-ester binding	4	0.36%	0.259546
SM00109:C1	5	0.45%	0.277455
GO:0019992~diacylglycerol binding	3	0.27%	0.495685

Median: 0.2646648643314025	Geo: 0.257178985179388		
Term	Count	%	PValue
GO:0031981~nuclear lumen	31	2.77%	0.137206
GO:0043233~organelle lumen	38	3.40%	0.201213
GO:0031974~membrane-enclosed lumen	38	3.40%	0.201213
GO:0044428~nuclear part	45	4.03%	0.264665
GO:0005667~transcription factor complex	13	1.16%	0.312198
GO:0005654~nucleoplasm	22	1.97%	0.320442
GO:0044451~nucleoplasm part	17	1.52%	0.505927

Median: 0.23201158734523689	Geo: 0.2583443959260503		
Term	Count	%	PValue
GO:0006903~vesicle targeting	3	0.27%	0.147649
GO:0051650~establishment of vesicle localiza	3	0.27%	0.210485
GO:0051648~vesicle localization	3	0.27%	0.232012
GO:0051640~organelle localization	4	0.36%	0.307828
GO:0051656~establishment of organelle loca	3	0.27%	0.518472

Median: 0.25368116831589566	Geo: 0.25999331869230846		
Term	Count	%	PValue
GO:0042745~circadian sleep/wake cycle	3	0.27%	0.189177
GO:0048512~circadian behavior	3	0.27%	0.210485
GO:0030431~sleep	3	0.27%	0.232012
GO:0007622~rhythmic behavior	3	0.27%	0.275351
GO:0048511~rhythmic process	9	0.81%	0.276768
GO:0007623~circadian rhythm	5	0.45%	0.438703

Median: 0.43323106073215817	Geo: 0.26210156016547564		
Term	Count	%	PValue
GO:0030031~cell projection biogenesis	8	0.72%	0.018509
GO:0048699~generation of neurons	27	2.42%	0.093349
GO:0022008~neurogenesis	29	2.59%	0.105274
GO:0048858~cell projection morphogenesis	20	1.79%	0.129073
GO:0030030~cell projection organization and	20	1.79%	0.129073
GO:0032990~cell part morphogenesis	20	1.79%	0.129073
GO:0030182~neuron differentiation	20	1.79%	0.370018
GO:0048666~neuron development	15	1.34%	0.405516
GO:0031175~neurite development	13	1.16%	0.460946
GO:0000902~cell morphogenesis	28	2.50%	0.512472
GO:0032989~cellular structure morphogenes	28	2.50%	0.512472
GO:0000904~cellular morphogenesis during c	12	1.07%	0.520972
GO:0048812~neurite morphogenesis	11	0.98%	0.525559
GO:0048667~neuron morphogenesis during c	11	0.98%	0.525559
GO:0009653~anatomical structure morphoge	59	5.28%	0.618174
GO:0007409~axonogenesis	8	0.72%	0.784897

Median: 0.2920008222498741	Geo: 0.27413750077368854		
Term	Count	%	PValue
GO:0033014~tetrapyrrole biosynthetic proces	3	0.27%	0.168192
GO:0006779~porphyrin biosynthetic process	3	0.27%	0.168192
GO:0051188~cofactor biosynthetic process	9	0.81%	0.244059
GO:0033013~tetrapyrrole metabolic process	3	0.27%	0.339943
GO:0006778~porphyrin metabolic process	3	0.27%	0.339943
GO:0046483~heterocycle metabolic process	7	0.63%	0.531976

Median: 0.2589829024123425	Geo: 0.2748545576081132		
Term	Count	%	PValue
GO:0009310~amine catabolic process	7	0.63%	0.174917
GO:0044270~nitrogen compound catabolic pr	7	0.63%	0.192318
binding site:Pyridoxal phosphate (covalent)	4	0.36%	0.258983
GO:0009063~amino acid catabolic process	5	0.45%	0.38641
rno00252:Alanine and aspartate metabolism	3	0.27%	0.465956

Median: 0.315135754469714	Geo: 0.27852397661412764		
Term	Count	%	PValue
GO:0005773~vacuole	13	1.16%	0.212609
GO:0005764~lysosome	11	0.98%	0.315136
GO:0000323~lytic vacuole	11	0.98%	0.322484

Median: 0.3837679797429484	Geo: 0.28680725809930663		
Term	Count	%	PValue
GO:0001701~in utero embryonic developmer	8	0.72%	0.09846
GO:0009790~embryonic development	21	1.88%	0.308097
GO:0043009~chordate embryonic developme	9	0.81%	0.459439

GO:0009792~embryonic development ending	9	0.81%	0.485498

Median: 0.43870341527122014 Geo: 0.29161371042556156

Term	Count	%	PValue
GO:0051241~negative regulation of multicellu	9	0.81%	7.98E-04
GO:0008016~regulation of heart contraction	5	0.45%	0.281088
GO:0003015~heart process	5	0.45%	0.373214
GO:0060047~heart contraction	5	0.45%	0.373214
GO:0035150~regulation of tube size	5	0.45%	0.412681
GO:0050880~regulation of blood vessel size	5	0.45%	0.412681
GO:0003018~vascular process in circulatory s	5	0.45%	0.438703
GO:0008217~regulation of blood pressure	6	0.54%	0.452147
GO:0042311~vasodilation	3	0.27%	0.553633
GO:0035295~tube development	11	0.98%	0.584276
GO:0035239~tube morphogenesis	8	0.72%	0.611771
GO:0008015~blood circulation	10	0.89%	0.726533
GO:0003013~circulatory system process	10	0.89%	0.726533

Median: 0.2779018162955732 Geo: 0.29279001064309174

Term	Count	%	PValue
GO:0043284~biopolymer biosynthetic process	7	0.63%	0.134529
GO:0033692~cellular polysaccharide biosynth	4	0.36%	0.213759
GO:0000271~polysaccharide biosynthetic pro	4	0.36%	0.229067
GO:0009250~glucan biosynthetic process	3	0.27%	0.232012
GO:0005978~glycogen biosynthetic process	3	0.27%	0.232012
GO:0005977~glycogen metabolic process	4	0.36%	0.323792
GO:0006073~glucan metabolic process	4	0.36%	0.339757
GO:0044264~cellular polysaccharide metabol	5	0.45%	0.412681
GO:0005976~polysaccharide metabolic proce	5	0.45%	0.42573
GO:0016051~carbohydrate biosynthetic proc	6	0.54%	0.675549

Median: 0.2934854081935494 Geo: 0.2955976854979881

Term	Count	%	PValue
GO:0051093~negative regulation of developm	9	0.81%	0.136133
GO:0050771~negative regulation of axonogen	3	0.27%	0.147649
GO:0050767~regulation of neurogenesis	6	0.54%	0.225853
GO:0050768~negative regulation of neurogen	3	0.27%	0.361117
GO:0050770~regulation of axonogenesis	3	0.27%	0.518472
GO:0007409~axonogenesis	8	0.72%	0.784897

Median: 0.3419389271765082 Geo: 0.3125283750727572

Term	Count	%	PValue
isomerase	7	0.63%	0.237841
Rotamase	3	0.27%	0.29287
GO:0003755~peptidyl-prolyl cis-trans isomera	3	0.27%	0.341939
GO:0016853~isomerase activity	9	0.81%	0.345609
GO:0016859~cis-trans isomerase activity	3	0.27%	0.362203

Median: 0.3185542039557815	Geo: 0.3141499002215701		
Term	Count	%	PValue
GO:0007043~intercellular junction assembly	3	0.27%	0.210485
GO:0045216~intercellular junction assembly a	3	0.27%	0.318554
GO:0007028~cytoplasm organization and bio	3	0.27%	0.462387

Median: 0.4768385859093126	Geo: 0.31852749595736973		
Term	Count	%	PValue
GO:0006767~water-soluble vitamin metabolic	9	0.81%	0.007896
GO:0006769~nicotinamide metabolic process	5	0.45%	0.089544
GO:0006766~vitamin metabolic process	9	0.81%	0.106836
GO:0019362~pyridine nucleotide metabolic p	5	0.45%	0.107637
GO:0044262~cellular carbohydrate metabolic	26	2.33%	0.11154
GO:0006098~pentose-phosphate shunt	3	0.27%	0.168192
GO:0006066~alcohol metabolic process	23	2.06%	0.243199
GO:0006739~NADP metabolic process	3	0.27%	0.253662
rno00010:Glycolysis / Gluconeogenesis	5	0.45%	0.360616
GO:0005975~carbohydrate metabolic process	30	2.68%	0.364572
glycolysis	4	0.36%	0.400613
GO:0016616~oxidoreductase activity, acting	7	0.63%	0.471222
GO:0006007~glucose catabolic process	7	0.63%	0.482455
GO:0006006~glucose metabolic process	10	0.89%	0.514797
GO:0046365~monosaccharide catabolic proce	7	0.63%	0.531976
GO:0019320~hexose catabolic process	7	0.63%	0.531976
GO:0046164~alcohol catabolic process	7	0.63%	0.56072
GO:0019318~hexose metabolic process	11	0.98%	0.659195
GO:0005996~monosaccharide metabolic proc	11	0.98%	0.67824
binding site:Substrate	6	0.54%	0.716583
GO:0050662~coenzyme binding	9	0.81%	0.750593
GO:0044275~cellular carbohydrate catabolic	7	0.63%	0.767266
GO:0016052~carbohydrate catabolic process	7	0.63%	0.813781
GO:0006096~glycolysis	4	0.36%	0.861085

Median: 0.32733578102708194	Geo: 0.32861072017313064		
Term	Count	%	PValue
GO:0006821~chloride transport	6	0.54%	0.16533
GO:0031404~chloride ion binding	5	0.45%	0.185787
GO:0043168~anion binding	5	0.45%	0.197106
GO:0008509~anion transmembrane transport	10	0.89%	0.276798
GO:0015698~inorganic anion transport	11	0.98%	0.294678
GO:0006820~anion transport	13	1.16%	0.327336
GO:0005253~anion channel activity	5	0.45%	0.331305
chloride channel	4	0.36%	0.400613
GO:0005254~chloride channel activity	4	0.36%	0.480429
chloride	4	0.36%	0.501672
GO:0022836~gated channel activity	11	0.98%	0.933086

Median: 0.38303656691347576 Geo: 0.3286281289729965

Term	Count	%	PValue
GO:0019198~transmembrane receptor protei	3	0.27%	0.177206
GO:0004725~protein tyrosine phosphatase ac	6	0.54%	0.383037
SM00194:PTPc	4	0.36%	0.522872

Median: 0.3137784044701102	Geo: 0.35567081697826736		
Term	Count	%	PValue
GO:0016575~histone deacetylation	3	0.27%	0.210485
GO:0004407~histone deacetylase activity	3	0.27%	0.259361
GO:0033558~protein deacetylase activity	3	0.27%	0.259361
GO:0006476~protein amino acid deacetylatio	3	0.27%	0.275351
GO:0016811~hydrolase activity, acting on car	5	0.45%	0.306082
GO:0019213~deacetylase activity	3	0.27%	0.321475
GO:0016570~histone modification	4	0.36%	0.464591
GO:0016569~covalent chromatin modificatior	4	0.36%	0.479537
GO:0016568~chromatin modification	7	0.63%	0.570116
GO:0016810~hydrolase activity, acting on car	6	0.54%	0.664852

Median: 0.37687762029993876	Geo: 0.35655060468790495		
Term	Count	%	PValue
GO:0031300~intrinsic to organelle membrane	9	0.81%	0.177475
GO:0031301~integral to organelle membrane	8	0.72%	0.216569
GO:0030173~integral to Golgi membrane	4	0.36%	0.323272
GO:0031228~intrinsic to Golgi membrane	4	0.36%	0.430483
GO:0030176~integral to endoplasmic reticulu	3	0.27%	0.570731
GO:0031227~intrinsic to endoplasmic reticulu	3	0.27%	0.673033

Median: 0.4075141240870743	Geo: 0.35866557898917073		
Term	Count	%	PValue
IPR000162:GPCR, family 3, metabotropic gluta	3	0.27%	0.08858
IPR001828:Extracellular ligand-binding recept	5	0.45%	0.310163
IPR000337:GPCR, family 3	4	0.36%	0.354689
IPR011500:GPCR, family 3, nine cysteines reg	3	0.27%	0.46034
GO:0008066~glutamate receptor activity	7	0.63%	0.608926
GO:0008067~metabotropic glutamate, GABA-	5	0.45%	0.779334

Median: 0.3606164229489288	Geo: 0.36505968111636955		
Term	Count	%	PValue
rno05210:Colorectal cancer	9	0.81%	0.144588
rno05215:Prostate cancer	9	0.81%	0.20278
rno05212:Pancreatic cancer	8	0.72%	0.209256
rno05219:Bladder cancer	5	0.45%	0.22194
rno05213:Endometrial cancer	6	0.54%	0.258366
rno04664:Fc epsilon RI signaling pathway	7	0.63%	0.308233
rno05214:Glioma	6	0.54%	0.35282
rno04930:Type II diabetes mellitus	5	0.45%	0.360616
rno04910:Insulin signaling pathway	10	0.89%	0.420725
rno04012:ErbB signaling pathway	7	0.63%	0.480269

rno05221:Acute myeloid leukemia	5	0.45%	0.499266
rno05220:Chronic myeloid leukemia	6	0.54%	0.601857
rno05223:Non-small cell lung cancer	4	0.36%	0.679289
rno04620:Toll-like receptor signaling pathway	6	0.54%	0.689423
rno05218:Melanoma	5	0.45%	0.694744

Median: 0.6107688362713029 Geo: 0.36786362508084713

Term	Count	%	PValue
GO:0043412~biopolymer modification	104	9.30%	0.010652
GO:0006464~protein modification process	101	9.03%	0.011152
GO:0043687~post-translational protein modif	81	7.25%	0.052405
serine/threonine-specific protein kinase	6	0.54%	0.054413
GO:0006796~phosphate metabolic process	58	5.19%	0.203992
GO:0006793~phosphorus metabolic process	58	5.19%	0.203992
phosphotransferase	9	0.81%	0.270275
IPR008271:Serine/threonine protein kinase, ac	14	1.25%	0.389865
atp-binding	39	3.49%	0.418874
ATP	11	0.98%	0.436506
binding site:ATP	15	1.34%	0.467178
GO:0016740~transferase activity	91	8.14%	0.503766
Serine/threonine-protein kinase	13	1.16%	0.523432
domain:Protein kinase	13	1.16%	0.606836
IPR000719:Protein kinase, core	18	1.61%	0.614701
IPR002290:Serine/threonine protein kinase	9	0.81%	0.665432
GO:0016310~phosphorylation	41	3.67%	0.697755
GO:0004713~protein-tyrosine kinase activity	15	1.34%	0.727277
GO:0004674~protein serine/threonine kinase	26	2.33%	0.739647
GO:0006468~protein amino acid phosphoryla	34	3.04%	0.74446
GO:0016301~kinase activity	43	3.85%	0.775186
GO:0016772~transferase activity, transferrin	50	4.47%	0.806929
active site:Proton acceptor	16	1.43%	0.829169
kinase	22	1.97%	0.878988
GO:0016773~phosphotransferase activity, alc	35	3.13%	0.882313
SM00220:S_TKc	16	1.43%	0.886968
nucleotide phosphate-binding region:ATP	15	1.34%	0.911234
GO:0004672~protein kinase activity	29	2.59%	0.915438

Median: 0.36474197152379556 Geo: 0.3745507585387694

Term	Count	%	PValue
domain:Helix-loop-helix motif	6	0.54%	0.103468
SM00353:HLH	7	0.63%	0.226373
DNA-binding region:Basic motif	6	0.54%	0.346947
IPR001092:Basic helix-loop-helix dimerisation	6	0.54%	0.364742
GO:0003705~RNA polymerase II transcription	3	0.27%	0.440534
IPR011598:Helix-loop-helix DNA-binding	3	0.27%	0.803061
GO:0003702~RNA polymerase II transcription	5	0.45%	0.986203

Median: 0.40335407232030307 Geo: 0.3871980889214496

Term	Count	%	PValue
GO:0009719~response to endogenous stimul	28	2.50%	0.170654
GO:0006974~response to DNA damage stimu	15	1.34%	0.366557
GO:0006281~DNA repair	12	1.07%	0.403354
GO:0006260~DNA replication	11	0.98%	0.533056
GO:0006259~DNA metabolic process	32	2.86%	0.647067

Median: 0.3945131660035479 Geo: 0.39080685877127763

Term	Count	%	PValue
IPR013766:Thioredoxin domain	3	0.27%	0.297638
GO:0045454~cell redox homeostasis	5	0.45%	0.373214
IPR006662:Thioredoxin-related	3	0.27%	0.415812
IPR012335:Thioredoxin fold	6	0.54%	0.505016

Median: 0.577788008160159 Geo: 0.40749427993885956

Term	Count	%	PValue
IPR001881:EGF-like calcium-binding	9	0.81%	0.027665
SM00179:EGF_CA	9	0.81%	0.039536
domain:EGF-like 1	6	0.54%	0.132888
IPR000152:Aspartic acid and asparagine hydr	7	0.63%	0.164861
IPR006209:EGF-like	8	0.72%	0.193811
egf-like domain	11	0.98%	0.205223
IPR000742:EGF-like, type 3	10	0.89%	0.212821
domain:EGF-like 3; calcium-binding	3	0.27%	0.220383
IPR013091:EGF calcium-binding	5	0.45%	0.251909
IPR006210:EGF	10	0.89%	0.254032
SM00181:EGF	10	0.89%	0.280013
domain:EGF-like 2	4	0.36%	0.316111
domain:EGF-like 2; calcium-binding	3	0.27%	0.423992
IPR013032:EGF-like region	11	0.98%	0.511755
rno04610:Complement and coagulation casca	5	0.45%	0.528473
serine proteinase	3	0.27%	0.627103
GO:0007596~blood coagulation	5	0.45%	0.688275
GO:0050817~coagulation	5	0.45%	0.697531
GO:0007599~hemostasis	5	0.45%	0.724107
active site:Charge relay system	5	0.45%	0.853922
serine protease	5	0.45%	0.860029
domain:Peptidase S1	3	0.27%	0.864596
IPR001314:Peptidase S1A, chymotrypsin	5	0.45%	0.871184
GO:0050878~regulation of body fluid levels	5	0.45%	0.897558
IPR001254:Peptidase S1 and S6, chymotrypsi	5	0.45%	0.903363
GO:0008236~serine-type peptidase activity	9	0.81%	0.911622
GO:0017171~serine hydrolase activity	9	0.81%	0.911622
SM00020:Tryp_SPc	5	0.45%	0.912062
GO:0004252~serine-type endopeptidase acti	7	0.63%	0.957408
protease	12	1.07%	0.976645

Median: 0.40647718696843593 Geo: 0.41412682105793264

Term	Count	%	PValue
GO:0006323~DNA packaging	16	1.43%	0.206447
IPR002164:Nucleosome assembly protein (NA	3	0.27%	0.273309
GO:0006334~nucleosome assembly	6	0.54%	0.31441
GO:0006325~establishment and/or maintenar	14	1.25%	0.371224
GO:0031497~chromatin assembly	6	0.54%	0.406477
GO:0000786~nucleosome	4	0.36%	0.489057
GO:0065004~protein-DNA complex assembly	7	0.63%	0.570116
GO:0051276~chromosome organization and b	15	1.34%	0.64874
GO:0006333~chromatin assembly or disasser	6	0.54%	0.739903

Median: 0.532431042883997 Geo: 0.4177511484099943

Term	Count	%	PValue
GO:0046851~negative regulation of bone rem	4	0.36%	0.059801
GO:0030279~negative regulation of ossificati	3	0.27%	0.127677
GO:0046850~regulation of bone remodeling	5	0.45%	0.242656
GO:0030278~regulation of ossification	4	0.36%	0.323792
GO:0048771~tissue remodeling	11	0.98%	0.51045
GO:0030282~bone mineralization	3	0.27%	0.518472
GO:0046849~bone remodeling	10	0.89%	0.54639
GO:0001503~ossification	8	0.72%	0.65935
GO:0031214~biomineral formation	8	0.72%	0.65935
GO:0001649~osteoblast differentiation	3	0.27%	0.84536
GO:0001501~skeletal development	11	0.98%	0.892239
GO:0009888~tissue development	12	1.07%	0.993113

Median: 0.4126814261238582 Geo: 0.42114604607328787

Term	Count	%	PValue
GO:0000245~spliceosome assembly	3	0.27%	0.402649
GO:0000377~RNA splicing, via transesterifica	5	0.45%	0.412681
GO:0000398~nuclear mRNA splicing, via splic	5	0.45%	0.412681
GO:0000375~RNA splicing, via transesterifica	5	0.45%	0.412681
GO:0008380~RNA splicing	9	0.81%	0.468157

Median: 0.46654621200982815 Geo: 0.42269776100474143

Term	Count	%	PValue
GO:0046058~cAMP metabolic process	4	0.36%	0.229067
GO:0006171~cAMP biosynthetic process	3	0.27%	0.382033
GO:0009187~cyclic nucleotide metabolic proc	4	0.36%	0.55106
GO:0009190~cyclic nucleotide biosynthetic p	3	0.27%	0.662001

Median: 0.9529865236334002 Geo: 0.42284236028087474

Term	Count	%	PValue
GO:0007187~G-protein signaling, coupled to c	14	1.25%	0.031366
GO:0019932~second-messenger-mediated si	24	2.15%	0.049374
GO:0019935~cyclic-nucleotide-mediated sign	14	1.25%	0.065869
GO:0007188~G-protein signaling, coupled to c	11	0.98%	0.066354
G protein-coupled receptor	11	0.98%	0.068436

GO:0019933~cAMP-mediated signaling	11	0.98%	0.127194
GO:0008227~amine receptor activity	4	0.36%	0.46613
rno04080:Neuroactive ligand-receptor interac	17	1.52%	0.673005
PIRSF002406:vertebrate rhodopsin	4	0.36%	0.841308
transducer	20	1.79%	0.952739
g-protein coupled receptor	18	1.61%	0.952987
IPR000276:Rhodopsin-like GPCR superfamily	12	1.07%	0.999899
receptor	36	3.22%	0.999976
GO:0007166~cell surface receptor linked sigr	76	6.80%	1
GO:0060089~molecular transducer activity	86	7.69%	1
GO:0004871~signal transducer activity	86	7.69%	1
GO:0007186~G-protein coupled receptor prot	44	3.94%	1
GO:0004872~receptor activity	60	5.37%	1
GO:0004888~transmembrane receptor activit	38	3.40%	1
GO:0004930~G-protein coupled receptor acti	27	2.42%	1
GO:0001584~rhodopsin-like receptor activity	16	1.43%	1

Median: 0.7093029113193254 Geo: 0.4274336816670745

Term	Count	%	PValue
IPR013753:Ras	9	0.81%	0.152954
IPR003579:Ras small GTPase, Rab type	3	0.27%	0.709303
SM00175:RAB	3	0.27%	0.719805

Median: 0.3316371203131925 Geo: 0.43087329622529147

Term	Count	%	PValue
GO:0031226~intrinsic to plasma membrane	57	5.10%	0.258809
GO:0044459~plasma membrane part	87	7.78%	0.295558
GO:0005887~integral to plasma membrane	55	4.92%	0.325735
GO:0005886~plasma membrane	106	9.48%	0.337539
topological domain:Cytoplasmic	69	6.17%	0.764369
topological domain:Extracellular	43	3.85%	0.99537

Median: 0.44945922077534506 Geo: 0.43464513235413105

Term	Count	%	PValue
GO:0051094~positive regulation of developm	8	0.72%	0.319107
GO:0048871~multicellular organismal homeo	4	0.36%	0.449459
GO:0001894~tissue homeostasis	4	0.36%	0.449459
GO:0045453~bone resorption	3	0.27%	0.553633

Median: 0.685960066589173 Geo: 0.4362470704578489

Term	Count	%	PValue
GO:0018193~peptidyl-amino acid modificatio	10	0.89%	0.171207
GO:0018108~peptidyl-tyrosine phosphorylatio	4	0.36%	0.68596
GO:0018212~peptidyl-tyrosine modification	4	0.36%	0.706929

Median: 0.40963610445819626 Geo: 0.44729887337555274

Term	Count	%	PValue
repeat:2	6	0.54%	0.388537

repeat:1	6	0.54%	0.388537
repeat:3	5	0.45%	0.430735
repeat:4	4	0.36%	0.615625

Median: 0.4774561483027405	Geo: 0.4484195189382892		
Term	Count	%	PValue
GO:0005739~mitochondrion	48	4.29%	0.270112
GO:0031966~mitochondrial membrane	17	1.52%	0.338256
GO:0019866~organelle inner membrane	15	1.34%	0.439673
GO:0005743~mitochondrial inner membrane	14	1.25%	0.470146
GO:0005740~mitochondrial envelope	17	1.52%	0.484766
GO:0031967~organelle envelope	23	2.06%	0.556712
GO:0031975~envelope	23	2.06%	0.556712
GO:0044429~mitochondrial part	20	1.79%	0.576142

Median: 0.4293023416983348	Geo: 0.4612341902779416		
Term	Count	%	PValue
GO:0015629~actin cytoskeleton	15	1.34%	0.308413
GO:0016459~myosin complex	5	0.45%	0.429302
GO:0003774~motor activity	8	0.72%	0.741087

Median: 0.48151244734237886	Geo: 0.47505105782779394		
Term	Count	%	PValue
GO:0007368~determination of left/right symr	3	0.27%	0.462387
GO:0009855~determination of bilateral symn	3	0.27%	0.481512
GO:0009799~determination of symmetry	3	0.27%	0.481512

Median: 0.4460592396549603	Geo: 0.4885246371145563		
Term	Count	%	PValue
GO:0015291~secondary active transmembrai	13	1.16%	0.32978
GO:0015293~symporter activity	9	0.81%	0.421411
GO:0022804~active transmembrane transpoı	22	1.97%	0.446059
GO:0015294~solute:cation symporter activity	5	0.45%	0.584325
GO:0015370~solute:sodium symporter activit	3	0.27%	0.768165

Median: 0.5088209052108131	Geo: 0.4962598576338997		
Term	Count	%	PValue
GO:0042733~embryonic digit morphogenesis	3	0.27%	0.127677
GO:0030326~embryonic limb morphogenesis	4	0.36%	0.494285
GO:0035113~embryonic appendage morphog	4	0.36%	0.494285
GO:0035108~limb morphogenesis	4	0.36%	0.508821
GO:0035107~appendage morphogenesis	4	0.36%	0.508821
GO:0048736~appendage development	4	0.36%	0.508821
GO:0060173~limb development	4	0.36%	0.508821
GO:0007389~pattern specification process	10	0.89%	0.54639
GO:0009952~anterior/posterior pattern forma	5	0.45%	0.58499
GO:0003002~regionalization	6	0.54%	0.754437
GO:0048598~embryonic morphogenesis	6	0.54%	0.89166

Median: 0.6014490269812658	Geo: 0.4973764616269408		
Term	Count	%	PValue
GO:0046466~membrane lipid catabolic proce	3	0.27%	0.297004
GO:0016042~lipid catabolic process	6	0.54%	0.601449
GO:0044242~cellular lipid catabolic process	3	0.27%	0.688803

Median: 0.8277224214823803	Geo: 0.5022243656471842		
Term	Count	%	PValue
propeptide:Removed in mature form	9	0.81%	0.175615
GO:0048503~GPI anchor binding	3	0.27%	0.827722
gpi-anchor	3	0.27%	0.87146

Median: 0.7482201941461548	Geo: 0.5043113219971841		
Term	Count	%	PValue
IPR013089:Kelch related	3	0.27%	0.176793
IPR013069:BTB/POZ	4	0.36%	0.390294
IPR011333:BTB/POZ fold	4	0.36%	0.74822
IPR000210:BTB/POZ-like	4	0.36%	0.767953
SM00225:BTB	4	0.36%	0.82276

Median: 0.6629167877214965	Geo: 0.5180844925108304		
Term	Count	%	PValue
GO:0016891~endoribonuclease activity, prod	3	0.27%	0.280132
GO:0016893~endonuclease activity, active w	3	0.27%	0.300856
GO:0004519~endonuclease activity	5	0.45%	0.636226
GO:0004540~ribonuclease activity	4	0.36%	0.689608
GO:0004521~endoribonuclease activity	3	0.27%	0.715294
GO:0004518~nuclease activity	7	0.63%	0.731114

Median: 0.5088209052108131	Geo: 0.5218217098726277		
Term	Count	%	PValue
GO:0042552~myelination	4	0.36%	0.464591
GO:0007272~ensheathment of neurons	4	0.36%	0.508821
GO:0008366~axon ensheathment	4	0.36%	0.508821
GO:0001508~regulation of action potential	4	0.36%	0.616436

Median: 0.5849051118463864	Geo: 0.5232757559198661		
Term	Count	%	PValue
IPR013087:Zinc finger, C2H2-type/integrase, I	8	0.72%	0.332645
IPR015880:Zinc finger, C2H2-like	9	0.81%	0.543597
SM00355:ZnF_C2H2	9	0.81%	0.584905
IPR007087:Zinc finger, C2H2-type	12	1.07%	0.585557
IPR001909:KRAB box	3	0.27%	0.633491

Median: 0.6445007927650344	Geo: 0.5232873981612741		
Term	Count	%	PValue
GO:0009401~phosphoenolpyruvate-depende	3	0.27%	0.189177

GO:0015293~symporter activity	9	0.81%	0.421411
GO:0005351~sugar:hydrogen ion symporter a	3	0.27%	0.610061
GO:0051119~sugar transmembrane transport	3	0.27%	0.67894
GO:0015144~carbohydrate transmembrane t	3	0.27%	0.748128
GO:0008643~carbohydrate transport	4	0.36%	0.831163

Median: 0.5536331420138444 Geo: 0.5271280856259419

Term	Count	%	PValue
GO:0000096~sulfur amino acid metabolic pro	3	0.27%	0.297004
GO:0044272~sulfur compound biosynthetic p	3	0.27%	0.553633
GO:0006790~sulfur metabolic process	4	0.36%	0.890767

Median: 0.601409864525576 Geo: 0.5314855310753033

Term	Count	%	PValue
GO:0051174~regulation of phosphorus metab	6	0.54%	0.383436
GO:0019220~regulation of phosphate metabo	6	0.54%	0.383436
GO:0042325~regulation of phosphorylation	5	0.45%	0.562107
GO:0001932~regulation of protein amino acid	4	0.36%	0.640713
GO:0033238~regulation of amine metabolic p	4	0.36%	0.652439
GO:0006521~regulation of amino acid metab	4	0.36%	0.652439

Median: 0.6966402340618993 Geo: 0.5381382996526384

Term	Count	%	PValue
GO:0050869~negative regulation of B cell act	3	0.27%	0.072716
GO:0051250~negative regulation of lymphocy	3	0.27%	0.382033
GO:0002521~leukocyte differentiation	8	0.72%	0.385125
GO:0050864~regulation of B cell activation	3	0.27%	0.442854
GO:0030183~B cell differentiation	3	0.27%	0.536282
GO:0051249~regulation of lymphocyte activa	5	0.45%	0.538554
GO:0050865~regulation of cell activation	5	0.45%	0.562107
GO:0042113~B cell activation	4	0.36%	0.628711
GO:0045321~leukocyte activation	11	0.98%	0.69664
GO:0050670~regulation of lymphocyte prolife	3	0.27%	0.701535
GO:0032944~regulation of mononuclear cell p	3	0.27%	0.701535
GO:0032943~mononuclear cell proliferation	4	0.36%	0.706929
GO:0046651~lymphocyte proliferation	4	0.36%	0.706929
GO:0051251~positive regulation of lymphocy	3	0.27%	0.713831
GO:0001775~cell activation	12	1.07%	0.731025
GO:0046649~lymphocyte activation	8	0.72%	0.773536
GO:0030098~lymphocyte differentiation	4	0.36%	0.795871

Median: 0.5464358420691726 Geo: 0.5464881667450793

Term	Count	%	PValue
repeat:LRR 4	4	0.36%	0.296977
repeat:LRR 3	4	0.36%	0.37346
repeat:LRR 2	4	0.36%	0.411215
repeat:LRR 1	4	0.36%	0.411215
leucine-rich repeat	6	0.54%	0.681657

IPR001611:Leucine-rich repeat	6	0.54%	0.823577
IPR003591:Leucine-rich repeat, typical subtyp	3	0.27%	0.862004
SM00369:LRR_TYP	3	0.27%	0.876519

Median: 0.5819051364898118 Geo: 0.5596950020270177

Term	Count	%	PValue
GO:0048646~anatomical structure formation	12	1.07%	0.301312
GO:0048514~blood vessel morphogenesis	10	0.89%	0.54639
GO:0001568~blood vessel development	11	0.98%	0.577108
GO:0001525~angiogenesis	8	0.72%	0.586703
GO:0001944~vasculature development	11	0.98%	0.605447
GO:0009887~organ morphogenesis	19	1.70%	0.910833

Median: 0.7146631166959486 Geo: 0.5600330435588263

Term	Count	%	PValue
GO:0007626~locomotory behavior	13	1.16%	0.260627
rno04620:Toll-like receptor signaling pathway	6	0.54%	0.689423
GO:0042330~taxis	6	0.54%	0.739903
GO:0006935~chemotaxis	6	0.54%	0.739903

Median: 0.595986061875998 Geo: 0.5617526454231732

Term	Count	%	PValue
rno01040:Polyunsaturated fatty acid biosynth	3	0.27%	0.319739
lipid synthesis	5	0.45%	0.462802
fatty acid biosynthesis	3	0.27%	0.516909
GO:0006633~fatty acid biosynthetic process	4	0.36%	0.675063
GO:0046394~carboxylic acid biosynthetic pro	4	0.36%	0.780118
GO:0016053~organic acid biosynthetic proce:	4	0.36%	0.780118

Median: 0.6014490269812658 Geo: 0.5625121080973473

Term	Count	%	PValue
GO:0004623~phospholipase A2 activity	3	0.27%	0.42142
GO:0004091~carboxylesterase activity	7	0.63%	0.42217
GO:0016042~lipid catabolic process	6	0.54%	0.601449
GO:0004620~phospholipase activity	4	0.36%	0.668674
GO:0016298~lipase activity	4	0.36%	0.787124

Median: 0.5787287488791943 Geo: 0.5668905827307934

Term	Count	%	PValue
GO:0051128~regulation of cellular componen	6	0.54%	0.33732
GO:0008064~regulation of actin polymerizati	3	0.27%	0.536282
GO:0030832~regulation of actin filament leng	3	0.27%	0.553633
GO:0032535~regulation of cellular componen	3	0.27%	0.57052
GO:0032956~regulation of actin cytoskeleton	3	0.27%	0.586938
GO:0051493~regulation of cytoskeleton orga	3	0.27%	0.662001
GO:0033043~regulation of organelle organiza	3	0.27%	0.662001
GO:0008154~actin polymerization and/or dep	3	0.27%	0.7257

Median: 0.5999486537353009	Geo: 0.5669168044626316		
Term	Count	%	PValue
GO:0043123~positive regulation of I-kappaB	6	0.54%	0.394966
GO:0043122~regulation of I-kappaB kinase/N	6	0.54%	0.452147
GO:0007249~I-kappaB kinase/NF-kappaB cas	7	0.63%	0.74775
GO:0009967~positive regulation of signal tra	8	0.72%	0.773536

Median: 1.0	Geo: 0.5670533146296532		
Term	Count	%	PValue
GO:0009582~detection of abiotic stimulus	5	0.45%	0.170357
GO:0009581~detection of external stimulus	5	0.45%	0.26816
GO:0050954~sensory perception of mechanic	5	0.45%	0.412681
GO:0007608~sensory perception of smell	5	0.45%	1
GO:0007606~sensory perception of chemical	6	0.54%	1
GO:0050906~detection of stimulus during se	5	0.45%	1
GO:0051606~detection of stimulus	7	0.63%	1

Median: 0.609218857807939	Geo: 0.5802975779525337		
Term	Count	%	PValue
GO:0009890~negative regulation of biosynth	4	0.36%	0.434156
GO:0017148~negative regulation of translatio	3	0.27%	0.57052
GO:0031327~negative regulation of cellular b	3	0.27%	0.647918
GO:0051248~negative regulation of protein n	5	0.45%	0.706588

Median: 0.6213308430829785	Geo: 0.6021493472760941		
Term	Count	%	PValue
IPR001440:Tetratricopeptide TPR-1	4	0.36%	0.509556
IPR011990:Tetratricopeptide-like helical	5	0.45%	0.592901
TPR repeat	4	0.36%	0.621331
IPR013026:Tetratricopeptide region	4	0.36%	0.642694
SM00028:TPR	4	0.36%	0.656174

Median: 0.6928277524443143	Geo: 0.6074386132124397		
Term	Count	%	PValue
GO:0055086~nucleobase, nucleoside and nuc	21	1.88%	0.079074
GO:0009117~nucleotide metabolic process	19	1.70%	0.109776
GO:0015992~proton transport	7	0.63%	0.358964
GO:0006163~purine nucleotide metabolic pro	8	0.72%	0.394626
GO:0006818~hydrogen transport	7	0.63%	0.421131
hydrogen ion transport	4	0.36%	0.435119
GO:0009144~purine nucleoside triphosphate	6	0.54%	0.46343
GO:0009165~nucleotide biosynthetic process	10	0.89%	0.498742
GO:0009141~nucleoside triphosphate metab	6	0.54%	0.507789
GO:0006164~purine nucleotide biosynthetic p	6	0.54%	0.540067
GO:0015077~monovalent inorganic cation tra	8	0.72%	0.566816
GO:0009108~coenzyme biosynthetic process	6	0.54%	0.601449
GO:0009199~ribonucleoside triphosphate me	5	0.45%	0.6286
GO:0009205~purine ribonucleoside triphosph	5	0.45%	0.6286

GO:0016469~proton-transporting two-sector A	4	0.36%	0.632594
GO:0009150~purine ribonucleotide metabolic	6	0.54%	0.639677
GO:0022890~inorganic cation transmembrane	10	0.89%	0.64058
GO:0009152~purine ribonucleotide biosynthe	5	0.45%	0.669169
GO:0009201~ribonucleoside triphosphate bio	4	0.36%	0.68596
GO:0009206~purine ribonucleoside triphosph	4	0.36%	0.68596
GO:0009145~purine nucleoside triphosphate	4	0.36%	0.68596
GO:0015662~ATPase activity, coupled to tran	4	0.36%	0.699695
GO:0009259~ribonucleotide metabolic proces	6	0.54%	0.700848
GO:0009142~nucleoside triphosphate biosynt	4	0.36%	0.706929
GO:0009260~ribonucleotide biosynthetic proc	5	0.45%	0.715446
GO:0046034~ATP metabolic process	4	0.36%	0.717004
GO:0015405~P-P-bond-hydrolysis-driven trans	9	0.81%	0.733388
GO:0015399~primary active transmembrane	9	0.81%	0.733388
GO:0046961~hydrogen ion transporting ATPa	3	0.27%	0.737562
GO:0015986~ATP synthesis coupled proton tr	3	0.27%	0.748188
GO:0043492~ATPase activity, coupled to mov	8	0.72%	0.775888
GO:0006753~nucleoside phosphate metabolic	3	0.27%	0.788436
GO:0006754~ATP biosynthetic process	3	0.27%	0.788436
GO:0015078~hydrogen ion transmembrane tr	6	0.54%	0.788459
GO:0042625~ATPase activity, coupled to tran	5	0.45%	0.805609
GO:0019829~cation-transporting ATPase activ	3	0.27%	0.848745
GO:0042626~ATPase activity, coupled to tran	7	0.63%	0.871155
GO:0006119~oxidative phosphorylation	4	0.36%	0.876716
GO:0016820~hydrolase activity, acting on aci	7	0.63%	0.888939
GO:0042623~ATPase activity, coupled	12	1.07%	0.897126
rno00190:Oxidative phosphorylation	5	0.45%	0.94111
GO:0016887~ATPase activity	13	1.16%	0.952816

Median: 0.7013168766235801 Geo: 0.6297977628010818

Term	Count	%	PValue
GO:0048511~rhythmic process	9	0.81%	0.276768
GO:0001541~ovarian follicle development	3	0.27%	0.402649
GO:0048608~reproductive structure developm	5	0.45%	0.502071
GO:0008406~gonad development	5	0.45%	0.502071
GO:0045137~development of primary sexual	5	0.45%	0.596168
GO:0022601~menstrual cycle phase	3	0.27%	0.662001
GO:0022602~menstrual cycle process	3	0.27%	0.675627
GO:0008585~female gonad development	3	0.27%	0.688803
GO:0046660~female sex differentiation	3	0.27%	0.713831
GO:0046545~development of primary female	3	0.27%	0.713831
GO:0042698~menstrual cycle	3	0.27%	0.7257
GO:0007548~sex differentiation	5	0.45%	0.756788
GO:0003006~reproductive developmental pro	5	0.45%	0.786396
GO:0032504~multicellular organism reproduc	4	0.36%	0.803388
GO:0048609~reproductive process in a multic	4	0.36%	0.803388
GO:0022414~reproductive process	13	1.16%	0.835992

Median: 0.5984469718441101	Geo: 0.6323830465290332		
Term	Count	%	PValue
GO:0007276~gamete generation	15	1.34%	0.566156
GO:0048232~male gamete generation	11	0.98%	0.598447
GO:0007283~spermatogenesis	11	0.98%	0.598447
GO:0019953~sexual reproduction	17	1.52%	0.624193
GO:0000003~reproduction	25	2.24%	0.799089

Median: 0.6418794153582141	Geo: 0.6434810458513377		
Term	Count	%	PValue
domain:C2 2	3	0.27%	0.557944
domain:C2 1	3	0.27%	0.557944
IPR000008:C2 calcium-dependent membrane	5	0.45%	0.725815
SM00239:C2	5	0.45%	0.758815

Median: 0.5705196311967391	Geo: 0.6563294464415198		
Term	Count	%	PValue
GO:0040014~regulation of multicellular orgar	3	0.27%	0.536282
GO:0035264~multicellular organism growth	3	0.27%	0.57052
GO:0040008~regulation of growth	7	0.63%	0.924063

Median: 0.7446078402660412	Geo: 0.6575850095074384		
Term	Count	%	PValue
GO:0000785~chromatin	10	0.89%	0.502927
GO:0005694~chromosome	15	1.34%	0.744608
GO:0044427~chromosomal part	13	1.16%	0.759317

Median: 0.5620393231910041	Geo: 0.6810252212277741		
Term	Count	%	PValue
GO:0001608~nucleotide receptor activity, G-p	4	0.36%	0.562039
GO:0016502~nucleotide receptor activity	4	0.36%	0.562039
IPR000276:Rhodopsin-like GPCR superfamily	12	1.07%	0.999899

Median: 0.6427726332095793	Geo: 0.7001764684951715		
Term	Count	%	PValue
IPR002126:Cadherin	4	0.36%	0.61543
SM00112:CA	4	0.36%	0.642773
GO:0007156~homophilic cell adhesion	6	0.54%	0.867733

Median: 0.793690309997469	Geo: 0.7071541215403462		
Term	Count	%	PValue
GO:0045860~positive regulation of protein ki	9	0.81%	0.459439
GO:0043405~regulation of MAP kinase activit	5	0.45%	0.764474
GO:0000187~activation of MAPK activity	3	0.27%	0.822907
GO:0043406~positive regulation of MAP kinas	3	0.27%	0.865197

Median: 0.779359351302318	Geo: 0.708304923911514		
Term	Count	%	PValue

GO:0009605~response to external stimulus	42	3.76%	0.465649
GO:0006954~inflammatory response	17	1.52%	0.676617
GO:0009611~response to wounding	23	2.06%	0.882102
GO:0006952~defense response	22	1.97%	0.905651

Median: 0.8552131779570957 Geo: 0.7091622268294516

Term	Count	%	PValue
GO:0022892~substrate-specific transporter a	65	5.81%	0.10346
GO:0005215~transporter activity	81	7.25%	0.112579
GO:0022891~substrate-specific transmembra	48	4.29%	0.401987
GO:0022857~transmembrane transporter act	52	4.65%	0.446023
transport	57	5.10%	0.500143
GO:0022890~inorganic cation transmembran	10	0.89%	0.64058
GO:0015075~ion transmembrane transporter	37	3.31%	0.666394
GO:0006811~ion transport	40	3.58%	0.704556
GO:0006813~potassium ion transport	8	0.72%	0.743226
potassium	6	0.54%	0.751663
GO:0030955~potassium ion binding	5	0.45%	0.779334
GO:0008324~cation transmembrane transpor	26	2.33%	0.820017
potassium transport	5	0.45%	0.823461
ion transport	22	1.97%	0.854332
GO:0022803~passive transmembrane transp	17	1.52%	0.855213
GO:0015267~channel activity	17	1.52%	0.855213
Ionic channel	13	1.16%	0.888466
voltage-gated channel	6	0.54%	0.896856
GO:0015672~monovalent inorganic cation tra	14	1.25%	0.89821
GO:0006812~cation transport	23	2.06%	0.925976
GO:0030001~metal ion transport	18	1.61%	0.928702
GO:0022836~gated channel activity	11	0.98%	0.933086
GO:0005216~ion channel activity	14	1.25%	0.940465
GO:0031420~alkali metal ion binding	6	0.54%	0.950483
GO:0046873~metal ion transmembrane trans	12	1.07%	0.950868
GO:0022838~substrate specific channel activ	14	1.25%	0.954803
GO:0022832~voltage-gated channel activity	6	0.54%	0.957042
GO:0005244~voltage-gated ion channel activ	6	0.54%	0.957042
GO:0005267~potassium channel activity	4	0.36%	0.973171
GO:0005261~cation channel activity	8	0.72%	0.988112
GO:0022843~voltage-gated cation channel ac	3	0.27%	0.996715

Median: 0.6691685395032168 Geo: 0.7168766539023791

Term	Count	%	PValue
GO:0007179~transforming growth factor beta	4	0.36%	0.616436
GO:0007178~transmembrane receptor protei	5	0.45%	0.669169
GO:0007167~enzyme linked receptor protein	12	1.07%	0.89312

Median: 0.6660073746312482 Geo: 0.7181513531178823

Term	Count	%	PValue
GO:0004601~peroxidase activity	3	0.27%	0.666007

GO:0016684~oxidoreductase activity, acting	3	0.27%	0.666007
GO:0016209~antioxidant activity	3	0.27%	0.835007

Median: 0.7350892210174229 Geo: 0.7192759782543552

Term	Count	%	PValue
GO:0042692~muscle cell differentiation	4	0.36%	0.591074
GO:0014706~striated muscle development	6	0.54%	0.648871
GO:0007517~muscle development	8	0.72%	0.821308
GO:0007519~skeletal muscle development	4	0.36%	0.849722

Median: 0.9984766275751946 Geo: 0.7250339984678495

Term	Count	%	PValue
GO:0048029~monosaccharide binding	3	0.27%	0.382226
GO:0030246~carbohydrate binding	8	0.72%	0.998477
GO:0005529~sugar binding	5	0.45%	0.998658

Median: 0.7884358417100072 Geo: 0.7321529665083604

Term	Count	%	PValue
GO:0006730~one-carbon compound metaboli	6	0.54%	0.571327
GO:0043414~biopolymer methylation	3	0.27%	0.788436
GO:0032259~methylation	3	0.27%	0.871273

Median: 0.6691685395032168 Geo: 0.7523899992015123

Term	Count	%	PValue
GO:0051789~response to protein stimulus	5	0.45%	0.669169
GO:0006986~response to unfolded protein	5	0.45%	0.669169
GO:0009607~response to biotic stimulus	8	0.72%	0.95117

Median: 0.7994349606199997 Geo: 0.7551374387493411

Term	Count	%	PValue
Mitochondrion	23	2.06%	0.658318
transit peptide	12	1.07%	0.799435
transit peptide:Mitochondrion	9	0.81%	0.8182

Median: 0.8350065324763454 Geo: 0.7573085527457687

Term	Count	%	PValue
GO:0030594~neurotransmitter receptor activ	7	0.63%	0.565097
GO:0042165~neurotransmitter binding	7	0.63%	0.642168
GO:0004983~neuropeptide Y receptor activity	3	0.27%	0.652657
GO:0008188~neuropeptide receptor activity	3	0.27%	0.835007
GO:0042923~neuropeptide binding	3	0.27%	0.835007
GO:0042277~peptide binding	9	0.81%	0.886941
GO:0001653~peptide receptor activity	4	0.36%	0.975391

Median: 0.7481884961189249 Geo: 0.7666946662279888

Term	Count	%	PValue
GO:0002009~morphogenesis of an epithelium	6	0.54%	0.675549
GO:0016331~morphogenesis of embryonic ep	3	0.27%	0.748188

GO:0048598~embryonic morphogenesis	6	0.54%	0.89166

Median: 0.8437420023447036 Geo: 0.7823281244291606

Term	Count	%	PValue
GO:0016064~immunoglobulin mediated immu	4	0.36%	0.523135
GO:0019724~B cell mediated immunity	4	0.36%	0.55106
GO:0002449~lymphocyte mediated immunity	4	0.36%	0.663889
GO:0002460~adaptive immune response bas	4	0.36%	0.706929
GO:0002250~adaptive immune response	4	0.36%	0.706929
GO:0002443~leukocyte mediated immunity	4	0.36%	0.754627
GO:0006959~humoral immune response	4	0.36%	0.843742
GO:0050776~regulation of immune response	5	0.45%	0.847884
GO:0002682~regulation of immune system pi	5	0.45%	0.872566
immune response	3	0.27%	0.955648
GO:0002252~immune effector process	4	0.36%	0.971089
GO:0002526~acute inflammatory response	3	0.27%	0.98349
GO:0006955~immune response	12	1.07%	0.999982

Median: 0.8311187328631705 Geo: 0.7899641669263333

Term	Count	%	PValue
GO:0031965~nuclear membrane	5	0.45%	0.656212
GO:0044453~nuclear membrane part	4	0.36%	0.771129
GO:0005635~nuclear envelope	6	0.54%	0.831119
GO:0005643~nuclear pore	3	0.27%	0.833261
GO:0046930~pore complex	3	0.27%	0.877853

Median: 0.8000862740701508 Geo: 0.7916258582136574

Term	Count	%	PValue
GO:0042107~cytokine metabolic process	4	0.36%	0.706929
GO:0001816~cytokine production	5	0.45%	0.800086
GO:0042089~cytokine biosynthetic process	3	0.27%	0.877096

Median: 0.758827055718108 Geo: 0.7991755408655077

Term	Count	%	PValue
GO:0006497~protein amino acid lipidation	3	0.27%	0.7257
GO:0042158~lipoprotein biosynthetic process	3	0.27%	0.758827
GO:0042157~lipoprotein metabolic process	3	0.27%	0.926887

Median: 0.8160828494606095 Geo: 0.8081255298438712

Term	Count	%	PValue
GO:0016337~cell-cell adhesion	13	1.16%	0.792443
GO:0022610~biological adhesion	30	2.68%	0.816083
GO:0007155~cell adhesion	30	2.68%	0.816083

Median: 0.9232974364126381 Geo: 0.8238423678796846

Term	Count	%	PValue
GO:0016126~sterol biosynthetic process	3	0.27%	0.536282
GO:0016125~sterol metabolic process	4	0.36%	0.831163

GO:0008203~cholesterol metabolic process	3	0.27%	0.923297
GO:0006694~steroid biosynthetic process	3	0.27%	0.936727
GO:0008202~steroid metabolic process	5	0.45%	0.984434

Median: 0.9362941363435059	Geo: 0.8360218956586637		
Term	Count	%	PValue
GO:0005843~cytosolic small ribosomal subun	3	0.27%	0.408313
GO:0015935~small ribosomal subunit	3	0.27%	0.733306
ribonucleoprotein	8	0.72%	0.797397
GO:0005830~cytosolic ribosome (sensu Euka	3	0.27%	0.818051
rno03010:Ribosome	4	0.36%	0.848076
GO:0033279~ribosomal subunit	4	0.36%	0.936294
ribosomal protein	5	0.45%	0.941543
ribosome	3	0.27%	0.958014
GO:0030529~ribonucleoprotein complex	17	1.52%	0.996945
GO:0003735~structural constituent of ribosor	7	0.63%	0.99988
GO:0005840~ribosome	7	0.63%	0.999957

Median: 0.8700920567964969	Geo: 0.8419358538470354		
Term	Count	%	PValue
GO:0003777~microtubule motor activity	4	0.36%	0.728459
GO:0007018~microtubule-based movement	5	0.45%	0.847884
GO:0005875~microtubule associated complex	5	0.45%	0.8923
GO:0030705~cytoskeleton-dependent intrace	5	0.45%	0.911724

Median: 0.8453599387629621	Geo: 0.8498359253984246		
Term	Count	%	PValue
GO:0007126~meiosis	3	0.27%	0.84536
GO:0051327~M phase of meiotic cell cycle	3	0.27%	0.84536
GO:0051321~meiotic cell cycle	3	0.27%	0.858859

Median: 0.8437813998480348	Geo: 0.861373682922828		
Term	Count	%	PValue
GO:0005615~extracellular space	88	7.87%	0.774662
GO:0044421~extracellular region part	91	8.14%	0.843781
GO:0005576~extracellular region	94	8.41%	0.97776

Median: 0.8877030784420337	Geo: 0.866705571947067		
Term	Count	%	PValue
GO:0015179~L-amino acid transmembrane tr	3	0.27%	0.666007
GO:0006865~amino acid transport	4	0.36%	0.837557
GO:0015171~amino acid transmembrane trar	3	0.27%	0.848745
GO:0046942~carboxylic acid transport	5	0.45%	0.885656
GO:0015849~organic acid transport	5	0.45%	0.889751
GO:0015837~amine transport	4	0.36%	0.924703
GO:0046943~carboxylic acid transmembrane	3	0.27%	0.95976
GO:0005342~organic acid transmembrane tra	3	0.27%	0.961621

Median: 0.9354118585438735	Geo: 0.8733251539872539		
Term	Count	%	PValue
IPR000047:Helix-turn-helix motif, lambda-like	3	0.27%	0.633491
IPR001356:Homeobox	5	0.45%	0.921786
SM00389:HOX	5	0.45%	0.935412
IPR012287:Homeodomain-related	4	0.36%	0.95928
Homeobox	4	0.36%	0.969529

Median: 0.9013406927694274	Geo: 0.8824357299623315		
Term	Count	%	PValue
GO:0045792~negative regulation of cell size	3	0.27%	0.748188
GO:0016049~cell growth	7	0.63%	0.869474
GO:0008361~regulation of cell size	7	0.63%	0.891665
GO:0040007~growth	13	1.16%	0.911017
GO:0040008~regulation of growth	7	0.63%	0.924063
GO:0001558~regulation of cell growth	4	0.36%	0.966943

Median: 0.8924802767520075	Geo: 0.8834873488794694		
Term	Count	%	PValue
GO:0006816~calcium ion transport	6	0.54%	0.823628
calcium transport	3	0.27%	0.889928
GO:0015674~di-, tri-valent inorganic cation tr	7	0.63%	0.895033
GO:0030001~metal ion transport	18	1.61%	0.928702

Median: 0.981699345405155	Geo: 0.8890739249359461		
Term	Count	%	PValue
GO:0009897~external side of plasma membra	6	0.54%	0.404713
IPR003598:Immunoglobulin subtype 2	4	0.36%	0.945364
SM00408:IGc2	4	0.36%	0.963565
IPR013151:Immunoglobulin	4	0.36%	0.971303
SM00409:IG	7	0.63%	0.981699
IPR003599:Immunoglobulin subtype	5	0.45%	0.99162
immunoglobulin domain	6	0.54%	0.996245
IPR007110:Immunoglobulin-like	8	0.72%	0.999529
IPR013783:Immunoglobulin-like fold	7	0.63%	0.999939

Median: 0.8515833251974014	Geo: 0.8906688475145337		
Term	Count	%	PValue
GO:0046906~tetrapyrrole binding	8	0.72%	0.851583
GO:0020037~heme binding	8	0.72%	0.851583
GO:0004497~monooxygenase activity	4	0.36%	0.974304

Median: 0.9962434132282394	Geo: 0.8976463845659044		
Term	Count	%	PValue
membrane	163	14.58%	0.3784
topological domain:Cytoplasmic	69	6.17%	0.764369
transmembrane region	83	7.42%	0.872932
transmembrane	117	10.47%	0.910233

GO:0044425~membrane part	261	23.35%	0.977345
GO:0016020~membrane	313	28.00%	0.991044
topological domain:Extracellular	43	3.85%	0.99537
glycoprotein	85	7.60%	0.997117
glycosylation site:N-linked (GlcNAc...)	68	6.08%	0.998767
signal	60	5.37%	0.999805
GO:0031224~intrinsic to membrane	216	19.32%	0.999831
GO:0016021~integral to membrane	214	19.14%	0.999877
disulfide bond	43	3.85%	0.999931
signal peptide	51	4.56%	0.999983

Median: 0.9770492762883788 Geo: 0.898924811484142

Term	Count	%	PValue
Serine protease inhibitor	3	0.27%	0.659227
protease inhibitor	3	0.27%	0.850183
GO:0004867~serine-type endopeptidase inhib	3	0.27%	0.963399
GO:0004866~endopeptidase inhibitor activity	4	0.36%	0.9907
GO:0030414~protease inhibitor activity	4	0.36%	0.991503
GO:0004857~enzyme inhibitor activity	7	0.63%	0.994834

Median: 0.9024340852042524 Geo: 0.8997282058107576

Term	Count	%	PValue
GO:0042060~wound healing	8	0.72%	0.839849
GO:0042246~tissue regeneration	3	0.27%	0.871273
GO:0031099~regeneration	3	0.27%	0.933595
GO:0048589~developmental growth	3	0.27%	0.959249

Median: 0.9522219354712798 Geo: 0.9072825421771014

Term	Count	%	PValue
GO:0005201~extracellular matrix structural c	4	0.36%	0.771531
GO:0044420~extracellular matrix part	4	0.36%	0.89221
GO:0005578~proteinaceous extracellular mat	9	0.81%	0.952222
GO:0031012~extracellular matrix	9	0.81%	0.963879
extracellular matrix	3	0.27%	0.973042

Median: 0.9356066609929128 Geo: 0.9188051673289847

Term	Count	%	PValue
GO:0050776~regulation of immune response	5	0.45%	0.847884
GO:0002682~regulation of immune system pr	5	0.45%	0.872566
GO:0002253~activation of immune response	3	0.27%	0.926887
GO:0051240~positive regulation of multicellu	5	0.45%	0.944326
GO:0050778~positive regulation of immune r	3	0.27%	0.961214
GO:0002684~positive regulation of immune s	3	0.27%	0.966578

Median: 0.9414350302664871 Geo: 0.9229533304598382

Term	Count	%	PValue
GO:0008201~heparin binding	3	0.27%	0.795615
GO:0005539~glycosaminoglycan binding	3	0.27%	0.929526

GO:0030247~polysaccharide binding	3	0.27%	0.941435
GO:0001871~pattern binding	3	0.27%	0.963399
GO:0030246~carbohydrate binding	8	0.72%	0.998477

Median: 0.9456647511946196 Geo: 0.9352281958909899

Term	Count	%	PValue
GO:0006508~proteolysis	36	3.22%	0.876179
GO:0008233~peptidase activity	28	2.50%	0.945665
GO:0004175~endopeptidase activity	16	1.43%	0.98724

Median: 0.9504320986748979 Geo: 0.9412154711720149

Term	Count	%	PValue
ank repeat	4	0.36%	0.916184
IPR002110:Ankyrin	4	0.36%	0.950432
SM00248:ANK	4	0.36%	0.957554

Median: 0.9658152263361006 Geo: 0.9730433063544447

Term	Count	%	PValue
growth factor	4	0.36%	0.962854
GO:0005125~cytokine activity	8	0.72%	0.965815
GO:0008083~growth factor activity	4	0.36%	0.9907
