# Supplementary material for: Gene Expression Patterns Underlying the Reinstatement of Plasticity in the Adult Visual System
Source: Neural Plast. 2013 Jun 26;2013:605079. doi: 10.1155/2013/605079 (PMC3710606; doi:10.1155/2013/605079)
Supplement: Supplementary file 2 [file 605079.f2.pdf]

Supplementary Table 1

ID	logFC	t	P.Value	B	GenbankAccession	UniGeneID
A_44_P505500	1.607556	3.752646	0.00122	-3.55829	CK358354	Rn.91828
A_43_P12141	1.561647	3.810252	0.001065	-3.53561	NM_021869	Rn.1993
A_44_P992854	1.324382	3.501498	0.002197	-3.65908	NM_057132	Rn.107401
A_44_P196717	1.318426	3.242831	0.004003	-3.7657	XM_344970	Rn.154758
A_44_P113842	1.173214	4.709021	0.000128	-3.20753	NM_022210	Rn.4210
A_44_P403482	1.15254	4.076258	0.000569	-3.4333	XM_213493	Rn.107395
A_44_P248111	1.143281	5.541433	1.87E-05	-2.95081	NM_001047107	Rn.198138
A_43_P16511	1.072354	2.854558	0.009672	-3.92914	NM_001044300	Rn.19946
A_42_P522171	1.030953	3.730579	0.001285	-3.56702	XM_001081512	
A_44_P223252	1.015676	2.880587	0.009124	-3.91812	NM_031595	Rn.11173
A_44_P239105	1.014573	3.317443	0.003369	-3.7347	XM_233341	Rn.23612
A_44_P563128	1.013565	3.964694	0.00074	-3.47572	XR_007778	Rn.161878
A_44_P864185	0.99365	3.198517	0.004432	-3.78419	AW142955	Rn.24135
A_44_P255954	0.985852	2.916596	0.008416	-3.90288	XM_573223	Rn.21595
A_44_P548507	0.976934	3.322881	0.003327	-3.73245	NM_032083	Rn.11166
A_42_P713975	0.96633	3.382278	0.002899	-3.70791	BC082799	Rn.30992
A_43_P13247	0.945033	3.704721	0.001365	-3.57728	XM_001070141	Rn.64489
A_44_P187789	0.919478	3.765582	0.001183	-3.55318	XM_001066818	Rn.98517
A_43_P16512	0.917668	2.964885	0.007548	-3.88245	XM_218824	Rn.3618
A_44_P769703	0.915946	2.855509	0.009651	-3.92874	NM_178096	Rn.8180
A_44_P210461	0.909428	3.068536	0.005967	-3.83872	NM_017025	Rn.107896
A_44_P176606	0.891879	3.077713	0.005843	-3.83486	NM_001007235	Rn.2135
A_44_P480605	0.877686	2.898617	0.008763	-3.91049	XM_001066230	Rn.196993
A_44_P1029397	0.874681	3.029251	0.006524	-3.85528	NM_198134	Rn.32168
A_44_P463858	0.858747	3.837533	0.000999	-3.52494	NM_133303	Rn.10784
A_44_P189326	0.845271	3.308173	0.003442	-3.73854	NM_017319	Rn.11527
A_43_P10162	0.837812	2.967703	0.0075	-3.88126	XM_224841	Rn.206094
A_43_P11622	0.797153	3.378305	0.002926	-3.70955	NM_012925	Rn.1231
A_44_P552865	0.794559	2.92484	0.008261	-3.89939	XM_221896	Rn.25814
A_44_P508264	0.752347	3.526847	0.002071	-3.64878	NM_031762	Rn.29897
A_44_P424570	0.739812	3.454555	0.002451	-3.67824	XM_235521	Rn.3893
A_43_P12779	0.734704	3.025752	0.006576	-3.85675	NM_053346	Rn.3546
A_44_P379976	0.719224	2.852731	0.009711	-3.92992	NM_001024782	Rn.33512
A_44_P797926	0.71717	2.871817	0.009305	-3.92183	BG673275	Rn.199501
A_44_P506374	0.709429	3.190639	0.004513	-3.78748	XM_001073596	Rn.133954
A_44_P356658	0.709393	2.874548	0.009249	-3.92068	XM_219716	Rn.28086
A_44_P108421	0.689004	3.061731	0.00606	-3.84159	XM_224350	Rn.100627
A_43_P16811	0.685518	2.937183	0.008035	-3.89417	NM_001014073	Rn.12770
A_44_P473322	0.680296	3.094868	0.005619	-3.82764	XM_224778	
A_44_P417077	0.679432	3.552775	0.001949	-3.63826	XM_573664	Rn.63942
A_44_P403410	0.65013	3.218297	0.004235	-3.77593	NM_001079895	Rn.137551
A_44_P714315	0.649504	3.019091	0.006677	-3.85956	CO404950	Rn.45255
A_44_P501483	0.646159	3.063059	0.006042	-3.84103	XR_006667	Rn.216235
A_44_P156966	0.642122	2.94394	0.007913	-3.89131	XM_228708	Rn.106891
A_44_P350794	0.5956	2.869386	0.009356	-3.92286	AA894317	Rn.11166
A_43_P10735	0.590686	2.854672	0.009669	-3.9291	BF555100	Rn.168452
A_43_P17622	0.586863	3.108625	0.005446	-3.82186	XM_345870	Rn.52275
A_42_P704370	0.57628	2.905127	0.008636	-3.90773	NM_023972	Rn.64512
A_44_P448307	0.576147	3.209166	0.004325	-3.77974	XM_217432	Rn.93317
A_44_P473472	0.569886	2.910531	0.008531	-3.90544	BC091233	Rn.9497
A_43_P21003	0.560543	3.007822	0.006849	-3.86432	NM_001033892	Rn.98615
A_44_P738831	0.55264	3.209084	0.004326	-3.77977	BE105152	Rn.148459

A_44_P189813	0.551359	3.113113	0.00539	-3.81998	XM_214295	Rn.105830
A_44_P161220	0.542591	2.893728	0.008859	-3.91256	NM_080482	Rn.211646
A_43_P19892	0.536862	3.04624	0.006277	-3.84811	NM_001014255	Rn.22786
A_44_P108680	0.53537	3.014469	0.006747	-3.86151	NM_057116	Rn.54550
A_44_P229618	0.527464	2.895836	0.008818	-3.91166	XM_228065	Rn.46413
A_44_P255236	0.525992	2.841785	0.009952	-3.93456	NM_019212	Rn.82732
A_44_P455065	0.518588	3.05703	0.006125	-3.84357	XM_001077680	Rn.2153
A_44_P208275	0.511361	3.692287	0.001406	-3.58223	NM_031795	Rn.24091
A_43_P15848	0.510606	2.965172	0.007543	-3.88233	M81783	Rn.91148
A_44_P180294	0.507879	3.015028	0.006738	-3.86128	XM_344235	
A_44_P180676	0.507554	3.147503	0.004982	-3.80554	XM_341141	Rn.206334
A_44_P274573	0.507395	2.967906	0.007497	-3.88118	XM_232283	Rn.21111
A_44_P222865	0.490304	2.871324	0.009316	-3.92204	XR_006686	Rn.197425
A_44_P101023	0.487674	2.909696	0.008547	-3.9058	XM_239335	Rn.22437
A_44_P194863	0.470415	3.085206	0.005744	-3.83171	XM_220574	Rn.102346
A_42_P573610	0.468416	3.286319	0.003621	-3.74761	XM_233544	Rn.62413
A_44_P142843	0.46753	2.877131	0.009195	-3.91958	XM_001065442	Rn.144456
A_42_P707937	0.463584	3.032801	0.006472	-3.85378	NM_080583	Rn.56138
A_44_P549028	0.462526	3.089647	0.005687	-3.82984	XM_001076082	Rn.207473
A_43_P17264	0.458705	2.931389	0.00814	-3.89662	XM_240329	Rn.19314
A_44_P720473	0.453531	2.941883	0.00795	-3.89218	CA512655	Rn.24394
A_44_P267155	0.448921	2.921416	0.008325	-3.90084	XM_221887	
A_44_P827752	0.443672	2.90014	0.008733	-3.90984		
A_43_P18570	0.433168	3.293144	0.003564	-3.74477	XM_236194	Rn.62341
A_43_P21027	0.433031	2.984758	0.007216	-3.87406	XM_235023	Rn.162943
A_44_P195291	0.431798	3.387985	0.002861	-3.70556	XM_233382	Rn.41038
A_43_P23298	0.431665	3.262797	0.003823	-3.75738	XM_001079737	Rn.161833
A_44_P1005858	0.428954	2.877915	0.009179	-3.91925	CO404131	Rn.21816
A_44_P147980	0.42782	3.280565	0.003669	-3.75	XM_576503	Rn.203950
A_44_P546901	0.41725	2.967204	0.007509	-3.88147	NM_012502	Rn.9813
A_44_P304493	0.417047	3.129617	0.005191	-3.81305	NM_001024970	Rn.213413
A_43_P21762	0.387674	2.984323	0.007224	-3.87424	NM_001013187	Rn.19440
A_44_P333550	0.370196	2.879751	0.009141	-3.91847	NM_001013986	Rn.23222
A_44_P119442	0.36235	2.963593	0.00757	-3.883	XM_216492	
A_44_P277126	0.362131	2.942351	0.007942	-3.89198	XM_221369	Rn.3336
A_44_P133226	0.361641	2.994234	0.007063	-3.87005	XM_224852	Rn.61768
A_42_P601971	0.351093	3.207843	0.004338	-3.78029	XM_220328	Rn.163114
A_42_P504240	-0.30416	-2.95103	0.007788	-3.88831	XM_237998	Rn.64941
A_44_P637629	-0.33755	-2.90152	0.008706	-3.90926	DV727233	Rn.25112
A_42_P771373	-0.35205	-2.91754	0.008398	-3.90248	NM_012614	Rn.9714
A_44_P561760	-0.37186	-2.84266	0.009932	-3.93418		
A_44_P479766	-0.3729	-2.93174	0.008134	-3.89647	BM986564	Rn.90077
A_44_P535706	-0.37591	-2.95306	0.007752	-3.88745	XM_216485	Rn.8332
A_42_P626023	-0.381	-3.01965	0.006668	-3.85932	XM_224232	Rn.19524
A_44_P255384	-0.38354	-2.87039	0.009335	-3.92244	XM_344225	
A_42_P570848	-0.38813	-2.9898	0.007135	-3.87193	NM_017091	Rn.11384
A_42_P791872	-0.39568	-2.8814	0.009108	-3.91777	XM_001061587	Rn.160555
A_44_P212695	-0.39929	-2.85185	0.00973	-3.93029	XM_212890	
A_42_P549321	-0.40263	-3.27203	0.003742	-3.75355		
A_44_P473731	-0.40425	-2.87113	0.00932	-3.92213	XM_220717	Rn.44897
A_44_P762268	-0.40531	-3.05625	0.006136	-3.84389		
A_44_P792063	-0.40636	-3.28687	0.003616	-3.74738		
A_44_P593331	-0.40716	-3.09169	0.00566	-3.82898		
A_44_P501390	-0.41007	-2.88437	0.009047	-3.91652	XM_001055728	Rn.199171

A_44_P884739	-0.41187	-3.14865	0.004969	-3.80506	XM_219339	Rn.30285
A_44_P1043979	-0.41616	-3.85418	0.000961	-3.51844	NM_001030052	Rn.156413
A_44_P161709	-0.41899	-3.11086	0.005418	-3.82092	XM_345662	
A_42_P634674	-0.42025	-2.92773	0.008207	-3.89816	NM_145783	Rn.11077
A_43_P11268	-0.42079	-2.98206	0.007261	-3.8752	NM_031762	Rn.29897
A_44_P525486	-0.44011	-2.84255	0.009935	-3.93423	XM_343841	Rn.101173
A_44_P592613	-0.44444	-2.92157	0.008322	-3.90077	XM_576601	Rn.208293
A_42_P703403	-0.44803	-3.75604	0.00121	-3.55695	NM_031974	Rn.112599
A_44_P866539	-0.44865	-3.35849	0.003064	-3.71773	AW913986	Rn.40480
A_44_P554867	-0.4491	-3.0488	0.006241	-3.84703	AW917688	Rn.16899
A_44_P792470	-0.45652	-3.23669	0.00406	-3.76825	DV722661	Rn.147243
A_43_P10505	-0.45976	-2.86522	0.009444	-3.92463	XM_226549	Rn.19188
A_44_P995316	-0.46086	-2.99405	0.007066	-3.87013	XM_343081	Rn.8423
A_44_P1055366	-0.46108	-3.07222	0.005917	-3.83717	AW919175	Rn.18892
A_44_P914323	-0.46109	-2.84335	0.009917	-3.93389	DV718684	Rn.130237
A_43_P16707	-0.46278	-2.99797	0.007004	-3.86848	NM_001004202	Rn.7857
A_44_P730454	-0.46305	-2.87433	0.009253	-3.92077	XM_341399	Rn.4090
A_42_P623151	-0.46981	-3.45868	0.002427	-3.67656	NM_031026	Rn.31981
A_44_P638345	-0.46998	-2.84292	0.009927	-3.93408	NM_001034130	Rn.11866
A_44_P152635	-0.47158	-3.14505	0.00501	-3.80657	NM_021857	Rn.44301
A_44_P822928	-0.47162	-3.90407	0.000854	-3.49907		
A_44_P156922	-0.47346	-2.90639	0.008611	-3.9072	NM_001008312	Rn.3775
A_44_P745579	-0.47912	-3.23123	0.004111	-3.77053		
A_44_P1031514	-0.48047	-3.09335	0.005639	-3.82828	NM_001014054	Rn.7056
A_44_P361202	-0.48173	-3.34691	0.003147	-3.72251	CB569688	Rn.14868
A_42_P619248	-0.48445	-3.09476	0.005621	-3.82769	XM_001053199	Rn.6409
A_44_P392886	-0.48485	-3.37934	0.002919	-3.70912	CB567353	Rn.203235
A_42_P614711	-0.49463	-3.3023	0.003489	-3.74098	BF548552	Rn.163711
A_42_P475623	-0.49595	-2.98704	0.007179	-3.87309	NM_053394	Rn.8954
A_43_P12692	-0.49722	-2.90714	0.008597	-3.90688	NM_031814	Rn.34521
A_44_P727480	-0.49961	-3.08582	0.005736	-3.83145	AW142602	Rn.164775
A_44_P976007	-0.50124	-2.99546	0.007044	-3.86953	DV719080	Rn.47100
A_44_P357478	-0.50317	-2.99207	0.007098	-3.87097	CK481254	Rn.199106
A_44_P775118	-0.5039	-3.42995	0.002595	-3.68832	NM_001014205	Rn.12767
A_44_P372261	-0.50553	-2.9543	0.00773	-3.88693	NM_053372	Rn.18560
A_44_P965429	-0.5063	-2.84121	0.009965	-3.9348		
A_44_P393551	-0.50772	-3.02511	0.006586	-3.85702	NM_012567	Rn.10346
A_44_P700333	-0.50776	-3.69479	0.001397	-3.58124	CX570601	Rn.124042
A_43_P15460	-0.50937	-2.92906	0.008183	-3.8976	NM_053879	Rn.10117
A_44_P299247	-0.51546	-2.87932	0.00915	-3.91866	NM_012778	Rn.1618
A_44_P311334	-0.51691	-2.96234	0.007592	-3.88353	L27060	Rn.163460
A_44_P996952	-0.51744	-2.96827	0.007491	-3.88102	NM_001039454	Rn.50178
A_44_P373681	-0.51761	-2.92182	0.008317	-3.90066	BF563424	Rn.9698
A_42_P544321	-0.52113	-2.9674	0.007505	-3.88139	AI136185	Rn.3287
A_44_P791696	-0.52155	-3.32216	0.003333	-3.73274	DV725758	Rn.49278
A_44_P493603	-0.52579	-2.94879	0.007827	-3.88926	DY472377	Rn.33045
A_44_P270700	-0.53738	-3.46727	0.002379	-3.67304	BG663460	Rn.164882
A_42_P521814	-0.53786	-2.85913	0.009573	-3.92721		
A_44_P640301	-0.54291	-3.88173	0.0009	-3.50773	XM_001065707	
A_42_P525317	-0.54563	-2.99904	0.006987	-3.86803	NM_053851	Rn.10739
A_44_P884444	-0.54608	-3.64086	0.001586	-3.60278	XM_343782	Rn.15602
A_44_P650550	-0.55803	-3.5501	0.001961	-3.63935	BF289404	Rn.40468
A_43_P11492	-0.55942	-3.43126	0.002587	-3.68778	NM_012629	Rn.9759
A_42_P839655	-0.5619	-3.35173	0.003112	-3.72052	BF555716	Rn.15193

A_44_P443657	-0.56411	-2.93788	0.008022	-3.89387	BI281988	Rn.199201
A_44_P655666	-0.57623	-3.27079	0.003753	-3.75406		
A_42_P503281	-0.57732	-3.39551	0.002812	-3.70247	NM_053603	Rn.1838
A_43_P12283	-0.57902	-3.20147	0.004402	-3.78296	NM_022668	Rn.9687
A_44_P884766	-0.5965	-4.26186	0.000367	-3.36441		
A_44_P702115	-0.60186	-3.32954	0.003276	-3.72969		
A_44_P283909	-0.60955	-2.90764	0.008587	-3.90667	XM_216079	
A_44_P175677	-0.6183	-3.65131	0.001548	-3.59859	XM_001062365	Rn.155482
A_44_P386633	-0.63065	-2.9648	0.00755	-3.88249	NM_145785	Rn.17383
A_44_P956310	-0.63105	-3.11704	0.005342	-3.81833	AI171999	Rn.4169
A_42_P621736	-0.6312	-3.09601	0.005605	-3.82716	NM_001007604	Rn.973
A_44_P469517	-0.63176	-3.08126	0.005796	-3.83337	BF551572	Rn.18432
A_44_P605123	-0.64472	-3.07388	0.005895	-3.83647	DV716579	Rn.164403
A_44_P770724	-0.65145	-3.40431	0.002755	-3.69885	XM_225941	Rn.8048
A_44_P1017996	-0.66361	-2.97547	0.00737	-3.87798	XM_341470	Rn.162375
A_44_P350074	-0.6662	-3.14932	0.004962	-3.80478	XM_001075009	Rn.202425
A_44_P914438	-0.66865	-2.879	0.009157	-3.91879	NM_080782	Rn.10089
A_44_P593071	-0.67445	-3.31712	0.003372	-3.73483		
A_42_P575140	-0.68397	-2.88046	0.009127	-3.91817	AW916021	Rn.162877
A_44_P652899	-0.70319	-3.19122	0.004507	-3.78724	NM_178105	Rn.34370
A_44_P793157	-0.71169	-3.10325	0.005513	-3.82412		
A_44_P416127	-0.71382	-3.53588	0.002027	-3.64511	BG665128	Rn.64518
A_44_P123857	-0.72997	-3.04958	0.00623	-3.8467	XM_227855	
A_44_P576019	-0.73169	-2.88149	0.009106	-3.91774		
A_44_P556677	-0.74267	-3.20897	0.004327	-3.77982	AW525326	Rn.6795
A_44_P531685	-0.77481	-3.42948	0.002598	-3.68851	NM_212511	Rn.3458
A_42_P484738	-0.77772	-2.87551	0.009229	-3.92027	NM_022266	Rn.17145
A_44_P976667	-0.77806	-3.4241	0.002631	-3.69072		
A_44_P575265	-0.78268	-3.13828	0.005089	-3.80941	XM_001059898	
A_44_P341298	-0.80182	-3.62365	0.001651	-3.60968	BF396386	Rn.172843
A_44_P684912	-0.86822	-3.22523	0.004168	-3.77303		
A_44_P344475	-0.87182	-2.98551	0.007204	-3.87374	NM_053522	Rn.4169
A_44_P608966	-0.89897	-3.44321	0.002516	-3.68289		
A_42_P783113	-0.94068	-2.9189	0.008372	-3.9019	XM_215044	Rn.1318
A_44_P728524	-0.94897	-3.15801	0.004864	-3.80114	XM_218037	Rn.176450
A_44_P353618	-1.59827	-3.06343	0.006037	-3.84087	NM_053587	Rn.6703
A_44_P1055780	-1.79949	-3.46899	0.00237	-3.67234	NM_053822	Rn.31839

Supplementary Table 2

ID	logFC	t	P.Value	B	GenbankAccession	UniGeneID
A_44_P255954	1.092079	3.230865	0.004114	-2.5529	XM_573223	Rn.21595
A_43_P16512	0.958391	3.096458	0.005599	-2.7098	XM_218824	Rn.3618
A_44_P199211	0.888268	2.998223	0.007	-2.82425	NM_001077641	Rn.45523
A_44_P519174	0.865479	3.663251	0.001505	-2.05	NM_001015027	Rn.107553
A_44_P797926	0.827698	3.314413	0.003393	-2.45534	BG673275	Rn.199501
A_44_P638014	0.786066	3.626017	0.001642	-2.09301	XM_001067431	Rn.12385
A_44_P253196	0.72852	4.218567	0.000407	-1.42152	XM_228114	Rn.204911
A_44_P817120	0.705276	4.211694	0.000413	-1.42911	BF563826	
A_44_P764308	0.70371	3.087846	0.00571	-2.71985		
A_44_P515211	0.702895	4.051445	0.000603	-1.60766	NM_145765	Rn.84873
A_44_P405203	0.692478	3.814967	0.001054	-1.87569	XM_218664	Rn.106778
A_44_P314492	0.692285	3.187177	0.004549	-2.60392	XM_221672	Rn.204561
A_44_P108421	0.689739	3.064999	0.006015	-2.74648	XM_224350	Rn.100627
A_44_P274573	0.681768	3.987864	0.000701	-1.67923	XM_232283	Rn.21111
A_44_P525431	0.678326	3.564303	0.001897	-2.16446		
A_43_P11622	0.670731	2.842532	0.009935	-3.00478	NM_012925	Rn.1231
A_44_P1050433	0.669816	2.936844	0.008041	-2.89557	XM_001078552	Rn.47597
A_43_P19994	0.651395	3.233036	0.004094	-2.55036	XM_233485	Rn.50631
A_44_P473472	0.649492	3.317094	0.003372	-2.45221	BC091233	Rn.9497
A_44_P168514	0.64246	3.565117	0.001894	-2.16352	XM_226964	Rn.133363
A_44_P377645	0.63785	3.95178	0.000763	-1.72002	XM_342774	
A_44_P300612	0.625149	2.933283	0.008106	-2.89971	NM_022850	Rn.10076
A_43_P17175	0.624011	3.800738	0.001089	-1.89197	NM_001031642	Rn.103402
A_43_P17180	0.623162	3.454849	0.002449	-2.29162	NM_001007696	Rn.763
A_44_P323430	0.620476	2.920378	0.008344	-2.91468	NM_138532	Rn.82718
A_44_P422271	0.600331	3.190954	0.00451	-2.59951	XM_217019	Rn.99537
A_44_P525645	0.592939	2.910561	0.008531	-2.92606	XM_220442	Rn.214179
A_44_P175495	0.59217	3.261685	0.003832	-2.5169	NM_182952	Rn.13664
A_44_P163242	0.587393	3.411361	0.00271	-2.34227	NM_138541	Rn.106481
A_44_P102291	0.58478	3.78593	0.001128	-1.90893	XM_341619	Rn.47386
A_43_P10020	0.579306	2.8681	0.009383	-2.97523	NM_012771	Rn.2283
A_44_P522492	0.577851	3.513423	0.002137	-2.22351	NM_133414	Rn.26586
A_44_P348973	0.574847	3.048348	0.006247	-2.76589	NM_001004277	Rn.93631
A_44_P160846	0.574796	3.871095	0.000923	-1.81164	NM_182673	Rn.107928
A_44_P1027904	0.574744	3.365231	0.003016	-2.39605	XM_213794	Rn.41651
A_44_P269425	0.57387	3.079186	0.005824	-2.72994	NM_133415	Rn.62703
A_43_P17880	0.572611	3.261621	0.003833	-2.51698	NM_182953	Rn.18744
A_44_P440633	0.569351	2.950357	0.0078	-2.87989	NM_031639	Rn.10238
A_44_P255236	0.561685	3.034627	0.006445	-2.78187	NM_019212	Rn.82732
A_44_P469113	0.561279	3.188915	0.004531	-2.60189	XM_220779	Rn.105052
A_42_P744464	0.559015	3.008124	0.006845	-2.81273	XM_340740	Rn.98642
A_44_P247219	0.558874	3.723233	0.001307	-1.9809	NM_053699	Rn.40380
A_42_P453976	0.554018	3.971332	0.000729	-1.6979	XM_219476	Rn.136568
A_44_P191022	0.553331	3.031764	0.006487	-2.78521	NM_053674	Rn.7279
A_44_P492629	0.552641	3.351295	0.003115	-2.4123	DV727624	Rn.203850
A_43_P10824	0.546754	3.722453	0.00131	-1.9818	NM_001015010	Rn.46320
A_43_P18186	0.539919	2.926248	0.008235	-2.90787	XM_232226	Rn.3111
A_44_P408520	0.539577	3.024085	0.006601	-2.79415	NM_031569	Rn.9762
A_44_P189813	0.539428	3.045752	0.006284	-2.76891	XM_214295	Rn.105830
A_44_P150471	0.535326	2.924382	0.00827	-2.91003	NM_031696	Rn.59513
A_43_P12802	0.5329	3.719108	0.00132	-1.98564	NM_053418	Rn.11946
A_44_P445369	0.532457	3.217287	0.004245	-2.56875	XM_001080476	Rn.178658
A_44_P114184	0.525917	3.306995	0.003452	-2.464	NM_022543	Rn.2193
A_44_P134634	0.519467	2.897277	0.008789	-2.94145	CB576476	Rn.14073
A_44_P309523	0.516512	2.96465	0.007552	-2.86328	XM_001076269	Rn.196791
A_44_P273449	0.514881	2.929609	0.008173	-2.90397	XM_214953	Rn.101762

A_44_P823303	0.512983	3.234316	0.004082	-2.54887		
A_44_P729266	0.503142	3.733255	0.001277	-1.96938	XR_005718	Rn.216190
A_43_P18294	0.500507	3.256681	0.003877	-2.52275	BC099835	Rn.134218
A_44_P314353	0.499854	2.919057	0.008369	-2.91621	NM_001037765	Rn.144447
A_44_P288241	0.496253	3.022922	0.006619	-2.7955	XM_341029	Rn.14770
A_44_P167408	0.492468	2.942735	0.007935	-2.88874	AW144014	Rn.13589
A_44_P229051	0.492248	3.13049	0.00518	-2.67009	NM_012748	Rn.10623
A_44_P668388	0.490809	3.228828	0.004134	-2.55528	BC098022	Rn.117989
A_44_P408108	0.475979	2.910473	0.008532	-2.92616	XM_236649	Rn.73847
A_44_P189953	0.471873	2.92019	0.008348	-2.9149	NM_031769	Rn.18815
A_44_P549028	0.471493	3.14955	0.004959	-2.64785	XM_001076082	Rn.207473
A_42_P839541	0.466727	2.906207	0.008615	-2.93111	NM_001007671	Rn.144924
A_44_P252756	0.466033	2.919283	0.008365	-2.91595	XM_221874	Rn.6774
A_44_P655158	0.462016	3.331279	0.003263	-2.43566	CO567276	Rn.203592
A_44_P426624	0.46039	3.393368	0.002826	-2.36324	XM_344380	
A_43_P23215	0.456773	3.575282	0.001849	-2.15174	NM_001025737	Rn.205854
A_44_P299123	0.452931	3.005041	0.006893	-2.81632	XM_215847	Rn.162718
A_44_P456747	0.438792	3.137424	0.005099	-2.662	AB012231	Rn.40435
A_44_P237220	0.438141	3.246938	0.003965	-2.53413	XM_214720	Rn.98912
A_44_P148280	0.437942	2.943752	0.007917	-2.88756	NM_053670	Rn.9449
A_43_P10534	0.434336	3.152137	0.00493	-2.64483	NM_001034108	Rn.145219
A_44_P332817	0.430724	2.920255	0.008347	-2.91482	NM_053527	Rn.54977
A_43_P20794	0.418891	2.940595	0.007973	-2.89122	BC091326	Rn.140527
A_44_P483177	0.416901	3.015735	0.006728	-2.80387	NM_001009344	Rn.129282
A_43_P19369	0.414985	2.928171	0.008199	-2.90564	XM_219805	Rn.204724
A_44_P545649	0.410999	3.632987	0.001615	-2.08495	NM_001014093	Rn.23423
A_44_P111825	0.410741	3.00833	0.006841	-2.81249	NM_001014144	Rn.16893
A_44_P1051765	0.405347	3.10861	0.005446	-2.69563	XM_342984	Rn.14849
A_44_P109834	0.401496	3.050681	0.006214	-2.76317	XM_341783	Rn.100654
A_44_P809514	0.398808	3.321156	0.00334	-2.44747		
A_44_P1038400	0.394146	3.141451	0.005052	-2.6573	DV727624	Rn.203850
A_43_P10315	0.393843	2.843644	0.009911	-3.00349	XM_341626	Rn.203753
A_44_P289173	0.390852	2.982177	0.007259	-2.84291	BC097985	Rn.19589
A_44_P187300	0.390448	2.923616	0.008284	-2.91092	XM_344202	
A_44_P228903	0.389952	3.779544	0.001145	-1.91625	NM_078621	Rn.212750
A_44_P532500	0.387401	2.920154	0.008349	-2.91494	XM_217354	Rn.207855
A_43_P12267	0.380794	3.335712	0.00323	-2.43048	NM_022623	Rn.48736
A_44_P424069	0.380306	3.0621	0.006055	-2.74986	XM_218463	Rn.213001
A_44_P370082	0.379629	3.098258	0.005576	-2.7077	XM_340856	Rn.41817
A_43_P15129	0.377676	2.871891	0.009304	-2.97084	BF558849	
A_44_P349817	0.373434	3.021743	0.006636	-2.79688	XM_237215	Rn.214104
A_44_P1010416	0.365499	3.311583	0.003415	-2.45865	NM_001002853	Rn.209780
A_44_P447927	0.362439	3.235128	0.004074	-2.54792		
A_44_P356482	0.359146	2.933233	0.008106	-2.89977		
A_44_P119442	0.353803	2.89369	0.00886	-2.94561	XM_216492	
A_44_P409539	0.350217	3.19752	0.004442	-2.59184	NM_175869	Rn.12945
A_44_P407800	0.350096	3.595319	0.001764	-2.12853	XM_225607	Rn.124742
A_43_P17282	0.349379	2.962993	0.00758	-2.86521	XM_219879	Rn.87733
A_42_P809101	0.345238	3.124555	0.005251	-2.67702	XM_342873	Rn.206137
A_44_P468816	0.340427	2.907558	0.008588	-2.92954	XM_001057103	Rn.7936
A_42_P601971	0.333952	3.051229	0.006207	-2.76253	XM_220328	Rn.163114
A_44_P398433	0.328459	2.88229	0.00909	-2.95881	XM_224923	Rn.206107
A_44_P156846	0.326852	2.843101	0.009923	-3.00412	XM_231139	Rn.211738
A_44_P370774	0.321406	2.997523	0.007011	-2.82506	XM_235183	Rn.161928
A_44_P260384	0.313235	2.936879	0.00804	-2.89553	XM_240915	Rn.64258
A_44_P388709	0.279068	2.946045	0.007876	-2.88489	NM_145767	Rn.10189
A_44_P591036	-0.31221	-2.92133	0.008327	-2.91357	XM_001069503	
A_43_P11492	-0.34147	-3.03134	0.006493	-2.7857	NM_012629	Rn.9759

A_44_P229557	-0.34405	-2.89908	0.008754	-2.93937	XM_230513	Rn.17049
A_44_P518050	-0.36419	-3.22135	0.004205	-2.564	XM_223566	Rn.24287
A_44_P184865	-0.37731	-2.9579	0.007668	-2.87112	NM_031579	Rn.9459
A_44_P1002407	-0.38311	-2.86879	0.009369	-2.97442	AI137378	Rn.166773
A_44_P334736	-0.38814	-3.37101	0.002976	-2.3893	NM_012548	Rn.10918
A_44_P839802	-0.38948	-2.91023	0.008537	-2.92644		
A_44_P307646	-0.39544	-2.96136	0.007608	-2.86711	BI289723	Rn.162620
A_44_P420046	-0.40913	-2.88381	0.009059	-2.95705	NM_021669	Rn.42103
A_42_P736812	-0.41247	-3.15582	0.004888	-2.64052	NM_134449	Rn.12281
A_44_P777113	-0.4134	-2.91736	0.008401	-2.91818	XM_001068152	
A_42_P840867	-0.41639	-3.13684	0.005105	-2.66268	XR_008037	Rn.3743
A_44_P597013	-0.42295	-3.01657	0.006715	-2.8029		
A_44_P288443	-0.42363	-3.19774	0.00444	-2.59158	XM_344542	Rn.100687
A_43_P13326	-0.42534	-3.27468	0.003719	-2.50173	NM_138894	Rn.82758
A_44_P110334	-0.42702	-3.23471	0.004078	-2.54841	XM_236387	Rn.162988
A_43_P23304	-0.42939	-3.2285	0.004137	-2.55566	NM_199397	Rn.204521
A_44_P428338	-0.43776	-3.84422	0.000983	-1.84228	AA817984	Rn.98260
A_44_P1050510	-0.44061	-2.94931	0.007818	-2.88111	XM_001075804	
A_44_P127962	-0.44219	-3.06498	0.006015	-2.74651	NM_181771	Rn.56673
A_44_P379645	-0.45022	-3.15771	0.004867	-2.63832	AF030090	Rn.202876
A_44_P578593	-0.4523	-3.18672	0.004554	-2.60444		
A_43_P18201	-0.45248	-2.98747	0.007172	-2.83676	XM_230639	Rn.104639
A_44_P283372	-0.45264	-3.16559	0.00478	-2.62912	BC062235	Rn.25771
A_44_P960490	-0.4539	-3.13199	0.005162	-2.66835	XM_573497	Rn.138943
A_44_P171321	-0.46049	-3.24816	0.003954	-2.5327	NM_001009671	Rn.101706
A_42_P737455	-0.46356	-3.21374	0.00428	-2.57289	AI071866	Rn.166480
A_44_P182739	-0.46677	-2.87175	0.009307	-2.971	XM_217651	Rn.43772
A_44_P853624	-0.46843	-3.06352	0.006035	-2.74821	XM_237855	Rn.29452
A_44_P650550	-0.47547	-3.02484	0.00659	-2.79327	BF289404	Rn.40468
A_44_P745585	-0.48381	-2.88277	0.00908	-2.95826	NM_001024309	Rn.116819
A_44_P376411	-0.4896	-2.94663	0.007865	-2.88422	BF548067	Rn.164650
A_44_P885461	-0.49151	-3.51716	0.002118	-2.21918		
A_44_P976153	-0.49246	-3.50989	0.002154	-2.22761		
A_44_P792746	-0.49464	-3.25277	0.003912	-2.52731		
A_43_P14760	-0.49524	-3.02068	0.006653	-2.79812		
A_44_P578847	-0.49731	-3.06915	0.005959	-2.74165		
A_44_P1031380	-0.49908	-2.85054	0.009759	-2.99552	XM_001077462	Rn.10092
A_44_P808218	-0.49984	-3.02304	0.006617	-2.79537	XM_233470	Rn.33652
A_44_P278659	-0.50967	-3.63278	0.001616	-2.08519	AJ517196	Rn.30616
A_44_P988767	-0.51023	-3.16631	0.004772	-2.62829	BF564195	Rn.10251
A_42_P475623	-0.51931	-3.12774	0.005213	-2.67331	NM_053394	Rn.8954
A_44_P653818	-0.5195	-3.42999	0.002595	-2.32057		
A_44_P254238	-0.51954	-3.07077	0.005937	-2.73976	NM_001033670	Rn.10323
A_44_P635089	-0.5201	-3.98677	0.000703	-1.68046	AI030078	Rn.165903
A_44_P724632	-0.52274	-3.03083	0.006501	-2.78629	AI176008	
A_44_P1026631	-0.52356	-2.91813	0.008387	-2.91729	XM_573823	Rn.10557
A_44_P840348	-0.53073	-2.91847	0.00838	-2.91689		
A_44_P559242	-0.53267	-3.18308	0.004592	-2.6087	XM_001065920	Rn.40465
A_42_P570848	-0.54445	-4.19397	0.000431	-1.44873	NM_017091	Rn.11384
A_44_P175677	-0.5551	-3.2781	0.00369	-2.49774	XM_001062365	Rn.155482
A_42_P788810	-0.56244	-2.88838	0.008966	-2.95176	AW918709	Rn.203224
A_44_P837846	-0.57464	-3.10858	0.005446	-2.69565	DV723320	Rn.106829
A_44_P895064	-0.57601	-2.89321	0.00887	-2.94617	XM_341290	Rn.140732
A_44_P570046	-0.57765	-2.98033	0.007289	-2.84505	XM_001067059	Rn.201930
A_42_P502759	-0.58777	-3.09092	0.00567	-2.71626	XM_343513	Rn.1423
A_44_P249774	-0.59498	-2.84342	0.009915	-3.00375	BG668003	Rn.201724
A_44_P731912	-0.60161	-3.01	0.006816	-2.81054		
A_42_P638620	-0.60852	-3.33924	0.003204	-2.42637	NM_130741	Rn.11303

A_44_P181718	-0.61556	-3.35125	0.003116	-2.41235	BI285476	Rn.6870
A_44_P521587	-0.61685	-3.84583	0.00098	-1.84043	XM_224588	Rn.11687
A_44_P374708	-0.61932	-2.91216	0.0085	-2.92421	NM_198750	Rn.43646
A_44_P962074	-0.63275	-3.03238	0.006478	-2.78449		
A_44_P551872	-0.63783	-2.84708	0.009835	-2.99952	AI555745	Rn.17145
A_42_P556083	-0.64137	-2.87961	0.009144	-2.96191	NM_013165	Rn.90997
A_44_P492624	-0.64313	-4.21612	0.000409	-1.42422	XM_340867	
A_44_P472989	-0.64342	-3.30579	0.003461	-2.46541	NM_017232	Rn.44369
A_44_P199107	-0.65155	-3.33422	0.003241	-2.43223	XM_345241	
A_44_P341298	-0.65452	-2.95796	0.007667	-2.87105	BF396386	Rn.172843
A_43_P12535	-0.65537	-2.8748	0.009243	-2.96747	NM_031135	Rn.2398
A_43_P14919	-0.66064	-3.51746	0.002117	-2.21883	NM_019372	Rn.31799
A_43_P12283	-0.66706	-3.68827	0.001419	-2.02115	NM_022668	Rn.9687
A_44_P776423	-0.67176	-3.64281	0.001579	-2.07361	NM_001017386	Rn.25053
A_44_P559239	-0.6929	-3.16442	0.004793	-2.63049	AW921223	Rn.40465
A_44_P1034541	-0.69456	-3.66028	0.001515	-2.05343	NM_022602	Rn.6343
A_44_P254130	-0.70132	-4.05855	0.000593	-1.59969	AA875032	
A_44_P337351	-0.70754	-4.29411	0.00034	-1.33838	NM_001033883	Rn.54439
A_44_P593071	-0.70957	-3.48989	0.002257	-2.25086		
A_44_P221885	-0.71164	-3.13697	0.005104	-2.66253	BF565038	Rn.156229
A_44_P589346	-0.72373	-3.25028	0.003934	-2.53022	DY471743	Rn.11709
A_42_P762508	-0.75728	-3.89528	0.000872	-1.78412	NM_001034199	Rn.162101
A_44_P670185	-0.75886	-3.50289	0.00219	-2.23575		
A_44_P823528	-0.76063	-4.08677	0.000555	-1.56807		
A_42_P681722	-0.76759	-3.7704	0.00117	-1.92673	BF554576	Rn.165233
A_44_P870183	-0.77114	-2.95087	0.007791	-2.87929		
A_42_P739860	-0.77204	-3.06879	0.005963	-2.74206	NM_133578	Rn.10877
A_44_P914438	-0.77853	-3.35211	0.003109	-2.41135	NM_080782	Rn.10089
A_44_P219026	-0.78547	-3.50001	0.002205	-2.2391	NM_012513	Rn.11266
A_44_P732510	-0.80447	-2.92646	0.008231	-2.90762	DV722942	Rn.173292
A_44_P307971	-0.82442	-3.16637	0.004772	-2.62821	L04739	
A_44_P807565	-0.83069	-3.50917	0.002158	-2.22845		
A_44_P1029253	-0.91482	-3.372	0.002969	-2.38815	NM_012757	Rn.21943
A_44_P378959	-0.93302	-6.04924	5.98E-06	0.369974	AA859348	
A_44_P763434	-0.94522	-3.10133	0.005537	-2.70412	XM_576579	Rn.141605
A_44_P437896	-0.95369	-5.74915	1.17E-05	0.110652	NM_012513	Rn.11266
A_44_P303430	-0.96464	-6.09195	5.44E-06	0.405763	AW142654	
A_44_P916210	-0.99037	-3.72701	0.001296	-1.97655		
A_44_P522524	-0.9989	-4.12394	0.000509	-1.52655	NM_019328	Rn.88129
A_44_P931389	-1.00677	-3.38108	0.002907	-2.37757	CA509267	Rn.179170
A_44_P1032732	-1.01013	-3.01546	0.006732	-2.80419	NM_031740	Rn.21126
A_42_P839593	-1.01732	-3.45972	0.002422	-2.28595	AW142999	Rn.35842
A_44_P424723	-1.05196	-3.08651	0.005727	-2.7214	NM_021693	Rn.42905
A_42_P484738	-1.14331	-4.22725	0.000399	-1.41193	NM_022266	Rn.17145
A_44_P512806	-1.18274	-3.94126	0.000782	-1.73193	NM_153626	Rn.86654
A_44_P654444	-1.18541	-5.88852	8.56E-06	0.232804	DQ268830	Rn.62694
A_44_P365379	-1.25143	-3.85157	0.000967	-1.83389	NM_001077640	Rn.16950
A_42_P594613	-1.2986	-4.31822	0.000322	-1.31199	NM_053883	Rn.4313
A_42_P540950	-1.31126	-3.1894	0.004526	-2.60132	NM_001009541	Rn.4298
A_44_P233080	-1.32317	-4.82589	9.76E-05	-0.77345	NM_012551	Rn.9096
A_44_P701809	-1.3923	-4.12159	0.000511	-1.52918		
A_44_P591857	-1.3938	-4.08569	0.000557	-1.56928		
A_42_P708068	-1.41456	-5.09071	5.27E-05	-0.50674	NM_019137	Rn.31998
A_44_P560710	-1.44987	-4.27919	0.000353	-1.35475		
A_44_P495480	-1.50825	-3.6907	0.001411	-2.01835	NM_022197	Rn.103750
A_43_P12619	-1.58827	-4.16732	0.000459	-1.47828	NM_031628	Rn.62694
A_44_P212803	-1.61214	-3.42414	0.002631	-2.32738	AF050659	Rn.202875
A_43_P12125	-1.61511	-3.31149	0.003416	-2.45876	NM_021836	Rn.15806

A_44_P218896	-1.65862	-3.03286	0.006471	-2.78393	NM_031327	Rn.22129
A_44_P398142	-1.69025	-3.50522	0.002178	-2.23305	NM_017259	Rn.27923
A_44_P380835	-1.72043	-5.81913	1.00E-05	0.172355	AB003726	Rn.37500
A_44_P839581	-1.75977	-4.10076	0.000537	-1.55243		
A_42_P682589	-1.88194	-4.39681	0.000267	-1.22644	NM_024388	Rn.10000
A_43_P11932	-2.06197	-4.10425	0.000533	-1.54854	NM_017352	Rn.62694
A_42_P750683	-2.09602	-3.96396	0.000742	-1.70624	NM_031327	Rn.22129
A_44_P118724	-2.15949	-5.13461	4.76E-05	-0.46353	NM_019361	Rn.10086
A_42_P473594	-2.43313	-6.27434	3.64E-06	0.555501	NM_053633	Rn.89235

Supplementary Table 3

ID	logFC	t	P.Value	B	GenbankAccession	UniGeneID
A_44_P839581	1.478474	3.445262	0.002504	-1.44321		
A_44_P157527	1.190031	3.95436	0.000759	-0.44392	BF285469	Rn.45224
A_44_P535706	1.064651	2.904313	0.008651	-2.47862	XM_216485	Rn.8332
A_42_P525886	1.056686	3.238891	0.004039	-1.84319	XM_236253	Rn.3207
A_44_P262625	0.984094	3.37229	0.002967	-1.58517	XM_344424	
A_44_P217250	0.951383	3.060295	0.00608	-2.18482	NM_012949	Rn.3443
A_44_P478637	0.946374	4.507965	0.000206	0.641561	BI274236	Rn.161389
A_42_P679251	0.920972	3.361842	0.00304	-1.60545	NM_031503	Rn.10486
A_44_P931389	0.919886	3.089285	0.005691	-2.12971	CA509267	Rn.179170
A_44_P913689	0.915771	3.324394	0.003316	-1.67805		
A_43_P16887	0.914468	3.027699	0.006547	-2.2466	XM_215059	Rn.136569
A_44_P479890	0.912017	3.880277	0.000903	-0.5898	NM_153821	Rn.203584
A_42_P793008	0.908714	2.954982	0.007719	-2.38371	NM_130432	Rn.198625
A_44_P231657	0.907535	3.86021	0.000947	-0.62931	XM_341779	Rn.140257
A_43_P14752	0.898158	4.12512	0.000507	-0.10785	XM_001073940	Rn.9542
A_44_P236566	0.897017	3.861663	0.000944	-0.62644	XM_230848	Rn.98792
A_44_P671992	0.894022	5.227009	3.85E-05	2.015107	XM_575387	Rn.208326
A_43_P16949	0.846619	3.038804	0.006384	-2.22557	XM_220884	Rn.136653
A_43_P11563	0.835843	4.47932	0.00022	0.585794	NM_012784	Rn.89774
A_44_P685508	0.821372	3.105956	0.005479	-2.09796		
A_44_P743576	0.820139	3.633806	0.001612	-1.07444	AW917533	Rn.2295
A_44_P1009948	0.806853	3.521605	0.002096	-1.29419	XM_220532	Rn.21630
A_44_P252855	0.803766	4.172639	0.000453	-0.01445		
A_44_P853254	0.800247	3.10884	0.005443	-2.09246		
A_44_P197290	0.792545	3.741201	0.001253	-0.86348	NM_021760	Rn.38654
A_44_P588759	0.792232	4.302654	0.000334	0.240641	BF563670	Rn.147739
A_44_P543813	0.776085	3.112261	0.005401	-2.08593	XM_228345	Rn.24209
A_44_P974651	0.774859	4.080057	0.000564	-0.19648	XM_575260	Rn.141393
A_42_P635881	0.773147	4.161272	0.000466	-0.03679	NM_031782	Rn.10846
A_44_P558838	0.769348	2.98332	0.00724	-2.3304	BF417038	Rn.137099
A_44_P413324	0.763004	3.092474	0.00565	-2.12364	BF558477	Rn.166297
A_44_P307308	0.761818	2.908038	0.008579	-2.47166	XM_237568	
A_43_P11044	0.760943	3.30244	0.003488	-1.72054	NM_001009632	Rn.1040
A_44_P409913	0.750951	2.925367	0.008251	-2.43925	XM_341043	
A_44_P685354	0.747361	3.567133	0.001885	-1.20512		
A_42_P596942	0.746022	4.901028	8.19E-05	1.399454	NM_145682	Rn.44931
A_42_P568943	0.738116	2.940044	0.007983	-2.41175	XM_217167	Rn.163007
A_44_P592800	0.732669	3.730891	0.001284	-0.88376		
A_44_P233786	0.73062	3.470434	0.002362	-1.39413	NM_030997	Rn.9704
A_44_P461587	0.727787	4.048997	0.000607	-0.2576	XM_341847	Rn.82375
A_44_P808072	0.727337	2.977696	0.007333	-2.341		
A_43_P11082	0.723487	3.362518	0.003035	-1.60414		
A_44_P304759	0.722884	3.252704	0.003913	-1.81658	XR_007322	Rn.196027
A_44_P670185	0.715761	3.303958	0.003476	-1.7176		
A_42_P814235	0.715392	2.859275	0.00957	-2.56252	NM_057124	Rn.10671
A_44_P848951	0.708819	3.488979	0.002262	-1.35794	AW915522	Rn.128940
A_44_P222447	0.706019	3.233068	0.004094	-1.85441	CF979076	Rn.101945
A_44_P319238	0.705051	3.356706	0.003076	-1.61542	XM_345977	Rn.124862
A_44_P589674	0.702823	3.376922	0.002936	-1.57618	AI406628	Rn.170660
A_44_P394279	0.701716	2.905192	0.008634	-2.47698	XM_001081777	Rn.143966
A_43_P19039	0.699688	3.834718	0.001006	-0.67949	XM_342487	Rn.18453
A_44_P1014527	0.698386	2.986488	0.007188	-2.32443	XM_216189	Rn.211939
A_44_P1000100	0.69182	4.700938	0.000131	1.015486	XM_215666	Rn.102590
A_44_P117216	0.691555	3.204203	0.004375	-1.90992	NM_001025007	Rn.159189
A_44_P559239	0.69093	3.15544	0.004893	-2.00343	AW921223	Rn.40465
A_44_P620208	0.688642	3.710377	0.001347	-0.92408	BF549419	Rn.204134

A_44_P868418	0.683253	3.43641	0.002557	-1.46046	AI043758	Rn.165978
A_44_P748361	0.683244	2.875105	0.009237	-2.53308		
A_42_P797638	0.677852	3.672912	0.001471	-0.99768	XM_341839	Rn.137669
A_43_P12401	0.677561	3.051365	0.006205	-2.20176	NM_024142	Rn.160717
A_42_P596050	0.674306	3.148338	0.004973	-2.01702	NM_053572	Rn.19780
A_44_P435158	0.674159	3.198819	0.004429	-1.92026	AW917664	
A_44_P930681	0.673171	3.264009	0.003812	-1.79478	DV723298	Rn.96324
A_44_P882551	0.671501	2.962127	0.007595	-2.37029	XM_001072622	Rn.12980
A_42_P782490	0.669773	3.097574	0.005585	-2.11393	XM_226464	Rn.204355
A_44_P640931	0.668623	3.788517	0.001121	-0.77042		
A_44_P1049275	0.666814	2.858297	0.009591	-2.56433	DQ889715	Rn.9039
A_44_P272253	0.66654	3.546667	0.001977	-1.24518	XM_213864	Rn.32209
A_44_P507773	0.661387	2.842171	0.009943	-2.59426	BG663014	Rn.8166
A_44_P971665	0.659826	3.270928	0.003752	-1.78143	AW916050	Rn.1821
A_43_P11476	0.656787	3.243832	0.003993	-1.83368	NM_012590	Rn.8831
A_44_P668572	0.65317	3.27028	0.003757	-1.78268		
A_44_P900576	0.653048	3.045666	0.006286	-2.21257		
A_44_P996952	0.653002	3.745901	0.001239	-0.85424	NM_001039454	Rn.50178
A_44_P376112	0.652891	3.074973	0.00588	-2.15693	X72758	
A_44_P178503	0.652686	2.946974	0.007859	-2.39875	XM_221452	Rn.140653
A_44_P1034937	0.650992	3.03071	0.006503	-2.2409	NM_001011966	Rn.20043
A_44_P296473	0.648189	3.262342	0.003827	-1.79799	AI013718	
A_44_P623694	0.648094	3.324128	0.003318	-1.67857	XM_001067023	Rn.153703
A_44_P358028	0.646793	2.843403	0.009916	-2.59198	CO569648	Rn.199091
A_44_P179338	0.646712	2.955214	0.007715	-2.38328	AI603168	Rn.3391
A_42_P740209	0.643911	4.146421	0.000482	-0.06598	NM_012601	Rn.11241
A_44_P669323	0.641396	3.324258	0.003317	-1.67832		
A_44_P500295	0.639834	3.132836	0.005152	-2.04666	BI274118	Rn.11495
A_42_P619403	0.638018	3.591535	0.00178	-1.15732	XM_341720	Rn.66325
A_44_P316898	0.636016	3.520201	0.002103	-1.29694	XM_213488	Rn.38891
A_44_P324906	0.635202	3.198998	0.004427	-1.91992	AW142380	Rn.161942
A_43_P11634	0.633438	3.160983	0.004831	-1.99282	NM_012959	Rn.88489
A_44_P753353	0.633317	2.936406	0.008049	-2.41857	CO401859	Rn.203889
A_44_P464065	0.632653	2.922288	0.008309	-2.44501	NM_001033687	Rn.107429
A_44_P729931	0.632187	3.614977	0.001685	-1.11137	XM_001061367	
A_44_P808566	0.631451	3.321584	0.003337	-1.6835		
A_43_P11350	0.629225	3.045993	0.006281	-2.21195	NM_001009271	Rn.2956
A_44_P637675	0.627415	3.168738	0.004746	-1.97797		
A_44_P1024315	0.625645	2.99959	0.006978	-2.29972	NM_031321	Rn.12298
A_42_P560984	0.625403	4.167745	0.000459	-0.02407	XM_574891	Rn.145579
A_44_P1048140	0.620488	4.308368	0.000329	0.251834	AI072036	Rn.139210
A_43_P12205	0.619793	2.980849	0.007281	-2.33506	NM_022294	Rn.8731
A_42_P558206	0.618407	3.089728	0.005686	-2.12887	AW144330	Rn.204841
A_44_P513232	0.618325	3.342207	0.003182	-1.64354	BC099181	Rn.18207
A_44_P747073	0.618093	3.38739	0.002865	-1.55584		
A_43_P11654	0.616996	3.297049	0.003532	-1.73097	NM_013007	Rn.87935
A_44_P1007449	0.6148	3.201161	0.004405	-1.91577	XM_222190	Rn.13452
A_44_P1026706	0.612765	3.280038	0.003673	-1.76384	XM_001061644	
A_44_P900390	0.611891	3.947031	0.000772	-0.45835		
A_42_P735417	0.610726	3.286718	0.003617	-1.75093	NM_175756	Rn.33323
A_44_P284079	0.610166	3.140546	0.005062	-2.03192		
A_44_P839432	0.609938	3.301155	0.003499	-1.72303		
A_44_P747182	0.60653	3.131454	0.005169	-2.0493		
A_44_P376411	0.606227	3.648519	0.001558	-1.04557	BF548067	Rn.164650
A_44_P559242	0.605856	3.620395	0.001664	-1.10074	XM_001065920	Rn.40465
A_42_P694208	0.602707	2.994447	0.00706	-2.30943	XM_216483	Rn.24241
A_43_P17094	0.602556	3.07041	0.005941	-2.16561	XM_575803	Rn.6325
A_44_P977637	0.599374	3.82937	0.001018	-0.69002		

A_43_P10036	0.598834	3.209102	0.004326	-1.90051		
A_43_P11441	0.598154	3.01763	0.006699	-2.26565	NM_012510	Rn.10033
A_44_P113907	0.59688	2.940465	0.007975	-2.41096	NM_170787	Rn.213719
A_42_P620042	0.594377	2.873178	0.009277	-2.53667	NM_032080	Rn.10426
A_43_P14264	0.593121	3.283711	0.003642	-1.75674	NM_053653	Rn.6913
A_44_P311963	0.588438	3.808942	0.001069	-0.73022	XM_575939	Rn.98341
A_44_P420046	0.586908	4.136951	0.000493	-0.08459	NM_021669	Rn.42103
A_44_P653264	0.586072	3.147643	0.004981	-2.01835		
A_44_P408050	0.585517	2.966132	0.007527	-2.36276	XM_343189	Rn.99508
A_44_P200522	0.584708	3.040838	0.006355	-2.22172	AI171232	Rn.96234
A_44_P404737	0.584359	3.045231	0.006292	-2.21339	BQ208553	Rn.205491
A_44_P1031380	0.584111	3.336205	0.003226	-1.65517	XM_001077462	Rn.10092
A_43_P22042	0.583503	2.870099	0.009341	-2.5424	NM_001009353	Rn.90768
A_43_P17061	0.582069	3.854289	0.00096	-0.64096	XM_223729	Rn.32563
A_44_P746478	0.581686	2.84888	0.009795	-2.58182		
A_42_P829349	0.580869	3.811434	0.001062	-0.72532	XM_223785	Rn.7399
A_44_P1017035	0.580629	3.300734	0.003502	-1.72384	NM_019214	Rn.11792
A_44_P250575	0.580384	2.882513	0.009085	-2.51929	XM_214236	Rn.144747
A_43_P13381	0.577117	3.578162	0.001837	-1.18352	NM_139326	Rn.108195
A_44_P1026631	0.577082	3.216417	0.004254	-1.88645	XM_573823	Rn.10557
A_44_P255837	0.576706	3.225213	0.004168	-1.86953	XR_007349	Rn.195887
A_44_P215917	0.573202	2.88599	0.009015	-2.51281	XM_344018	Rn.198266
A_44_P686665	0.566384	2.962935	0.007581	-2.36877		
A_44_P1036170	0.565807	3.011334	0.006795	-2.27755	XM_220770	Rn.19087
A_44_P434118	0.565666	2.983611	0.007235	-2.32985	XM_213408	Rn.207605
A_42_P695770	0.565462	3.938881	0.000787	-0.4744	XM_214288	Rn.98475
A_42_P686756	0.564589	2.924454	0.008268	-2.44096	BC087706	Rn.172334
A_42_P781585	0.56446	3.024954	0.006588	-2.2518	NM_001037554	Rn.9287
A_44_P1027971	0.564132	3.430071	0.002595	-1.47281	XM_001068081	
A_43_P15659	0.563997	4.011897	0.000662	-0.33063	XM_342062	
A_44_P457599	0.563541	3.265752	0.003797	-1.79141	NM_053832	Rn.202954
A_43_P17945	0.56207	3.01518	0.006736	-2.27028	NM_001014006	Rn.53943
A_43_P18226	0.561037	2.877533	0.009187	-2.52856	XM_234942	Rn.198774
A_44_P956958	0.560997	3.634502	0.00161	-1.07307	AW143890	Rn.19306
A_42_P747311	0.560893	2.91521	0.008442	-2.45826	NM_057204	Rn.54442
A_44_P533531	0.560063	3.364838	0.003019	-1.59964	AI406723	Rn.144389
A_44_P302265	0.558124	3.08554	0.00574	-2.13684	XM_001065833	Rn.47025
A_44_P1050510	0.55727	3.730183	0.001286	-0.88515	XM_001075804	
A_44_P302383	0.557114	3.05974	0.006088	-2.18587	XR_006725	Rn.156427
A_44_P273734	0.556905	3.263089	0.00382	-1.79655	BC090336	Rn.3576
A_44_P983188	0.555079	3.090441	0.005676	-2.12751	AA818818	Rn.119809
A_44_P128820	0.554639	4.004296	0.000674	-0.34559		
A_42_P454907	0.554121	3.699759	0.001381	-0.94494	NM_053644	Rn.208796
A_43_P14870	0.553953	2.890333	0.008927	-2.50471	NM_001013224	Rn.101356
A_42_P771373	0.553627	3.586543	0.001801	-1.1671	NM_012614	Rn.9714
A_44_P454065	0.552253	3.04399	0.00631	-2.21575	NM_019147	Rn.88804
A_44_P527154	0.552208	3.479596	0.002312	-1.37625	NM_001024282	Rn.196555
A_42_P550172	0.55146	2.925806	0.008243	-2.43843	XM_001072438	Rn.138353
A_44_P356679	0.550647	3.482498	0.002296	-1.37059	XM_342932	Rn.32671
A_44_P186883	0.550626	3.206527	0.004351	-1.90546	NM_173103	Rn.10339
A_44_P731439	0.550347	3.488114	0.002267	-1.35962		
A_44_P1020748	0.549987	3.451634	0.002468	-1.43079	XM_343405	Rn.29863
A_43_P19791	0.549438	2.95487	0.007721	-2.38392	XM_343063	Rn.145668
A_43_P13996	0.548706	2.983588	0.007236	-2.3299		
A_42_P515028	0.548351	2.962016	0.007597	-2.37049	XM_574162	Rn.24564
A_44_P527089	0.5474	3.215169	0.004266	-1.88885	M11597	Rn.90085
A_44_P117015	0.546888	2.845737	0.009864	-2.58765	XM_342664	Rn.18013
A_44_P454847	0.546823	3.520162	0.002103	-1.29701	XM_342460	Rn.108023

A_42_P502768	0.543328	2.873787	0.009264	-2.53553	XM_001065468	Rn.198509
A_44_P974137	0.541887	4.053499	0.0006	-0.24874	AI716115	Rn.100997
A_44_P354018	0.540368	3.113921	0.00538	-2.08277	XM_224779	
A_43_P16588	0.540157	3.339672	0.0032	-1.64845	XM_342414	Rn.16537
A_44_P993027	0.538938	2.847305	0.00983	-2.58474	NM_017060	Rn.11377
A_44_P186860	0.537831	2.972818	0.007414	-2.35018	NM_030867	Rn.8395
A_44_P779143	0.537821	3.223984	0.00418	-1.87189		
A_44_P468661	0.536414	3.330719	0.003267	-1.6658	XM_001067949	Rn.27139
A_42_P797965	0.535476	3.565821	0.00189	-1.20769	XM_341902	Rn.106344
A_44_P728951	0.534878	3.025448	0.006581	-2.25086	NM_031838	Rn.2115
A_44_P960935	0.5334	3.478224	0.002319	-1.37893		
A_44_P525649	0.533214	3.327552	0.003291	-1.67194		
A_44_P1020608	0.533079	2.941113	0.007964	-2.40974	XM_227821	Rn.43876
A_44_P115509	0.532461	3.365016	0.003018	-1.59929	CN542475	Rn.2509
A_44_P901878	0.531861	3.584054	0.001812	-1.17198		
A_44_P389590	0.530125	3.019892	0.006664	-2.26137	XM_236348	Rn.163026
A_44_P1034346	0.527902	3.415038	0.002687	-1.50207	NM_001013922	Rn.11832
A_44_P996729	0.524978	3.290193	0.003588	-1.74422	NM_031054	Rn.6422
A_44_P792646	0.524628	3.04204	0.006338	-2.21944		
A_44_P187641	0.524616	2.879368	0.009149	-2.52515	XM_342871	Rn.98798
A_42_P700089	0.52346	2.84146	0.009959	-2.59558	NM_001034157	Rn.203143
A_44_P791860	0.521772	3.60352	0.001731	-1.13383		
A_42_P501233	0.521734	3.27362	0.003728	-1.77623	NM_024483	Rn.11314
A_44_P592844	0.521615	4.054903	0.000598	-0.24598		
A_44_P159613	0.521193	3.000508	0.006964	-2.29799	XM_344763	Rn.3278
A_43_P14871	0.520827	3.495944	0.002226	-1.34433	NM_021587	Rn.40942
A_44_P792446	0.520423	2.880358	0.009129	-2.5233	XM_235529	Rn.8673
A_44_P189859	0.517844	3.025102	0.006586	-2.25152	NM_001012094	Rn.7867
A_43_P22700	0.517808	3.05666	0.00613	-2.19172		
A_42_P578953	0.517652	2.878098	0.009175	-2.52751	XM_342416	Rn.203694
A_44_P210736	0.51718	2.88403	0.009054	-2.51646	XR_007818	Rn.195982
A_44_P668620	0.516418	3.307904	0.003444	-1.70997	XM_340915	Rn.13165
A_42_P837214	0.516128	3.100087	0.005553	-2.10914	NM_012861	Rn.9836
A_44_P164848	0.514671	2.98784	0.007166	-2.32188	AW142951	
A_43_P10498	0.514272	3.733602	0.001276	-0.87843	XM_237039	
A_44_P421333	0.513769	2.997905	0.007005	-2.3029	NM_080778	Rn.17815
A_44_P122206	0.513365	3.247602	0.003959	-1.82641	NM_022541	Rn.3792
A_44_P133485	0.512323	3.347197	0.003145	-1.63386	XM_342883	Rn.87596
A_44_P337311	0.511511	3.788606	0.001121	-0.77024	XM_342172	Rn.11207
A_44_P670043	0.510834	2.949975	0.007806	-2.39312		
A_44_P1026123	0.509573	2.912482	0.008494	-2.46336	XM_341806	
A_44_P978083	0.507157	3.233427	0.00409	-1.85372		
A_42_P737455	0.506634	3.512384	0.002142	-1.31222	AI071866	Rn.166480
A_44_P607632	0.505455	3.171842	0.004712	-1.97202	XM_001079243	Rn.17349
A_44_P412236	0.505256	3.225278	0.004168	-1.8694	NM_053730	Rn.18997
A_44_P976153	0.504629	3.596657	0.001759	-1.14728		
A_44_P853624	0.503794	3.294774	0.003551	-1.73536	XM_237855	Rn.29452
A_44_P370645	0.503213	2.952194	0.007767	-2.38895	XM_216400	Rn.103030
A_44_P561643	0.502716	2.889284	0.008948	-2.50667		
A_44_P274083	0.502042	2.968587	0.007485	-2.35814	NM_173105	Rn.20144
A_44_P263854	0.500949	3.539401	0.002011	-1.25939	AA819299	Rn.35934
A_44_P997787	0.499178	3.545056	0.001984	-1.24833	XM_213799	Rn.13057
A_44_P215056	0.49916	3.352026	0.00311	-1.6245	NM_001007235	Rn.2135
A_42_P621832	0.498006	3.413777	0.002695	-1.50453	XM_215083	Rn.61660
A_43_P12437	0.496721	2.897275	0.008789	-2.49176	NM_024385	Rn.12188
A_44_P513360	0.494845	3.886942	0.000889	-0.57667	XM_214152	Rn.107220
A_43_P14954	0.493343	3.238154	0.004046	-1.84462	XM_233820	Rn.91844
A_44_P447701	0.492105	2.893343	0.008867	-2.4991		

A_43_P19306	0.491696	2.901791	0.0087	-2.48333	XM_223660	Rn.6266
A_43_P19265	0.490977	2.846506	0.009847	-2.58622	XM_342507	Rn.205773
A_44_P330109	0.490805	3.190897	0.00451	-1.93547	NM_040669	Rn.17691
A_43_P10072	0.490183	3.132327	0.005158	-2.04763	NM_213564	Rn.15118
A_44_P241190	0.48927	3.246072	0.003973	-1.82936	NM_001013199	Rn.153577
A_42_P601961	0.487725	2.915305	0.00844	-2.45808	XM_225203	Rn.3005
A_44_P932158	0.487385	3.142702	0.005037	-2.0278		
A_42_P512838	0.487372	3.194685	0.004471	-1.9282	XM_217335	Rn.106975
A_44_P562712	0.486028	3.142979	0.005034	-2.02727		
A_44_P409232	0.485898	3.284883	0.003633	-1.75448	NM_031543	Rn.1372
A_44_P370335	0.48549	3.011417	0.006794	-2.27739	NM_001011911	Rn.137862
A_44_P597013	0.484512	3.455686	0.002444	-1.42289		
A_44_P713589	0.482311	2.905104	0.008636	-2.47714		
A_44_P591963	0.481999	2.881548	0.009105	-2.52109	DV728639	Rn.31695
A_44_P822852	0.480143	2.907057	0.008598	-2.4735	XM_001073919	
A_43_P10505	0.479408	2.987691	0.007169	-2.32217	XM_226549	Rn.19188
A_44_P428922	0.479017	2.858597	0.009585	-2.56378	XM_341979	Rn.4188
A_42_P824657	0.47757	3.395593	0.002811	-1.53989	XM_214678	Rn.106111
A_44_P306626	0.476762	2.957734	0.007671	-2.37854	XM_577573	
A_44_P277669	0.47676	2.919375	0.008363	-2.45047	XM_573168	Rn.17552
A_42_P770330	0.47665	2.905529	0.008628	-2.47635	XM_001062632	
A_44_P125145	0.476294	3.001697	0.006945	-2.29575	CF109884	Rn.22478
A_44_P387314	0.476035	2.992752	0.007087	-2.31262		
A_42_P738337	0.475807	3.1039	0.005505	-2.10188	NM_012575	Rn.9709
A_43_P16816	0.47557	2.880264	0.009131	-2.52348	NM_001044290	Rn.168323
A_44_P1022403	0.474294	3.431959	0.002583	-1.46913	NM_001004204	Rn.99691
A_42_P506956	0.473183	3.030181	0.006511	-2.2419	NM_133559	Rn.2899
A_43_P17196	0.473149	2.940814	0.007969	-2.41031	XM_576184	Rn.9087
A_44_P1058112	0.473068	3.475989	0.002332	-1.38329	NM_001009627	Rn.163187
A_44_P578250	0.472654	2.955687	0.007706	-2.38239	DV714571	Rn.12430
A_44_P290327	0.471468	2.889571	0.008942	-2.50613	NM_001004234	Rn.154505
A_44_P669746	0.471266	3.554143	0.001943	-1.23055	XM_229131	Rn.20175
A_44_P776556	0.470685	2.976764	0.007348	-2.34275	XM_342497	Rn.23367
A_44_P239176	0.468474	2.8779	0.009179	-2.52788	NM_001008380	Rn.24884
A_43_P14144	0.468252	3.43806	0.002547	-1.45725	XM_222855	Rn.24811
A_42_P700150	0.467273	4.006422	0.000671	-0.34141	BP483355	Rn.199375
A_44_P240414	0.466987	3.114036	0.005379	-2.08255	NM_001017537	Rn.198161
A_42_P515931	0.466948	2.882067	0.009094	-2.52012	XM_220256	
A_44_P225353	0.465362	2.893573	0.008862	-2.49867	NM_001009678	Rn.59338
A_44_P492624	0.465045	3.048643	0.006243	-2.20692	XM_340867	
A_44_P930534	0.464955	2.895203	0.00883	-2.49563	BC092660	Rn.19800
A_43_P11236	0.463992	3.566089	0.001889	-1.20716	NM_053538	Rn.24799
A_44_P281457	0.463175	3.050164	0.006222	-2.20404	XM_001054883	Rn.129941
A_44_P792746	0.462911	3.04413	0.006308	-2.21548		
A_43_P10692	0.461194	2.880233	0.009132	-2.52353		
A_44_P859124	0.460771	3.061668	0.006061	-2.18221	CB328239	Rn.199012
A_44_P437346	0.460583	3.378211	0.002927	-1.57367	AW435442	Rn.23741
A_44_P368513	0.459819	3.348318	0.003137	-1.63169	BF282682	Rn.96145
A_44_P816687	0.457365	3.01667	0.006713	-2.26746		
A_44_P1008245	0.457175	3.177477	0.004652	-1.96122	XM_216310	Rn.162764
A_44_P997737	0.457138	2.873741	0.009265	-2.53562	AA942848	Rn.7458
A_44_P288443	0.456325	3.444562	0.002509	-1.44458	XM_344542	Rn.100687
A_44_P472635	0.454909	3.455808	0.002444	-1.42266	AA900458	Rn.3340
A_44_P938669	0.454871	2.949417	0.007816	-2.39416	BC127463	Rn.146030
A_44_P496788	0.454063	2.892821	0.008877	-2.50007	NM_001008295	Rn.138993
A_42_P459306	0.453602	2.8625	0.009502	-2.55653	CB314148	Rn.1025
A_44_P1030801	0.453237	3.093839	0.005632	-2.12104	NM_031749	Rn.22161
A_44_P557994	0.451938	3.461765	0.00241	-1.41104	XR_006555	Rn.197577

A_44_P142472	0.451815	3.521363	0.002097	-1.29467	XM_214293	Rn.160899
A_44_P1027866	0.45081	3.185967	0.004562	-1.94493	NM_001011925	Rn.137413
A_44_P292419	0.449724	3.052538	0.006188	-2.19954	AA944408	Rn.96497
A_44_P1045652	0.449511	2.871676	0.009308	-2.53946	XM_001057185	Rn.9266
A_44_P843798	0.448234	3.184221	0.00458	-1.94828	AW144049	Rn.24594
A_42_P792497	0.446787	2.952596	0.00776	-2.38819	NM_023025	Rn.44992
A_44_P747708	0.445849	3.047084	0.006265	-2.20988		
A_42_P809733	0.445214	2.86869	0.009371	-2.54502	XM_347094	Rn.24677
A_44_P281216	0.444489	3.007757	0.00685	-2.2843	XM_341975	Rn.23628
A_42_P666843	0.44252	4.044054	0.000614	-0.26733	XM_214296	Rn.19855
A_44_P838483	0.439995	3.362802	0.003033	-1.60359		
A_44_P980178	0.439693	2.956285	0.007696	-2.38127		
A_42_P838051	0.439586	3.378402	0.002926	-1.5733	XM_216446	Rn.14712
A_44_P211997	0.436673	3.01299	0.00677	-2.27442	BF282863	Rn.92148
A_44_P381177	0.433194	3.01294	0.00677	-2.27451	AA925047	Rn.5827
A_44_P1058421	0.432785	2.963818	0.007566	-2.36711	BC091438	Rn.101513
A_44_P884119	0.432377	2.965508	0.007537	-2.36393	NM_001014061	Rn.42180
A_44_P303883	0.431123	2.981722	0.007266	-2.33341	AI602844	Rn.38282
A_43_P17192	0.430405	3.035662	0.00643	-2.23153		
A_44_P274785	0.427234	2.885761	0.009019	-2.51324	XM_213260	Rn.106750
A_44_P1004260	0.426859	2.843252	0.009919	-2.59226	XM_231121	
A_44_P147888	0.424227	2.870978	0.009323	-2.54076	AA925444	Rn.8224
A_43_P11580	0.423705	2.989546	0.007139	-2.31867	NM_012833	Rn.10265
A_44_P402559	0.422607	3.4454	0.002504	-1.44294	NM_001000910	Rn.142331
A_44_P307646	0.422312	3.162588	0.004813	-1.98975	BI289723	Rn.162620
A_44_P591370	0.421966	3.028304	0.006538	-2.24546		
A_44_P993577	0.42188	3.113554	0.005385	-2.08347	XM_214633	Rn.18307
A_44_P710192	0.419518	2.943288	0.007925	-2.40566	NM_001033067	Rn.42073
A_44_P251542	0.418155	3.706452	0.00136	-0.93179	NM_001008864	Rn.126315
A_44_P1045612	0.416475	3.083853	0.005762	-2.14005	NM_172317	Rn.3896
A_44_P156317	0.413928	3.10122	0.005538	-2.10698	NM_138910	Rn.7262
A_42_P705083	0.413592	3.145056	0.00501	-2.0233	XM_341621	Rn.50003
A_44_P638411	0.411681	2.853388	0.009697	-2.57345		
A_44_P1030648	0.41131	2.865526	0.009437	-2.5509	XM_001079792	Rn.196319
A_43_P23304	0.410921	3.089621	0.005687	-2.12907	NM_199397	Rn.204521
A_44_P476237	0.409138	3.115608	0.005359	-2.07955	BF406637	Rn.7586
A_44_P822839	0.408016	2.958897	0.007651	-2.37636		
A_44_P890053	0.407782	3.120236	0.005303	-2.07072	CA505010	Rn.31650
A_44_P899264	0.405945	2.944854	0.007897	-2.40273		
A_44_P421534	0.405341	3.094016	0.00563	-2.1207	NM_017196	Rn.32080
A_44_P981783	0.402686	2.964042	0.007562	-2.36669		
A_44_P558411	0.39837	3.108826	0.005443	-2.09248	NM_033499	Rn.7724
A_42_P802520	0.396592	2.88759	0.008982	-2.50983	NM_172063	Rn.7844
A_44_P340498	0.396466	2.979403	0.007304	-2.33778	XM_213445	Rn.107812
A_44_P1050444	0.396311	2.966331	0.007523	-2.36238	NM_001007749	Rn.106766
A_44_P229769	0.395256	3.053513	0.006174	-2.19769	NM_031838	Rn.2115
A_44_P140266	0.39507	2.958909	0.007651	-2.37634	XM_227523	Rn.46389
A_44_P562685	0.393179	3.317519	0.003369	-1.69136	XM_575917	Rn.6676
A_44_P810151	0.392701	3.328845	0.003282	-1.66943		
A_43_P14262	0.391785	3.005939	0.006879	-2.28774	XM_001057346	Rn.14962
A_44_P422602	0.390835	2.96519	0.007543	-2.36453	AI177360	Rn.3093
A_44_P667745	0.387442	2.958169	0.007663	-2.37772	DY311022	Rn.198695
A_44_P475617	0.386967	3.213792	0.004279	-1.89149	XM_238082	Rn.153598
A_43_P21364	0.383104	2.963846	0.007566	-2.36706	XM_343342	Rn.56018
A_44_P399999	0.379395	2.872445	0.009292	-2.53803	NM_053752	Rn.3766
A_44_P1002320	0.378477	3.037917	0.006397	-2.22725	BF289377	Rn.11887
A_44_P505188	0.378105	2.861306	0.009527	-2.55875	AI454498	Rn.98152
A_44_P378322	0.375598	3.11716	0.00534	-2.07659	AA858768	

A_44_P290016	0.375006	2.869646	0.009351	-2.54324	BG666843	Rn.163635
A_43_P22494	0.37441	2.98671	0.007185	-2.32401		
A_43_P16530	0.374106	2.874744	0.009245	-2.53376	XM_213733	Rn.16344
A_44_P1043668	0.372798	3.005015	0.006893	-2.28948	NM_001014003	Rn.36169
A_44_P1051894	0.368997	2.981165	0.007275	-2.33446	XR_009243	Rn.23050
A_44_P1047992	0.367006	2.935442	0.008066	-2.42038	NM_012803	Rn.91064
A_42_P685138	0.366955	2.875254	0.009234	-2.53281	NM_153621	Rn.206534
A_44_P351239	0.366576	2.899928	0.008737	-2.48681	NM_001000949	Rn.141828
A_43_P16804	0.363022	2.983577	0.007236	-2.32992	NM_001007652	Rn.3290
A_42_P626023	0.358798	2.843669	0.00991	-2.59148	XM_224232	Rn.19524
A_44_P134465	0.350702	2.917437	0.0084	-2.45409	AA963226	Rn.7262
A_44_P257522	0.348981	2.94563	0.007883	-2.40127	XM_574228	Rn.137597
A_44_P178014	0.346895	2.941703	0.007953	-2.40864	BC079202	Rn.108613
A_44_P273515	0.339925	3.104249	0.0055	-2.10121	DV723169	Rn.87868
A_44_P108993	0.338519	3.041493	0.006345	-2.22048	BF420718	Rn.199052
A_43_P14576	0.33491	3.053342	0.006177	-2.19801		
A_44_P794680	0.332151	2.872494	0.009291	-2.53794		
A_44_P505488	0.329268	2.891953	0.008895	-2.50169	BI288152	Rn.73677
A_44_P591036	0.30765	2.878633	0.009164	-2.52651	XM_001069503	
A_44_P388709	-0.27711	-2.92542	0.00825	-2.43915	NM_145767	Rn.10189
A_44_P163408	-0.30136	-2.9754	0.007371	-2.34532	NM_031798	Rn.11523
A_44_P370774	-0.30644	-2.85795	0.009599	-2.56498	XM_235183	Rn.161928
A_44_P300110	-0.31546	-2.96537	0.00754	-2.36419	XM_234335	Rn.16048
A_44_P515461	-0.31765	-2.8635	0.00948	-2.55466	NM_171990	Rn.8453
A_44_P228903	-0.31934	-3.09516	0.005616	-2.11852	NM_078621	Rn.212750
A_44_P349720	-0.32616	-3.02935	0.006523	-2.24349	NM_001039174	Rn.162979
A_44_P374795	-0.32804	-2.87231	0.009295	-2.53828	XM_232197	Rn.7350
A_44_P242252	-0.32834	-2.90914	0.008558	-2.46961	NM_031237	Rn.2778
A_44_P557376	-0.33462	-2.87129	0.009316	-2.54018	NM_012546	Rn.24039
A_43_P18979	-0.33798	-2.87551	0.009229	-2.53232	XM_340992	Rn.198272
A_43_P16060	-0.33835	-3.09337	0.005639	-2.12194	XM_001065759	Rn.207359
A_44_P332429	-0.34648	-3.15692	0.004876	-2.00061	NM_138900	Rn.4037
A_44_P274526	-0.34742	-3.37997	0.002915	-1.57025	XM_230889	Rn.104945
A_44_P865552	-0.35207	-2.95792	0.007668	-2.37819	AW143038	Rn.31067
A_44_P291081	-0.35425	-2.92225	0.008309	-2.44508	XM_001054316	Rn.154642
A_44_P372555	-0.35573	-2.88808	0.008972	-2.50891	NM_175764	Rn.10541
A_44_P604860	-0.35591	-2.92551	0.008249	-2.43899	BC098774	Rn.23514
A_44_P150432	-0.356	-3.90886	0.000845	-0.53351	D38557	
A_43_P15352	-0.36057	-3.06041	0.006078	-2.1846	NM_080577	Rn.144645
A_43_P18495	-0.36216	-2.85334	0.009698	-2.57354	NM_001011952	Rn.28321
A_44_P304374	-0.36385	-2.95064	0.007795	-2.39186	NM_001012745	Rn.105961
A_44_P187388	-0.37136	-2.93285	0.008114	-2.42524	XR_006961	Rn.197837
A_44_P464428	-0.37173	-3.80866	0.001069	-0.73078	XM_343279	Rn.135394
A_43_P21231	-0.37205	-2.91815	0.008386	-2.45275	XM_217594	Rn.98526
A_42_P703664	-0.37262	-2.90802	0.00858	-2.47169	CV118897	Rn.2835
A_44_P1010416	-0.37272	-3.37702	0.002935	-1.57598	NM_001002853	Rn.209780
A_44_P187524	-0.37635	-2.91619	0.008423	-2.45643	XM_214690	Rn.8645
A_44_P527635	-0.37735	-2.9018	0.0087	-2.48332	NM_001014055	Rn.72687
A_44_P874814	-0.37738	-2.95738	0.007677	-2.3792		
A_44_P437973	-0.37778	-3.08967	0.005686	-2.12898	NM_020090	Rn.205732
A_44_P412418	-0.37849	-3.1606	0.004835	-1.99356	XM_001063618	Rn.214693
A_43_P17282	-0.3807	-3.22863	0.004136	-1.86296	XM_219879	Rn.87733
A_44_P180645	-0.38258	-3.0295	0.006521	-2.2432	BC098851	Rn.74199
A_44_P333452	-0.38551	-3.17521	0.004676	-1.96556	XM_347258	Rn.17832
A_44_P1000561	-0.38605	-2.97455	0.007385	-2.34692	XM_235378	Rn.19593
A_44_P136911	-0.38622	-3.05054	0.006216	-2.20332	BC078917	Rn.8555
A_43_P16885	-0.38682	-2.96898	0.007478	-2.35739	XM_235169	Rn.91829
A_44_P536125	-0.38814	-3.02661	0.006564	-2.24867	NM_053557	Rn.33389

A_44_P656668	-0.38824	-2.86918	0.009361	-2.54411	CK476135	Rn.168123
A_44_P154721	-0.3897	-3.03107	0.006498	-2.24023	XM_220217	Rn.45317
A_44_P116606	-0.39262	-2.98599	0.007196	-2.32538	XM_342337	Rn.133131
A_43_P12267	-0.3927	-3.44003	0.002535	-1.4534	NM_022623	Rn.48736
A_44_P829047	-0.39348	-3.00345	0.006918	-2.29244	CO402576	Rn.170969
A_44_P541431	-0.3953	-2.84737	0.009828	-2.58461	NM_001024246	Rn.24927
A_44_P925410	-0.39681	-3.35767	0.00307	-1.61355	BF386740	Rn.164774
A_43_P22351	-0.39861	-2.98814	0.007162	-2.32132	NM_001024968	Rn.16498
A_43_P19646	-0.39904	-3.37204	0.002969	-1.58566	XM_341833	
A_44_P984158	-0.40054	-3.37646	0.002939	-1.57708	BI291259	Rn.210959
A_43_P11729	-0.4012	-3.11769	0.005334	-2.07558	NM_013134	Rn.9437
A_44_P184955	-0.40216	-2.84347	0.009914	-2.59185	XM_215006	Rn.48
A_44_P374341	-0.40241	-2.84908	0.009791	-2.58144	NM_001029897	Rn.8394
A_44_P395572	-0.40262	-3.15562	0.004891	-2.00308	NM_147210	Rn.10055
A_43_P18148	-0.40267	-2.93286	0.008113	-2.42523	XM_218963	Rn.106856
A_44_P333374	-0.40293	-3.17212	0.004709	-1.97148	XM_001064905	Rn.200853
A_44_P998042	-0.40503	-3.09585	0.005607	-2.11722	XM_229579	Rn.101585
A_44_P128986	-0.40663	-3.06591	0.006003	-2.17415	NM_001034959	Rn.162222
A_44_P893828	-0.4084	-2.85027	0.009765	-2.57923		
A_42_P584710	-0.40976	-3.09809	0.005578	-2.11294	XM_213677	Rn.24350
A_44_P545649	-0.41176	-3.63974	0.00159	-1.06279	NM_001014093	Rn.23423
A_44_P147980	-0.41267	-3.16439	0.004793	-1.98629	XM_576503	Rn.203950
A_44_P126027	-0.41471	-3.39222	0.002833	-1.54646	NM_019333	Rn.90092
A_44_P149599	-0.41546	-2.90685	0.008602	-2.47388	NM_001079705	Rn.16095
A_43_P22795	-0.41616	-2.88041	0.009128	-2.52321	NM_001037204	Rn.163238
A_44_P303716	-0.41734	-3.2341	0.004084	-1.85242	XM_345266	Rn.7999
A_44_P100609	-0.41831	-2.90845	0.008571	-2.4709	XM_001064153	Rn.195635
A_44_P655158	-0.4186	-3.01823	0.00669	-2.26452	CO567276	Rn.203592
A_44_P337579	-0.41874	-2.98399	0.007229	-2.32913	NM_001008346	Rn.14828
A_43_P16847	-0.41912	-3.22015	0.004217	-1.87927	XM_233686	Rn.75037
A_44_P523190	-0.42005	-3.24038	0.004025	-1.84034	XM_343095	Rn.140091
A_44_P683082	-0.42234	-2.88885	0.008957	-2.50748		
A_44_P473314	-0.42418	-2.8769	0.0092	-2.52974	NM_001013997	Rn.139140
A_44_P729266	-0.42506	-3.15391	0.00491	-2.00636	XR_005718	Rn.216190
A_44_P357870	-0.42506	-3.01038	0.00681	-2.27935	NM_031559	Rn.2856
A_43_P15840	-0.42512	-3.05402	0.006167	-2.19672	XM_221874	Rn.6774
A_44_P166464	-0.42539	-3.06617	0.005999	-2.17367	XM_235050	Rn.103930
A_43_P15468	-0.42609	-3.16332	0.004805	-1.98835	NM_012585	Rn.44486
A_44_P549198	-0.42676	-2.96309	0.007579	-2.36849	XM_224165	Rn.132110
A_44_P520700	-0.42685	-2.98657	0.007187	-2.32428	XM_341377	Rn.105397
A_44_P986848	-0.42793	-3.24133	0.004016	-1.8385	XM_341882	Rn.7634
A_44_P206258	-0.43084	-2.93552	0.008065	-2.42023	NM_001034146	Rn.24862
A_44_P123813	-0.43159	-3.14524	0.005008	-2.02295	XM_223597	Rn.114948
A_44_P258277	-0.43204	-2.95456	0.007726	-2.3845	NM_001008507	Rn.203124
A_44_P304493	-0.43247	-3.24532	0.00398	-1.8308	NM_001024970	Rn.213413
A_44_P668388	-0.43357	-2.85225	0.009722	-2.57556	BC098022	Rn.117989
A_44_P1004392	-0.43527	-3.35998	0.003053	-1.60907	XM_221455	Rn.138746
A_44_P328654	-0.43642	-2.95673	0.007688	-2.38042	XM_576882	Rn.155010
A_43_P19234	-0.43781	-2.88006	0.009135	-2.52386	NM_001034926	Rn.106141
A_44_P1026191	-0.43806	-3.23707	0.004056	-1.8467	NM_001039338	Rn.4240
A_44_P116809	-0.43918	-3.84083	0.000991	-0.66746	NM_001014136	Rn.108552
A_44_P501242	-0.43922	-3.00717	0.006859	-2.28542	XM_215947	Rn.20691
A_44_P327515	-0.4406	-3.24906	0.003946	-1.8236	XM_001060307	Rn.16458
A_44_P206154	-0.4407	-3.10407	0.005502	-2.10154	XM_345231	Rn.162605
A_43_P10608	-0.44239	-3.3871	0.002867	-1.5564	XR_006750	Rn.27612
A_44_P188869	-0.44305	-3.2967	0.003535	-1.73163	AW915311	Rn.74134
A_44_P333550	-0.44326	-3.44815	0.002488	-1.43758	NM_001013986	Rn.23222
A_44_P360563	-0.44391	-3.2587	0.003859	-1.80503	NM_017012	Rn.29972

A_44_P456747	-0.44521	-3.18332	0.00459	-1.95	AB012231	Rn.40435
A_43_P12243	-0.44537	-3.0277	0.006547	-2.2466	NM_022540	Rn.2011
A_44_P134186	-0.4455	-3.04818	0.00625	-2.2078	NM_145678	Rn.12477
A_44_P377722	-0.44603	-2.84796	0.009816	-2.58352	NM_001007651	Rn.96097
A_44_P183227	-0.44645	-2.93765	0.008026	-2.41623	CO394022	Rn.100426
A_44_P545867	-0.44655	-3.77292	0.001163	-0.80111	XM_222229	Rn.204260
A_42_P574960	-0.44781	-3.69359	0.001401	-0.95707	XR_009643	Rn.12130
A_44_P682849	-0.44825	-3.11801	0.00533	-2.07497	XM_001081465	
A_44_P462285	-0.45038	-3.33703	0.00322	-1.65358	XM_342391	Rn.6975
A_44_P170864	-0.45042	-3.03589	0.006427	-2.2311	XM_223651	Rn.105388
A_44_P729342	-0.4519	-2.94146	0.007958	-2.40909	XR_009643	
A_44_P124027	-0.45232	-3.64745	0.001562	-1.04766	XM_226458	Rn.50250
A_44_P193644	-0.45245	-2.87449	0.00925	-2.53423	XM_001062296	Rn.22904
A_44_P466608	-0.45412	-2.93843	0.008012	-2.41478	XM_238380	Rn.44078
A_43_P22288	-0.45513	-2.91168	0.008509	-2.46486	XM_001068814	Rn.7810
A_44_P437721	-0.45517	-3.23173	0.004106	-1.85699	CO566833	Rn.202638
A_44_P449819	-0.45565	-3.03144	0.006492	-2.23952	NM_053851	Rn.10739
A_44_P443493	-0.45642	-2.94761	0.007848	-2.39755	NM_001025754	Rn.154781
A_43_P17816	-0.45667	-2.86919	0.00936	-2.54408	NM_199120	Rn.47911
A_44_P544297	-0.45713	-3.20162	0.004401	-1.91488	NM_001009661	Rn.67490
A_44_P114184	-0.45758	-2.8773	0.009192	-2.52899	NM_022543	Rn.2193
A_44_P485592	-0.45768	-2.85191	0.009729	-2.57619	XM_217388	Rn.13913
A_44_P257832	-0.45849	-2.95075	0.007793	-2.39166	XM_224535	Rn.92109
A_44_P174982	-0.45879	-3.17361	0.004693	-1.96862	NM_022601	Rn.158883
A_43_P10332	-0.46048	-3.21317	0.004285	-1.89269	XR_009629	Rn.47423
A_44_P759334	-0.46188	-2.85273	0.009711	-2.57467	NM_001011914	Rn.6666
A_44_P424069	-0.46397	-3.73575	0.001269	-0.8742	XM_218463	Rn.213001
A_44_P324034	-0.46547	-3.0331	0.006468	-2.23638	XM_235942	Rn.92828
A_43_P19519	-0.46565	-3.61272	0.001694	-1.11578	XM_223781	Rn.164613
A_43_P22080	-0.46567	-3.20162	0.004401	-1.91489	NM_001012044	Rn.14256
A_43_P15916	-0.46586	-3.04935	0.006233	-2.20558	XM_342491	Rn.20282
A_44_P249717	-0.46592	-3.18306	0.004592	-1.95051	AW144114	Rn.27433
A_43_P10414	-0.46648	-3.54962	0.001963	-1.2394	NM_001017445	Rn.203456
A_44_P515792	-0.46682	-2.9115	0.008513	-2.46519	XM_341532	Rn.24911
A_44_P260475	-0.46702	-2.98321	0.007242	-2.33061	XM_238362	Rn.28065
A_44_P492629	-0.46977	-2.84876	0.009798	-2.58204	DV727624	Rn.203850
A_44_P412710	-0.46992	-3.09009	0.005681	-2.12818	NM_001025423	Rn.17173
A_44_P211111	-0.47024	-2.94709	0.007857	-2.39853	XM_236367	Rn.147817
A_44_P445521	-0.47043	-3.86055	0.000946	-0.62864	XM_226843	Rn.41113
A_44_P267501	-0.47192	-2.97701	0.007344	-2.34228	NM_001017448	Rn.72531
A_44_P520557	-0.47244	-2.8786	0.009165	-2.52658	XM_230284	Rn.64898
A_43_P21150	-0.47247	-2.97532	0.007372	-2.34548	XM_345825	Rn.15910
A_44_P484877	-0.47253	-2.85768	0.009604	-2.56548	AF475093	Rn.3040
A_44_P283772	-0.47292	-3.07687	0.005855	-2.15332	XM_573876	Rn.162262
A_44_P345136	-0.47316	-2.9995	0.00698	-2.29989	XM_340879	Rn.24583
A_44_P625829	-0.47415	-2.87192	0.009303	-2.53901		
A_44_P427067	-0.47471	-2.92813	0.0082	-2.43407	XM_001076752	Rn.204813
A_43_P13424	-0.47481	-3.15426	0.004906	-2.0057	NM_173290	Rn.162877
A_44_P231655	-0.47639	-2.94462	0.007901	-2.40317	XM_222147	Rn.214191
A_44_P414704	-0.47772	-2.93625	0.008052	-2.41886		
A_44_P397851	-0.47778	-2.98688	0.007182	-2.32369	NM_053518	Rn.44274
A_44_P730048	-0.47798	-2.91687	0.008411	-2.45516		
A_43_P21938	-0.47819	-2.93355	0.008101	-2.42392	XM_220541	Rn.17602
A_44_P381842	-0.47935	-2.85471	0.009669	-2.571	XM_343640	Rn.205336
A_44_P1058502	-0.4794	-3.2103	0.004314	-1.8982	XM_238162	Rn.105910
A_44_P463973	-0.47994	-3.04737	0.006261	-2.20934	NM_001079889	Rn.100471
A_44_P126498	-0.47995	-2.87826	0.009172	-2.52721	XM_234272	Rn.96357
A_43_P15409	-0.48005	-3.04994	0.006225	-2.20447	NM_023971	Rn.162341

A_44_P384833	-0.48023	-2.9969	0.007021	-2.30481	XM_343166	Rn.92148
A_44_P324124	-0.48059	-2.84088	0.009972	-2.59664	XM_220595	Rn.203763
A_44_P350098	-0.48064	-3.05518	0.006151	-2.19453	XM_345938	Rn.135879
A_44_P107177	-0.48124	-2.90317	0.008674	-2.48076	NM_017018	Rn.81032
A_42_P645142	-0.48345	-2.92485	0.008261	-2.44022	NM_019375	Rn.48803
A_44_P356445	-0.48386	-3.42438	0.002629	-1.48388	XM_227547	Rn.162624
A_44_P376905	-0.48447	-3.34695	0.003147	-1.63434	XM_214994	Rn.7939
A_44_P532020	-0.48493	-2.89266	0.008881	-2.50037	XM_219414	Rn.102186
A_44_P501220	-0.48534	-3.3408	0.003192	-1.64627	NM_053951	Rn.81062
A_44_P331109	-0.48553	-3.04809	0.006251	-2.20797	XM_220175	Rn.49741
A_44_P105229	-0.48555	-3.2408	0.004021	-1.83951	NM_001013157	Rn.3128
A_44_P664802	-0.48591	-2.95902	0.007649	-2.37613	DV728182	Rn.164504
A_44_P136845	-0.48601	-3.06977	0.00595	-2.16681	XM_340879	Rn.24583
A_44_P397495	-0.4878	-3.24097	0.00402	-1.83919	NM_024359	Rn.10852
A_44_P311955	-0.48841	-3.19652	0.004453	-1.92467	NM_017171	Rn.159093
A_42_P765031	-0.48852	-2.95014	0.007803	-2.3928	XM_346083	Rn.15475
A_44_P480830	-0.48934	-3.11408	0.005378	-2.08246	NM_001079943	Rn.120278
A_44_P661516	-0.4905	-3.44322	0.002516	-1.44718		
A_43_P18324	-0.49096	-3.08858	0.0057	-2.13105	NM_207165	Rn.105801
A_44_P215931	-0.49107	-3.19707	0.004447	-1.92361	AI232088	Rn.7630
A_44_P501992	-0.49147	-3.30406	0.003475	-1.71741	BC071175	Rn.64648
A_44_P693682	-0.49161	-2.88948	0.008944	-2.50631		
A_43_P12802	-0.49163	-3.43107	0.002589	-1.47085	NM_053418	Rn.11946
A_43_P18343	-0.49212	-3.14003	0.005068	-2.03291	XM_001072617	Rn.18248
A_44_P299123	-0.49241	-3.26694	0.003786	-1.78913	XM_215847	Rn.162718
A_44_P301512	-0.49254	-3.3645	0.003021	-1.6003	NM_176077	Rn.66254
A_42_P612592	-0.49502	-3.40554	0.002747	-1.52055	XM_001067905	
A_44_P583834	-0.49572	-3.10123	0.005538	-2.10696	XM_223597	Rn.114948
A_44_P534113	-0.49615	-2.92727	0.008216	-2.43569	NM_053866	Rn.22260
A_44_P409977	-0.49681	-3.33505	0.003235	-1.65741	NM_001012742	Rn.208255
A_44_P403816	-0.49734	-3.3934	0.002825	-1.54417	AW142588	Rn.164773
A_44_P1057763	-0.49735	-2.98209	0.00726	-2.33271	NM_053576	Rn.42
A_44_P768438	-0.49816	-3.59387	0.00177	-1.15275	XM_225314	Rn.146842
A_44_P539167	-0.49833	-3.83026	0.001016	-0.68827	XR_009059	Rn.196664
A_44_P729229	-0.49851	-2.96652	0.00752	-2.36204	XR_009322	Rn.197449
A_44_P210753	-0.49943	-3.05798	0.006112	-2.18921	M22631	Rn.6033
A_44_P935947	-0.49967	-3.17126	0.004718	-1.97313	XM_342684	Rn.4057
A_44_P303044	-0.49995	-3.02514	0.006585	-2.25144	XM_239258	Rn.7144
A_44_P555760	-0.50031	-3.53246	0.002044	-1.27297	XM_001064688	Rn.213803
A_44_P286467	-0.50037	-2.84527	0.009875	-2.58852	XM_237056	Rn.63731
A_44_P388973	-0.5011	-3.16409	0.004797	-1.98686	NM_012571	Rn.5819
A_44_P123102	-0.50115	-3.33832	0.00321	-1.65107	NM_001039607	Rn.116613
A_44_P293438	-0.50272	-2.92651	0.00823	-2.4371	XM_340949	Rn.105372
A_44_P993382	-0.50339	-2.87293	0.009282	-2.53713	NM_031121	Rn.35908
A_44_P351490	-0.50483	-4.10147	0.000536	-0.15435	NM_031797	Rn.3022
A_44_P370213	-0.50505	-2.97275	0.007415	-2.3503	NM_012749	Rn.144561
A_43_P16583	-0.50505	-3.43122	0.002588	-1.47056	XM_214539	Rn.106977
A_44_P400520	-0.50551	-3.17475	0.004681	-1.96644	NM_012866	Rn.1457
A_44_P133464	-0.51116	-2.9112	0.008518	-2.46575		
A_43_P23298	-0.51124	-3.86426	0.000938	-0.62133	XM_001079737	Rn.161833
A_44_P281750	-0.51157	-3.20239	0.004393	-1.9134	XM_237790	Rn.1344
A_44_P533572	-0.51184	-3.29941	0.003513	-1.7264	AI639343	Rn.13858
A_44_P292900	-0.51223	-2.94967	0.007812	-2.39368	XM_219377	Rn.162468
A_44_P340180	-0.51227	-2.94135	0.00796	-2.4093	XM_341668	Rn.9619
A_44_P1048296	-0.51255	-3.08876	0.005698	-2.1307	NM_172023	Rn.178664
A_44_P238019	-0.51394	-3.07438	0.005888	-2.15807	AW143671	Rn.15201
A_43_P21191	-0.51398	-3.711	0.001345	-0.92285	XM_234988	Rn.99361
A_44_P1034030	-0.51459	-2.93789	0.008022	-2.41579	XM_220420	Rn.17594

A_44_P290424	-0.51475	-3.15403	0.004908	-2.00613	NM_080905	Rn.73937
A_44_P461678	-0.51506	-3.46513	0.002391	-1.40448	XM_574677	Rn.23632
A_44_P363522	-0.51511	-3.14014	0.005067	-2.03269	NM_001031661	Rn.41210
A_43_P23210	-0.51525	-2.92979	0.00817	-2.43096	NM_001012153	Rn.14671
A_44_P477633	-0.51654	-2.96959	0.007468	-2.35626	NM_174864	Rn.51975
A_44_P226906	-0.51705	-3.15618	0.004884	-2.00202	NM_147205	Rn.54567
A_44_P540279	-0.5175	-3.27808	0.00369	-1.76761	AW144704	Rn.91572
A_44_P377698	-0.51802	-3.17959	0.004629	-1.95717	XR_006438	Rn.196722
A_44_P157009	-0.51818	-2.90577	0.008623	-2.4759	XM_232995	Rn.15969
A_44_P445369	-0.5182	-3.13113	0.005173	-2.04992	XM_001080476	Rn.178658
A_44_P520929	-0.51833	-3.7294	0.001288	-0.88669	NM_001007675	Rn.203149
A_44_P208566	-0.52022	-3.87786	0.000909	-0.59456	XM_341567	Rn.36200
A_44_P301875	-0.52102	-3.13858	0.005085	-2.03568	XM_343761	Rn.79380
A_43_P10744	-0.52121	-2.95779	0.00767	-2.37843	NM_001014212	Rn.105523
A_43_P16724	-0.5214	-2.91993	0.008353	-2.44943	NM_001014188	Rn.25170
A_44_P974892	-0.52152	-3.87896	0.000906	-0.59238		
A_43_P18451	-0.52167	-2.986	0.007196	-2.32535	NM_080579	Rn.99370
A_44_P342730	-0.5218	-3.12478	0.005248	-2.06205	NM_001012275	Rn.9570
A_44_P362498	-0.52359	-2.92854	0.008193	-2.43332	AW143788	Rn.7599
A_44_P255678	-0.52368	-3.20679	0.004349	-1.90496	XM_228777	Rn.61758
A_43_P13675	-0.5242	-3.26187	0.003831	-1.7989	CO570281	Rn.11891
A_44_P150877	-0.52545	-2.92217	0.008311	-2.44524	XM_001080836	
A_44_P109834	-0.52606	-3.99713	0.000686	-0.35971	XM_341783	Rn.100654
A_42_P644929	-0.52782	-2.88149	0.009106	-2.52119	NM_021579	Rn.159847
A_44_P171155	-0.52796	-3.3758	0.002943	-1.57836	NM_001014221	Rn.198445
A_44_P142843	-0.52822	-3.25062	0.003931	-1.8206	XM_001065442	Rn.144456
A_43_P17871	-0.52907	-2.93669	0.008044	-2.41804	NM_001013186	Rn.153573
A_44_P940553	-0.52977	-3.0938	0.005633	-2.12112	AW143148	
A_43_P21003	-0.52993	-2.84354	0.009913	-2.59172	NM_001033892	Rn.98615
A_44_P651694	-0.53017	-3.31877	0.003359	-1.68894		
A_44_P776937	-0.53028	-3.11071	0.00542	-2.08889	CR754170	Rn.200075
A_44_P227201	-0.53071	-3.39678	0.002803	-1.53759	XM_225013	Rn.99777
A_43_P10797	-0.53226	-3.6099	0.001705	-1.12133	BG666794	Rn.3027
A_44_P471691	-0.53234	-4.40431	0.000262	0.439495	XM_223781	Rn.164613
A_44_P378225	-0.53263	-3.26956	0.003763	-1.78406	NM_001011993	Rn.40859
A_44_P315714	-0.53523	-2.88563	0.009022	-2.51348	NM_021577	Rn.64591
A_44_P683068	-0.53525	-3.12787	0.005211	-2.05615	NM_017032	Rn.95959
A_44_P260055	-0.53652	-3.20182	0.004399	-1.9145	NM_031043	Rn.3661
A_42_P750029	-0.53712	-3.55609	0.001934	-1.22673	BF559198	Rn.50843
A_44_P490460	-0.53724	-3.60883	0.001709	-1.12341	XM_224518	Rn.106438
A_44_P230960	-0.5381	-3.08142	0.005794	-2.14468	AW144271	Rn.7380
A_44_P415459	-0.53854	-2.96777	0.007499	-2.35967	CB547990	Rn.161999
A_44_P228874	-0.53882	-2.99646	0.007028	-2.30563	NM_012815	Rn.8365
A_44_P516073	-0.53914	-3.63738	0.001599	-1.06742	XM_217194	Rn.23973
A_44_P223808	-0.54002	-3.31299	0.003404	-1.70014	XM_225711	Rn.195658
A_44_P170338	-0.54027	-2.85659	0.009628	-2.56751	NM_001002815	Rn.160715
A_44_P485614	-0.54041	-3.42581	0.00262	-1.4811		
A_44_P241371	-0.54044	-2.97622	0.007357	-2.34377	NM_001013161	Rn.8860
A_44_P175543	-0.54102	-3.07212	0.005918	-2.16235	NM_001033699	Rn.1309
A_44_P579630	-0.54104	-2.85254	0.009716	-2.57502		
A_44_P515211	-0.54129	-3.11998	0.005306	-2.07121	NM_145765	Rn.84873
A_44_P466183	-0.54131	-3.02375	0.006606	-2.25407	NM_133410	Rn.23625
A_44_P168778	-0.54181	-2.91599	0.008427	-2.4568	XM_233220	Rn.213735
A_44_P201601	-0.54268	-3.00096	0.006957	-2.29713	XM_216096	Rn.45039
A_44_P180080	-0.54313	-3.37249	0.002966	-1.58478	NM_022867	Rn.41412
A_44_P353927	-0.54317	-3.39999	0.002782	-1.53135	XM_341931	Rn.145190
A_44_P344340	-0.54321	-3.02846	0.006536	-2.24516	U78135	Rn.198302
A_44_P310949	-0.54343	-3.14843	0.004972	-2.01684	NM_001004283	Rn.3463

A_43_P12473	-0.54368	-3.97258	0.000727	-0.40803	NM_031006	Rn.10056
A_44_P207215	-0.54399	-3.71816	0.001323	-0.90879	XM_237115	Rn.9067
A_44_P281155	-0.54407	-2.91962	0.008359	-2.45001	XM_341127	Rn.3649
A_44_P323729	-0.54477	-3.21994	0.004219	-1.87968	NM_001042621	Rn.1836
A_43_P10248	-0.54491	-2.97352	0.007402	-2.34885	NM_001014117	Rn.34113
A_44_P147622	-0.54517	-3.81095	0.001064	-0.72627	XM_343462	Rn.36397
A_44_P386375	-0.54538	-3.16405	0.004797	-1.98695	NM_053842	Rn.34914
A_44_P333232	-0.54545	-3.66568	0.001496	-1.01189	XM_342803	Rn.11991
A_44_P265326	-0.54573	-3.2835	0.003644	-1.75714	BC087674	Rn.22763
A_43_P19260	-0.54602	-3.23084	0.004115	-1.8587	XM_001053368	Rn.155491
A_44_P525101	-0.54616	-3.59325	0.001773	-1.15395	XR_006056	Rn.157025
A_44_P534508	-0.54659	-3.24439	0.003988	-1.83261	XM_231749	Rn.204902
A_44_P447514	-0.54691	-3.299	0.003516	-1.72718	NM_017155	Rn.32078
A_44_P493688	-0.54701	-3.60887	0.001709	-1.12333	NM_001008371	Rn.162484
A_44_P587322	-0.54784	-3.46677	0.002382	-1.40128	AW917250	
A_43_P15866	-0.54831	-3.51439	0.002132	-1.3083	XM_341686	Rn.9446
A_43_P21374	-0.54873	-3.25803	0.003865	-1.80631	NM_001008353	Rn.8839
A_44_P557474	-0.54968	-3.03358	0.006461	-2.23547	NM_053742	Rn.2399
A_44_P191022	-0.54984	-3.01264	0.006775	-2.27508	NM_053674	Rn.7279
A_44_P338068	-0.55011	-3.50831	0.002162	-1.32018	NM_001024355	Rn.19890
A_44_P331003	-0.55171	-3.54098	0.002003	-1.25631	XM_234281	Rn.106963
A_44_P222603	-0.55189	-3.19846	0.004433	-1.92096	XM_341580	Rn.1014
A_44_P995544	-0.55211	-3.05489	0.006155	-2.19507	NM_001039034	Rn.106818
A_44_P241781	-0.55211	-3.1802	0.004623	-1.95599	BF558420	Rn.19106
A_44_P228715	-0.55251	-4.37986	0.000278	0.391719	NM_019275	Rn.9774
A_44_P803129	-0.55256	-3.49264	0.002243	-1.35079	AW918772	Rn.204958
A_44_P352104	-0.55268	-3.02151	0.00664	-2.2583	XM_216993	Rn.94733
A_44_P168083	-0.55308	-3.10274	0.005519	-2.10409	NM_019381	Rn.107678
A_44_P443228	-0.55414	-3.06694	0.005989	-2.17221	XM_341622	Rn.92711
A_43_P21031	-0.55451	-3.52301	0.002089	-1.29145	XM_232343	Rn.14316
A_44_P171128	-0.55544	-2.84548	0.00987	-2.58812	XM_232987	Rn.98222
A_44_P160550	-0.55574	-2.92182	0.008317	-2.44589	NM_017116	Rn.6822
A_44_P349689	-0.55591	-2.88141	0.009107	-2.52134	NM_001009831	Rn.135117
A_44_P550670	-0.55632	-3.08033	0.005809	-2.14675	XM_223611	Rn.21479
A_44_P454426	-0.55634	-3.39767	0.002798	-1.53585	AF133906	Rn.202873
A_43_P15280	-0.5565	-3.60497	0.001725	-1.13099	NM_019194	Rn.59871
A_43_P21715	-0.55746	-2.90672	0.008605	-2.47413	BC091390	Rn.206833
A_44_P147582	-0.55818	-3.22017	0.004217	-1.87922	NM_001079936	Rn.3305
A_44_P173345	-0.5586	-3.37421	0.002954	-1.58144	XM_342595	Rn.198182
A_44_P381483	-0.55869	-3.27132	0.003748	-1.78067	XM_213930	Rn.203104
A_44_P167403	-0.55872	-3.00447	0.006902	-2.29051	AA875633	Rn.13589
A_44_P191924	-0.55935	-3.06514	0.006013	-2.17562	NM_019906	Rn.11008
A_43_P20321	-0.55979	-4.06732	0.000581	-0.22154	XM_228770	Rn.38478
A_44_P744309	-0.56059	-3.1147	0.005371	-2.08128	XM_222534	Rn.195555
A_44_P289630	-0.56239	-3.13467	0.005131	-2.04316	NM_017261	Rn.91361
A_44_P1019326	-0.56271	-2.99167	0.007105	-2.31467	NM_199372	Rn.91523
A_44_P292479	-0.56303	-2.98757	0.007171	-2.3224	NM_053880	Rn.11014
A_44_P146833	-0.56378	-2.95072	0.007793	-2.39172	M92076	Rn.41715
A_44_P284462	-0.56395	-3.36363	0.003027	-1.60199	NM_031728	Rn.11022
A_44_P529934	-0.56395	-2.99514	0.007049	-2.30812	XM_225661	Rn.132678
A_43_P16274	-0.5643	-3.15864	0.004857	-1.9973	S53987	
A_43_P16927	-0.56431	-3.27905	0.003682	-1.76575	XM_228073	Rn.11702
A_43_P19892	-0.56484	-3.205	0.004367	-1.90839	NM_001014255	Rn.22786
A_44_P147062	-0.56493	-2.92845	0.008194	-2.43348	XM_220095	Rn.26552
A_42_P627394	-0.56496	-4.06189	0.000589	-0.23223	BC089998	Rn.36437
A_44_P299699	-0.56503	-3.06735	0.005983	-2.17142	NM_001013896	Rn.19940
A_44_P309513	-0.56612	-3.32534	0.003308	-1.67622	NM_001024367	Rn.140506
A_44_P207634	-0.5662	-2.88958	0.008942	-2.50612	NM_001014148	Rn.1073

A_44_P618177	-0.56659	-3.25823	0.003863	-1.80592	CF110664	Rn.16536
A_44_P156805	-0.56674	-2.91367	0.008471	-2.46113	XM_227475	Rn.103975
A_44_P280759	-0.56806	-2.94932	0.007818	-2.39435	NM_012992	Rn.54537
A_44_P212355	-0.56835	-3.49354	0.002238	-1.34904	XM_232466	Rn.32960
A_44_P468544	-0.56844	-3.19639	0.004454	-1.92493	NM_001013126	Rn.100496
A_44_P738643	-0.56863	-3.29295	0.003566	-1.73889		
A_44_P348973	-0.56889	-3.01678	0.006712	-2.26725	NM_001004277	Rn.93631
A_44_P261883	-0.56906	-2.92282	0.008299	-2.44401	NM_134402	Rn.13196
A_44_P650648	-0.57023	-4.19154	0.000434	0.022684	BF561546	Rn.92969
A_44_P1060064	-0.57094	-3.12563	0.005238	-2.06043	NM_001024883	Rn.39247
A_44_P409866	-0.57204	-3.36268	0.003034	-1.60383	NM_013221	Rn.89226
A_44_P347771	-0.57225	-3.37072	0.002978	-1.58823	CB579735	Rn.171407
A_44_P384688	-0.57263	-3.44036	0.002533	-1.45276		
A_44_P356027	-0.57294	-2.92043	0.008344	-2.4485	NM_053642	Rn.18741
A_44_P249606	-0.57308	-2.90444	0.008649	-2.47839	NM_001025718	Rn.13772
A_43_P15129	-0.57378	-4.36307	0.000289	0.358901	BF558849	
A_44_P137356	-0.5742	-2.85938	0.009568	-2.56232	NM_053523	Rn.4028
A_44_P550497	-0.57495	-3.48169	0.002301	-1.37217	NM_022684	Rn.162782
A_44_P365765	-0.57566	-3.11654	0.005348	-2.07778	XM_224579	Rn.3704
A_44_P190115	-0.57607	-3.42078	0.002651	-1.4909	XM_235940	Rn.8862
A_43_P19005	-0.57629	-2.90927	0.008555	-2.46936	XM_574285	Rn.79194
A_44_P229352	-0.57702	-3.11145	0.005411	-2.08749	XM_222717	Rn.28005
A_44_P729514	-0.57738	-2.84434	0.009895	-2.59024	XM_573199	Rn.8286
A_44_P130849	-0.57774	-2.88796	0.008975	-2.50914	XM_224627	Rn.19370
A_44_P534249	-0.57802	-2.84357	0.009912	-2.59166	NM_001013874	Rn.98296
A_44_P707011	-0.57859	-3.34419	0.003167	-1.63969	CO393635	Rn.3583
A_42_P747877	-0.57873	-3.36184	0.00304	-1.60545	NM_172029	Rn.9181
A_44_P101223	-0.57904	-3.77967	0.001145	-0.78781	XM_344744	Rn.99039
A_44_P246311	-0.58042	-3.0232	0.006615	-2.25511	NM_001017473	Rn.8249
A_44_P548450	-0.58071	-2.96256	0.007588	-2.36947	XM_225160	Rn.44057
A_44_P356596	-0.58119	-2.91812	0.008387	-2.45281	XM_342783	Rn.62095
A_44_P368170	-0.58179	-3.62106	0.001661	-1.09945	XM_342563	Rn.51809
A_44_P987846	-0.58185	-3.03809	0.006395	-2.22692	XM_001075075	Rn.17532
A_44_P246554	-0.582	-3.26244	0.003826	-1.7978	CO566833	Rn.202638
A_44_P463557	-0.58263	-3.2119	0.004298	-1.89513	NM_013067	Rn.4224
A_44_P231583	-0.58303	-2.98095	0.007279	-2.33488	NM_032462	Rn.74239
A_44_P130732	-0.58382	-3.33593	0.003228	-1.6557	XM_001064066	Rn.179747
A_43_P15355	-0.58468	-2.99721	0.007016	-2.30421	NM_130755	Rn.66581
A_44_P235090	-0.58628	-3.07251	0.005913	-2.16161	XM_234988	Rn.99361
A_44_P271989	-0.58637	-2.86728	0.0094	-2.54764	XM_345169	Rn.11634
A_44_P170070	-0.58651	-3.24275	0.004003	-1.83576	XM_236380	Rn.82537
A_44_P810365	-0.5868	-2.87055	0.009332	-2.54156	CV104092	Rn.8410
A_44_P382611	-0.58712	-2.96243	0.00759	-2.36972	NM_001031651	Rn.143886
A_44_P444039	-0.58729	-3.00114	0.006954	-2.2968	XM_215897	Rn.98236
A_44_P637334	-0.58783	-3.14295	0.005035	-2.02733		
A_44_P262569	-0.58791	-3.63106	0.001623	-1.07983	XM_341961	Rn.137040
A_44_P344730	-0.58803	-3.63375	0.001613	-1.07454	NM_001009405	Rn.162617
A_44_P103692	-0.58922	-2.93733	0.008032	-2.41684	XM_234484	Rn.22873
A_44_P698466	-0.58941	-2.91385	0.008468	-2.46081	XM_001062636	Rn.167797
A_44_P504298	-0.58951	-2.94017	0.007981	-2.41152	XM_221077	Rn.8595
A_44_P509129	-0.58977	-2.87832	0.009171	-2.52709	BC083806	Rn.94152
A_42_P744464	-0.59018	-3.17585	0.004669	-1.96434	XM_340740	Rn.98642
A_43_P17175	-0.5903	-3.5954	0.001764	-1.14974	NM_001031642	Rn.103402
A_43_P21386	-0.59044	-3.17171	0.004713	-1.97227	CB546740	
A_43_P11905	-0.59083	-3.05051	0.006217	-2.20339	NM_017303	Rn.32090
A_44_P471386	-0.59181	-2.88997	0.008934	-2.50539	NM_001013107	Rn.107901
A_44_P530041	-0.59218	-2.96383	0.007566	-2.36709	XM_343804	Rn.208476
A_44_P529718	-0.59257	-3.81242	0.00106	-0.72338	XM_341914	Rn.162461

A_44_P237220	-0.59259	-4.39152	0.000271	0.4145	XM_214720	Rn.98912
A_44_P398587	-0.59283	-3.0323	0.006479	-2.23789	XM_343766	Rn.7273
A_44_P292327	-0.59331	-3.03932	0.006377	-2.2246	NM_017006	Rn.11040
A_44_P782486	-0.5935	-3.29362	0.00356	-1.7376	XM_575380	Rn.204577
A_44_P508462	-0.59352	-2.8832	0.009071	-2.518	NM_131911	Rn.20465
A_44_P222011	-0.59391	-3.13405	0.005138	-2.04434	NM_017343	Rn.103179
A_44_P312371	-0.59459	-3.50097	0.0022	-1.33452	NM_172066	Rn.90036
A_43_P21821	-0.59573	-3.17816	0.004644	-1.9599	CB547114	
A_44_P222353	-0.59609	-3.54516	0.001984	-1.24812	NM_001040128	Rn.207169
A_44_P217462	-0.59611	-3.52564	0.002077	-1.2863	U78138	
A_43_P13557	-0.59734	-3.37099	0.002976	-1.58769	CF110620	Rn.65543
A_44_P226353	-0.59801	-3.19841	0.004433	-1.92106	NM_031672	Rn.89268
A_44_P438269	-0.59811	-3.87659	0.000911	-0.59705	NM_022585	Rn.6290
A_44_P1009692	-0.5983	-3.21504	0.004267	-1.88909	NM_001025624	Rn.3517
A_43_P12191	-0.59871	-3.50489	0.00218	-1.32686	NM_022265	Rn.206228
A_44_P302996	-0.59922	-3.48459	0.002285	-1.36651	XM_342171	Rn.137445
A_44_P461489	-0.59927	-2.86872	0.00937	-2.54495	XM_231528	Rn.91746
A_44_P686863	-0.59961	-3.75756	0.001206	-0.83131		
A_43_P18281	-0.59965	-4.07866	0.000566	-0.19924	XM_001057305	Rn.18063
A_44_P703930	-0.60001	-3.46466	0.002394	-1.40539		
A_44_P955997	-0.60011	-3.38018	0.002913	-1.56985	DN932367	Rn.47341
A_44_P327535	-0.6004	-3.66964	0.001482	-1.00409	XR_007094	Rn.203188
A_44_P1049827	-0.60075	-2.89238	0.008886	-2.50089	NM_024128	Rn.2865
A_44_P516958	-0.60117	-3.16315	0.004807	-1.98867	EF076766	Rn.203841
A_43_P10835	-0.60123	-2.90511	0.008636	-2.47713	BP482727	Rn.101963
A_44_P823303	-0.60152	-3.79252	0.001111	-0.76255		
A_44_P186813	-0.60233	-3.18022	0.004622	-1.95597	XM_213276	Rn.18711
A_44_P128711	-0.60233	-2.90838	0.008573	-2.47103	XM_217566	Rn.58623
A_43_P18294	-0.60239	-3.91961	0.000823	-0.51235	BC099835	Rn.134218
A_44_P326176	-0.60268	-3.09586	0.005607	-2.1172	XM_222244	Rn.207591
A_44_P217909	-0.60295	-3.43241	0.002581	-1.46825	XM_228644	Rn.18311
A_44_P851961	-0.60325	-2.87498	0.00924	-2.53332	NM_001009504	Rn.214871
A_44_P325189	-0.60366	-4.06665	0.000582	-0.22286	NM_175578	Rn.24428
A_44_P789739	-0.60378	-3.50519	0.002178	-1.32628	NM_001030024	Rn.19386
A_42_P688027	-0.60651	-3.11857	0.005323	-2.0739	NM_030999	Rn.10499
A_44_P114298	-0.60651	-3.38942	0.002852	-1.55189	XM_215693	Rn.7301
A_44_P344096	-0.60691	-2.86739	0.009398	-2.54744	NM_138890	Rn.7771
A_43_P10536	-0.60796	-2.9444	0.007905	-2.40357	XR_007603	Rn.34413
A_44_P917935	-0.60799	-2.95671	0.007689	-2.38047		
A_44_P377653	-0.60836	-3.65253	0.001543	-1.03769		
A_43_P17872	-0.60838	-3.01364	0.006759	-2.27318	CB545618	Rn.128947
A_44_P142318	-0.60841	-3.00995	0.006816	-2.28015	XM_222171	Rn.162124
A_44_P400936	-0.60866	-3.02171	0.006637	-2.25793	XR_006350	Rn.196876
A_44_P735345	-0.6091	-3.63084	0.001624	-1.08026	XM_576015	Rn.16708
A_44_P495832	-0.6102	-3.6337	0.001613	-1.07465	XM_343442	Rn.24236
A_44_P356711	-0.61056	-3.05191	0.006197	-2.20073	XM_343094	Rn.64438
A_44_P702685	-0.61115	-3.01591	0.006725	-2.2689	CO402564	Rn.127735
A_43_P18909	-0.61132	-3.04512	0.006293	-2.21361	XM_236648	Rn.53471
A_44_P809536	-0.61165	-3.41439	0.002691	-1.50333	AW144200	Rn.7037
A_44_P475932	-0.61234	-2.91548	0.008437	-2.45775	NM_001037496	Rn.97794
A_44_P465188	-0.61384	-3.06277	0.006046	-2.18012	NM_053445	Rn.28161
A_44_P192018	-0.61388	-4.13581	0.000494	-0.08684		
A_44_P354806	-0.6145	-3.10653	0.005472	-2.09686	NM_172072	Rn.9316
A_44_P477135	-0.61523	-3.41577	0.002682	-1.50064	NM_052801	Rn.11059
A_44_P513246	-0.61532	-3.22288	0.004191	-1.87402	XM_341091	Rn.137498
A_44_P348983	-0.61597	-2.9451	0.007893	-2.40226	XM_340889	Rn.12214
A_44_P671422	-0.61639	-3.22546	0.004166	-1.86906		
A_43_P18993	-0.61667	-2.95805	0.007665	-2.37796	XM_216679	Rn.48387

A_44_P261179	-0.61674	-3.75183	0.001222	-0.84258	XM_343177	Rn.41593
A_44_P384223	-0.61722	-3.30717	0.00345	-1.71139	NM_024151	Rn.35935
A_44_P358664	-0.61725	-2.8855	0.009024	-2.51372	XM_223454	Rn.162277
A_44_P418824	-0.61742	-3.61845	0.001671	-1.10456	NM_053430	Rn.16664
A_44_P253196	-0.61787	-3.57781	0.001838	-1.1842	XM_228114	Rn.204911
A_43_P18795	-0.61804	-2.92636	0.008233	-2.43738	NM_001047849	Rn.6835
A_44_P108680	-0.61847	-3.48234	0.002297	-1.37089	NM_057116	Rn.54550
A_44_P396772	-0.61934	-3.7538	0.001217	-0.83871	NM_001037363	Rn.96330
A_44_P457129	-0.61955	-3.00071	0.006961	-2.2976	XM_227809	Rn.198185
A_44_P301812	-0.61984	-3.24171	0.004013	-1.83777	NM_001005765	Rn.106321
A_44_P183048	-0.62005	-3.44636	0.002498	-1.44107	XM_233467	Rn.162834
A_44_P199358	-0.6203	-2.94943	0.007816	-2.39414	XM_229162	
A_43_P12127	-0.62081	-3.05431	0.006163	-2.19617	NM_021842	Rn.27154
A_44_P526729	-0.62244	-3.0417	0.006342	-2.22008	NM_031135	Rn.2398
A_44_P240973	-0.62249	-3.90138	0.00086	-0.54824	NM_022264	Rn.54004
A_44_P370761	-0.62259	-3.28713	0.003614	-1.75013	XM_236009	Rn.96240
A_44_P135944	-0.62263	-2.90869	0.008567	-2.47045	XM_220178	Rn.162033
A_44_P396198	-0.62327	-3.02282	0.00662	-2.25583	NM_001025421	Rn.203134
A_44_P400884	-0.62356	-3.20606	0.004356	-1.90635	XM_345073	
A_44_P552986	-0.6238	-3.46457	0.002394	-1.40556	NM_001007693	Rn.162477
A_44_P523016	-0.62391	-2.86862	0.009372	-2.54514	NM_001024314	Rn.128955
A_44_P1024729	-0.62396	-3.78055	0.001142	-0.78609	XR_006336	Rn.24932
A_44_P303777	-0.62401	-3.11804	0.00533	-2.07491	AW143533	Rn.98831
A_44_P271625	-0.62466	-3.41883	0.002663	-1.49469	NM_053291	Rn.108127
A_43_P17160	-0.62594	-2.95723	0.00768	-2.3795	NM_001017383	Rn.22468
A_44_P469040	-0.62652	-3.78657	0.001126	-0.77425	XM_235547	Rn.36745
A_44_P917437	-0.62664	-3.44398	0.002512	-1.4457		
A_44_P548203	-0.62671	-3.06936	0.005956	-2.1676	BC087677	Rn.18057
A_44_P293124	-0.62819	-2.88076	0.009121	-2.52256	NM_001044243	Rn.22314
A_44_P321359	-0.62827	-3.04759	0.006258	-2.20892	XM_226455	Rn.213190
A_44_P779609	-0.6288	-3.7657	0.001183	-0.81531	NM_001037652	Rn.19409
A_44_P171183	-0.6293	-3.37775	0.00293	-1.57456	NM_001044267	Rn.7910
A_43_P13046	-0.62963	-3.65917	0.001519	-1.02466	NM_057147	Rn.34191
A_44_P318999	-0.62973	-2.91329	0.008478	-2.46184		
A_43_P10386	-0.63022	-4.11636	0.000518	-0.12509	CA511607	Rn.96943
A_44_P192548	-0.63034	-3.57658	0.001843	-1.18662	XM_343535	Rn.3595
A_44_P494723	-0.6305	-2.86557	0.009436	-2.55082	XM_343716	
A_44_P262192	-0.63125	-2.85725	0.009614	-2.56629	NM_182814	Rn.97889
A_44_P240248	-0.63219	-3.34634	0.003151	-1.63553	AB012232	Rn.40435
A_44_P492268	-0.63219	-3.03368	0.006459	-2.23527	XM_212982	
A_44_P403084	-0.63229	-3.77737	0.001151	-0.79235	NM_001039044	Rn.111901
A_44_P461807	-0.6327	-4.1281	0.000504	-0.10199	XM_229225	Rn.139712
A_44_P348825	-0.63298	-3.27325	0.003731	-1.77694	NM_024150	Rn.62175
A_44_P398014	-0.63313	-3.13731	0.0051	-2.03811	NM_053448	Rn.17284
A_44_P527134	-0.63402	-2.96823	0.007491	-2.35881	BC081744	Rn.4252
A_44_P191962	-0.63406	-3.09882	0.005569	-2.11155	NM_001007734	Rn.4220
A_43_P19178	-0.63417	-3.81515	0.001053	-0.718	NM_031574	Rn.23055
A_44_P400857	-0.63445	-3.02525	0.006584	-2.25123	NM_001011926	Rn.201686
A_43_P13362	-0.63472	-3.00067	0.006961	-2.29769	NM_139094	Rn.10529
A_44_P908874	-0.63479	-3.3134	0.003401	-1.69933		
A_44_P112488	-0.63547	-3.011	0.0068	-2.27818	XM_343087	Rn.40168
A_44_P454180	-0.63552	-3.31439	0.003393	-1.69742	NM_182675	Rn.2496
A_44_P288548	-0.63558	-3.18014	0.004623	-1.95612	XR_008866	Rn.14163
A_44_P139291	-0.63599	-3.59259	0.001776	-1.15524	XM_001058939	Rn.153570
A_44_P791631	-0.636117	-3.35283	0.003104	-1.62293	XM_227139	Rn.11681
A_44_P473472	-0.63614	-3.2489	0.003947	-1.82391	BC091233	Rn.9497
A_44_P760606	-0.63755	-3.49997	0.002205	-1.33648	XR_007237	Rn.197475
A_42_P521902	-0.63773	-3.92036	0.000822	-0.51086	NM_001079941	Rn.95230

A_44_P345101	-0.63791	-3.08645	0.005728	-2.1351	XM_217367	Rn.204758
A_44_P178179	-0.63841	-2.9104	0.008534	-2.46725	XM_226561	Rn.23325
A_44_P508750	-0.63843	-3.16079	0.004833	-1.9932	XM_001056358	Rn.155456
A_42_P707937	-0.63908	-4.18089	0.000445	0.00175	NM_080583	Rn.56138
A_44_P1007105	-0.63928	-3.20662	0.00435	-1.90528	NM_138905	Rn.12038
A_43_P22124	-0.63968	-3.16554	0.004781	-1.98409	NM_213625	Rn.92155
A_44_P859113	-0.64021	-3.1886	0.004534	-1.93988	NM_199463	Rn.32539
A_44_P148695	-0.64055	-3.87671	0.000911	-0.59683	XM_240915	Rn.64258
A_43_P12462	-0.64129	-3.49666	0.002222	-1.34294	NM_030863	Rn.2762
A_44_P383199	-0.64138	-3.4322	0.002582	-1.46866	XM_214649	Rn.12969
A_44_P267595	-0.64173	-2.97495	0.007378	-2.34616	XM_233839	Rn.22926
A_44_P323882	-0.642	-3.33145	0.003262	-1.66439	NM_001013073	Rn.48897
A_44_P477889	-0.64209	-3.19738	0.004444	-1.92303	NM_031783	Rn.18568
A_44_P523112	-0.64314	-3.30012	0.003507	-1.72502	XM_232937	Rn.154631
A_44_P840751	-0.64365	-3.55707	0.00193	-1.22482		
A_43_P11938	-0.64367	-2.98574	0.0072	-2.32583	NM_017364	Rn.98773
A_44_P340839	-0.64374	-3.18645	0.004557	-1.944	XM_001055446	Rn.19439
A_43_P17983	-0.64498	-3.02279	0.006621	-2.2559	XM_342679	Rn.68078
A_44_P1054864	-0.64518	-3.48096	0.002305	-1.37359	NM_001007662	Rn.2185
A_44_P825644	-0.64519	-3.27638	0.003705	-1.7709		
A_44_P369701	-0.64608	-3.33381	0.003244	-1.65981	NM_001025660	Rn.6593
A_44_P152395	-0.64643	-2.89866	0.008762	-2.48917	NM_001013222	Rn.198250
A_44_P365882	-0.64695	-3.24269	0.004004	-1.83588	NM_001014046	Rn.18772
A_44_P177678	-0.64722	-2.99464	0.007057	-2.30906	CB547239	Rn.205856
A_44_P375613	-0.64727	-3.01873	0.006682	-2.26356	NM_001013183	Rn.14772
A_44_P235336	-0.64853	-3.54983	0.001962	-1.23899	NM_001083966	Rn.8189
A_44_P438478	-0.64857	-2.84789	0.009817	-2.58366	XR_006258	Rn.197053
A_44_P523924	-0.64863	-2.89466	0.008841	-2.49664	NM_001007689	Rn.1274
A_44_P449969	-0.64889	-3.7702	0.001171	-0.80646	NM_001009258	Rn.15515
A_43_P17172	-0.64899	-4.24465	0.000382	0.12693	XM_213385	Rn.106432
A_44_P514539	-0.64943	-3.06895	0.005961	-2.16837	NM_001008324	Rn.95954
A_44_P442941	-0.64956	-3.2948	0.00355	-1.73531	NM_032067	Rn.7107
A_43_P18744	-0.65011	-3.42375	0.002633	-1.48511	NM_001012744	Rn.198838
A_44_P443343	-0.65059	-3.64924	0.001555	-1.04416	XM_216091	Rn.18101
A_44_P137262	-0.65064	-3.24492	0.003983	-1.83158	NM_148891	Rn.830
A_44_P366056	-0.6509	-3.71162	0.001343	-0.92163	XM_233418	Rn.162094
A_44_P395709	-0.6513	-3.10766	0.005458	-2.0947	NM_134366	Rn.29157
A_44_P422063	-0.65167	-3.03302	0.006469	-2.23652	XM_228173	Rn.27865
A_43_P17344	-0.65206	-3.6268	0.001639	-1.08818	XM_232252	Rn.103700
A_44_P555261	-0.65208	-2.8993	0.008749	-2.48799	NM_031057	Rn.2098
A_43_P12591	-0.65239	-4.09578	0.000543	-0.16555	NM_031569	Rn.9762
A_44_P441434	-0.6525	-3.29659	0.003536	-1.73185	NM_131914	Rn.81070
A_44_P248111	-0.65291	-3.16463	0.004791	-1.98584	NM_001047107	Rn.198138
A_44_P794608	-0.65716	-2.88473	0.00904	-2.51516	AI112975	Rn.166643
A_44_P594969	-0.65741	-4.05414	0.0006	-0.24748		
A_43_P18497	-0.65771	-3.58089	0.001825	-1.17817	NM_001007609	Rn.163119
A_43_P13344	-0.65785	-2.9207	0.008338	-2.44799	NM_138976	Rn.160939
A_44_P272294	-0.65802	-3.85068	0.000969	-0.64807	XM_233937	Rn.153991
A_44_P255341	-0.65803	-3.27778	0.003693	-1.76819	XM_222661	Rn.198415
A_44_P342314	-0.65816	-2.87154	0.009311	-2.53971	NM_001014232	Rn.8173
A_43_P18271	-0.65821	-3.09993	0.005555	-2.10943	XM_576264	Rn.99794
A_44_P466827	-0.6585	-2.98158	0.007269	-2.33369	NM_001034835	Rn.34623
A_44_P233867	-0.659	-3.29424	0.003555	-1.7364	NM_172067	Rn.7546
A_44_P1007438	-0.65937	-3.23016	0.004121	-1.86001	NM_001044238	Rn.138410
A_44_P215210	-0.65958	-3.21472	0.00427	-1.8897	XM_342568	Rn.198665
A_44_P166216	-0.65964	-3.20287	0.004388	-1.91248	NM_001013059	Rn.59254
A_44_P886861	-0.65992	-3.45001	0.002477	-1.43395	NM_001024245	Rn.154291
A_44_P170710	-0.66004	-2.94659	0.007866	-2.39947	NM_013060	Rn.3272

A_44_P344397	-0.66131	-2.96792	0.007497	-2.3594	NM_001024233	Rn.7405
A_43_P17012	-0.66174	-3.26322	0.003819	-1.7963	NM_001014227	Rn.24630
A_44_P667977	-0.66311	-3.18193	0.004604	-1.95268	XR_007894	Rn.216140
A_44_P902742	-0.66332	-3.81164	0.001062	-0.72491	XM_343576	Rn.8070
A_42_P826202	-0.66357	-3.00449	0.006901	-2.29047	NM_153732	Rn.64641
A_44_P147702	-0.66364	-3.29206	0.003573	-1.74061	XM_340853	Rn.145141
A_43_P12487	-0.66408	-3.57566	0.001847	-1.18842	NM_031040	Rn.10409
A_44_P324042	-0.6641	-3.21721	0.004246	-1.88492	NM_001034150	Rn.18349
A_44_P477927	-0.66518	-3.33075	0.003267	-1.66574	NM_013225	Rn.10644
A_44_P1026688	-0.66549	-3.03185	0.006486	-2.23875	NM_001014263	Rn.17193
A_44_P552977	-0.66572	-3.28585	0.003625	-1.75261	NM_001007693	Rn.162477
A_44_P175751	-0.66592	-3.05042	0.006218	-2.20356	XR_009314	Rn.137473
A_44_P352268	-0.666	-4.14352	0.000486	-0.07168	NM_031821	Rn.12100
A_44_P241439	-0.66602	-3.28469	0.003634	-1.75485	NM_001024789	Rn.43670
A_44_P288691	-0.66628	-2.91154	0.008512	-2.46511		
A_44_P475371	-0.66667	-3.21003	0.004316	-1.89873	XM_342812	Rn.106758
A_44_P334195	-0.66668	-3.24699	0.003964	-1.8276	NM_001007624	Rn.98697
A_44_P175461	-0.66704	-3.57929	0.001832	-1.18131	XM_239761	Rn.22866
A_43_P12936	-0.66754	-2.87059	0.009331	-2.54149	NM_053801	Rn.46850
A_44_P485453	-0.66763	-2.89374	0.008859	-2.49835	XM_001063164	
A_44_P744519	-0.66792	-4.19457	0.00043	0.028632	NM_012992	Rn.54537
A_42_P654739	-0.66826	-3.25711	0.003873	-1.80808	XM_221888	Rn.53030
A_44_P208880	-0.6684	-3.03326	0.006465	-2.23608	NM_001038595	Rn.198254
A_44_P476107	-0.66859	-3.30152	0.003496	-1.72232	XM_217278	
A_44_P217590	-0.66867	-3.97294	0.000726	-0.40733	NM_053794	Rn.27409
A_44_P199661	-0.66944	-3.01763	0.006699	-2.26564	XM_341464	Rn.1987
A_44_P632544	-0.66951	-3.08227	0.005783	-2.14306	AA944304	Rn.22753
A_44_P231556	-0.66977	-4.01993	0.00065	-0.31481	NM_001077589	Rn.202892
A_44_P658594	-0.67014	-4.42073	0.000253	0.471549	CO575464	Rn.13909
A_44_P269596	-0.6713	-3.05342	0.006176	-2.19787	XM_344976	Rn.16918
A_44_P328302	-0.67259	-3.41861	0.002665	-1.49513	XM_213992	Rn.44333
A_44_P473322	-0.67266	-3.06011	0.006082	-2.18516	XM_224778	
A_44_P293944	-0.67267	-2.95414	0.007733	-2.3853	NM_001005908	Rn.98375
A_44_P775787	-0.67334	-3.84582	0.00098	-0.65763	XM_575783	Rn.9543
A_44_P281959	-0.6734	-3.84436	0.000983	-0.66052	XM_218006	Rn.2666
A_44_P421391	-0.67373	-2.90785	0.008583	-2.47202	NM_198763	Rn.214724
A_44_P414757	-0.6745	-3.1099	0.00543	-2.09044	NM_001024770	Rn.204678
A_44_P521893	-0.67463	-3.16286	0.00481	-1.98922	BG669130	Rn.107945
A_43_P11734	-0.67487	-2.90785	0.008583	-2.47201	NM_013145	Rn.11391
A_42_P814410	-0.67517	-2.9877	0.007169	-2.32214	NM_212500	Rn.35325
A_44_P175681	-0.6754	-3.1213	0.00529	-2.06868	NM_001014011	Rn.53932
A_44_P492635	-0.67561	-3.10728	0.005462	-2.09543	NM_001008281	Rn.101332
A_44_P243102	-0.67704	-2.92309	0.008294	-2.44351	NM_017007	Rn.91245
A_44_P172941	-0.67789	-3.05037	0.006219	-2.20364	NM_182821	Rn.86175
A_43_P20530	-0.67809	-3.06014	0.006082	-2.18511	XM_231400	Rn.203614
A_44_P482200	-0.67851	-3.31217	0.003411	-1.70172	XM_222785	Rn.28132
A_44_P112546	-0.67921	-2.88545	0.009026	-2.51382	CO400633	Rn.45933
A_44_P182555	-0.68121	-2.90844	0.008571	-2.4709	NM_138509	Rn.7652
A_44_P375322	-0.68151	-2.91478	0.00845	-2.45907	XM_001074519	Rn.100109
A_44_P338095	-0.6816	-3.16288	0.00481	-1.98919	NM_001013112	Rn.8745
A_44_P525482	-0.68227	-3.13366	0.005143	-2.04508	CB546704	Rn.110783
A_44_P300612	-0.68367	-3.20786	0.004338	-1.9029	NM_022850	Rn.10076
A_42_P712718	-0.68384	-3.08844	0.005702	-2.13131	NM_001004280	Rn.3269
A_43_P19662	-0.68386	-3.23572	0.004069	-1.8493	CB547048	Rn.11982
A_44_P121457	-0.68392	-3.20492	0.004367	-1.90854	NM_001037778	Rn.25812
A_44_P340490	-0.68409	-3.11358	0.005384	-2.08342	XM_221232	Rn.198882
A_44_P104679	-0.68485	-3.08117	0.005798	-2.14515	NM_053481	Rn.44268
A_43_P18706	-0.6849	-3.24369	0.003995	-1.83396	NM_001012103	Rn.4118

A_44_P686038	-0.68623	-4.12732	0.000504	-0.10352	NM_022198	Rn.44406
A_44_P509099	-0.68629	-3.26147	0.003834	-1.79967	NM_001008382	Rn.203142
A_44_P248598	-0.68654	-5.19967	4.10E-05	1.963993	NM_001017459	Rn.162942
A_44_P332041	-0.68664	-2.84127	0.009963	-2.59593	NM_022385	Rn.3065
A_44_P525827	-0.68677	-3.24407	0.003991	-1.83322	NM_199091	Rn.203147
A_44_P324066	-0.68706	-3.24282	0.004003	-1.83563	XM_001072853	Rn.8212
A_44_P149734	-0.68795	-3.11878	0.005321	-2.0735	NM_001009657	Rn.139751
A_44_P466041	-0.68804	-3.64423	0.001573	-1.05397	NM_001007682	Rn.163276
A_43_P11763	-0.68995	-3.65196	0.001545	-1.0388	Z49081	Rn.5788
A_44_P466565	-0.69036	-3.12991	0.005187	-2.05224	XM_001058018	Rn.108042
A_44_P959355	-0.69051	-3.60627	0.00172	-1.12845	XR_008469	Rn.196073
A_44_P636077	-0.69106	-3.53297	0.002041	-1.27198	XM_225093	Rn.204848
A_44_P192568	-0.69135	-3.13713	0.005102	-2.03846	NM_172335	Rn.98783
A_44_P321605	-0.69153	-2.84277	0.00993	-2.59314	XM_576504	
A_44_P715184	-0.69258	-3.30086	0.003501	-1.72359	XM_215883	Rn.41581
A_44_P557979	-0.69268	-3.5054	0.002177	-1.32586	NM_001012190	Rn.98217
A_44_P285741	-0.69275	-2.84028	0.009985	-2.59777	NM_031556	Rn.22518
A_44_P128629	-0.69321	-3.00767	0.006852	-2.28447	XM_344744	Rn.99039
A_43_P18261	-0.6933	-3.51756	0.002116	-1.30209	NM_001008345	Rn.41045
A_44_P356538	-0.69375	-3.48274	0.002295	-1.37011	XM_341666	Rn.137414
A_43_P16735	-0.69432	-2.9709	0.007446	-2.35379	XM_340886	Rn.21931
A_44_P426707	-0.69439	-2.84496	0.009882	-2.5891	XM_001053199	
A_44_P370502	-0.69542	-2.84442	0.009894	-2.5901	XM_342445	Rn.214587
A_44_P516017	-0.69597	-3.02013	0.006661	-2.26093	NM_001031648	Rn.92713
A_44_P234362	-0.69624	-4.21969	0.000406	0.077943	NM_001007745	Rn.15201
A_44_P781559	-0.69644	-3.06049	0.006077	-2.18444	XM_233944	Rn.165307
A_44_P543350	-0.69678	-4.25814	0.00037	0.153383	NM_017063	Rn.11061
A_44_P371823	-0.69746	-3.06606	0.006001	-2.17387	XM_242940	Rn.23309
A_44_P471818	-0.69774	-3.29467	0.003551	-1.73557	XM_001071384	Rn.199044
A_44_P405679	-0.69778	-2.87125	0.009317	-2.54026	XM_233609	Rn.18036
A_43_P16946	-0.69781	-2.98551	0.007204	-2.32628	XM_213649	Rn.105738
A_44_P509473	-0.6985	-3.14203	0.005045	-2.02908	NM_139060	Rn.8046
A_44_P503673	-0.69915	-3.06187	0.006058	-2.18183	XM_341100	Rn.24509
A_44_P386748	-0.70208	-2.87533	0.009233	-2.53267	NM_001007616	Rn.4063
A_43_P20777	-0.70238	-2.87859	0.009165	-2.5266	XM_341474	Rn.27691
A_43_P15781	-0.70269	-2.84183	0.009951	-2.59489	BC090029	Rn.90191
A_44_P345009	-0.70403	-3.01838	0.006687	-2.26423	NM_001008526	Rn.76368
A_43_P16640	-0.70418	-3.03733	0.006406	-2.22837	XM_343298	Rn.4204
A_44_P822082	-0.70432	-3.16733	0.004761	-1.98067	XR_009104	Rn.2946
A_44_P648671	-0.70677	-3.03278	0.006472	-2.23699	XM_001076507	Rn.45013
A_44_P928907	-0.7069	-4.03553	0.000626	-0.2841	XM_574241	Rn.215820
A_44_P229079	-0.70706	-3.30973	0.00343	-1.70644	XM_344434	Rn.55115
A_44_P510470	-0.70706	-2.91863	0.008377	-2.45186	NM_053487	Rn.14519
A_44_P278923	-0.70774	-5.03147	6.05E-05	1.64738	XM_224623	Rn.3785
A_44_P132822	-0.70776	-3.82244	0.001035	-0.70366	NM_001005265	Rn.5825
A_44_P110098	-0.70832	-3.78825	0.001122	-0.77093	NM_001080783	Rn.46305
A_44_P260850	-0.70843	-3.20167	0.0044	-1.91479	CO394432	Rn.202175
A_44_P206520	-0.70932	-3.22865	0.004135	-1.86292	XM_217385	Rn.102571
A_44_P429364	-0.71011	-3.87808	0.000908	-0.59411	NM_001012113	Rn.203301
A_44_P135339	-0.71013	-3.38129	0.002906	-1.56768	NM_175761	Rn.119867
A_44_P459851	-0.71035	-3.73373	0.001275	-0.87818	XM_340974	Rn.84362
A_44_P442873	-0.71044	-4.17651	0.000449	-0.00684	NM_053739	Rn.2776
A_44_P535400	-0.71076	-2.89118	0.00891	-2.50313	AW916612	Rn.101953
A_44_P463996	-0.7109	-3.91058	0.000841	-0.53013	XM_344971	Rn.201738
A_44_P637889	-0.71114	-3.74526	0.001241	-0.85551		
A_44_P271368	-0.71186	-3.16018	0.00484	-1.99436	AW917142	Rn.12035
A_44_P494644	-0.71248	-3.00452	0.006901	-2.29042	NM_001012075	Rn.161747
A_44_P365653	-0.71364	-3.09375	0.005634	-2.12121	NM_001014161	Rn.3490

A_44_P222779	-0.71394	-4.11081	0.000525	-0.13599	XM_342937	Rn.27338
A_44_P543288	-0.71455	-3.3429	0.003176	-1.64219	NM_031704	Rn.5782
A_42_P724600	-0.71565	-3.24569	0.003976	-1.83009	NM_001006976	Rn.22757
A_44_P281540	-0.71569	-4.06235	0.000588	-0.23133	XM_215428	Rn.44172
A_44_P329139	-0.71569	-3.02789	0.006545	-2.24624	XM_224588	Rn.11687
A_43_P17974	-0.71659	-3.09709	0.005591	-2.11486	XM_341688	Rn.107745
A_44_P295151	-0.71685	-3.89589	0.000871	-0.55906	XM_224561	Rn.1761
A_44_P403209	-0.7176	-3.13424	0.005136	-2.04398	NM_001013073	Rn.48897
A_43_P19299	-0.71838	-4.52062	0.0002	0.666171	XM_235571	Rn.207175
A_44_P323430	-0.7185	-3.38173	0.002903	-1.56684	NM_138532	Rn.82718
A_44_P128447	-0.71917	-3.34909	0.003131	-1.63019	NM_001007680	Rn.15395
A_44_P229618	-0.71918	-3.94837	0.000769	-0.4557	XM_228065	Rn.46413
A_44_P187789	-0.71996	-2.94849	0.007832	-2.3959	XM_001066818	Rn.98517
A_44_P474509	-0.72016	-3.81817	0.001046	-0.71206	CO394478	Rn.163525
A_44_P389212	-0.72065	-2.94181	0.007951	-2.40844	NM_001024975	Rn.103625
A_44_P887061	-0.7207	-2.97887	0.007313	-2.33879	BC107438	
A_44_P260644	-0.72127	-3.67213	0.001474	-0.9992	XM_576264	Rn.99794
A_44_P437641	-0.72133	-3.55864	0.001922	-1.22174	NM_001007702	Rn.13725
A_44_P366177	-0.72167	-3.20233	0.004393	-1.91351	DN933141	Rn.102946
A_43_P17527	-0.72211	-3.09015	0.00568	-2.12807	NM_001024261	Rn.159746
A_44_P250983	-0.72229	-3.89095	0.000881	-0.56878	XM_001057073	Rn.198238
A_44_P124294	-0.72267	-3.2203	0.004216	-1.87898	NM_001012003	Rn.75605
A_44_P538531	-0.72287	-3.37055	0.002979	-1.58855	NM_053742	Rn.2399
A_44_P424092	-0.72288	-3.01236	0.006779	-2.27561	NM_001025669	Rn.3660
A_44_P189909	-0.72397	-3.50545	0.002177	-1.32577	NM_001025020	Rn.162709
A_44_P607982	-0.72405	-2.85647	0.009631	-2.56773		
A_44_P210461	-0.72422	-3.52168	0.002096	-1.29405	NM_017025	Rn.107896
A_44_P227121	-0.72543	-4.45355	0.000234	0.535574	XM_341301	Rn.16701
A_44_P356322	-0.72578	-3.61809	0.001673	-1.10525	XM_219482	Rn.145015
A_44_P126131	-0.72618	-3.58859	0.001792	-1.16308	XM_341215	Rn.24535
A_44_P827919	-0.72647	-2.85465	0.00967	-2.5711		
A_42_P521609	-0.72696	-3.50752	0.002166	-1.32173	XM_221635	Rn.17121
A_43_P12890	-0.72708	-3.27977	0.003676	-1.76436	NM_053665	Rn.163146
A_44_P126310	-0.72742	-3.1213	0.00529	-2.06868	NM_001005537	Rn.19642
A_44_P438442	-0.72806	-3.06307	0.006042	-2.17955	XM_001069431	Rn.32026
A_44_P197041	-0.72808	-3.6864	0.001425	-0.97119	NM_001035221	Rn.1136
A_44_P320894	-0.7285	-3.31887	0.003358	-1.68875	NM_013090	Rn.31977
A_44_P854406	-0.729	-2.96613	0.007527	-2.36277		
A_44_P400112	-0.72918	-3.00103	0.006955	-2.297	CO384551	Rn.96238
A_44_P326124	-0.72969	-3.37839	0.002926	-1.57333	XM_001056105	
A_44_P154367	-0.73034	-3.22323	0.004187	-1.87334	NM_001024744	Rn.66098
A_44_P530424	-0.73208	-3.18087	0.004615	-1.95471	BC085931	Rn.2639
A_44_P116846	-0.73271	-4.86812	8.84E-05	1.336596	XM_341384	Rn.162241
A_44_P250412	-0.73329	-3.3408	0.003192	-1.64627	NM_001007713	Rn.204023
A_43_P13047	-0.73513	-3.35038	0.003122	-1.6277	NM_057148	Rn.98570
A_44_P103160	-0.73566	-3.57859	0.001835	-1.18268	XM_342909	Rn.2252
A_43_P11514	-0.73639	-3.44909	0.002482	-1.43576	NM_012678	Rn.108199
A_44_P119007	-0.73665	-3.5243	0.002083	-1.28892	NM_172321	Rn.9716
A_44_P729866	-0.73771	-3.73729	0.001265	-0.87117	CA511309	Rn.179256
A_44_P300506	-0.73803	-3.9381	0.000788	-0.47594	NM_022399	Rn.974
A_44_P656856	-0.73846	-2.93043	0.008158	-2.42976	CK596682	Rn.14625
A_44_P557962	-0.73918	-3.47445	0.00234	-1.38629	XM_232602	
A_44_P520190	-0.73951	-2.98168	0.007267	-2.33349	NM_134414	Rn.2869
A_44_P443030	-0.73953	-3.14476	0.005014	-2.02386	XM_223227	Rn.27798
A_44_P525645	-0.73972	-3.63108	0.001623	-1.07978	XM_220442	Rn.214179
A_44_P550581	-0.73976	-3.66198	0.001509	-1.01913	XM_218411	Rn.130535
A_44_P434216	-0.74031	-3.18205	0.004603	-1.95244	NM_001012193	Rn.24910
A_43_P12932	-0.74065	-3.38875	0.002856	-1.5532	NM_053788	Rn.9943

A_44_P233736	-0.74102	-3.195	0.004468	-1.9276	NM_001004090	Rn.98240
A_44_P489120	-0.74147	-2.9228	0.008299	-2.44406	NM_001007701	Rn.104193
A_44_P440633	-0.74154	-3.84263	0.000987	-0.66392	NM_031639	Rn.10238
A_44_P415412	-0.74256	-3.12698	0.005222	-2.05785	NM_001004252	Rn.17054
A_44_P445318	-0.74296	-3.41425	0.002692	-1.5036	XM_215985	Rn.42890
A_43_P15432	-0.74297	-3.35463	0.003091	-1.61945	NM_053910	Rn.10672
A_44_P1015001	-0.74347	-2.898	0.008775	-2.49041	U50842	Rn.99540
A_44_P1014311	-0.74378	-3.27494	0.003717	-1.77369	XM_343619	Rn.101734
A_44_P248484	-0.7439	-3.26738	0.003782	-1.78828	XM_001069080	Rn.78670
A_44_P312484	-0.74397	-3.50663	0.002171	-1.32346	XM_213426	Rn.2567
A_44_P405793	-0.7458	-3.91347	0.000835	-0.52443	XM_244089	Rn.208665
A_44_P473754	-0.74689	-3.60769	0.001714	-1.12566	XM_221656	Rn.7967
A_43_P18072	-0.74696	-3.72996	0.001287	-0.88558	NM_001013413	Rn.6408
A_44_P714315	-0.74724	-3.47339	0.002346	-1.38836	CO404950	Rn.45255
A_43_P18263	-0.7503	-3.09377	0.005633	-2.12117	NM_001025421	Rn.203134
A_44_P760403	-0.75037	-3.85965	0.000948	-0.6304		
A_44_P454177	-0.75055	-3.0849	0.005748	-2.13805	NM_182675	Rn.2496
A_43_P20387	-0.75055	-3.65848	0.001522	-1.02602	XM_232614	Rn.6834
A_44_P853268	-0.75101	-4.47339	0.000223	0.574235	CO393484	Rn.203091
A_43_P16469	-0.75132	-3.0425	0.006331	-2.21857	NM_001083313	Rn.6239
A_43_P10226	-0.75144	-2.84782	0.009819	-2.58379	XM_236333	Rn.26984
A_44_P159569	-0.75145	-4.04903	0.000607	-0.25754	NM_001039587	Rn.11823
A_44_P330332	-0.75155	-3.28325	0.003646	-1.75762	BC129128	Rn.20
A_44_P229800	-0.75432	-2.86797	0.009386	-2.54636	NM_001011991	Rn.153992
A_43_P15423	-0.75444	-4.13143	0.0005	-0.09545	NM_019239	Rn.9803
A_44_P806641	-0.75496	-3.09707	0.005591	-2.11488	XM_236024	
A_44_P187293	-0.75558	-3.01753	0.0067	-2.26585	XM_344175	
A_44_P286719	-0.75658	-3.20694	0.004347	-1.90466	XM_224863	Rn.16962
A_44_P321686	-0.75683	-3.03412	0.006453	-2.23444	XM_001060299	Rn.212953
A_44_P270510	-0.75737	-3.10968	0.005432	-2.09086	XM_220420	Rn.17594
A_44_P203842	-0.75798	-3.07948	0.00582	-2.14836	BP485454	Rn.216026
A_44_P145416	-0.75854	-3.25682	0.003876	-1.80865	NM_001007646	Rn.105976
A_44_P775952	-0.75929	-3.59058	0.001784	-1.15919		
A_44_P482512	-0.75967	-3.93524	0.000794	-0.48157	XM_343513	Rn.1423
A_44_P548303	-0.76019	-3.15577	0.004889	-2.0028	NM_001009688	Rn.12345
A_44_P375252	-0.76043	-3.64515	0.00157	-1.05217	XM_342379	Rn.7645
A_44_P838313	-0.76068	-3.83701	0.001	-0.67497		
A_43_P17880	-0.76267	-4.34422	0.000302	0.322027	NM_182953	Rn.18744
A_44_P545635	-0.76267	-3.92659	0.00081	-0.4986	AY325140	Rn.208293
A_44_P541378	-0.76295	-4.38151	0.000277	0.394951	NM_001014242	Rn.1166
A_44_P475159	-0.76295	-3.58374	0.001813	-1.17259	NM_012673	Rn.108198
A_44_P261818	-0.76305	-3.20759	0.004341	-1.90342	BC087666	Rn.8161
A_44_P806713	-0.76383	-4.22581	0.0004	0.089969		
A_44_P1020219	-0.7645	-3.33879	0.003207	-1.65017	CO574509	Rn.162190
A_44_P439573	-0.76454	-4.60261	0.000165	0.825362	DV728042	Rn.1619
A_43_P12453	-0.76465	-3.07191	0.005921	-2.16275	NM_030839	Rn.204880
A_44_P253601	-0.76599	-3.71387	0.001336	-0.91722	NM_001025009	Rn.2495
A_44_P203793	-0.76667	-3.47191	0.002354	-1.39126	NM_001013912	Rn.144837
A_44_P172936	-0.76738	-2.84087	0.009972	-2.59667	NM_138613	Rn.7544
A_44_P229670	-0.76753	-2.92229	0.008309	-2.44501	XM_001075438	Rn.109840
A_44_P451014	-0.76848	-3.01906	0.006677	-2.26294	NM_017322	Rn.9910
A_44_P203665	-0.76852	-3.18739	0.004547	-1.94221	XM_223270	Rn.162246
A_44_P550454	-0.76885	-4.09248	0.000548	-0.17204		
A_44_P213320	-0.76886	-2.99317	0.00708	-2.31183	XM_236873	Rn.198381
A_44_P852634	-0.76985	-3.63252	0.001617	-1.07696	XR_007953	Rn.197347
A_44_P961330	-0.77033	-3.17022	0.00473	-1.97514		
A_44_P351789	-0.77082	-3.08008	0.005812	-2.14723	XM_341509	Rn.20096
A_44_P134405	-0.77089	-3.17456	0.004683	-1.96681	NM_031798	Rn.11523

A_44_P545727	-0.77122	-3.64864	0.001557	-1.04533	XM_341086	Rn.16272
A_44_P236656	-0.77127	-3.79641	0.001101	-0.75489	XM_343817	Rn.98982
A_44_P832837	-0.77152	-2.94154	0.007956	-2.40895	AW143397	
A_44_P230410	-0.77156	-2.93941	0.007994	-2.41294	NM_031600	Rn.11044
A_44_P363204	-0.77167	-3.00867	0.006836	-2.28258	NM_053824	Rn.4231
A_44_P274397	-0.7718	-3.24276	0.004003	-1.83574	NM_001033700	Rn.1448
A_43_P17671	-0.7721	-3.7467	0.001237	-0.85268	NM_001014186	Rn.137519
A_44_P150089	-0.7723	-3.62236	0.001656	-1.09689	NM_001013229	Rn.99076
A_44_P671470	-0.7723	-3.19848	0.004433	-1.92092		
A_44_P459301	-0.77262	-2.98892	0.007149	-2.31985	XM_219377	Rn.162468
A_44_P377645	-0.77345	-4.79188	0.000106	1.190514	XM_342774	
A_44_P287250	-0.77376	-2.91751	0.008399	-2.45396	NM_022521	Rn.1430
A_44_P538538	-0.77411	-2.90255	0.008686	-2.48191	NM_017357	Rn.34529
A_44_P250030	-0.77556	-2.87185	0.009305	-2.53915	NM_013107	Rn.40476
A_44_P142562	-0.77634	-3.36026	0.003051	-1.60853	NM_001007643	Rn.3027
A_44_P215689	-0.77949	-2.98835	0.007158	-2.32092	XM_343744	
A_44_P725617	-0.7797	-3.64065	0.001587	-1.06102	BF548107	Rn.162539
A_44_P138363	-0.77975	-2.88998	0.008934	-2.50537	XM_344057	Rn.14920
A_44_P184279	-0.78011	-2.92214	0.008311	-2.44529	NM_012504	Rn.2992
A_43_P17443	-0.78041	-4.82551	9.77E-05	1.255038	XM_230647	Rn.34193
A_44_P157118	-0.78119	-3.19712	0.004446	-1.92353	BC089114	Rn.42737
A_44_P159455	-0.78271	-3.50529	0.002178	-1.32607	XM_001062352	
A_44_P208711	-0.78277	-2.88113	0.009113	-2.52186	XM_001075438	Rn.109840
A_44_P290703	-0.78279	-3.38015	0.002914	-1.5699	XM_226680	
A_44_P322500	-0.78291	-3.58783	0.001796	-1.16458	CK473706	Rn.22996
A_44_P100740	-0.7831	-3.11203	0.005403	-2.08638	NM_001024243	Rn.195252
A_44_P386515	-0.78324	-3.00668	0.006867	-2.28634	NM_001008771	Rn.14128
A_44_P322960	-0.78375	-4.69167	0.000134	0.997599	NM_022532	Rn.1714
A_44_P759589	-0.78386	-3.49734	0.002218	-1.34161	CF110322	Rn.164561
A_44_P452668	-0.78399	-4.04999	0.000605	-0.25566	XM_342731	Rn.203214
A_44_P356468	-0.78492	-3.45006	0.002477	-1.43386	XR_007829	Rn.155466
A_44_P283971	-0.78501	-4.45247	0.000234	0.53347	XM_232972	Rn.19954
A_43_P19994	-0.78518	-3.89703	0.000868	-0.5568	XM_233485	Rn.50631
A_43_P20118	-0.78579	-2.92516	0.008255	-2.43964	BC099746	Rn.4098
A_43_P21464	-0.78587	-3.0589	0.006099	-2.18746	XM_233220	Rn.213735
A_44_P382523	-0.78601	-2.86569	0.009434	-2.5506	XM_236376	Rn.163030
A_44_P344425	-0.78611	-3.23431	0.004082	-1.85202	XM_224538	Rn.10431
A_44_P1039444	-0.78639	-2.99522	0.007048	-2.30796	NM_032613	Rn.94195
A_44_P326090	-0.78652	-3.55564	0.001936	-1.22761	XM_342312	Rn.162625
A_44_P425380	-0.7868	-5.39361	2.62E-05	2.324366	NM_001011991	Rn.153992
A_44_P480240	-0.78696	-2.94627	0.007872	-2.40007	NM_019182	Rn.205059
A_44_P831218	-0.7889	-2.91452	0.008455	-2.45955	CK596627	Rn.161389
A_43_P23188	-0.78924	-3.02334	0.006612	-2.25485	XM_001053087	
A_44_P422271	-0.78954	-4.19664	0.000428	0.032702	XM_217019	Rn.99537
A_44_P825629	-0.78999	-3.15321	0.004918	-2.0077		
A_44_P966141	-0.79039	-3.19602	0.004458	-1.92563	CO399145	Rn.25185
A_44_P501082	-0.79087	-2.93503	0.008074	-2.42116	NM_134346	Rn.95071
A_44_P367698	-0.79136	-3.10955	0.005434	-2.09111	NM_031720	Rn.88380
A_43_P10511	-0.79225	-3.37342	0.00296	-1.58298	XM_342289	Rn.137428
A_44_P340393	-0.79238	-5.074	5.48E-05	1.72778	XR_009391	Rn.195880
A_44_P246128	-0.79279	-3.00167	0.006945	-2.2958	XM_576696	Rn.101054
A_44_P168102	-0.79296	-2.84876	0.009798	-2.58204	NM_031729	Rn.6107
A_43_P17837	-0.793	-3.20673	0.004349	-1.90507	XM_215423	Rn.22395
A_43_P18051	-0.79346	-3.58182	0.001821	-1.17636	XM_001054856	
A_44_P536676	-0.79376	-3.1337	0.005142	-2.045	NM_001013158	Rn.3666
A_44_P506374	-0.79424	-3.57209	0.001863	-1.19541	XM_001073596	Rn.133954
A_44_P553939	-0.79454	-3.31451	0.003392	-1.69718	BG667845	Rn.13677
A_44_P149954	-0.7946	-3.00072	0.00696	-2.2976	XM_576917	Rn.196493

A_44_P324452	-0.79464	-3.14623	0.004997	-2.02106	NM_001048184	Rn.6455
A_44_P1029770	-0.79519	-3.43116	0.002588	-1.4707	NM_001025732	Rn.145204
A_44_P485246	-0.7955	-4.50143	0.000209	0.628841	XR_006015	Rn.197769
A_43_P21256	-0.79594	-2.97218	0.007425	-2.35137	NM_001083966	Rn.8189
A_44_P764308	-0.79678	-3.49622	0.002224	-1.3438		
A_44_P1008284	-0.79708	-4.16045	0.000467	-0.0384		
A_44_P500250	-0.79737	-2.94713	0.007857	-2.39845	XM_343404	Rn.11883
A_44_P380157	-0.79748	-3.19337	0.004485	-1.93072	XM_001074009	Rn.145750
A_44_P539738	-0.79803	-2.99091	0.007117	-2.3161	NM_022862	Rn.10127
A_44_P268837	-0.80088	-3.40251	0.002766	-1.52644	NM_001013923	Rn.93910
A_44_P536282	-0.80115	-4.439	0.000242	0.507203	NM_001013130	Rn.93208
A_44_P506464	-0.80237	-3.36662	0.003007	-1.59619	XR_005489	Rn.196156
A_42_P505280	-0.80242	-3.49041	0.002254	-1.35513	NM_001012063	Rn.9014
A_43_P16672	-0.80295	-3.36842	0.002994	-1.59269	NM_001024800	Rn.1822
A_44_P331164	-0.80451	-3.20958	0.004321	-1.89959	NM_001013974	Rn.107772
A_44_P218229	-0.80511	-2.96274	0.007585	-2.36914	XR_006724	Rn.197351
A_44_P806917	-0.80515	-3.81591	0.001051	-0.71652		
A_44_P366005	-0.80517	-3.29426	0.003555	-1.73635	XR_008061	Rn.195662
A_44_P387374	-0.80534	-3.29148	0.003578	-1.74173	XM_221315	Rn.63502
A_44_P944991	-0.80573	-4.00033	0.000681	-0.3534		
A_44_P335898	-0.80598	-3.27264	0.003737	-1.77812	BC097982	Rn.48329
A_44_P871871	-0.80724	-4.61119	0.000161	0.841983		
A_44_P987960	-0.80876	-3.50306	0.002189	-1.33044	CO398009	Rn.25416
A_44_P365155	-0.80965	-3.15841	0.004859	-1.99775	NM_012583	Rn.47
A_44_P410080	-0.81097	-2.9771	0.007343	-2.34212	XM_001061719	Rn.7745
A_43_P13247	-0.811	-3.17927	0.004632	-1.95779	XM_001070141	Rn.64489
A_43_P10441	-0.81137	-3.53204	0.002046	-1.27378	XM_217149	Rn.137571
A_44_P558119	-0.81138	-3.19236	0.004495	-1.93267	XM_236476	Rn.7567
A_44_P612824	-0.81147	-3.03376	0.006458	-2.23512	NM_183326	Rn.28463
A_44_P524981	-0.81184	-3.10221	0.005526	-2.10509	NM_024373	Rn.10266
A_43_P13175	-0.81215	-3.3448	0.003163	-1.63851	NM_133405	Rn.212745
A_44_P177793	-0.81247	-2.91864	0.008377	-2.45185		
A_44_P165692	-0.81344	-3.86802	0.00093	-0.61393	NM_001034003	Rn.2352
A_44_P344421	-0.81448	-3.63381	0.001612	-1.07443	XM_224538	Rn.10431
A_44_P590197	-0.81577	-3.62257	0.001655	-1.09647	AW915353	Rn.4268
A_44_P419565	-0.81707	-3.76594	0.001182	-0.81483	XM_231128	Rn.17259
A_43_P10318	-0.81731	-2.84924	0.009787	-2.58115	XM_001062954	Rn.107561
A_44_P375593	-0.81746	-3.24267	0.004004	-1.83591	XM_001053401	Rn.159763
A_42_P658005	-0.81775	-3.92758	0.000808	-0.49666	XM_573442	Rn.60443
A_44_P821396	-0.81801	-3.13023	0.005183	-2.05164	XM_220013	Rn.17150
A_44_P297114	-0.8182	-3.83834	0.000997	-0.67237	NM_001014226	Rn.13255
A_43_P10712	-0.81831	-4.28674	0.000346	0.209449	CO385963	Rn.169246
A_44_P135148	-0.81887	-3.1113	0.005412	-2.08777	NM_130894	Rn.8570
A_43_P20941	-0.81895	-3.32789	0.003289	-1.67129	NM_001009685	Rn.102143
A_44_P114774	-0.81968	-3.25356	0.003905	-1.81493	XM_343190	Rn.7015
A_44_P231724	-0.8197	-2.92887	0.008186	-2.43269	XR_009038	Rn.196606
A_44_P287286	-0.82102	-3.21399	0.004277	-1.89111	XM_216910	Rn.140163
A_44_P589989	-0.82136	-3.05041	0.006218	-2.20358	CA339579	Rn.91712
A_42_P544887	-0.82314	-3.21152	0.004302	-1.89586	NM_031665	Rn.100217
A_44_P509872	-0.82419	-3.00105	0.006955	-2.29698	XM_238280	Rn.20506
A_43_P23313	-0.82456	-3.02399	0.006603	-2.25361	NM_001006960	Rn.106244
A_44_P637687	-0.8257	-3.21735	0.004244	-1.88466	XM_001079537	
A_44_P476598	-0.82636	-3.31993	0.00335	-1.6867	XM_575808	Rn.169839
A_44_P471848	-0.82645	-3.48049	0.002307	-1.3745	XM_225257	Rn.128652
A_44_P560788	-0.82684	-3.60226	0.001736	-1.13631	CO566111	Rn.114933
A_43_P18579	-0.82732	-3.20081	0.004409	-1.91645	XM_231162	Rn.103250
A_44_P507881	-0.82746	-3.06747	0.005981	-2.1712	AI502676	Rn.54645
A_44_P550412	-0.82778	-3.7411	0.001253	-0.86368	AF375874	Rn.86991

A_44_P455174	-0.83021	-3.97363	0.000725	-0.40598	NM_001014116	Rn.155261
A_44_P203682	-0.83053	-3.54434	0.001988	-1.24974	XM_215113	Rn.104893
A_44_P671835	-0.83057	-3.28807	0.003606	-1.74832		
A_44_P259574	-0.83064	-3.16576	0.004778	-1.98367	NM_053290	Rn.1383
A_44_P494510	-0.83076	-3.38952	0.002851	-1.5517	XM_001068808	Rn.155453
A_44_P166777	-0.83115	-3.00862	0.006837	-2.28267	XM_574528	Rn.122632
A_44_P920120	-0.83168	-3.4174	0.002672	-1.49748	XM_001066536	Rn.110441
A_44_P606516	-0.83203	-3.91695	0.000829	-0.51759		
A_44_P209817	-0.83242	-4.29276	0.000341	0.221246	BC078744	Rn.22518
A_43_P21088	-0.83259	-3.45525	0.002447	-1.42375	XM_229993	Rn.51147
A_44_P274491	-0.83289	-3.22701	0.004151	-1.86607	XM_341645	Rn.24338
A_44_P141513	-0.83354	-3.26832	0.003774	-1.78646	NM_001014175	Rn.15859
A_44_P123908	-0.83393	-2.89561	0.008822	-2.49487	XM_342338	Rn.1713
A_43_P18107	-0.83474	-3.44676	0.002496	-1.44029	XR_008372	Rn.36184
A_44_P432432	-0.83528	-3.47686	0.002327	-1.3816	NM_013151	Rn.107102
A_43_P10854	-0.8356	-2.94035	0.007977	-2.41117	NM_001011934	Rn.98198
A_44_P124225	-0.83573	-3.41754	0.002671	-1.49719	NM_001025701	Rn.3991
A_44_P852513	-0.83581	-3.89638	0.00087	-0.55809	XR_005712	Rn.216204
A_44_P883146	-0.83589	-3.64378	0.001575	-1.05487	NM_001007682	Rn.163276
A_43_P10831	-0.83625	-3.43918	0.00254	-1.45507	NM_001013207	Rn.8555
A_44_P662685	-0.83669	-3.50289	0.00219	-1.33076	NM_001025046	Rn.161821
A_44_P540542	-0.83697	-3.21521	0.004265	-1.88876	XM_001054154	Rn.1269
A_44_P720404	-0.83721	-3.23545	0.004071	-1.84982	XM_231564	Rn.8222
A_44_P963185	-0.83819	-3.67974	0.001448	-0.98427	AB014879	Rn.90013
A_44_P226881	-0.83954	-2.96361	0.00757	-2.36751	NM_031079	Rn.10044
A_44_P177807	-0.83999	-4.80135	0.000103	1.208701	NM_023090	Rn.55138
A_44_P511049	-0.84018	-3.65688	0.001527	-1.02915	XM_224947	Rn.1880
A_44_P974681	-0.84028	-3.58459	0.001809	-1.17093	XM_575362	Rn.195816
A_43_P17622	-0.84034	-4.4513	0.000235	0.531183	XM_345870	Rn.52275
A_44_P794598	-0.84037	-3.27501	0.003716	-1.77354	NM_198132	Rn.107690
A_44_P621980	-0.84087	-2.94899	0.007824	-2.39497	M31178	Rn.3908
A_44_P163645	-0.84116	-4.13063	0.000501	-0.09701	NM_001014135	Rn.3168
A_43_P10843	-0.84205	-3.08125	0.005797	-2.145	XM_342312	Rn.162625
A_44_P856158	-0.84209	-4.17989	0.000446	-0.0002	XM_001058383	Rn.172982
A_44_P680369	-0.84222	-3.99521	0.000689	-0.36349	BF545930	Rn.163742
A_44_P487562	-0.84318	-3.7762	0.001154	-0.79464	NM_001039023	Rn.3889
A_43_P15854	-0.8438	-3.411	0.002712	-1.50993	XM_227134	Rn.15040
A_44_P241862	-0.84419	-3.4067	0.002739	-1.51829	NM_022615	Rn.91572
A_44_P403415	-0.84445	-3.58254	0.001818	-1.17494	XM_343301	Rn.162000
A_44_P543548	-0.84493	-3.41833	0.002666	-1.49566	NM_057119	Rn.8538
A_44_P1039760	-0.84495	-2.87286	0.009284	-2.53726	NM_001013228	Rn.106023
A_44_P249164	-0.84596	-3.23384	0.004086	-1.85293	NM_031028	Rn.30059
A_43_P15265	-0.84787	-4.11875	0.000515	-0.12039	NM_017042	Rn.11063
A_44_P242781	-0.84849	-3.7436	0.001246	-0.85877	DY308836	Rn.167869
A_44_P501483	-0.85038	-4.03117	0.000633	-0.2927	XR_006667	Rn.216235
A_44_P475744	-0.8507	-3.12427	0.005254	-2.06301	XM_215524	Rn.22753
A_44_P151399	-0.85178	-3.33929	0.003203	-1.64919	NM_198737	Rn.3366
A_44_P112788	-0.85269	-4.04472	0.000613	-0.26603	NM_021850	Rn.44267
A_42_P693042	-0.85293	-3.66701	0.001492	-1.00926	XM_214555	Rn.209444
A_44_P932876	-0.85312	-3.07002	0.005947	-2.16634	NM_001014239	Rn.3663
A_42_P713975	-0.85318	-2.98626	0.007192	-2.32487	BC082799	Rn.30992
A_43_P10213	-0.85468	-3.32828	0.003286	-1.67052	XM_232515	Rn.7630
A_44_P323496	-0.85738	-3.58245	0.001818	-1.17512	NM_019311	Rn.10659
A_44_P322590	-0.85893	-3.21225	0.004295	-1.89447	NM_057211	Rn.19481
A_44_P243701	-0.85938	-2.99564	0.007041	-2.30718	XM_578871	Rn.195709
A_44_P753384	-0.8606	-3.64714	0.001563	-1.04827		
A_44_P319042	-0.86163	-4.0356	0.000626	-0.28396	XM_231798	Rn.1688
A_44_P342586	-0.86213	-3.47059	0.002361	-1.39383	NM_001014019	Rn.160642

A_44_P425367	-0.86303	-3.14314	0.005032	-2.02697	NM_001024332	Rn.6283
A_44_P759588	-0.86457	-4.15327	0.000475	-0.05251	CF110322	Rn.164561
A_44_P667768	-0.86515	-3.42155	0.002647	-1.48939		
A_44_P520324	-0.86534	-3.54747	0.001973	-1.2436	XM_213911	
A_44_P342657	-0.86777	-3.2654	0.0038	-1.79209	XM_233792	Rn.2644
A_44_P534374	-0.86788	-3.53105	0.00205	-1.27572	XM_227614	Rn.6718
A_44_P199420	-0.86915	-3.37253	0.002966	-1.5847	XM_235924	Rn.4026
A_42_P522171	-0.86972	-3.14713	0.004987	-2.01933	XM_001081512	
A_44_P930764	-0.87037	-3.62275	0.001655	-1.09612	NM_001013125	Rn.159939
A_44_P145549	-0.87039	-3.69008	0.001413	-0.96397	NM_019182	Rn.205059
A_44_P230875	-0.8704	-3.22381	0.004182	-1.87222	BF567478	Rn.985
A_42_P545928	-0.87106	-2.94139	0.007959	-2.40922	NM_153730	Rn.198416
A_44_P233569	-0.87169	-4.19053	0.000435	0.020694	NM_031770	Rn.23042
A_44_P198535	-0.87452	-4.60517	0.000164	0.830324	NM_057103	Rn.122094
A_44_P975286	-0.87515	-4.2714	0.000359	0.179399	CO403766	Rn.164897
A_44_P944912	-0.8768	-3.49984	0.002205	-1.33673		
A_44_P362541	-0.87682	-3.76685	0.00118	-0.81303	NM_001013235	Rn.18315
A_44_P283715	-0.87695	-3.04935	0.006233	-2.20559	XM_223664	Rn.3740
A_44_P219796	-0.87924	-3.30582	0.003461	-1.714	XM_230038	Rn.2611
A_44_P102628	-0.8802	-3.06102	0.00607	-2.18344	NM_021688	Rn.15693
A_44_P548507	-0.88214	-3.00044	0.006965	-2.29811	NM_032083	Rn.11166
A_44_P471440	-0.88288	-3.15969	0.004845	-1.9953	NM_001024765	Rn.94975
A_44_P476663	-0.88324	-3.8279	0.001022	-0.69292	XM_344277	Rn.214546
A_44_P465329	-0.8854	-4.56407	0.00018	0.7506	NM_012798	Rn.10174
A_44_P157167	-0.88618	-3.80928	0.001068	-0.72956	NM_001008324	Rn.95954
A_43_P17496	-0.88747	-2.91406	0.008464	-2.4604	XM_231176	Rn.2745
A_44_P137247	-0.89159	-3.49294	0.002241	-1.35021	NM_001025142	Rn.153980
A_44_P438744	-0.8925	-3.65382	0.001538	-1.03516	XM_233718	
A_44_P112050	-0.8936	-3.37157	0.002972	-1.58656	XM_573428	Rn.41420
A_44_P895660	-0.89381	-2.84273	0.009931	-2.59322	AW916954	Rn.162467
A_43_P17964	-0.89386	-3.76477	0.001186	-0.81713	NM_001007662	Rn.2185
A_44_P393929	-0.89431	-3.3768	0.002936	-1.57641	NM_001039713	Rn.195046
A_44_P207721	-0.89434	-3.0179	0.006695	-2.26513	XM_222946	Rn.9047
A_44_P267724	-0.89515	-2.88781	0.008978	-2.50941	XM_217022	Rn.52470
A_44_P512255	-0.89516	-3.05534	0.006149	-2.19423	NM_017214	Rn.11065
A_44_P354208	-0.89604	-2.9517	0.007776	-2.38988	XM_232315	Rn.29825
A_44_P860400	-0.89607	-3.36861	0.002993	-1.59232	CK839443	Rn.203309
A_44_P344273	-0.89797	-3.49454	0.002233	-1.34707	NM_001034129	Rn.98327
A_44_P948202	-0.89955	-4.87682	8.66E-05	1.35322		
A_44_P463759	-0.89962	-5.01509	6.28E-05	1.616357	XM_235609	Rn.141040
A_44_P667548	-0.89963	-3.17326	0.004697	-1.96929		
A_44_P918128	-0.9016	-3.47545	0.002334	-1.38434		
A_44_P233742	-0.90275	-3.92732	0.000809	-0.49716	NM_001004090	Rn.98240
A_44_P409694	-0.90372	-3.44939	0.002481	-1.43516	XM_343604	Rn.2271
A_44_P232642	-0.90572	-3.03096	0.006499	-2.24043	NM_001008301	Rn.11790
A_44_P121401	-0.90904	-4.05801	0.000594	-0.23986	XM_237998	Rn.64941
A_44_P356078	-0.91218	-3.89307	0.000877	-0.56461	NM_017033	Rn.9970
A_44_P157604	-0.91228	-3.11259	0.005396	-2.0853	DY315019	Rn.199546
A_44_P745191	-0.913	-3.37028	0.002981	-1.58908	CF978988	Rn.3502
A_43_P11416	-0.91374	-3.2102	0.004315	-1.89839	XM_225625	Rn.146822
A_44_P158973	-0.9141	-3.74856	0.001232	-0.84902	NM_001013046	Rn.18008
A_43_P21122	-0.9169	-4.00759	0.000669	-0.33911	XM_341882	Rn.7634
A_44_P552865	-0.91711	-3.37597	0.002942	-1.57802	XM_221896	Rn.25814
A_44_P147509	-0.91732	-4.39104	0.000271	0.413562	NM_199412	Rn.214252
A_43_P13702	-0.91972	-3.28359	0.003644	-1.75698	NM_001025136	Rn.113703
A_44_P492153	-0.91998	-3.24137	0.004016	-1.83841	XM_224271	Rn.11877
A_43_P10791	-0.92115	-3.65415	0.001537	-1.03451	CB547648	
A_43_P12189	-0.92265	-2.99137	0.007109	-2.31523	NM_022262	Rn.2315

A_44_P526933	-0.92267	-3.57335	0.001857	-1.19295	NM_053357	Rn.112601
A_43_P10856	-0.92277	-3.03779	0.006399	-2.22749	XM_001077761	Rn.205725
A_44_P415115	-0.92346	-3.28387	0.003641	-1.75643	XM_345660	
A_44_P221077	-0.92647	-2.98673	0.007184	-2.32398	NM_001017479	Rn.18850
A_44_P505932	-0.92758	-4.47811	0.000221	0.583443	NM_021868	Rn.107869
A_44_P469394	-0.92767	-4.01403	0.000659	-0.32643	NM_022217	Rn.44463
A_44_P409339	-0.93101	-3.07934	0.005822	-2.14864	NM_053357	Rn.112601
A_44_P621820	-0.93372	-3.50585	0.002175	-1.32498	XR_005838	Rn.196836
A_44_P349309	-0.93648	-2.94883	0.007826	-2.39526	XM_223327	Rn.66912
A_44_P269752	-0.93726	-3.11306	0.005391	-2.0844	XM_341581	Rn.163367
A_44_P261326	-0.93768	-3.27729	0.003697	-1.76914	XM_342782	Rn.1895
A_44_P278639	-0.93897	-3.35496	0.003089	-1.6188	NM_053693	Rn.116028
A_44_P210563	-0.93952	-3.25811	0.003864	-1.80615	NM_012578	Rn.3129
A_44_P443976	-0.9398	-3.2626	0.003824	-1.7975	NM_031697	Rn.44390
A_44_P868060	-0.94004	-4.31057	0.000327	0.256138	XR_005433	
A_44_P143974	-0.94095	-3.60331	0.001732	-1.13424	NM_001013235	Rn.18315
A_43_P21660	-0.94104	-4.6039	0.000164	0.827861	NM_001007147	Rn.100642
A_43_P18787	-0.9414	-3.52178	0.002095	-1.29385	XM_342552	Rn.9077
A_44_P115461	-0.94268	-2.99425	0.007063	-2.30979	NM_017007	Rn.91245
A_44_P410062	-0.94397	-3.62267	0.001655	-1.09627	XR_005977	Rn.197086
A_43_P17406	-0.94555	-3.56182	0.001908	-1.21551	NM_001005541	Rn.14968
A_44_P794770	-0.94696	-3.20786	0.004338	-1.9029	NM_133560	Rn.26957
A_44_P973796	-0.94702	-3.19587	0.004459	-1.92592	DY319575	Rn.165597
A_43_P10574	-0.94742	-4.25752	0.000371	0.15218	NM_001012012	Rn.13268
A_44_P164287	-0.94768	-4.14358	0.000485	-0.07156	NM_001013925	Rn.72440
A_44_P427089	-0.94834	-3.89974	0.000863	-0.55147	XM_341008	
A_44_P606344	-0.94897	-3.33258	0.003253	-1.66219	XM_343550	Rn.214678
A_44_P356876	-0.95063	-4.87652	8.67E-05	1.352647	NM_001039002	Rn.198270
A_44_P113842	-0.95164	-3.81968	0.001042	-0.70909	NM_022210	Rn.4210
A_44_P365516	-0.95207	-3.65613	0.00153	-1.03063	BC059146	Rn.3545
A_43_P10725	-0.95239	-3.04435	0.006304	-2.21506	NM_001012468	Rn.53248
A_43_P16458	-0.95617	-3.80122	0.001088	-0.74541	NM_001011992	Rn.32254
A_44_P433334	-0.95734	-3.66368	0.001503	-1.01581	NM_001012197	Rn.101146
A_44_P466489	-0.95739	-3.0822	0.005784	-2.14319	NM_001007642	Rn.1266
A_44_P413294	-0.9585	-3.15311	0.004919	-2.0079	NM_022864	Rn.10133
A_44_P288796	-0.95854	-3.64723	0.001562	-1.0481	NM_001013125	Rn.159939
A_44_P609842	-0.95905	-3.00601	0.006878	-2.28761		
A_44_P822078	-0.95962	-3.48448	0.002286	-1.36671	XM_576832	
A_44_P984248	-0.95963	-3.93127	0.000801	-0.48938	XM_222785	Rn.28132
A_42_P832417	-0.95987	-3.71298	0.001339	-0.91896	NM_012686	Rn.105934
A_44_P317520	-0.96009	-3.89718	0.000868	-0.55651	BC079339	Rn.12944
A_44_P286573	-0.96102	-4.03759	0.000623	-0.28005	AA955833	Rn.203532
A_43_P11752	-0.96434	-3.66447	0.0015	-1.01425	NM_013198	Rn.6656
A_44_P342908	-0.96525	-4.33915	0.000306	0.312097	NM_001012076	Rn.161745
A_43_P17784	-0.96692	-3.85118	0.000967	-0.64709	NM_001025722	Rn.20472
A_44_P487021	-0.96709	-3.22271	0.004192	-1.87435	NM_001002830	Rn.36357
A_44_P698487	-0.96725	-3.34306	0.003175	-1.64189	XR_007600	Rn.195696
A_44_P776000	-0.9679	-3.70176	0.001375	-0.94102	XM_001061754	Rn.215588
A_44_P196757	-0.96821	-3.45633	0.002441	-1.42164	XM_224256	Rn.206255
A_44_P764653	-0.9685	-3.99149	0.000695	-0.37081	CO559017	Rn.103732
A_44_P166396	-0.96998	-4.06898	0.000579	-0.21828	XM_216348	Rn.3404
A_44_P111368	-0.9704	-3.08231	0.005783	-2.14299	NM_001037356	Rn.115052
A_44_P191903	-0.97132	-3.94575	0.000774	-0.46087	NM_053693	Rn.116028
A_44_P625415	-0.97156	-4.22269	0.000403	0.083845	XM_220333	Rn.44008
A_44_P237886	-0.97168	-2.88155	0.009105	-2.52107	NM_031353	Rn.54594
A_44_P318891	-0.97356	-3.19171	0.004502	-1.93392	XR_007422	Rn.195786
A_44_P248541	-0.9747	-3.21344	0.004283	-1.89218	NM_001014263	Rn.17193
A_44_P111959	-0.97489	-6.34664	3.11E-06	4.010944	BC087709	Rn.10770

A_44_P187827	-0.9753	-3.11962	0.00531	-2.07189	XM_213437	Rn.162165
A_44_P156439	-0.97622	-3.55027	0.00196	-1.23813	XM_344861	Rn.198494
A_44_P193210	-0.97906	-3.08299	0.005774	-2.1417	AY724483	Rn.104592
A_44_P114023	-0.97983	-3.4074	0.002735	-1.51694	NM_001047972	Rn.44723
A_44_P501593	-0.98128	-3.74016	0.001256	-0.86554	XM_230773	Rn.3514
A_44_P555525	-0.98329	-2.93825	0.008015	-2.41511	NM_001007625	Rn.2881
A_44_P347007	-0.98573	-3.13331	0.005147	-2.04576	XM_001058095	Rn.1431
A_44_P290231	-0.99131	-3.12235	0.005277	-2.06667	NM_013013	Rn.97173
A_44_P201511	-0.99178	-4.63903	0.000151	0.895878	NM_001012087	Rn.117620
A_44_P386840	-0.99281	-3.34017	0.003197	-1.64748	NM_001013209	Rn.129050
A_44_P401170	-0.99407	-3.09524	0.005615	-2.11838	NM_001025014	Rn.992
A_44_P328311	-0.99578	-3.15084	0.004944	-2.01224	XR_007180	Rn.197941
A_44_P149161	-0.99613	-2.95476	0.007722	-2.38413	NM_198738	Rn.100813
A_44_P238710	-0.99781	-3.92811	0.000807	-0.4956	XM_218747	Rn.101871
A_43_P10867	-1.00068	-3.54991	0.001962	-1.23884	CB547678	
A_44_P239095	-1.00192	-4.10018	0.000538	-0.15689	XM_223440	Rn.203482
A_43_P15490	-1.00254	-4.80208	0.000103	1.2101	NM_134377	Rn.212755
A_44_P123898	-1.00282	-3.33367	0.003245	-1.66009	XM_341452	
A_44_P214054	-1.00415	-2.89273	0.008879	-2.50024	NM_031722	Rn.1022
A_44_P162099	-1.00419	-3.39594	0.002809	-1.53921	NM_053296	Rn.162569
A_43_P15623	-1.00434	-3.76129	0.001195	-0.82398	NM_053414	Rn.65536
A_44_P479520	-1.00546	-3.26176	0.003832	-1.79912	XM_341907	Rn.121213
A_44_P905187	-1.00547	-3.52755	0.002067	-1.28257	XM_233231	Rn.23812
A_44_P170309	-1.00656	-4.48435	0.000217	0.595593	XM_001064372	Rn.91234
A_44_P950583	-1.00883	-3.41496	0.002687	-1.50222	CO396335	Rn.165102
A_44_P500906	-1.01088	-3.1624	0.004815	-1.99011	NM_012788	Rn.89331
A_44_P912711	-1.01221	-3.21018	0.004315	-1.89844	XM_224429	Rn.35614
A_44_P929345	-1.01292	-3.73269	0.001278	-0.88022	XM_576703	Rn.215588
A_44_P426919	-1.01334	-3.12144	0.005288	-2.06842	XM_345601	
A_44_P328121	-1.01696	-3.06056	0.006076	-2.18432	NM_057207	Rn.58137
A_44_P978901	-1.01726	-3.49325	0.00224	-1.34959	XM_218747	Rn.101871
A_44_P296084	-1.02016	-4.16188	0.000465	-0.03558	CB544459	Rn.8671
A_44_P159296	-1.02072	-2.99417	0.007064	-2.30994	NM_001013929	Rn.21454
A_44_P100991	-1.02072	-3.67798	0.001454	-0.98772	XM_220404	Rn.106999
A_44_P519174	-1.02111	-4.32198	0.000319	0.278498	NM_001015027	Rn.107553
A_44_P320955	-1.02329	-3.288	0.003607	-1.74846	NM_001012213	Rn.115752
A_44_P231994	-1.0233	-3.92708	0.000809	-0.49764	XM_231626	Rn.68205
A_44_P183241	-1.02358	-3.10951	0.005435	-2.09118	XM_221333	Rn.20571
A_43_P16889	-1.02591	-3.41346	0.002697	-1.50515	XM_220636	Rn.3552
A_44_P131031	-1.02638	-3.52242	0.002092	-1.2926	XR_006475	Rn.196926
A_44_P428872	-1.02704	-2.95938	0.007642	-2.37545	XM_341137	Rn.8617
A_44_P769703	-1.02981	-3.21047	0.004312	-1.89788	NM_178096	Rn.8180
A_42_P843366	-1.03064	-3.78033	0.001143	-0.78652	NM_022681	Rn.48777
A_44_P394401	-1.03564	-4.87997	8.60E-05	1.359247	XM_230531	Rn.19932
A_44_P269675	-1.03937	-3.0476	0.006258	-2.2089	XM_001065667	Rn.195775
A_44_P251692	-1.04279	-2.9408	0.00797	-2.41034	NM_019257	Rn.54448
A_43_P15601	-1.04306	-4.3204	0.00032	0.275402	NM_134351	Rn.41420
A_44_P118993	-1.04578	-3.22708	0.00415	-1.86593	NM_134351	Rn.41420
A_44_P108728	-1.05234	-3.13893	0.005081	-2.03502	CA512164	Rn.201978
A_44_P356658	-1.05278	-4.266	0.000364	0.168793	XM_219716	Rn.28086
A_44_P433568	-1.05514	-2.92076	0.008337	-2.44787	XM_341882	Rn.7634
A_44_P420005	-1.06014	-3.64043	0.001587	-1.06144	NM_001047897	Rn.202345
A_44_P110392	-1.06231	-2.84945	0.009783	-2.58075		
A_44_P917564	-1.06249	-3.93918	0.000786	-0.4738	NM_001044282	Rn.1752
A_44_P790861	-1.06264	-3.69124	0.001409	-0.96167		
A_44_P189326	-1.06692	-4.17566	0.00045	-0.00851	NM_017319	Rn.11527
A_44_P215393	-1.06753	-3.05585	0.006142	-2.19326	NM_001025129	Rn.1569
A_44_P109887	-1.06914	-3.12635	0.005229	-2.05905	XM_214007	Rn.6670

A_42_P509365	-1.06929	-3.14179	0.005048	-2.02955	NM_031140	Rn.2710
A_44_P374848	-1.06963	-4.38819	0.000273	0.408003	NM_024139	Rn.11041
A_44_P354008	-1.07248	-3.72763	0.001294	-0.89016	XM_224429	Rn.35614
A_44_P482267	-1.07356	-3.12736	0.005217	-2.05712	NM_134382	Rn.212229
A_43_P16591	-1.07594	-3.60758	0.001714	-1.12587	AY724483	Rn.104592
A_44_P461470	-1.07792	-4.38835	0.000273	0.40831	NM_053714	Rn.211841
A_44_P699787	-1.078	-3.94204	0.000781	-0.46818	XM_221369	Rn.3336
A_44_P443138	-1.08064	-3.44016	0.002534	-1.45316	XM_001054273	Rn.201277
A_44_P116904	-1.08116	-3.96884	0.000733	-0.4154	XM_225314	Rn.146842
A_43_P11707	-1.08119	-3.87981	0.000904	-0.59071	NM_013090	Rn.31977
A_44_P484826	-1.08174	-3.3917	0.002837	-1.54745	NM_017276	Rn.1441
A_44_P856083	-1.08279	-3.76204	0.001193	-0.82251		
A_44_P732939	-1.08875	-2.92692	0.008222	-2.43634	XM_220269	Rn.18066
A_44_P828123	-1.08882	-3.52778	0.002066	-1.28212	CO393245	Rn.17209
A_44_P687063	-1.09454	-2.87937	0.009149	-2.52515		
A_43_P16511	-1.10021	-2.92872	0.008189	-2.43297	NM_001044300	Rn.19946
A_44_P651866	-1.10089	-3.85676	0.000955	-0.63611	XR_009004	Rn.159826
A_44_P846589	-1.10464	-3.44052	0.002532	-1.45244	NM_022229	Rn.102058
A_44_P109862	-1.10669	-5.37824	2.72E-05	2.29599	NM_001047085	Rn.36174
A_44_P141351	-1.11934	-3.44598	0.0025	-1.44182	AY035551	Rn.25058
A_44_P855981	-1.12135	-3.48886	0.002263	-1.35818		
A_44_P110158	-1.12302	-3.95796	0.000752	-0.43683	XM_232077	Rn.214082
A_44_P433542	-1.126	-2.99336	0.007077	-2.31148	NM_198132	Rn.107690
A_44_P112641	-1.12738	-4.94143	7.45E-05	1.476456	XM_221214	Rn.205861
A_44_P108982	-1.13111	-3.16454	0.004792	-1.98601	XM_341354	Rn.62159
A_44_P283366	-1.13155	-4.33169	0.000311	0.297508	NM_181081	Rn.12618
A_44_P379420	-1.13262	-4.81935	9.91E-05	1.243223	NM_017088	Rn.4000
A_44_P903850	-1.13435	-2.8515	0.009738	-2.57695	NM_001042621	Rn.1836
A_44_P419757	-1.13688	-3.94714	0.000772	-0.45813	XM_346210	
A_44_P187144	-1.14415	-3.81555	0.001052	-0.71722	NM_080896	Rn.23677
A_44_P205301	-1.14422	-4.00187	0.000678	-0.35037	NM_183402	Rn.144666
A_44_P281435	-1.14894	-4.38392	0.000275	0.399662	NM_001013923	Rn.93910
A_44_P206284	-1.15026	-3.97809	0.000717	-0.39719	XM_217496	Rn.207764
A_44_P458704	-1.15889	-4.46688	0.000227	0.561562	NM_017073	Rn.2204
A_44_P426808	-1.16124	-3.9761	0.000721	-0.4011	NM_001013033	Rn.99721
A_42_P828898	-1.16964	-4.03832	0.000622	-0.27861	XM_219958	Rn.105679
A_43_P21284	-1.17777	-3.01953	0.00667	-2.26205	NM_001012072	Rn.162528
A_44_P365598	-1.18163	-4.18667	0.000439	0.013107	NM_001012060	Rn.16849
A_44_P554271	-1.18236	-3.36088	0.003047	-1.60732	NM_001012235	Rn.190651
A_43_P22174	-1.18905	-4.96768	7.01E-05	1.526394	NM_001014135	Rn.3168
A_44_P549445	-1.19	-3.76594	0.001182	-0.81484	NM_130755	Rn.66581
A_44_P149114	-1.1902	-2.94652	0.007867	-2.3996	NM_057213	Rn.8109
A_43_P12430	-1.19174	-3.57875	0.001834	-1.18237	NM_024374	Rn.3239
A_43_P11881	-1.19527	-5.53102	1.92E-05	2.576423	NM_017253	Rn.8273
A_44_P112597	-1.19667	-3.6968	0.001391	-0.95075	XM_220423	Rn.204170
A_44_P196717	-1.21055	-2.9775	0.007336	-2.34136	XM_344970	Rn.154758
A_44_P496496	-1.21685	-3.71666	0.001327	-0.91172	XM_342857	Rn.10787
A_44_P852695	-1.23085	-3.37033	0.002981	-1.58898	XR_007221	Rn.196906
A_44_P594455	-1.23274	-3.47024	0.002363	-1.3945	XM_341907	Rn.121213
A_43_P10894	-1.23765	-3.93409	0.000796	-0.48382	NM_001025635	Rn.48209
A_44_P409336	-1.24237	-3.51661	0.002121	-1.30396	U56261	Rn.107689
A_44_P555389	-1.24392	-3.07815	0.005838	-2.1509	NM_138839	Rn.109048
A_43_P12816	-1.24615	-3.06355	0.006035	-2.17864	U45479	Rn.22685
A_44_P550364	-1.24756	-2.98026	0.00729	-2.33616	NM_080697	Rn.36362
A_44_P538779	-1.25001	-3.35783	0.003068	-1.61324	NM_001017385	Rn.1819
A_44_P625348	-1.26332	-3.14607	0.004999	-2.02136		
A_43_P11613	-1.27292	-2.97037	0.007455	-2.35478	NM_012903	Rn.10123
A_44_P992854	-1.28517	-3.39782	0.002797	-1.53556	NM_057132	Rn.107401

A_44_P480605	-1.28519	-3.35511	0.003088	-1.61851	XM_001066230	Rn.196993
A_44_P243145	-1.292	-3.69775	0.001388	-0.94888	NM_021748	Rn.13345
A_44_P1040736	-1.29428	-4.22966	0.000396	0.097515	NM_199384	Rn.108012
A_44_P196625	-1.30009	-3.27419	0.003723	-1.77513	XM_222253	Rn.105079
A_43_P18282	-1.30497	-4.22411	0.000401	0.086615	XM_341483	Rn.53930
A_44_P858481	-1.31791	-3.96044	0.000748	-0.43195		
A_44_P442161	-1.31929	-3.29046	0.003586	-1.74371	XM_342405	Rn.24600
A_44_P964633	-1.33495	-3.76832	0.001176	-0.81016	CO393123	Rn.203361
A_44_P104976	-1.34123	-4.53792	0.000192	0.699814	NM_080583	Rn.56138
A_44_P515029	-1.34738	-3.17276	0.004702	-1.97026	XM_215469	Rn.98152
A_44_P293750	-1.35762	-3.68183	0.001441	-0.98016	XM_001066493	Rn.115357
A_44_P133044	-1.36699	-2.93484	0.008077	-2.42151		
A_44_P338059	-1.37093	-4.63171	0.000154	0.881723	XM_343264	Rn.204679
A_44_P127156	-1.3759	-3.65076	0.00155	-1.04117	M55017	
A_44_P480533	-1.37601	-4.01424	0.000659	-0.32602		
A_44_P544023	-1.37978	-3.09798	0.00558	-2.11316	NM_001011995	Rn.154526
A_44_P902244	-1.3828	-3.42489	0.002626	-1.48289	XM_001066493	Rn.115357
A_43_P12839	-1.39288	-3.28852	0.003602	-1.74746	NM_053531	Rn.34151
A_44_P198396	-1.39777	-3.17063	0.004725	-1.97434	NM_031841	Rn.83595
A_44_P958966	-1.41457	-5.80308	1.04E-05	3.067006	XR_005722	Rn.160875
A_42_P618436	-1.42587	-4.07546	0.00057	-0.20552	XM_235689	Rn.17060
A_44_P213415	-1.44118	-3.6434	0.001576	-1.0556	NM_031353	Rn.54594
A_44_P529743	-1.45184	-3.4711	0.002358	-1.39283	XM_574587	Rn.11638
A_44_P107219	-1.46982	-3.35138	0.003115	-1.62576	NM_207591	Rn.8075
A_44_P238314	-1.4851	-2.86196	0.009513	-2.55753	NM_019621	Rn.9765
A_44_P918103	-1.50925	-3.41022	0.002717	-1.51144		
A_44_P177434	-1.51232	-3.85269	0.000964	-0.64412	NM_178105	Rn.34370
A_44_P488637	-1.5264	-3.15338	0.004916	-2.00737	NM_019351	Rn.22514
A_44_P180786	-1.56348	-3.74904	0.00123	-0.84808	XM_217372	Rn.1410
A_44_P217648	-1.66614	-4.23607	0.00039	0.110087	NM_001037217	Rn.154557
A_44_P488022	-1.66743	-5.76861	1.12E-05	3.005493	XM_576451	Rn.17305
A_44_P238312	-1.67663	-3.08466	0.005752	-2.13851	NM_019621	Rn.9765
A_44_P168842	-1.68399	-4.22397	0.000402	0.08634	XR_006947	Rn.197396
A_44_P477322	-1.69687	-4.03898	0.000621	-0.27731	NM_031831	Rn.1348
A_43_P12141	-1.79599	-4.38202	0.000277	0.395949	NM_021869	Rn.1993
A_44_P448181	-1.79904	-4.5501	0.000186	0.723483	AY569014	Rn.99876
A_43_P18735	-1.80003	-3.75371	0.001217	-0.83889	NM_001030030	Rn.100522
A_43_P15367	-1.83533	-3.63936	0.001591	-1.06354	NM_139189	Rn.201860
A_44_P149148	-1.92399	-4.28889	0.000345	0.21367	NM_017309	Rn.42903

Table S4

Median: 0.009991979436682584	Geo: 0.013098146982064234		
Term	Count	%	PValue
GO:0032502~developmental process	29	24.37%	0.0019038
GO:0048513~organ development	17	14.29%	0.00264471
GO:0048468~cell development	16	13.45%	0.00365645
GO:0030154~cell differentiation	19	15.97%	0.00467809
GO:0048869~cellular developmental process	19	15.97%	0.00467809
GO:0048731~system development	19	15.97%	0.00999198
GO:0007275~multicellular organismal development	21	17.65%	0.0146788
GO:0048856~anatomical structure development	20	16.81%	0.02188862
GO:0007399~nervous system development	10	8.40%	0.03763158
GO:0009653~anatomical structure morphogenesis	11	9.24%	0.09039635
GO:0032501~multicellular organismal process	27	22.69%	0.44243405

Median: 0.007011150321556307	Geo: 0.01656665519371505		
Term	Count	%	PValue
GO:0065007~biological regulation	36	30.25%	0.00358475
GO:0050789~regulation of biological process	32	26.89%	0.00624207
GO:0050794~regulation of cellular process	29	24.37%	0.00778023
GO:0019222~regulation of metabolic process	13	10.92%	0.43267287

Median: 0.05358450190602306	Geo: 0.03213539348414222		
Term	Count	%	PValue
GO:0048468~cell development	16	13.45%	0.00365645
GO:0030154~cell differentiation	19	15.97%	0.00467809
GO:0048869~cellular developmental process	19	15.97%	0.00467809
GO:0016265~death	11	9.24%	0.00950467
GO:0008219~cell death	11	9.24%	0.00950467
GO:0008283~cell proliferation	11	9.24%	0.01140801
GO:0006915~apoptosis	10	8.40%	0.01943025
GO:0012501~programmed cell death	10	8.40%	0.02096655
GO:0048518~positive regulation of biological proce	12	10.08%	0.04580325
GO:0048523~negative regulation of cellular proces	11	9.24%	0.05139072
GO:0048522~positive regulation of cellular process	11	9.24%	0.05577828
GO:0008284~positive regulation of cell proliferatior	5	4.20%	0.05685355
GO:0043065~positive regulation of apoptosis	5	4.20%	0.06626229
GO:0043068~positive regulation of programmed ce	5	4.20%	0.06854287
GO:0048519~negative regulation of biological proc	11	9.24%	0.07541499
GO:0042127~regulation of cell proliferation	7	5.88%	0.08055805
GO:0042981~regulation of apoptosis	7	5.88%	0.09226092
GO:0043067~regulation of programmed cell death	7	5.88%	0.09745399
GO:0043066~negative regulation of apoptosis	4	3.36%	0.147503
GO:0043069~negative regulation of programmed c	4	3.36%	0.15030364

Median: 0.029321235494187657	Geo: 0.038754254726694026		
Term	Count	%	PValue
GO:0051179~localization	30	25.21%	7.56E-04
GO:0006810~transport	25	21.01%	0.00354746
GO:0051234~establishment of localization	25	21.01%	0.00574139
GO:0022892~substrate-specific transporter activity	14	11.76%	0.00815607
GO:0022857~transmembrane transporter activity	13	10.92%	0.00963755
GO:0006812~cation transport	9	7.56%	0.00979958
GO:0030001~metal ion transport	8	6.72%	0.01000551
GO:0022891~substrate-specific transmembrane tra	12	10.08%	0.01211268
GO:0015674~di-, tri-valent inorganic cation transpo	5	4.20%	0.01456401
GO:0005215~transporter activity	15	12.61%	0.02445628
GO:0022803~passive transmembrane transporter a	7	5.88%	0.02559288
GO:0015267~channel activity	7	5.88%	0.02559288
GO:0015075~ion transmembrane transporter activi	10	8.40%	0.02773796
GO:0006811~ion transport	10	8.40%	0.02932124
GO:0006816~calcium ion transport	4	3.36%	0.03470812
GO:0046873~metal ion transmembrane transporter	6	5.04%	0.04073964
GO:0008324~cation transmembrane transporter ac	8	6.72%	0.04694332
GO:0022838~substrate specific channel activity	6	5.04%	0.06781793
GO:0022890~inorganic cation transmembrane trans	4	3.36%	0.09697663
GO:0005216~ion channel activity	5	4.20%	0.16194302
GO:0015077~monovalent inorganic cation transme	3	2.52%	0.19009553
GO:0005261~cation channel activity	4	3.36%	0.20972592
GO:0015672~monovalent inorganic cation transpor	4	3.36%	0.27459217
GO:0005244~voltage-gated ion channel activity	3	2.52%	0.27903633
GO:0022832~voltage-gated channel activity	3	2.52%	0.27903633
GO:0022836~gated channel activity	3	2.52%	0.50244347
transport	4	3.36%	0.92879381

Median: 0.05103870428246421	Geo: 0.04066100997140925		
Term	Count	%	PValue
gpi-anchor	4	3.36%	0.00498683
GO:0048503~GPI anchor binding	4	3.36%	0.00526782
GO:0035091~phosphoinositide binding	4	3.36%	0.03295023
GO:0008289~lipid binding	6	5.04%	0.0510387
lipoprotein	6	5.04%	0.0603392
GO:0005543~phospholipid binding	4	3.36%	0.08847203
glycoprotein	10	8.40%	0.77916217

Median: 0.06375143382925563	Geo: 0.055121287609943094		
Term	Count	%	PValue
GO:0000902~cell morphogenesis	9	7.56%	0.00654198
GO:0032989~cellular structure morphogenesis	9	7.56%	0.00654198
GO:0051674~localization of cell	8	6.72%	0.00843971

GO:0006928~cell motility	8	6.72%	0.00843971
GO:0045664~regulation of neuron differentiation	3	2.52%	0.02142502
GO:0030182~neuron differentiation	6	5.04%	0.03435246
GO:0007399~nervous system development	10	8.40%	0.03763158
GO:0009966~regulation of signal transduction	7	5.88%	0.0550051
GO:0048699~generation of neurons	6	5.04%	0.0551754
GO:0009888~tissue development	6	5.04%	0.06087848
GO:0030030~cell projection organization and bioge	5	4.20%	0.06255205
GO:0032990~cell part morphogenesis	5	4.20%	0.06255205
GO:0048858~cell projection morphogenesis	5	4.20%	0.06255205
GO:0009611~response to wounding	7	5.88%	0.06495082
GO:0022008~neurogenesis	6	5.04%	0.07722863
GO:0048667~neuron morphogenesis during differe	4	3.36%	0.08301481
GO:0048812~neurite morphogenesis	4	3.36%	0.08301481
GO:0050793~regulation of developmental process	5	4.20%	0.0890998
GO:0009653~anatomical structure morphogenesis	11	9.24%	0.09039635
GO:0016477~cell migration	5	4.20%	0.09174819
GO:0040007~growth	5	4.20%	0.09533805
GO:0000904~cellular morphogenesis during differe	4	3.36%	0.10288219
GO:0031175~neurite development	4	3.36%	0.11410047
GO:0048666~neuron development	4	3.36%	0.14890113
GO:0045595~regulation of cell differentiation	3	2.52%	0.29754846
GO:0007243~protein kinase cascade	4	3.36%	0.38887732

Median: 0.06568497554225985 Geo: 0.07576685350125058

Term	Count	%	PValue
GO:0006665~sphingolipid metabolic process	4	3.36%	0.00289589
GO:0046519~sphingoid metabolic process	3	2.52%	0.01366877
GO:0006643~membrane lipid metabolic process	4	3.36%	0.06305875
GO:0005783~endoplasmic reticulum	8	6.72%	0.06568498
GO:0044255~cellular lipid metabolic process	5	4.20%	0.38170379
GO:0008610~lipid biosynthetic process	3	2.52%	0.43645072
GO:0006629~lipid metabolic process	5	4.20%	0.52477172

Median: 0.12698373523485457 Geo: 0.1085747713659542

Term	Count	%	PValue
GO:0030659~cytoplasmic vesicle membrane	4	3.36%	0.00995806
GO:0044433~cytoplasmic vesicle part	4	3.36%	0.01786045
GO:0012506~vesicle membrane	4	3.36%	0.02250049
GO:0006886~intracellular protein transport	7	5.88%	0.03008529
GO:0030662~coated vesicle membrane	3	2.52%	0.03867681
GO:0046907~intracellular transport	9	7.56%	0.04142686
GO:0031982~vesicle	6	5.04%	0.05214339
calcium	6	5.04%	0.0881864
GO:0016023~cytoplasmic membrane-bound vesicle	5	4.20%	0.10152223

GO:0031988~membrane-bound vesicle	5	4.20%	0.10963595
GO:0015031~protein transport	7	5.88%	0.11611726
GO:0031410~cytoplasmic vesicle	5	4.20%	0.12233789
GO:0051649~establishment of cellular localization	9	7.56%	0.12639205
GO:0031090~organelle membrane	8	6.72%	0.12757542
GO:0051641~cellular localization	9	7.56%	0.13266272
GO:0045184~establishment of protein localization	7	5.88%	0.1517592
GO:0022607~cellular component assembly	6	5.04%	0.16807623
GO:0008104~protein localization	7	5.88%	0.18813107
GO:0006461~protein complex assembly	4	3.36%	0.20488479
GO:0033036~macromolecule localization	7	5.88%	0.21509493
GO:0030135~coated vesicle	3	2.52%	0.24447744
GO:0016192~vesicle-mediated transport	5	4.20%	0.27460159
GO:0065003~macromolecular complex assembly	5	4.20%	0.27726166
alternative splicing	7	5.88%	0.28705867
GO:0012505~endomembrane system	4	3.36%	0.49566323
splice variant	6	5.04%	0.50529718

Median: 0.09566593468640966 Geo: 0.12641338012275546

Term	Count	%	PValue
GO:0048514~blood vessel morphogenesis	4	3.36%	0.06705093
GO:0009653~anatomical structure morphogenesis	11	9.24%	0.09039635
GO:0001568~blood vessel development	4	3.36%	0.09097701
GO:0001944~vasculature development	4	3.36%	0.09566593
GO:0001525~angiogenesis	3	2.52%	0.17276244
GO:0009887~organ morphogenesis	5	4.20%	0.23150988
GO:0048646~anatomical structure formation	3	2.52%	0.244505

Median: 0.4181424454299303 Geo: 0.12708631750829097

Term	Count	%	PValue
GO:0051258~protein polymerization	5	4.20%	3.37E-04
GO:0046785~microtubule polymerization	3	2.52%	0.00122634
propeptide:Removed in mature form	5	4.20%	0.00483429
GO:0007010~cytoskeleton organization and biogen	9	7.56%	0.00554424
GO:0051128~regulation of cellular component orga	4	3.36%	0.00739916
GO:0031109~microtubule polymerization or depoly	3	2.52%	0.00960909
GO:0030036~actin cytoskeleton organization and b	5	4.20%	0.0231797
Methylation	4	3.36%	0.02721095
GO:0030029~actin filament-based process	5	4.20%	0.02752056
GO:0006996~organelle organization and biogenesis	11	9.24%	0.04869729
GO:0000226~microtubule cytoskeleton organization	3	2.52%	0.07868213
GO:0050793~regulation of developmental process	5	4.20%	0.0890998
GO:0007017~microtubule-based process	4	3.36%	0.11923121
rno04810:Regulation of actin cytoskeleton	4	3.36%	0.30419871

cytoplasm	9	7.56%	0.40296436
GO:0017111~nucleoside-triphosphatase activity	5	4.20%	0.41814245
GO:0016462~pyrophosphatase activity	5	4.20%	0.44739985
GO:0016818~hydrolase activity, acting on acid anh	5	4.20%	0.45292712
GO:0016817~hydrolase activity, acting on acid anh	5	4.20%	0.4598126
GO:0042623~ATPase activity, coupled	3	2.52%	0.51221187
nucleotide-binding	6	5.04%	0.55462111
GO:0016787~hydrolase activity	13	10.92%	0.58971636
GO:0016887~ATPase activity	3	2.52%	0.6078906
atp-binding	4	3.36%	0.72056333
GO:0032553~ribonucleotide binding	6	5.04%	0.96851476
GO:0032555~purine ribonucleotide binding	6	5.04%	0.96851476
GO:0017076~purine nucleotide binding	6	5.04%	0.97692857
GO:0000166~nucleotide binding	7	5.88%	0.97699087
GO:0005524~ATP binding	4	3.36%	0.9859679
GO:0032559~adenyl ribonucleotide binding	4	3.36%	0.98743697
GO:0030554~adenyl nucleotide binding	4	3.36%	0.99120971

Median: 0.22688672366390947 Geo: 0.17317491533194845

Term	Count	%	PValue
GO:0040007~growth	5	4.20%	0.09533805
GO:0016049~cell growth	3	2.52%	0.22688672
GO:0008361~regulation of cell size	3	2.52%	0.24009306

Median: 0.2165902008057497 Geo: 0.19738681531543417

Term	Count	%	PValue
GO:0009611~response to wounding	7	5.88%	0.06495082
GO:0006950~response to stress	10	8.40%	0.16020793
GO:0006954~inflammatory response	4	3.36%	0.27297247
GO:0006952~defense response	4	3.36%	0.53442293

Median: 0.21605817549213288 Geo: 0.19865088907109454

Term	Count	%	PValue
GO:0009888~tissue development	6	5.04%	0.06087848
GO:0001503~ossification	3	2.52%	0.1920233
GO:0031214~biomineral formation	3	2.52%	0.1920233
GO:0046849~bone remodeling	3	2.52%	0.24009306
GO:0048771~tissue remodeling	3	2.52%	0.26881863
GO:0001501~skeletal development	3	2.52%	0.42416269

Median: 0.27889043603661545 Geo: 0.2011624834289807

Term	Count	%	PValue
GO:0044444~cytoplasmic part	27	22.69%	0.03720137
GO:0005737~cytoplasm	33	27.73%	0.05258976
GO:0005622~intracellular	51	42.86%	0.13093863

GO:0043229~intracellular organelle	41	34.45%	0.1348883
GO:0043226~organelle	41	34.45%	0.13808623
GO:0044424~intracellular part	45	37.82%	0.27889044
GO:0043231~intracellular membrane-bound organe	33	27.73%	0.30426919
GO:0043227~membrane-bound organelle	33	27.73%	0.30690129
GO:0005623~cell	75	63.03%	0.37068418
GO:0044464~cell part	74	62.18%	0.54899273
GO:0009987~cellular process	64	53.78%	0.863184

Median: 0.15196001235368436	Geo: 0.20457578861941178		
Term	Count	%	PValue
GO:0005783~endoplasmic reticulum	8	6.72%	0.06568498
endoplasmic reticulum	5	4.20%	0.15196001
transmembrane region	10	8.40%	0.85776306

Median: 0.13366383848907484	Geo: 0.22905797793122154		
Term	Count	%	PValue
GO:0006935~chemotaxis	3	2.52%	0.13340296
GO:0042330~taxis	3	2.52%	0.13340296
GO:0007610~behavior	5	4.20%	0.13366384
GO:0007626~locomotory behavior	3	2.52%	0.26660663
GO:0042221~response to chemical stimulus	6	5.04%	0.99428592

Median: 0.21635803992904926	Geo: 0.2376488035116084		
Term	Count	%	PValue
GO:0005096~GTPase activator activity	3	2.52%	0.18339936
GO:0030234~enzyme regulator activity	7	5.88%	0.20844452
GO:0030695~GTPase regulator activity	4	3.36%	0.22427156
GO:0008047~enzyme activator activity	3	2.52%	0.37203176

Median: 0.22736533800174696	Geo: 0.2824205975609152		
Term	Count	%	PValue
GO:0048545~response to steroid hormone stimulus	3	2.52%	0.12540959
GO:0009719~response to endogenous stimulus	5	4.20%	0.20139135
GO:0009725~response to hormone stimulus	3	2.52%	0.25333932
GO:0042221~response to chemical stimulus	6	5.04%	0.99428592

Median: 0.2986929605585694	Geo: 0.2956774482192189		
Term	Count	%	PValue
GO:0050790~regulation of catalytic activity	5	4.20%	0.23023173
GO:0065009~regulation of a molecular function	5	4.20%	0.29869296
GO:0051338~regulation of transferase activity	3	2.52%	0.37589317

Median: 0.48319041257918804	Geo: 0.2994837815815022		
Term	Count	%	PValue

GO:0007050~cell cycle arrest	3	2.52%	0.03831729
GO:0045786~negative regulation of progression thr	3	2.52%	0.13139359
GO:0000074~regulation of progression through cell	3	2.52%	0.48028099
GO:0051726~regulation of cell cycle	3	2.52%	0.48609983
GO:0022402~cell cycle process	3	2.52%	0.74850836
GO:0007049~cell cycle	3	2.52%	0.82007454

Median: 0.36012413372890034 Geo: 0.34114833908466563

Term	Count	%	PValue
GO:0005615~extracellular space	16	13.45%	0.10473089
GO:0044421~extracellular region part	16	13.45%	0.14825301
GO:0005576~extracellular region	16	13.45%	0.28147749
signal	11	9.24%	0.36012413
disulfide bond	10	8.40%	0.49379321
signal peptide	9	7.56%	0.82725323
Secreted	4	3.36%	0.83645974

Median: 0.41578343518711314 Geo: 0.3415495270682285

Term	Count	%	PValue
GO:0031090~organelle membrane	8	6.72%	0.12757542
GO:0031966~mitochondrial membrane	4	3.36%	0.21886529
GO:0005740~mitochondrial envelope	4	3.36%	0.26119565
GO:0044429~mitochondrial part	4	3.36%	0.38474803
GO:0005743~mitochondrial inner membrane	3	2.52%	0.41578344
GO:0019866~organelle inner membrane	3	2.52%	0.44419225
GO:0031975~envelope	4	3.36%	0.47119996
GO:0031967~organelle envelope	4	3.36%	0.47119996
GO:0005739~mitochondrion	6	5.04%	0.54972855

Median: 0.6692685796256796 Geo: 0.34206884401432364

Term	Count	%	PValue
GO:0005886~plasma membrane	21	17.65%	0.00824633
membrane	23	19.33%	0.04504188
GO:0044459~plasma membrane part	15	12.61%	0.07477456
GO:0005887~integral to plasma membrane	8	6.72%	0.35720236
GO:0016020~membrane	42	35.29%	0.37195217
GO:0031226~intrinsic to plasma membrane	8	6.72%	0.3720425
transmembrane	13	10.92%	0.66010746
GO:0044425~membrane part	32	26.89%	0.6784297
transmembrane protein	3	2.52%	0.77150498
topological domain:Cytoplasmic	8	6.72%	0.85461623
transmembrane region	10	8.40%	0.85776306
GO:0016021~integral to membrane	24	20.17%	0.94918057
GO:0031224~intrinsic to membrane	24	20.17%	0.95202413
topological domain:Extracellular	5	4.20%	0.95580871

Median: 0.6697869754096847	Geo: 0.3551016719532347		
Term	Count	%	PValue
GO:0007242~intracellular signaling cascade	14	11.76%	0.0380455
GO:0007154~cell communication	27	22.69%	0.54613563
GO:0007165~signal transduction	22	18.49%	0.79343832
GO:0007166~cell surface receptor linked signal trai	11	9.24%	0.9644815

Median: 0.4580394280214789	Geo: 0.3817041963201787		
Term	Count	%	PValue
GO:0003714~transcription corepressor activity	3	2.52%	0.10708232
GO:0016564~transcription repressor activity	3	2.52%	0.33159958
GO:0003712~transcription cofactor activity	3	2.52%	0.45803943
GO:0008134~transcription factor binding	3	2.52%	0.65248975
GO:0030528~transcription regulator activity	7	5.88%	0.76353048

Median: 0.9948670647412782	Geo: 0.38869644747229287		
Term	Count	%	PValue
GO:0007267~cell-cell signaling	10	8.40%	0.02010251
GO:0007268~synaptic transmission	6	5.04%	0.08269739
GO:0019226~transmission of nerve impulse	6	5.04%	0.1264984
GO:0003008~system process	9	7.56%	0.96937986
GO:0050877~neurological system process	6	5.04%	0.99486706
GO:0007186~G-protein coupled receptor protein si	5	4.20%	0.99888011
GO:0001584~rhodopsin-like receptor activity	3	2.52%	0.99985866
GO:0004930~G-protein coupled receptor activity	3	2.52%	0.99994731
GO:0004888~transmembrane receptor activity	4	3.36%	0.99995356

Median: 0.7490033588699851	Geo: 0.4160549356873782		
Term	Count	%	PValue
GO:0031090~organelle membrane	8	6.72%	0.12757542
GO:0044446~intracellular organelle part	13	10.92%	0.74900336
GO:0044422~organelle part	13	10.92%	0.75370476

Median: 0.4748806082394614	Geo: 0.45696154314135434		
Term	Count	%	PValue
GO:0005509~calcium ion binding	7	5.88%	0.30641968
GO:0043167~ion binding	20	16.81%	0.35301025
GO:0046872~metal ion binding	19	15.97%	0.41200894
GO:0043169~cation binding	17	14.29%	0.53775228
GO:0008270~zinc ion binding	9	7.56%	0.58962396
GO:0046914~transition metal ion binding	11	9.24%	0.64433232

Median: 0.6108548056123693	Geo: 0.46366787538666515		
Term	Count	%	PValue

GO:0009055~electron carrier activity	4	3.36%	0.16010272
rno00190:Oxidative phosphorylation	3	2.52%	0.29465413
GO:0005739~mitochondrion	6	5.04%	0.54972855
GO:0006091~generation of precursor metabolites a	4	3.36%	0.67198106
GO:0006118~electron transport	3	2.52%	0.72145424
GO:0016491~oxidoreductase activity	5	4.20%	0.79034494

Median: 0.5781882914454173 Geo: 0.4860358594698407

Term	Count	%	PValue
GO:0005856~cytoskeleton	8	6.72%	0.18425912
GO:0043232~intracellular non-membrane-bound or	10	8.40%	0.57818829
GO:0043228~non-membrane-bound organelle	10	8.40%	0.57818829
GO:0044430~cytoskeletal part	4	3.36%	0.64268789
GO:0005198~structural molecule activity	5	4.20%	0.68513139

Median: 0.9423352055185812 Geo: 0.5612926576195539

Term	Count	%	PValue
GO:0006119~oxidative phosphorylation	3	2.52%	0.09294528
GO:0006091~generation of precursor metabolites a	4	3.36%	0.67198106
GO:0016310~phosphorylation	3	2.52%	0.94233521
GO:0006793~phosphorus metabolic process	3	2.52%	0.97292307
GO:0006796~phosphate metabolic process	3	2.52%	0.97292307

Median: 0.9706858737440671 Geo: 0.6755664698446382

Term	Count	%	PValue
rno04012:ErbB signaling pathway	3	2.52%	0.19756883
kinase	3	2.52%	0.74437278
GO:0016301~kinase activity	3	2.52%	0.97068587
GO:0016772~transferase activity, transferring phos	3	2.52%	0.98728428
GO:0016740~transferase activity	4	3.36%	0.99841383

Median: 0.7330494427919996 Geo: 0.689925413309536

Term	Count	%	PValue
Ionic channel	3	2.52%	0.48234063
ion transport	3	2.52%	0.73304944
transport	4	3.36%	0.92879381

Median: 0.7762607275814594 Geo: 0.7381337367173091

Term	Count	%	PValue
GO:0019538~protein metabolic process	20	16.81%	0.51096511
GO:0043170~macromolecule metabolic process	32	26.89%	0.65084492
GO:0044260~cellular macromolecule metabolic pro	17	14.29%	0.72895769
GO:0044238~primary metabolic process	36	30.25%	0.76037186
GO:0044267~cellular protein metabolic process	16	13.45%	0.79214959
GO:0044237~cellular metabolic process	35	29.41%	0.81029799

GO:0008152~metabolic process	39	32.77%	0.85052368
GO:0043283~biopolymer metabolic process	19	15.97%	0.87567903

Median: 0.796723032466517 Geo: 0.7558166759021745

Term	Count	%	PValue
GO:0019222~regulation of metabolic process	13	10.92%	0.43267287
GO:0010468~regulation of gene expression	11	9.24%	0.53827834
GO:0031323~regulation of cellular metabolic proce:	11	9.24%	0.62702663
GO:0045449~regulation of transcription	9	7.56%	0.71296839
GO:0006355~regulation of transcription, DNA-depe	8	6.72%	0.74680584
GO:0019219~regulation of nucleobase, nucleoside,	9	7.56%	0.74927528
GO:0030528~transcription regulator activity	7	5.88%	0.76353048
GO:0006350~transcription	9	7.56%	0.77827361
GO:0006351~transcription, DNA-dependent	8	6.72%	0.79672303
GO:0032774~RNA biosynthetic process	8	6.72%	0.79846712
GO:0010467~gene expression	13	10.92%	0.81665599
GO:0006139~nucleobase, nucleoside, nucleotide ar	14	11.76%	0.82572682
GO:0016070~RNA metabolic process	9	7.56%	0.8732745
GO:0043283~biopolymer metabolic process	19	15.97%	0.87567903
GO:0003700~transcription factor activity	4	3.36%	0.89256127
GO:0003677~DNA binding	7	5.88%	0.91650031
GO:0003676~nucleic acid binding	12	10.08%	0.92266281

Median: 0.7346365499807694 Geo: 0.7567949594906387

Term	Count	%	PValue
GO:0006412~translation	4	3.36%	0.6856941
GO:0009058~biosynthetic process	8	6.72%	0.73223591
GO:0009059~macromolecule biosynthetic process	5	4.20%	0.73703719
GO:0044249~cellular biosynthetic process	5	4.20%	0.88642519

Median: 0.8973573679866338 Geo: 0.8617550111058944

Term	Count	%	PValue
zinc-finger	3	2.52%	0.77262736
metal-binding	5	4.20%	0.89735737
zinc	3	2.52%	0.92303026

Median: 0.9736314715800498 Geo: 0.9595490109139895

Term	Count	%	PValue
GO:0043687~post-translational protein modificatior	5	4.20%	0.92717983
GO:0006464~protein modification process	5	4.20%	0.97363147
GO:0043412~biopolymer modification	5	4.20%	0.97868494

Median: 0.9468890540524483 Geo: 0.9630591152749226

Term	Count	%	PValue
GO:0004871~signal transducer activity	14	11.76%	0.94688905

GO:0060089~molecular transducer activity	14	11.76%	0.94688905
GO:0004872~receptor activity	9	7.56%	0.99623236

Table S5

Median: 6.748340700062148E-4	Geo: 6.426805056597486E-4		
Term	Count	%	PValue
GO:0032502~developmental process	45	29.61%	4.78E-06
GO:0048869~cellular developmental process	30	19.74%	2.99E-05
GO:0030154~cell differentiation	30	19.74%	2.99E-05
GO:0007275~multicellular organismal development	32	21.05%	5.16E-04
GO:0048731~system development	28	18.42%	6.75E-04
GO:0048856~anatomical structure development	30	19.74%	0.00152982
GO:0048513~organ development	22	14.47%	0.00159909
GO:0009653~anatomical structure morphogenesis	15	9.87%	0.05367044
GO:0032501~multicellular organismal process	43	28.29%	0.09576885

Median: 0.0036852104896518256	Geo: 0.00177884271287579		
Term	Count	%	PValue
GO:0042127~regulation of cell proliferation	15	9.87%	8.15E-05
GO:0008283~cell proliferation	18	11.84%	1.21E-04
GO:0008285~negative regulation of cell proliferatior	8	5.26%	0.00304683
GO:0008284~positive regulation of cell proliferation	8	5.26%	0.00432359
GO:0048523~negative regulation of cellular process	16	10.53%	0.01161529
GO:0048519~negative regulation of biological proce	16	10.53%	0.02091638

Median: 0.011553468381934585	Geo: 0.003868567826100308		
Term	Count	%	PValue
GO:0032502~developmental process	45	29.61%	4.78E-06
GO:0048869~cellular developmental process	30	19.74%	2.99E-05
GO:0030154~cell differentiation	30	19.74%	2.99E-05
GO:0048468~cell development	23	15.13%	2.67E-04
GO:0008219~cell death	14	9.21%	0.0066416
GO:0016265~death	14	9.21%	0.0066416
GO:0006915~apoptosis	13	8.55%	0.01155347
GO:0012501~programmed cell death	13	8.55%	0.01272079
GO:0042981~regulation of apoptosis	10	6.58%	0.03207116
GO:0043067~regulation of programmed cell death	10	6.58%	0.03482955
GO:0043066~negative regulation of apoptosis	5	3.29%	0.11766584
GO:0043069~negative regulation of programmed ce	5	3.29%	0.12049239
GO:0006916~anti-apoptosis	3	1.97%	0.37109276

Median: 0.011139084952939873	Geo: 0.004790451489712513		
Term	Count	%	PValue
GO:0048518~positive regulation of biological proces	26	17.11%	1.34E-06
GO:0032502~developmental process	45	29.61%	4.78E-06
GO:0048522~positive regulation of cellular process	23	15.13%	1.10E-05
GO:0065007~biological regulation	55	36.18%	1.57E-05
GO:0050794~regulation of cellular process	46	30.26%	2.45E-05
Transcription	15	9.87%	1.83E-04
GO:0050789~regulation of biological process	47	30.92%	2.50E-04
Transcription regulation	14	9.21%	5.62E-04
GO:0045941~positive regulation of transcription	10	6.58%	7.67E-04

GO:0045935~positive regulation of nucleobase, nucl	10	6.58%	0.00109242
GO:0031325~positive regulation of cellular metaboli	10	6.58%	0.00394122
dna-binding	13	8.55%	0.00417032
GO:0043283~biopolymer metabolic process	45	29.61%	0.00463287
GO:0006355~regulation of transcription, DNA-depen	22	14.47%	0.00601692
GO:0009893~positive regulation of metabolic proces	10	6.58%	0.00619934
GO:0003677~DNA binding	23	15.13%	0.00621432
nucleus	22	14.47%	0.00675776
GO:0016070~RNA metabolic process	26	17.11%	0.00861611
GO:0006351~transcription, DNA-dependent	22	14.47%	0.01101814
GO:0032774~RNA biosynthetic process	22	14.47%	0.01126003
GO:0003676~nucleic acid binding	32	21.05%	0.01151421
GO:0019219~regulation of nucleobase, nucleoside, r	23	15.13%	0.01208922
GO:0045449~regulation of transcription	22	14.47%	0.01618277
GO:0019222~regulation of metabolic process	25	16.45%	0.01911386
GO:0003700~transcription factor activity	14	9.21%	0.01981277
GO:0030528~transcription regulator activity	18	11.84%	0.02085853
GO:0005634~nucleus	35	23.03%	0.02523932
GO:0006139~nucleobase, nucleoside, nucleotide and	32	21.05%	0.02781943
GO:0006350~transcription	22	14.47%	0.03053968
GO:0010468~regulation of gene expression	22	14.47%	0.03448082
GO:0031323~regulation of cellular metabolic proces	23	15.13%	0.03705001
GO:0045893~positive regulation of transcription, DN	6	3.95%	0.05128254
GO:0045944~positive regulation of transcription fror	5	3.29%	0.06361487
GO:0010467~gene expression	27	17.76%	0.11393444
GO:0006357~regulation of transcription from RNA pc	7	4.61%	0.14555294
GO:0016563~transcription activator activity	5	3.29%	0.20323926
GO:0006366~transcription from RNA polymerase II p	7	4.61%	0.30991157
GO:0046983~protein dimerization activity	4	2.63%	0.5722885

Median: 0.00888381322916805 Geo: 0.005713472237800778

Term	Count	%	PValue
GO:0046872~metal ion binding	38	25.00%	9.66E-04
GO:0043167~ion binding	38	25.00%	0.00152226
GO:0046914~transition metal ion binding	26	17.11%	0.00400902
GO:0008270~zinc ion binding	20	13.16%	0.0137586
GO:0043169~cation binding	32	21.05%	0.01799826
metal-binding	18	11.84%	0.02383083

Median: 0.004267893538795904 Geo: 0.005825031646840147

Term	Count	%	PValue
zinc finger	6	3.95%	2.17E-05
DNA binding	7	4.61%	5.82E-04
zinc finger region:C2H2-type 3	4	2.63%	0.00360219
zinc finger region:C2H2-type 2	4	2.63%	0.00403583
zinc finger region:C2H2-type 1	4	2.63%	0.00449996
zinc-finger	9	5.92%	0.07610532
IPR007087:Zinc finger, C2H2-type	5	3.29%	0.10183541
zinc	10	6.58%	0.20726519

Median: 0.00782651025183407	Geo: 0.00761178627655859		
Term	Count	%	PValue
GO:0001569~patterning of blood vessels	4	2.63%	2.96E-04
GO:0007389~pattern specification process	7	4.61%	0.00204985
GO:0048646~anatomical structure formation	7	4.61%	0.00218479
GO:0009880~embryonic pattern specification	4	2.63%	0.00361935
GO:0001525~angiogenesis	6	3.95%	0.00412935
GO:0048754~branching morphogenesis of a tube	4	2.63%	0.00484675
GO:0001763~morphogenesis of a branching structu	4	2.63%	0.00669642
GO:0009887~organ morphogenesis	10	6.58%	0.00782651
GO:0048514~blood vessel morphogenesis	6	3.95%	0.01028697
GO:0001568~blood vessel development	6	3.95%	0.01730714
GO:0035295~tube development	6	3.95%	0.0176862
GO:0001944~vasculature development	6	3.95%	0.01885633
GO:0035239~tube morphogenesis	5	3.29%	0.0231404
GO:0009790~embryonic development	7	4.61%	0.044897
GO:0009653~anatomical structure morphogenesis	15	9.87%	0.05367044

Median: 0.05457297922436276	Geo: 0.01462053696013146		
Term	Count	%	PValue
zinc finger	6	3.95%	2.17E-05
IPR003070:Orphan nuclear receptor	3	1.97%	2.97E-04
PIRSF002524:probable hormone receptor N10, nucle	3	1.97%	3.95E-04
DNA binding	7	4.61%	5.82E-04
region of interest:Ligand-binding	3	1.97%	0.02103787
IPR001723:Steroid hormone receptor	3	1.97%	0.05028855
zinc finger region:NR C4-type	3	1.97%	0.05111912
DNA-binding region:Nuclear receptor	3	1.97%	0.05111912
IPR001628:Zinc finger, nuclear hormone receptor-typ	3	1.97%	0.05802684
IPR000536:Nuclear hormone receptor, ligand-binding	3	1.97%	0.05802684
IPR008946:Nuclear hormone receptor, ligand-binding	3	1.97%	0.05802684
GO:0003707~steroid hormone receptor activity	3	1.97%	0.06048219
SM00430:HOLI	3	1.97%	0.07872766
SM00399:ZnF_C4	3	1.97%	0.07872766
GO:0043565~sequence-specific DNA binding	7	4.61%	0.11289371
GO:0004879~ligand-dependent nuclear receptor act	3	1.97%	0.12910237

Median: 0.027819433619917497	Geo: 0.0306394706702835		
Term	Count	%	PValue
GO:0043283~biopolymer metabolic process	45	29.61%	0.00463287
GO:0009987~cellular process	101	66.45%	0.01801886
GO:0044237~cellular metabolic process	64	42.11%	0.02644205
GO:0006139~nucleobase, nucleoside, nucleotide an	32	21.05%	0.02781943
GO:0044238~primary metabolic process	62	40.79%	0.07000976
GO:0008152~metabolic process	68	44.74%	0.0740469
GO:0043170~macromolecule metabolic process	54	35.53%	0.07963106

Median: 0.043534823014015345	Geo: 0.042953132282386765

Term	Count	%	PValue
GO:0019932~second-messenger-mediated signaling	7	4.61%	0.0298774
GO:0007200~G-protein signaling, coupled to IP3 sec	4	2.63%	0.03872718
GO:0043085~positive regulation of catalytic activity	6	3.95%	0.04834246
GO:0048015~phosphoinositide-mediated signaling	4	2.63%	0.06085438

Median: 0.04555127796655499 Geo: 0.04839470743609883

Term	Count	%	PValue
GO:0030335~positive regulation of cell migration	3	1.97%	0.01085114
GO:0040017~positive regulation of locomotion	3	1.97%	0.01842662
GO:0051272~positive regulation of cell motility	3	1.97%	0.01842662
GO:0016477~cell migration	7	4.61%	0.03362988
GO:0006928~cell motility	8	5.26%	0.04555128
GO:0051674~localization of cell	8	5.26%	0.04555128
GO:0030334~regulation of cell migration	3	1.97%	0.10050712
GO:0051270~regulation of cell motility	3	1.97%	0.12495972
GO:0040012~regulation of locomotion	3	1.97%	0.14485459
GO:0040011~locomotion	3	1.97%	0.15065598

Median: 0.04022831394862833 Geo: 0.04844946335952177

Term	Count	%	PValue
GO:0005622~intracellular	77	50.66%	0.00474
GO:0043231~intracellular membrane-bound organel	54	35.53%	0.01200889
GO:0043227~membrane-bound organelle	54	35.53%	0.01229198
GO:0005634~nucleus	35	23.03%	0.02523932
GO:0044424~intracellular part	68	44.74%	0.03572386
GO:0043229~intracellular organelle	59	38.82%	0.04022831
GO:0043226~organelle	59	38.82%	0.0417487
GO:0005737~cytoplasm	42	27.63%	0.11320826
GO:0044464~cell part	105	69.08%	0.23012183
GO:0005623~cell	105	69.08%	0.23111091
GO:0044444~cytoplasmic part	27	17.76%	0.54126325

Median: 0.06545635625828403 Geo: 0.04962889637919206

Term	Count	%	PValue
GO:0050790~regulation of catalytic activity	10	6.58%	0.0077088
GO:0032147~activation of protein kinase activity	4	2.63%	0.0132609
GO:0065009~regulation of a molecular function	10	6.58%	0.01587624
GO:0045859~regulation of protein kinase activity	6	3.95%	0.02954193
GO:0043549~regulation of kinase activity	6	3.95%	0.03284997
GO:0051338~regulation of transferase activity	6	3.95%	0.0369918
GO:0007243~protein kinase cascade	8	5.26%	0.0386475
GO:0043085~positive regulation of catalytic activity	6	3.95%	0.04834246
GO:0006469~negative regulation of protein kinase a	3	1.97%	0.08257025
GO:0033673~negative regulation of kinase activity	3	1.97%	0.08257025
GO:0051348~negative regulation of transferase acti	3	1.97%	0.08758647
GO:0045860~positive regulation of protein kinase ac	4	2.63%	0.0941953
GO:0033674~positive regulation of kinase activity	4	2.63%	0.10092596
GO:0051347~positive regulation of transferase activ	4	2.63%	0.10960265

GO:0051336~regulation of hydrolase activity	4	2.63%	0.12777791
GO:0043086~negative regulation of catalytic activity	3	1.97%	0.15650345

Median: 0.14206229159700978 Geo: 0.07125983280189296

Term	Count	%	PValue
GO:0007242~intracellular signaling cascade	22	14.47%	0.00196264
GO:0007154~cell communication	45	29.61%	0.08178314
GO:0007165~signal transduction	39	25.66%	0.20234145
GO:0007166~cell surface receptor linked signal tran	20	13.16%	0.79394355

Median: 0.10637149401814736 Geo: 0.0849474832216158

Term	Count	%	PValue
GO:0000902~cell morphogenesis	10	6.58%	0.01669842
GO:0032989~cellular structure morphogenesis	10	6.58%	0.01669842
GO:0016477~cell migration	7	4.61%	0.03362988
GO:0006928~cell motility	8	5.26%	0.04555128
GO:0051674~localization of cell	8	5.26%	0.04555128
GO:0030030~cell projection organization and bioger	6	3.95%	0.05990029
GO:0048858~cell projection morphogenesis	6	3.95%	0.05990029
GO:0032990~cell part morphogenesis	6	3.95%	0.05990029
GO:0022008~neurogenesis	7	4.61%	0.10172001
GO:0030182~neuron differentiation	6	3.95%	0.11102298
GO:0016043~cellular component organization and b	26	17.11%	0.11236985
GO:0007409~axonogenesis	4	2.63%	0.13916871
GO:0048699~generation of neurons	6	3.95%	0.1651285
GO:0048812~neurite morphogenesis	4	2.63%	0.1752294
GO:0048667~neuron morphogenesis during differen	4	2.63%	0.1752294
GO:0000904~cellular morphogenesis during differen	4	2.63%	0.21139848
GO:0031175~neurite development	4	2.63%	0.23117678
GO:0048666~neuron development	4	2.63%	0.28990286

Median: 0.09104503852992898 Geo: 0.0908244416758216

Term	Count	%	PValue
GO:0046649~lymphocyte activation	5	3.29%	0.03889336
GO:0042110~T cell activation	4	2.63%	0.05815535
GO:0045321~leukocyte activation	5	3.29%	0.08376054
GO:0002376~immune system process	11	7.24%	0.0842084
GO:0046651~lymphocyte proliferation	3	1.97%	0.09788168
GO:0032943~mononuclear cell proliferation	3	1.97%	0.09788168
GO:0001775~cell activation	5	3.29%	0.1162637
GO:0006955~immune response	7	4.61%	0.26056428

Median: 0.10263827467169256 Geo: 0.1099454504314445

Term	Count	%	PValue
protein phosphatase	4	2.63%	0.01933435
domain:Tyrosine-protein phosphatase	3	1.97%	0.03341908
active site:Phosphocysteine intermediate	3	1.97%	0.04191161
GO:0006470~protein amino acid dephosphorylation	4	2.63%	0.08289082
IPR000387:Protein-tyrosine phosphatase, Tyr-specifi	3	1.97%	0.08945855

GO:0042578~phosphoric ester hydrolase activity	6	3.95%	0.09679904
GO:0016311~dephosphorylation	4	2.63%	0.10263827
GO:0016788~hydrolase activity, acting on ester bon	9	5.92%	0.10830484
GO:0004721~phosphoprotein phosphatase activity	4	2.63%	0.11146389
GO:0004725~protein tyrosine phosphatase activity	3	1.97%	0.12631583
GO:0016791~phosphoric monoester hydrolase activi	4	2.63%	0.28627894
GO:0016787~hydrolase activity	18	11.84%	0.49881661
hydrolase	7	4.61%	0.78952711

Median: 0.07802447275373386 Geo: 0.11298829618483724

Term	Count	%	PValue
GO:0048168~regulation of neuronal synaptic plastic	3	1.97%	0.03105792
GO:0007268~synaptic transmission	8	5.26%	0.04555128
GO:0007267~cell-cell signaling	11	7.24%	0.06121429
GO:0048167~regulation of synaptic plasticity	3	1.97%	0.07522256
GO:0019226~transmission of nerve impulse	8	5.26%	0.08082638
GO:0050803~regulation of synapse structure and ac	3	1.97%	0.08758647
GO:0003008~system process	18	11.84%	0.71987716
GO:0050877~neurological system process	15	9.87%	0.80010184

Median: 0.07946974656261849 Geo: 0.12741174769041366

Term	Count	%	PValue
GO:0007049~cell cycle	9	5.92%	0.06925923
cell cycle	5	3.29%	0.07650756
GO:0000074~regulation of progression through cell	6	3.95%	0.07661957
GO:0051726~regulation of cell cycle	6	3.95%	0.07946975
GO:0022402~cell cycle process	8	5.26%	0.08008684
GO:0000278~mitotic cell cycle	4	2.63%	0.32204552
GO:0022403~cell cycle phase	3	1.97%	0.65503475

Median: 0.12910236754218637 Geo: 0.1315662955643494

Term	Count	%	PValue
GO:0004620~phospholipase activity	3	1.97%	0.09416653
GO:0016298~lipase activity	3	1.97%	0.12910237
GO:0004091~carboxylesterase activity	3	1.97%	0.18732821

Median: 0.23450793810192835 Geo: 0.1421944033974187

Term	Count	%	PValue
GO:0042981~regulation of apoptosis	10	6.58%	0.03207116
GO:0043067~regulation of programmed cell death	10	6.58%	0.03482955
GO:0006917~induction of apoptosis	4	2.63%	0.23339623
GO:0012502~induction of programmed cell death	4	2.63%	0.23561965
GO:0043065~positive regulation of apoptosis	4	2.63%	0.36341037
GO:0043068~positive regulation of programmed cel	4	2.63%	0.37027961

Median: 0.21298981099252623 Geo: 0.14655099926417334

Term	Count	%	PValue
GO:0043687~post-translational protein modification	17	11.18%	0.03447465
GO:0043412~biopolymer modification	19	12.50%	0.05642001

GO:0006464~protein modification process	18	11.84%	0.07583392
GO:0043170~macromolecule metabolic process	54	35.53%	0.07963106
GO:0006793~phosphorus metabolic process	11	7.24%	0.21298981
GO:0006796~phosphate metabolic process	11	7.24%	0.21298981
GO:0044260~cellular macromolecule metabolic proc	28	18.42%	0.36408905
GO:0019538~protein metabolic process	29	19.08%	0.38759877
GO:0044267~cellular protein metabolic process	27	17.76%	0.41467928

Median: 0.14499032954436994 Geo: 0.15202648386524137

Term	Count	%	PValue
GO:0040007~growth	6	3.95%	0.09774778
GO:0016049~cell growth	4	2.63%	0.13343014
GO:0008361~regulation of cell size	4	2.63%	0.14499033
GO:0040008~regulation of growth	4	2.63%	0.16699377
GO:0001558~regulation of cell growth	3	1.97%	0.25715566

Median: 0.28092879778048224 Geo: 0.15998774749004374

Term	Count	%	PValue
GO:0035295~tube development	6	3.95%	0.0176862
GO:0045934~negative regulation of nucleobase, nuc	5	3.29%	0.17765547
GO:0031324~negative regulation of cellular metabo	5	3.29%	0.2809288
GO:0016481~negative regulation of transcription	4	2.63%	0.33814868
GO:0009892~negative regulation of metabolic proce	5	3.29%	0.35116929

Median: 0.21679396770072448 Geo: 0.17254682489492937

Term	Count	%	PValue
GO:0007631~feeding behavior	4	2.63%	0.0132609
GO:0019932~second-messenger-mediated signaling	7	4.61%	0.0298774
GO:0006873~cellular ion homeostasis	5	3.29%	0.14107926
GO:0055082~cellular chemical homeostasis	5	3.29%	0.14107926
GO:0007204~elevation of cytosolic calcium ion conc	3	1.97%	0.14485459
GO:0051480~cytosolic calcium ion homeostasis	3	1.97%	0.15357415
GO:0050801~ion homeostasis	5	3.29%	0.17931515
GO:0030005~cellular di-, tri-valent inorganic cation l	4	2.63%	0.18779203
GO:0055066~di-, tri-valent inorganic cation homeost	4	2.63%	0.18779203
GO:0048878~chemical homeostasis	5	3.29%	0.24579591
GO:0030003~cellular cation homeostasis	4	2.63%	0.24679262
GO:0055080~cation homeostasis	4	2.63%	0.24679262
GO:0019725~cellular homeostasis	5	3.29%	0.2753221
GO:0042592~homeostatic process	6	3.95%	0.3060075
GO:0006874~cellular calcium ion homeostasis	3	1.97%	0.31923838
GO:0055074~calcium ion homeostasis	3	1.97%	0.31923838
GO:0006875~cellular metal ion homeostasis	3	1.97%	0.3590126
GO:0055065~metal ion homeostasis	3	1.97%	0.3590126

Median: 0.21687007947550468 Geo: 0.18081047887105914

Term	Count	%	PValue
GO:0002376~immune system process	11	7.24%	0.0842084
GO:0030097~hemopoiesis	4	2.63%	0.19629554

GO:0002521~leukocyte differentiation	3	1.97%	0.21687008
GO:0048534~hemopoietic or lymphoid organ develo	4	2.63%	0.22454334
GO:0002520~immune system development	4	2.63%	0.24007799

Median: 0.1515089135837126 Geo: 0.18575887357629942

Term	Count	%	PValue
GO:0006665~sphingolipid metabolic process	3	1.97%	0.0612205
GO:0006643~membrane lipid metabolic process	4	2.63%	0.13724646
GO:0044255~cellular lipid metabolic process	8	5.26%	0.15150891
GO:0006629~lipid metabolic process	8	5.26%	0.28070626
GO:0008610~lipid biosynthetic process	3	1.97%	0.61895567

Median: 0.31306330276550215 Geo: 0.20213993292493965

Term	Count	%	PValue
GO:0009888~tissue development	8	5.26%	0.03004377
GO:0001501~skeletal development	5	3.29%	0.14566491
GO:0001503~ossification	3	1.97%	0.3130633
GO:0031214~biomineral formation	3	1.97%	0.3130633
GO:0046849~bone remodeling	3	1.97%	0.38009492
GO:0048771~tissue remodeling	3	1.97%	0.41845332

Median: 0.22612678300387035 Geo: 0.20522610538248465

Term	Count	%	PValue
GO:0007626~locomotory behavior	4	2.63%	0.16904168
GO:0006935~chemotaxis	3	1.97%	0.22612678
GO:0042330~taxis	3	1.97%	0.22612678

Median: 0.18718910532733748 Geo: 0.2140013630480883

Term	Count	%	PValue
disulfide bond	18	11.84%	0.09644361
GO:0005576~extracellular region	24	15.79%	0.10978214
GO:0044421~extracellular region part	21	13.82%	0.1520038
GO:0005615~extracellular space	20	13.16%	0.15518646
signal	18	11.84%	0.21919175
glycoprotein	20	13.16%	0.36803111
Secreted	9	5.92%	0.43196977
signal peptide	16	10.53%	0.50542507

Median: 0.28744567816976296 Geo: 0.24435719127448727

Term	Count	%	PValue
IPR007087:Zinc finger, C2H2-type	5	3.29%	0.10183541
IPR013087:Zinc finger, C2H2-type/integrase, DNA-bi	3	1.97%	0.22072902
IPR015880:Zinc finger, C2H2-like	3	1.97%	0.35416233
SM00355:ZnF_C2H2	3	1.97%	0.44785751

Median: 0.35873012641214763 Geo: 0.2739158732596765

Term	Count	%	PValue
GO:0004857~enzyme inhibitor activity	5	3.29%	0.1570748
GO:0004866~endopeptidase inhibitor activity	3	1.97%	0.35873013

GO:0030414~protease inhibitor activity	3	1.97%	0.36473484

Median: 0.25732520791296887 Geo: 0.3070428593235986

Term	Count	%	PValue
GO:0006397~mRNA processing	4	2.63%	0.13533357
GO:0005681~spliceosome	3	1.97%	0.14319607
GO:0016071~mRNA metabolic process	4	2.63%	0.19416055
GO:0006396~RNA processing	5	3.29%	0.21707126
GO:0008380~RNA splicing	3	1.97%	0.29757916
GO:0044428~nuclear part	7	4.61%	0.61839088
GO:0030529~ribonucleoprotein complex	5	3.29%	0.6720952
GO:0003723~RNA binding	4	2.63%	0.78198005

Median: 0.3776840545803085 Geo: 0.35546088644285123

Term	Count	%	PValue
Postsynaptic cell membrane	3	1.97%	0.24967477
cell junction	4	2.63%	0.37768405
synapse	3	1.97%	0.47629076

Median: 0.4350696695030194 Geo: 0.3726779361542909

Term	Count	%	PValue
lipoprotein	6	3.95%	0.25929439
Palmitate	3	1.97%	0.43506967
lipid moiety-binding region:S-palmitoyl cysteine	3	1.97%	0.45882716

Median: 0.6550347493280828 Geo: 0.3930222011751995

Term	Count	%	PValue
GO:0051704~multi-organism process	5	3.29%	0.11907542
GO:0022414~reproductive process	3	1.97%	0.65503475
GO:0000003~reproduction	4	2.63%	0.77833184

Median: 0.5887866746955883 Geo: 0.40006057508686044

Term	Count	%	PValue
GO:0000287~magnesium ion binding	8	5.26%	0.00212904
GO:0016301~kinase activity	12	7.89%	0.09401846
magnesium	5	3.29%	0.11028258
GO:0016772~transferase activity, transferring phosp	12	7.89%	0.20462216
GO:0006793~phosphorus metabolic process	11	7.24%	0.21298981
GO:0006796~phosphate metabolic process	11	7.24%	0.21298981
kinase	7	4.61%	0.24441827
GO:0016773~phosphotransferase activity, alcohol gr	9	5.92%	0.27451616
SM00220:S_TKc	6	3.95%	0.28396519
GO:0016740~transferase activity	17	11.18%	0.2851711
IPR002290:Serine/threonine protein kinase	3	1.97%	0.40439199
GO:0004672~protein kinase activity	7	4.61%	0.44285187
binding site:ATP	4	2.63%	0.45273372
IPR000719:Protein kinase, core	4	2.63%	0.5317224
IPR008271:Serine/threonine protein kinase, active sit	3	1.97%	0.55142859
Serine/threonine-protein kinase	3	1.97%	0.57400518

GO:0016310~phosphorylation	7	4.61%	0.60356817
GO:0004674~protein serine/threonine kinase activity	5	3.29%	0.61344031
GO:0006468~protein amino acid phosphorylation	6	3.95%	0.62083328
active site:Proton acceptor	4	2.63%	0.66904821
domain:Protein kinase	3	1.97%	0.68221601
nucleotide phosphate-binding region:ATP	4	2.63%	0.68868595
nucleotide-binding	8	5.26%	0.69231599
atp-binding	6	3.95%	0.71410301
GO:0005524~ATP binding	9	5.92%	0.82230441
GO:0032559~adenyl ribonucleotide binding	9	5.92%	0.83617534
GO:0000166~nucleotide binding	13	8.55%	0.84942789
transferase	6	3.95%	0.87435008
GO:0030554~adenyl nucleotide binding	9	5.92%	0.87489505
GO:0032555~purine ribonucleotide binding	10	6.58%	0.90630926
GO:0032553~ribonucleotide binding	10	6.58%	0.90630926
GO:0017076~purine nucleotide binding	10	6.58%	0.93066493

Median: 0.38568296458186957 Geo: 0.4004411400930015

Term	Count	%	PValue
GO:0005764~lysosome	3	1.97%	0.38252226
GO:0000323~lytic vacuole	3	1.97%	0.38568296
GO:0005773~vacuole	3	1.97%	0.43524009

Median: 0.6351311617719959 Geo: 0.4636426277900228

Term	Count	%	PValue
transducer	10	6.58%	0.02538471
g-protein coupled receptor	8	5.26%	0.09168277
GO:0001653~peptide receptor activity	4	2.63%	0.09275957
GO:0042277~peptide binding	5	3.29%	0.09441662
rno04080:Neuroactive ligand-receptor interaction	5	3.29%	0.23679494
membrane	30	19.74%	0.2394147
IPR000276:Rhodopsin-like GPCR superfamily	7	4.61%	0.24170315
G protein-coupled receptor	3	1.97%	0.24587694
GO:0005886~plasma membrane	20	13.16%	0.25253032
glycoprotein	20	13.16%	0.36803111
receptor	12	7.89%	0.40674756
GO:0044459~plasma membrane part	14	9.21%	0.54022955
GO:0005887~integral to plasma membrane	9	5.92%	0.56466453
GO:0031226~intrinsic to plasma membrane	9	5.92%	0.58241564
glycosylation site:N-linked (GlcNAc...)	17	11.18%	0.61271877
transmembrane	21	13.82%	0.6131457
transmembrane protein	5	3.29%	0.65711662
topological domain:Extracellular	11	7.24%	0.6586014
topological domain:Cytoplasmic	13	8.55%	0.72610211
GO:0007186~G-protein coupled receptor protein sig	16	10.53%	0.74139492
GO:0007166~cell surface receptor linked signal tran	20	13.16%	0.79394355
transmembrane region	15	9.87%	0.8266659
GO:0004871~signal transducer activity	20	13.16%	0.92876504
GO:0060089~molecular transducer activity	20	13.16%	0.92876504

GO:0004930~G-protein coupled receptor activity	11	7.24%	0.96003914
GO:0001584~rhodopsin-like receptor activity	9	5.92%	0.9810981
GO:0004888~transmembrane receptor activity	12	7.89%	0.9839537
GO:0004872~receptor activity	15	9.87%	0.98458069
GO:0016020~membrane	41	26.97%	0.9982432
GO:0044425~membrane part	31	20.39%	0.99928109
GO:0016021~integral to membrane	26	17.11%	0.99967323
GO:0031224~intrinsic to membrane	26	17.11%	0.99970986

Median: 0.4708182436304623 Geo: 0.4660174692927841

Term	Count	%	PValue
GO:0030135~coated vesicle	3	1.97%	0.38252226
GO:0016023~cytoplasmic membrane-bound vesicle	4	2.63%	0.45233669
GO:0031988~membrane-bound vesicle	4	2.63%	0.47081824
GO:0031410~cytoplasmic vesicle	4	2.63%	0.49801369
GO:0031982~vesicle	4	2.63%	0.54174871

Median: 0.5977868973943758 Geo: 0.5188412456329615

Term	Count	%	PValue
GO:0006396~RNA processing	5	3.29%	0.21707126
GO:0043233~organelle lumen	8	5.26%	0.25090659
GO:0031974~membrane-enclosed lumen	8	5.26%	0.25090659
rna-binding	3	1.97%	0.45932768
GO:0031981~nuclear lumen	5	3.29%	0.54864712
GO:0005654~nucleoplasm	4	2.63%	0.57718292
GO:0044428~nuclear part	7	4.61%	0.61839088
GO:0030529~ribonucleoprotein complex	5	3.29%	0.6720952
GO:0044451~nucleoplasm part	3	1.97%	0.72030841
GO:0044446~intracellular organelle part	17	11.18%	0.83082855
GO:0044422~organelle part	17	11.18%	0.83519455
GO:0032991~macromolecular complex	15	9.87%	0.92160933

Median: 0.5316807174174902 Geo: 0.5256863931597372

Term	Count	%	PValue
GO:0006954~inflammatory response	4	2.63%	0.47305709
GO:0006950~response to stress	10	6.58%	0.50424836
GO:0006952~defense response	5	3.29%	0.55911307
GO:0009611~response to wounding	5	3.29%	0.57259724

Median: 0.6050691824575416 Geo: 0.5538417602513612

Term	Count	%	PValue
GO:0005739~mitochondrion	9	5.92%	0.38018834
GO:0044429~mitochondrial part	4	2.63%	0.60506918
Mitochondrion	4	2.63%	0.7385047

Median: 0.6203505593051134 Geo: 0.5574080784731396

Term	Count	%	PValue
GO:0005578~proteinaceous extracellular matrix	4	2.63%	0.34465906
GO:0031012~extracellular matrix	4	2.63%	0.36653473

GO:0022610~biological adhesion	4	2.63%	0.87416638
GO:0007155~cell adhesion	4	2.63%	0.87416638

Median: 0.6077307778369707 Geo: 0.6401225395288845

Term	Count	%	PValue
GO:0051179~localization	25	16.45%	0.45835904
GO:0046907~intracellular transport	7	4.61%	0.47664479
GO:0033036~macromolecule localization	7	4.61%	0.50456307
GO:0006605~protein targeting	3	1.97%	0.55097123
GO:0051649~establishment of cellular localization	8	5.26%	0.56384228
GO:0051641~cellular localization	8	5.26%	0.57663052
GO:0008104~protein localization	6	3.95%	0.63883103
GO:0006886~intracellular protein transport	4	2.63%	0.64815115
GO:0045184~establishment of protein localization	5	3.29%	0.7541813
GO:0015031~protein transport	4	2.63%	0.85437133
GO:0051234~establishment of localization	16	10.53%	0.92870858
GO:0006810~transport	14	9.21%	0.96737074

Median: 0.7180410061501701 Geo: 0.6532110505514424

Term	Count	%	PValue
GO:0005506~iron ion binding	4	2.63%	0.40215727
iron	3	1.97%	0.58274416
GO:0016491~oxidoreductase activity	6	3.95%	0.85333785
oxidoreductase	3	1.97%	0.9103727

Median: 0.7898650649158409 Geo: 0.8021733696316998

Term	Count	%	PValue
GO:0006508~proteolysis	6	3.95%	0.73437339
GO:0008233~peptidase activity	5	3.29%	0.78986506
GO:0004175~endopeptidase activity	3	1.97%	0.88988704

Median: 0.970335217397613 Geo: 0.9247462277976668

Term	Count	%	PValue
GO:0008092~cytoskeletal protein binding	3	1.97%	0.79393513
GO:0005856~cytoskeleton	4	2.63%	0.9623272
GO:0043228~non-membrane-bound organelle	8	5.26%	0.97834324
GO:0043232~intracellular non-membrane-bound org	8	5.26%	0.97834324

Median: 0.9839158915997125 Geo: 0.9253029306672969

Term	Count	%	PValue
GO:0015267~channel activity	3	1.97%	0.81163057
GO:0022803~passive transmembrane transporter ac	3	1.97%	0.81163057
GO:0022857~transmembrane transporter activity	4	2.63%	0.9832207
GO:0005215~transporter activity	6	3.95%	0.98461108
GO:0022892~substrate-specific transporter activity	4	2.63%	0.99122499
GO:0022891~substrate-specific transmembrane trar	3	1.97%	0.9928848

Median: 0.9998834069831795 Geo: 0.9976789972437883

Term	Count	%	PValue

GO:0007600~sensory perception	7	4.61%	0.99325095
GO:0007608~sensory perception of smell	3	1.97%	0.99988341
GO:0007606~sensory perception of chemical stimulu	3	1.97%	0.99991743

Table S6

Median: 7.044309624431602E-9	Geo: 1.1045055549475803E-8		
Term	Count	%	PValue
GO:0005622~intracellular	501	44.81%	1.21E-19
GO:0044424~intracellular part	453	40.52%	3.90E-14
GO:0005737~cytoplasm	291	26.03%	8.02E-10
GO:0043227~membrane-bound organelle	333	29.79%	1.05E-09
GO:0043231~intracellular membrane-bound o	332	29.70%	1.48E-09
GO:0043226~organelle	377	33.72%	7.04E-09
GO:0043229~intracellular organelle	376	33.63%	8.62E-09
GO:0044444~cytoplasmic part	215	19.23%	2.79E-06
GO:0005634~nucleus	184	16.46%	0.005189
GO:0005623~cell	652	58.32%	0.006045
GO:0044464~cell part	651	58.23%	0.009543

Median: 3.542056092261983E-5	Geo: 3.775890795601803E-5		
Term	Count	%	PValue
GO:0016192~vesicle-mediated transport	55	4.92%	1.25E-07
GO:0015031~protein transport	64	5.72%	1.64E-07
GO:0045184~establishment of protein localiz	67	5.99%	2.77E-07
GO:0008104~protein localization	69	6.17%	7.17E-07
GO:0033036~macromolecule localization	70	6.26%	1.71E-06
GO:0006886~intracellular protein transport	47	4.20%	3.20E-06
GO:0016043~cellular component organizatior	181	16.19%	6.81E-06
GO:0046907~intracellular transport	64	5.72%	3.54E-05
GO:0051649~establishment of cellular localiz	74	6.62%	2.04E-04
GO:0051641~cellular localization	74	6.62%	2.87E-04
GO:0006810~transport	169	15.12%	5.43E-04
GO:0051234~establishment of localization	174	15.56%	6.17E-04
GO:0051179~localization	194	17.35%	8.66E-04
GO:0032940~secretion by cell	26	2.33%	0.044288
GO:0005215~transporter activity	81	7.25%	0.112579

Median: 0.0011776838916593433	Geo: 6.354060560924282E-4		
Term	Count	%	PValue
GO:0031090~organelle membrane	61	5.46%	1.14E-04
GO:0044422~organelle part	153	13.69%	0.001178
GO:0044446~intracellular organelle part	151	13.51%	0.001906

Median: 0.00409552834425737	Geo: 0.0011073699870049756		
Term	Count	%	PValue
GO:0016791~phosphoric monoester hydrolas	31	2.77%	5.27E-06
GO:0042578~phosphoric ester hydrolase acti	35	3.13%	2.72E-05
GO:0016788~hydrolase activity, acting on est	56	5.01%	4.15E-05
GO:0016311~dephosphorylation	17	1.52%	0.004096
GO:0004721~phosphoprotein phosphatase ac	16	1.43%	0.009877
GO:0006470~protein amino acid dephosphory	14	1.25%	0.022158
GO:0004725~protein tyrosine phosphatase ac	6	0.54%	0.383037

Median: 0.003228915351500921	Geo: 0.001441955382347955		
Term	Count	%	PValue
GO:0044238~primary metabolic process	417	37.30%	1.56E-05
GO:0008152~metabolic process	458	40.97%	2.05E-05
GO:0044237~cellular metabolic process	411	36.76%	4.95E-05
GO:0019538~protein metabolic process	218	19.50%	6.51E-04
GO:0043170~macromolecule metabolic proce	348	31.13%	0.002058
GO:0009987~cellular process	658	58.86%	0.002091
GO:0044267~cellular protein metabolic proce	199	17.80%	0.004366
GO:0044260~cellular macromolecule metabo	202	18.07%	0.004398
GO:0043412~biopolymer modification	104	9.30%	0.010652
GO:0006464~protein modification process	101	9.03%	0.011152
GO:0043283~biopolymer metabolic process	239	21.38%	0.015262
GO:0043687~post-translational protein modif	81	7.25%	0.052405

Median: 0.001445832600658012	Geo: 0.001837479914945177		
Term	Count	%	PValue
GO:0004437~inositol or phosphatidylinositol p	9	0.81%	2.56E-04
GO:0042577~lipid phosphatase activity	5	0.45%	0.001446
GO:0004439~phosphoinositide 5-phosphatase	3	0.27%	0.016744

Median: 0.023947488057755185	Geo: 0.001988447689941352		
Term	Count	%	PValue
GO:0016192~vesicle-mediated transport	55	4.92%	1.25E-07
GO:0048193~Golgi vesicle transport	13	1.16%	0.007169
GO:0045045~secretory pathway	24	2.15%	0.023947
GO:0046903~secretion	33	2.95%	0.032828
GO:0032940~secretion by cell	26	2.33%	0.044288

Median: 0.001635749236877623	Geo: 0.003254836007611388		
Term	Count	%	PValue
GO:0014069~postsynaptic density	8	0.72%	5.29E-04
GO:0042734~presynaptic membrane	7	0.63%	0.001636
GO:0032279~asymmetric synapse	3	0.27%	0.039821

Median: 0.004086253621505005	Geo: 0.00368195326563183		
Term	Count	%	PValue
GO:0006807~nitrogen compound metabolic p	44	3.94%	3.81E-04
GO:0006519~amino acid and derivative meta	38	3.40%	5.58E-04
GO:0009308~amine metabolic process	41	3.67%	7.89E-04
GO:0006520~amino acid metabolic process	27	2.42%	0.007384
GO:0019752~carboxylic acid metabolic proce	41	3.67%	0.044166
GO:0006082~organic acid metabolic process	41	3.67%	0.04559

Median: 0.002465966866617341	Geo: 0.003898690700606445		
Term	Count	%	PValue
GO:0031988~membrane-bound vesicle	32	2.86%	2.95E-04
GO:0016023~cytoplasmic membrane-bound v	30	2.68%	8.53E-04

GO:0031982~vesicle	32	2.86%	0.001726
GO:0031410~cytoplasmic vesicle	30	2.68%	0.002466
GO:0030135~coated vesicle	17	1.52%	0.008603
GO:0030136~clathrin-coated vesicle	15	1.34%	0.011561
GO:0008021~synaptic vesicle	8	0.72%	0.128535

Median: 0.00569726956942036l Geo: 0.003905295818069365

Term	Count	%	PValue
GO:0012505~endomembrane system	41	3.67%	4.89E-04
GO:0005789~endoplasmic reticulum membra	15	1.34%	0.004224
GO:0042175~nuclear envelope-endoplasmic r	15	1.34%	0.007171
GO:0044432~endoplasmic reticulum part	16	1.43%	0.01569

Median: 0.00580008303153623l Geo: 0.0045423103766470746

Term	Count	%	PValue
GO:0065003~macromolecular complex assen	42	3.76%	0.00193
GO:0022607~cellular component assembly	43	3.85%	0.0058
GO:0006461~protein complex assembly	26	2.33%	0.008372

Median: 0.003102987338819654 Geo: 0.005413943633266698

Term	Count	%	PValue
GO:0000166~nucleotide binding	139	12.43%	4.33E-05
GO:0032553~ribonucleotide binding	114	10.20%	6.94E-04
GO:0032555~purine ribonucleotide binding	114	10.20%	6.94E-04
GO:0017076~purine nucleotide binding	116	10.38%	0.001642
nucleotide-binding	68	6.08%	0.004564
GO:0032559~adenyl ribonucleotide binding	79	7.07%	0.140823
GO:0005524~ATP binding	77	6.89%	0.165516
GO:0030554~adenyl nucleotide binding	81	7.25%	0.202837

Median: 0.004872413994231359 Geo: 0.006380150986284573

Term	Count	%	PValue
GO:0004437~inositol or phosphatidylinositol p	9	0.81%	2.56E-04
SM00128:IPPc	5	0.45%	0.002727
GO:0046030~inositol trisphosphate phosphat	4	0.36%	0.004872
GO:0004445~inositol-polyphosphate 5-phospl	4	0.36%	0.004872
IPR000300:Inositol polyphosphate related pho	4	0.36%	0.011463
IPR005135:Endonuclease/exonuclease/phosph	4	0.36%	0.354689

Median: 0.01031240595268623 Geo: 0.00687033963953285

Term	Count	%	PValue
GO:0007049~cell cycle	50	4.47%	3.48E-04
GO:0007050~cell cycle arrest	10	0.89%	0.002501
GO:0045786~negative regulation of progress	15	1.34%	0.002807
GO:0022402~cell cycle process	39	3.49%	0.008623
GO:0000074~regulation of progression throu	26	2.33%	0.012002
GO:0051726~regulation of cell cycle	26	2.33%	0.013531
GO:0000278~mitotic cell cycle	23	2.06%	0.017947

GO:0022403~cell cycle phase	22	1.97%	0.080816

Median: 0.0028084324789411762	Geo: 0.008228285605659864		
Term	Count	%	PValue
GO:0048519~negative regulation of biologica	80	7.16%	0.00239
GO:0048523~negative regulation of cellular p	75	6.71%	0.002808
GO:0009892~negative regulation of metaboli	28	2.50%	0.082998

Median: 0.012960373553618398	Geo: 0.008546649490151655		
Term	Count	%	PValue
GO:0006457~protein folding	23	2.06%	0.001451
chaperone	10	0.89%	0.011169
GO:0051082~unfolded protein binding	12	1.07%	0.014752
IPR002423:Chaperonin Cpn60/TCP-1	4	0.36%	0.022313

Median: 0.9967247590148626	Geo: 0.01098528701294567		
Term	Count	%	PValue
GO:0007242~intracellular signaling cascade	114	10.20%	1.33E-06
GO:0007154~cell communication	218	19.50%	0.996725
GO:0007165~signal transduction	196	17.53%	0.997839

Median: 0.040660299161142804	Geo: 0.012755242088997738		
Term	Count	%	PValue
GO:0016044~membrane organization and bic	29	2.59%	0.001255
GO:0010324~membrane invagination	16	1.43%	0.04066
GO:0006897~endocytosis	16	1.43%	0.04066

Median: 0.10964998236347706	Geo: 0.014021621729689819		
Term	Count	%	PValue
GO:0005525~GTP binding	37	3.31%	7.53E-06
GO:0032561~guanyl ribonucleotide binding	37	3.31%	1.26E-05
GO:0019001~guanyl nucleotide binding	37	3.31%	1.46E-05
GTP-binding	26	2.33%	2.21E-04
GO:0003924~GTPase activity	21	1.88%	4.06E-04
IPR005225:Small GTP-binding protein domain	15	1.34%	0.007434
IPR001806:Ras GTPase	15	1.34%	0.007434
nucleotide phosphate-binding region:GTP	14	1.25%	0.023175
lipid moiety-binding region:S-geranylgeranyl c	7	0.63%	0.041332
short sequence motif:Effector region	7	0.63%	0.062708
GO:0017111~nucleoside-triphosphatase activ	37	3.31%	0.10965
GO:0016462~pyrophosphatase activity	38	3.40%	0.122742
GO:0016818~hydrolase activity, acting on aci	38	3.40%	0.12873
GO:0016817~hydrolase activity, acting on aci	38	3.40%	0.139562
prenylation	8	0.72%	0.152535
IPR013753:Ras	9	0.81%	0.152954
PIRSF037165:ras protein	3	0.27%	0.159725
propeptide:Removed in mature form	9	0.81%	0.175615
Methylation	10	0.89%	0.203346

IPR003577:Ras small GTPase, Ras type	3	0.27%	0.297638
SM00173:RAS	3	0.27%	0.305554

Median: 0.01276382284052376	Geo: 0.01838815303424093		
Term	Count	%	PValue
GO:0004774~succinate-CoA ligase activity	3	0.27%	0.008688
GO:0016405~CoA-ligase activity	4	0.36%	0.010767
GO:0016878~acid-thiol ligase activity	4	0.36%	0.014761
GO:0016877~ligase activity, forming carbon-s	4	0.36%	0.082806

Median: 0.017439820365672112	Geo: 0.024768054688680787		
Term	Count	%	PValue
IPR000504:RNA recognition motif, RNP-1	14	1.25%	0.012923
rna-binding	18	1.61%	0.014434
IPR012677:Nucleotide-binding, alpha-beta pla	14	1.25%	0.01744
SM00360:RRM	16	1.43%	0.042508
GO:0003723~RNA binding	36	3.22%	0.067407

Median: 0.035923506232415525	Geo: 0.0260307834995732		
Term	Count	%	PValue
GO:0044271~nitrogen compound biosyntheti	13	1.16%	0.010719
GO:0009309~amine biosynthetic process	10	0.89%	0.035924
GO:0008652~amino acid biosynthetic process	7	0.63%	0.045807

Median: 0.020676498351106368	Geo: 0.02792059126257564		
Term	Count	%	PValue
GO:0005794~Golgi apparatus	31	2.77%	0.015073
GO:0044431~Golgi apparatus part	19	1.70%	0.020676
GO:0000139~Golgi membrane	12	1.07%	0.069837

Median: 0.04179945450168271	Geo: 0.02866270308739085		
Term	Count	%	PValue
GO:0051020~GTPase binding	11	0.98%	0.005704
GO:0031267~small GTPase binding	9	0.81%	0.0147
GO:0017016~Ras GTPase binding	7	0.63%	0.068898
GO:0017048~Rho GTPase binding	4	0.36%	0.116835

Median: 0.05672383173871821	Geo: 0.03746482697220645		
Term	Count	%	PValue
GO:0007264~small GTPase mediated signal t	43	3.85%	9.56E-06
GO:0005083~small GTPase regulator activity	20	1.79%	7.72E-04
GO:0030695~GTPase regulator activity	27	2.42%	0.002797
GO:0005085~guanyl-nucleotide exchange fac	15	1.34%	0.007996
GO:0051056~regulation of small GTPase med	17	1.52%	0.017757
GO:0008047~enzyme activator activity	20	1.79%	0.02122
GO:0005088~Ras guanyl-nucleotide exchang	9	0.81%	0.025163
GO:0007265~Ras protein signal transduction	17	1.52%	0.031174
GO:0030234~enzyme regulator activity	47	4.20%	0.038539

GO:0046578~regulation of Ras protein signal	12	1.07%	0.039714
GO:0007266~Rho protein signal transduction	10	0.89%	0.056724
GO:0009966~regulation of signal transductio	35	3.13%	0.071205
GO:0005089~Rho guanyl-nucleotide exchang	7	0.63%	0.074098
GO:0005096~GTPase activator activity	12	1.07%	0.085486
GO:0035023~regulation of Rho protein signal	7	0.63%	0.112689
IPR011993:Pleckstrin homology-type	12	1.07%	0.182677
IPR001849:Pleckstrin-like	9	0.81%	0.407613
IPR000219:DH	3	0.27%	0.415812
SM00325:RhoGEF	3	0.27%	0.425544
SM00233:PH	9	0.81%	0.488891
domain:PH	3	0.27%	0.801822

Median: 0.05790452203551337 Geo: 0.03831891115558686

Term	Count	%	PValue
GO:0044463~cell projection part	14	1.25%	0.004533
GO:0043198~dendritic shaft	5	0.45%	0.015482
GO:0042995~cell projection	33	2.95%	0.033403
GO:0043197~dendritic spine	5	0.45%	0.082407
GO:0043005~neuron projection	18	1.61%	0.125864
GO:0030425~dendrite	11	0.98%	0.130214

Median: 0.029417122574276287 Geo: 0.03899252756753423

Term	Count	%	PValue
aminoacyl-tRNA synthetase	6	0.54%	0.018385
GO:0043039~tRNA aminoacylation	9	0.81%	0.024262
GO:0006418~tRNA aminoacylation for proteir	9	0.81%	0.024262
GO:0043038~amino acid activation	9	0.81%	0.024262
GO:0006399~tRNA metabolic process	12	1.07%	0.029253
GO:0016875~ligase activity, forming carbon-c	9	0.81%	0.029581
GO:0016876~ligase activity, forming aminoac	9	0.81%	0.029581
GO:0004812~aminoacyl-tRNA ligase activity	9	0.81%	0.029581
rno00970:Aminoacyl-tRNA biosynthesis	4	0.36%	0.116794
protein biosynthesis	11	0.98%	0.349878

Median: 0.07501409807774695 Geo: 0.04573781156503287

Term	Count	%	PValue
GO:0048193~Golgi vesicle transport	13	1.16%	0.007169
GO:0006888~ER to Golgi vesicle-mediated tra	7	0.63%	0.075014
er-golgi transport	5	0.45%	0.177915

Median: 0.06899050268680867 Geo: 0.046416713865919654

Term	Count	%	PValue
outer membrane	6	0.54%	0.010652
GO:0019867~outer membrane	9	0.81%	0.020773
GO:0005741~mitochondrial outer membrane	6	0.54%	0.068991
GO:0031968~organelle outer membrane	6	0.54%	0.118051
topological domain:Mitochondrial intermembra	4	0.36%	0.119556

Median: 0.06513242583564044 Geo: 0.05036987687278405

Term	Count	%	PValue
GO:0016874~ligase activity	34	3.04%	1.59E-04
GO:0016881~acid-amino acid ligase activity	15	1.34%	0.04563
GO:0019787~small conjugating protein ligase	14	1.25%	0.046039
GO:0016879~ligase activity, forming carbon-r	17	1.52%	0.047902
GO:0004842~ubiquitin-protein ligase activity	12	1.07%	0.050809
GO:0008639~small protein conjugating enzyr	12	1.07%	0.065132
GO:0006512~ubiquitin cycle	21	1.88%	0.111332
GO:0016567~protein ubiquitination	8	0.72%	0.143279
GO:0000209~protein polyubiquitination	3	0.27%	0.168192
rno04120:Ubiquitin mediated proteolysis	12	1.07%	0.18278
GO:0032446~protein modification by small pr	8	0.72%	0.204258

Median: 0.0316532809502792 Geo: 0.05575989202701649

Term	Count	%	PValue
GO:0048468~cell development	89	7.96%	0.005336
GO:0042981~regulation of apoptosis	44	3.94%	0.011114
GO:0043067~regulation of programmed cell (	44	3.94%	0.01401
GO:0008219~cell death	56	5.01%	0.014591
GO:0016265~death	56	5.01%	0.014591
GO:0048869~cellular developmental process	110	9.84%	0.021203
GO:0030154~cell differentiation	110	9.84%	0.021203
GO:0006915~apoptosis	52	4.65%	0.028182
GO:0012501~programmed cell death	52	4.65%	0.035124
GO:0043066~negative regulation of apoptosi	18	1.61%	0.138967
GO:0043069~negative regulation of programı	18	1.61%	0.146863
GO:0006916~anti-apoptosis	12	1.07%	0.266561
GO:0043065~positive regulation of apoptosis	18	1.61%	0.29451
GO:0043068~positive regulation of programn	18	1.61%	0.311435
GO:0006917~induction of apoptosis	13	1.16%	0.467993
GO:0012502~induction of programmed cell d	13	1.16%	0.475026

Median: 0.1562812317333729 Geo: 0.057707693908155444

Term	Count	%	PValue
GO:0005856~cytoskeleton	60	5.37%	0.002472
GO:0044430~cytoskeletal part	37	3.31%	0.093461
GO:0043232~intracellular non-membrane-bou	88	7.87%	0.219101
GO:0043228~non-membrane-bound organelle	88	7.87%	0.219101

Median: 0.06825579194341394 Geo: 0.06084916171997176

Term	Count	%	PValue
IPR006689:ARF/SAR superfamily	5	0.45%	0.041646
IPR006688:ADP-ribosylation factor	4	0.36%	0.066748
SM00177:ARF	4	0.36%	0.069764
PIRSF001711:ADP-ribosylation factor	4	0.36%	0.070693

Median: 0.142736866623424	Geo: 0.0615377040316669		
Term	Count	%	PValue
GO:0065007~biological regulation	277	24.78%	1.82E-05
GO:0050789~regulation of biological process	237	21.20%	6.06E-04
GO:0050794~regulation of cellular process	210	18.78%	0.001453
GO:0005634~nucleus	184	16.46%	0.005189
GO:0003676~nucleic acid binding	160	14.31%	0.018851
GO:0019222~regulation of metabolic process	125	11.18%	0.037235
GO:0006139~nucleobase, nucleoside, nucleot	171	15.30%	0.051081
GO:0003677~DNA binding	96	8.59%	0.081483
GO:0031323~regulation of cellular metabolic	115	10.29%	0.098182
GO:0010468~regulation of gene expression	107	9.57%	0.117897
GO:0010467~gene expression	153	13.69%	0.133621
GO:0016070~RNA metabolic process	116	10.38%	0.151853
Transcription regulation	35	3.13%	0.152907
Transcription	35	3.13%	0.176114
GO:0006350~transcription	100	8.94%	0.289473
GO:0045449~regulation of transcription	94	8.41%	0.302651
GO:0019219~regulation of nucleobase, nucle	95	8.50%	0.390213
GO:0006355~regulation of transcription, DNA	84	7.51%	0.393237
GO:0006351~transcription, DNA-dependent	86	7.69%	0.51837
GO:0032774~RNA biosynthetic process	86	7.69%	0.526486
GO:0030528~transcription regulator activity	66	5.90%	0.563567
GO:0003700~transcription factor activity	43	3.85%	0.721366

Median: 0.2068132443128243	Geo: 0.06384945590624247		
Term	Count	%	PValue
lipoprotein	35	3.13%	0.004159
lipid moiety-binding region:S-palmitoyl cysteir	12	1.07%	0.206813
Palmitate	12	1.07%	0.302654

Median: 0.05902448559063275	Geo: 0.06952667627562897		
Term	Count	%	PValue
GO:0008270~zinc ion binding	90	8.05%	0.027333
GO:0043167~ion binding	172	15.38%	0.047907
GO:0046872~metal ion binding	167	14.94%	0.059024
GO:0046914~transition metal ion binding	109	9.75%	0.084958
GO:0043169~cation binding	150	13.42%	0.247418

Median: 0.08069942792163615	Geo: 0.07022645291284828		
Term	Count	%	PValue
lipid moiety-binding region:N-myristoyl glycine	9	0.81%	0.01454
myristate	9	0.81%	0.017341
myristylation	6	0.54%	0.018385
blocked amino end	6	0.54%	0.143013
nucleotide binding	6	0.54%	0.406721
P-loop	6	0.54%	0.444845

Median: 0.052526849789520304	Geo: 0.07343970394205135		
Term	Count	%	PValue
GO:0006584~catecholamine metabolic proce	6	0.54%	0.019824
GO:0018958~phenol metabolic process	6	0.54%	0.02286
GO:0006576~biogenic amine metabolic proce	10	0.89%	0.022861
GO:0006575~amino acid derivative metabolic	12	1.07%	0.052527
GO:0042133~neurotransmitter metabolic pro	5	0.45%	0.181854
GO:0042417~dopamine metabolic process	3	0.27%	0.297004
GO:0006725~aromatic compound metabolic p	10	0.89%	0.392012

Median: 0.08857971092806124	Geo: 0.07428537664101921		
Term	Count	%	PValue
rno04130:SNARE interactions in vesicular trar	7	0.63%	0.027406
IPR006012:Syntaxin/epimorphin coiled-coil	4	0.36%	0.046104
domain:t-SNARE coiled-coil homology	4	0.36%	0.053705
IPR006011:Syntaxin, N-terminal	3	0.27%	0.08858
SM00503:SynN	3	0.27%	0.091493
IPR000727:Target SNARE coiled-coil region	4	0.36%	0.147697
SM00397:t_SNARE	4	0.36%	0.153687

Median: 0.062429438292995174	Geo: 0.07578489960391194		
Term	Count	%	PValue
lipid moiety-binding region:N-myristoyl glycine	9	0.81%	0.01454
myristate	9	0.81%	0.017341
calcium-binding region:1	8	0.72%	0.019181
domain:EF-hand 3	7	0.63%	0.02202
calcium-binding region:2	7	0.63%	0.046167
IPR002048:Calcium-binding EF-hand	14	1.25%	0.056269
domain:EF-hand 2	9	0.81%	0.057434
domain:EF-hand 4	5	0.45%	0.058266
IPR011992:EF-Hand type	14	1.25%	0.062354
SM00054:EFh	14	1.25%	0.062505
PIRSF002350:calmodulin	4	0.36%	0.070693
domain:EF-hand 1	8	0.72%	0.090052
calcium	30	2.68%	0.152543
calcium-binding region:3	3	0.27%	0.246277
IPR001125:Recoverin	3	0.27%	0.273309
Signal transduction mechanisms / Cytoskeleto	6	0.54%	0.282244
EF hand	4	0.36%	0.294715
calcium binding	5	0.45%	0.346603

Median: 0.08419109283311178	Geo: 0.07865101152463738		
Term	Count	%	PValue
GO:0008289~lipid binding	27	2.42%	0.034511
GO:0005543~phospholipid binding	15	1.34%	0.084191
GO:0035091~phosphoinositide binding	10	0.89%	0.167453

Median: 0.06749757640491225 Geo: 0.08163632183901466

Term	Count	%	PValue
GO:0006396~RNA processing	26	2.33%	0.021741
GO:0006397~mRNA processing	16	1.43%	0.027483
mrna processing	10	0.89%	0.03976
GO:0016071~mRNA metabolic process	16	1.43%	0.095236
mrna splicing	6	0.54%	0.279462
GO:0008380~RNA splicing	9	0.81%	0.468157

Median: 0.08367445242528601 Geo: 0.09325118835443283

Term	Count	%	PValue
GO:0019226~transmission of nerve impulse	39	3.49%	0.008986
GO:0007268~synaptic transmission	35	3.13%	0.009379
GO:0007267~cell-cell signaling	49	4.38%	0.083674
GO:0003008~system process	69	6.17%	1
GO:0050877~neurological system process	57	5.10%	1

Median: 0.10197451032252433 Geo: 0.09443930654308128

Term	Count	%	PValue
GO:0008285~negative regulation of cell prolif	22	1.97%	0.021171
GO:0042127~regulation of cell proliferation	37	3.31%	0.101975
GO:0008283~cell proliferation	45	4.03%	0.390141

Median: 0.07380752782690993 Geo: 0.09919375989807463

Term	Count	%	PValue
GO:0006536~glutamate metabolic process	3	0.27%	0.072716
rno00251:Glutamate metabolism	5	0.45%	0.073808
GO:0009064~glutamine family amino acid me	5	0.45%	0.181854

Median: 0.15290711979150115 Geo: 0.10087618409325069

Term	Count	%	PValue
repressor	12	1.07%	0.02792
nucleus	91	8.14%	0.033644
Transcription regulation	35	3.13%	0.152907
Transcription	35	3.13%	0.176114
dna-binding	34	3.04%	0.412953

Median: 0.1309296131274359 Geo: 0.10616709332351958

Term	Count	%	PValue
domain:BAR	3	0.27%	0.057758
IPR004148:BAR	3	0.27%	0.13093
SM00721:BAR	3	0.27%	0.158241

Median: 0.10584057003619658 Geo: 0.10679216096906936

Term	Count	%	PValue
GO:0005100~Rho GTPase activator activity	4	0.36%	0.053969
GO:0005096~GTPase activator activity	12	1.07%	0.085486
GO:0043087~regulation of GTPase activity	7	0.63%	0.105841
gtpase activation	5	0.45%	0.141009

GO:0005099~Ras GTPase activator activity	6	0.54%	0.201724

Median: 0.10189181414834529 Geo: 0.10949812218825965

Term	Count	%	PValue
GO:0019725~cellular homeostasis	29	2.59%	0.014671
GO:0042592~homeostatic process	36	3.22%	0.02805
GO:0006875~cellular metal ion homeostasis	14	1.25%	0.088302
GO:0055065~metal ion homeostasis	14	1.25%	0.088302
GO:0006873~cellular ion homeostasis	20	1.79%	0.091552
GO:0055082~cellular chemical homeostasis	20	1.79%	0.091552
GO:0006874~cellular calcium ion homeostasi	13	1.16%	0.093013
GO:0055074~calcium ion homeostasis	13	1.16%	0.093013
GO:0065008~regulation of biological quality	59	5.28%	0.101892
GO:0050801~ion homeostasis	21	1.88%	0.117543
GO:0030003~cellular cation homeostasis	17	1.52%	0.128115
GO:0055080~cation homeostasis	17	1.52%	0.128115
GO:0048878~chemical homeostasis	22	1.97%	0.204232
GO:0030005~cellular di-, tri-valent inorganic	14	1.25%	0.219321
GO:0055066~di-, tri-valent inorganic cation h	14	1.25%	0.219321
GO:0007204~elevation of cytosolic calcium ic	7	0.63%	0.307615
GO:0051480~cytosolic calcium ion homeosta	7	0.63%	0.338314

Median: 0.10842151729013733 Geo: 0.11186370049581089

Term	Count	%	PValue
GO:0032012~regulation of ARF protein signal	3	0.27%	0.108422
GO:0032011~ARF protein signal transduction	3	0.27%	0.108422
GO:0005086~ARF guanyl-nucleotide exchang	3	0.27%	0.119079

Median: 0.1076855020720717 Geo: 0.11547631425780384

Term	Count	%	PValue
rno00510:N-Glycan biosynthesis	8	0.72%	0.013869
GO:0009100~glycoprotein metabolic process	13	1.16%	0.054018
rno01030:Glycan structures - biosynthesis 1	12	1.07%	0.088684
GO:0016757~transferase activity, transferring	17	1.52%	0.098527
GO:0006486~protein amino acid glycosylatio	10	0.89%	0.107686
GO:0043413~biopolymer glycosylation	10	0.89%	0.118084
GO:0009101~glycoprotein biosynthetic proce	10	0.89%	0.152395
GO:0016758~transferase activity, transferring	10	0.89%	0.369148
glycosyltransferase	7	0.63%	0.779679

Median: 0.1258641009200506 Geo: 0.11806714660258627

Term	Count	%	PValue
GO:0030424~axon	10	0.89%	0.076941
GO:0043005~neuron projection	18	1.61%	0.125864
GO:0043025~cell soma	10	0.89%	0.169952

Median: 0.1677959048829996 Geo: 0.11965887011097136

Term	Count	%	PValue

GO:0007010~cytoskeleton organization and b	37	3.31%	0.029817
GO:0007015~actin filament organization	7	0.63%	0.092817
GO:0030036~actin cytoskeleton organization	14	1.25%	0.242775
GO:0030029~actin filament-based process	14	1.25%	0.305131

Median: 0.18185380918816363 Geo: 0.12058492068067901

Term	Count	%	PValue
GO:0008652~amino acid biosynthetic process	7	0.63%	0.045807
GO:0009064~glutamine family amino acid me	5	0.45%	0.181854
GO:0009084~glutamine family amino acid bic	3	0.27%	0.210485

Median: 0.13346020508450196 Geo: 0.1210625157076649

Term	Count	%	PValue
GO:0051246~regulation of protein metabolic	22	1.97%	0.040026
GO:0022618~protein-RNA complex assembly	10	0.89%	0.053362
GO:0009889~regulation of biosynthetic proce	16	1.43%	0.060281
GO:0006413~translational initiation	7	0.63%	0.092817
GO:0008135~translation factor activity, nucle	9	0.81%	0.104059
GO:0003743~translation initiation factor activ	6	0.54%	0.129377
GO:0006446~regulation of translational initia	5	0.45%	0.137543
GO:0022613~ribonucleoprotein complex biog	12	1.07%	0.150133
GO:0045182~translation regulator activity	9	0.81%	0.175896
GO:0031326~regulation of cellular biosynthet	12	1.07%	0.207834
initiation factor	4	0.36%	0.277076
GO:0006417~regulation of translation	10	0.89%	0.294521

Median: 0.1803019793844538 Geo: 0.12215092015596596

Term	Count	%	PValue
GO:0051301~cell division	16	1.43%	0.002944
GO:0000278~mitotic cell cycle	23	2.06%	0.017947
GO:0022403~cell cycle phase	22	1.97%	0.080816
cell cycle	15	1.34%	0.121315
cell division	9	0.81%	0.125236
GO:0051329~interphase of mitotic cell cycle	8	0.72%	0.172594
GO:0051325~interphase	8	0.72%	0.180302
GO:0051318~G1 phase	3	0.27%	0.210485
GO:0000279~M phase	15	1.34%	0.249235
GO:0007067~mitosis	11	0.98%	0.302128
mitosis	5	0.45%	0.302678
GO:0000087~M phase of mitotic cell cycle	11	0.98%	0.332362
GO:0000082~G1/S transition of mitotic cell cy	4	0.36%	0.418699

Median: 0.11267688765907659 Geo: 0.1268010744333647

Term	Count	%	PValue
GO:0035258~steroid hormone receptor bindir	5	0.45%	0.025487
GO:0030518~steroid hormone receptor signa	6	0.54%	0.047282
GO:0050681~androgen receptor binding	4	0.36%	0.053969
GO:0003713~transcription coactivator activity	14	1.25%	0.060449

GO:0030522~intracellular receptor-mediated	6	0.54%	0.097158
GO:0030521~androgen receptor signaling pat	4	0.36%	0.128196
GO:0035257~nuclear hormone receptor bindi	6	0.54%	0.211598
GO:0051427~hormone receptor binding	6	0.54%	0.242086
GO:0046966~thyroid hormone receptor bindi	3	0.27%	0.610061
GO:0045944~positive regulation of transcript	10	0.89%	0.702208

Median: 0.10065090511468879 Geo: 0.1276364001100667

Term	Count	%	PValue
pyridoxal phosphate	7	0.63%	0.047012
GO:0008483~transaminase activity	5	0.45%	0.051374
aminotransferase	4	0.36%	0.076229
GO:0016769~transferase activity, transferring	5	0.45%	0.095855
IPR015421:Pyridoxal phosphate-dependent tra	5	0.45%	0.100651
GO:0019842~vitamin binding	10	0.89%	0.155451
GO:0030170~pyridoxal phosphate binding	5	0.45%	0.256409
binding site:Pyridoxal phosphate (covalent)	4	0.36%	0.258983
GO:0048037~cofactor binding	14	1.25%	0.490306

Median: 0.09004221777709542 Geo: 0.13237772337733444

Term	Count	%	PValue
GO:0048583~regulation of response to stimul	9	0.81%	0.01677
GO:0032107~regulation of response to nutrie	3	0.27%	0.072716
GO:0032104~regulation of response to extrac	3	0.27%	0.072716
GO:0032095~regulation of response to food	3	0.27%	0.072716
GO:0032101~regulation of response to exterr	3	0.27%	0.090042
GO:0032094~response to food	3	0.27%	0.127677
GO:0031667~response to nutrient levels	8	0.72%	0.507172
GO:0009991~response to extracellular stimul	8	0.72%	0.552089
GO:0007584~response to nutrient	6	0.54%	0.601449

Median: 0.24146442504751328 Geo: 0.1327707699357756

Term	Count	%	PValue
GO:0032502~developmental process	184	16.46%	0.015368
GO:0048869~cellular developmental process	110	9.84%	0.021203
GO:0030154~cell differentiation	110	9.84%	0.021203
GO:0007275~multicellular organismal develo	131	11.72%	0.100626
GO:0048856~anatomical structure developme	118	10.55%	0.382303
GO:0048731~system development	101	9.03%	0.421206
GO:0048513~organ development	65	5.81%	0.862571
GO:0032501~multicellular organismal proces	185	16.55%	0.999998

Median: 0.17519858930582505 Geo: 0.1329722840960057

Term	Count	%	PValue
GO:0006626~protein targeting to mitochondr	5	0.45%	0.017079
GO:0006839~mitochondrial transport	6	0.54%	0.082855
GO:0007005~mitochondrion organization and	7	0.63%	0.267542
GO:0044455~mitochondrial membrane part	3	0.27%	0.825805

Median: 0.10305094387637022 Geo: 0.1330202062600308

Term	Count	%	PValue
GO:0001937~negative regulation of endothel	3	0.27%	0.090042
GO:0001935~endothelial cell proliferation	4	0.36%	0.103051
GO:0001936~regulation of endothelial cell pr	3	0.27%	0.253662

Median: 0.11990710670990666 Geo: 0.13685778237651414

Term	Count	%	PValue
GO:0007187~G-protein signaling, coupled to	14	1.25%	0.031366
GO:0019935~cyclic-nucleotide-mediated sign	14	1.25%	0.065869
GO:0007188~G-protein signaling, coupled to	11	0.98%	0.066354
GO:0045761~regulation of adenylate cyclase	6	0.54%	0.097158
GO:0051339~regulation of lyase activity	6	0.54%	0.11262
GO:0031279~regulation of cyclase activity	6	0.54%	0.11262
GO:0019933~cAMP-mediated signaling	11	0.98%	0.127194
GO:0007190~adenylate cyclase activation	4	0.36%	0.213759
GO:0051349~positive regulation of lyase acti	4	0.36%	0.244569
GO:0045762~positive regulation of adenylate	4	0.36%	0.244569
GO:0031281~positive regulation of cyclase a	4	0.36%	0.260231
GO:0007189~G-protein signaling, adenylate c	4	0.36%	0.603889

Median: 0.0908459699550602 Geo: 0.13704169444018185

Term	Count	%	PValue
IPR003958:Transcription factor CBF/NF-Y/arch	3	0.27%	0.035936
IPR009072:Histone-fold	4	0.36%	0.090846
Transcription	3	0.27%	0.788366

Median: 0.13734646667265776 Geo: 0.14002507969953631

Term	Count	%	PValue
GO:0005798~Golgi-associated vesicle	8	0.72%	0.034523
GO:0012506~vesicle membrane	11	0.98%	0.037788
IPR008152:Clathrin adaptor, alpha/beta/gamn	3	0.27%	0.051649
SM00809:Alpha_adaptinC2	3	0.27%	0.053424
GO:0030659~cytoplasmic vesicle membrane	8	0.72%	0.092881
GO:0030133~transport vesicle	6	0.54%	0.095371
GO:0044433~cytoplasmic vesicle part	9	0.81%	0.109754
GO:0048475~coated membrane	6	0.54%	0.134454
GO:0030117~membrane coat	6	0.54%	0.134454
GO:0030118~clathrin coat	5	0.45%	0.137346
GO:0030140~trans-Golgi network transport v	4	0.36%	0.159338
GO:0030130~clathrin coat of trans-Golgi netw	3	0.27%	0.201342
GO:0030120~vesicle coat	5	0.45%	0.239078
GO:0012510~trans-Golgi network transport v	3	0.27%	0.243116
GO:0030125~clathrin vesicle coat	4	0.36%	0.276901
GO:0030662~coated vesicle membrane	5	0.45%	0.288866
GO:0030665~clathrin coated vesicle membra	4	0.36%	0.307779
GO:0030658~transport vesicle membrane	3	0.27%	0.368185

GO:0030660~Golgi-associated vesicle membr	3	0.27%	0.408313

Median: 0.1361326064824238	Geo: 0.1440097981822845		
Term	Count	%	PValue
GO:0002761~regulation of myeloid leukocyte	6	0.54%	0.008587
GO:0002762~negative regulation of myeloid l	4	0.36%	0.016525
GO:0045637~regulation of myeloid cell differe	7	0.63%	0.024981
GO:0002573~myeloid leukocyte differentiatio	6	0.54%	0.063669
GO:0045638~negative regulation of myeloid c	4	0.36%	0.069637
GO:0045670~regulation of osteoclast differen	4	0.36%	0.069637
GO:0030316~osteoclast differentiation	4	0.36%	0.091299
GO:0048534~hemopoietic or lymphoid organ	17	1.52%	0.093025
GO:0030097~hemopoiesis	16	1.43%	0.098623
GO:0002520~immune system development	17	1.52%	0.116874
GO:0051093~negative regulation of developm	9	0.81%	0.136133
GO:0030099~myeloid cell differentiation	9	0.81%	0.155467
GO:0045639~positive regulation of myeloid c	3	0.27%	0.253662
GO:0050793~regulation of developmental pro	20	1.79%	0.270145
GO:0051094~positive regulation of developm	8	0.72%	0.319107
GO:0045596~negative regulation of cell differ	6	0.54%	0.33732
GO:0002521~leukocyte differentiation	8	0.72%	0.385125
GO:0045595~regulation of cell differentiation	12	1.07%	0.477352
GO:0045597~positive regulation of cell differe	4	0.36%	0.754627
GO:0045165~cell fate commitment	3	0.27%	0.955029
GO:0002376~immune system process	33	2.95%	0.956468

Median: 0.3508793236375377	Geo: 0.14658367233857106		
Term	Count	%	PValue
GO:0048489~synaptic vesicle transport	9	0.81%	0.004764
GO:0001505~regulation of neurotransmitter l	13	1.16%	0.089241
GO:0016079~synaptic vesicle exocytosis	4	0.36%	0.16939
GO:0003001~generation of a signal involved	10	0.89%	0.350879
GO:0045055~regulated secretory pathway	8	0.72%	0.356681
GO:0007269~neurotransmitter secretion	7	0.63%	0.390062
GO:0006887~exocytosis	8	0.72%	0.413622

Median: 0.43415643114745134	Geo: 0.14675904481537738		
Term	Count	%	PValue
GO:0048583~regulation of response to stimul	9	0.81%	0.01677
GO:0031347~regulation of defense response	4	0.36%	0.434156
GO:0050727~regulation of inflammatory resp	4	0.36%	0.434156

Median: 0.24998858310372712	Geo: 0.15290988756460436		
Term	Count	%	PValue
wd repeat	13	1.16%	0.025128
IPR001680:WD40 repeat	13	1.16%	0.035564
SM00320:WD40	13	1.16%	0.053411
repeat:WD 6	5	0.45%	0.170135

repeat:WD 5	5	0.45%	0.242372
repeat:WD 4	5	0.45%	0.257606
repeat:WD 3	5	0.45%	0.288595
repeat:WD 2	5	0.45%	0.304286
repeat:WD 1	5	0.45%	0.304286
repeat:WD 7	3	0.27%	0.5158

Median: 0.15565765283845925 Geo: 0.1529854602605251

Term	Count	%	PValue
GO:0051186~cofactor metabolic process	20	1.79%	0.055485
GO:0006732~coenzyme metabolic process	17	1.52%	0.067256
GO:0051188~cofactor biosynthetic process	9	0.81%	0.244059
GO:0009108~coenzyme biosynthetic process	6	0.54%	0.601449

Median: 0.15899314901573366 Geo: 0.15637827340600607

Term	Count	%	PValue
GO:0000302~response to reactive oxygen sp	6	0.54%	0.069757
GO:0009725~response to hormone stimulus	15	1.34%	0.083759
GO:0043627~response to estrogen stimulus	6	0.54%	0.146779
GO:0006979~response to oxidative stress	10	0.89%	0.171207
GO:0042542~response to hydrogen peroxide	4	0.36%	0.183882
GO:0048545~response to steroid hormone sti	7	0.63%	0.541646

Median: 0.3508793236375377 Geo: 0.1679204348599385

Term	Count	%	PValue
GO:0065009~regulation of a molecular functi	40	3.58%	0.01017
GO:0050790~regulation of catalytic activity	36	3.22%	0.012368
GO:0043085~positive regulation of catalytic a	22	1.97%	0.029565
GO:0043549~regulation of kinase activity	17	1.52%	0.156675
GO:0051338~regulation of transferase activit	17	1.52%	0.188353
GO:0045859~regulation of protein kinase acti	16	1.43%	0.201339
GO:0033674~positive regulation of kinase act	10	0.89%	0.350879
GO:0051347~positive regulation of transferas	10	0.89%	0.392012
GO:0007200~G-protein signaling, coupled to	7	0.63%	0.400431
GO:0032147~activation of protein kinase acti	5	0.45%	0.438703
GO:0045860~positive regulation of protein ki	9	0.81%	0.459439
GO:0007205~protein kinase C activation	3	0.27%	0.553633
GO:0000165~MAPKKK cascade	10	0.89%	0.621474

Median: 0.200554335921077 Geo: 0.16822064139055293

Term	Count	%	PValue
PIRSF001741:membrane-associated guanylate	3	0.27%	0.023567
SM00072:GuKc	5	0.45%	0.026247
GO:0008022~protein C-terminus binding	8	0.72%	0.029859
domain:Guanylate kinase-like	3	0.27%	0.057758
domain:SH3	7	0.63%	0.075448
IPR008145:Guanylate kinase/L-type calcium c	4	0.36%	0.07839
domain:PDZ 3	3	0.27%	0.121188

GO:0030165~PDZ domain binding	6	0.54%	0.17311
IPR008144:Guanylate kinase	3	0.27%	0.200554
domain:PDZ 1	3	0.27%	0.29823
domain:PDZ 2	3	0.27%	0.29823
IPR000108:Neutrophil cytosol factor 2	3	0.27%	0.345817
IPR001452:Src homology-3	9	0.81%	0.397693
SM00326:SH3	9	0.81%	0.46892
SH3 domain	8	0.72%	0.47937
IPR001478:PDZ/DHR/GLGF	3	0.27%	0.971386
SM00228:PDZ	3	0.27%	0.975655

Median: 0.10305094387637022 Geo: 0.1746413970914796

Term	Count	%	PValue
GO:0010003~gastrulation (sensu Mammalia)	4	0.36%	0.042285
GO:0001702~gastrulation with mouth forming	4	0.36%	0.103051
GO:0048276~gastrulation (sensu Vertebrata)	4	0.36%	0.103051
GO:0007369~gastrulation	4	0.36%	0.479537
GO:0003002~regionalization	6	0.54%	0.754437

Median: 0.146778845974888 Geo: 0.175730085063464

Term	Count	%	PValue
GO:0014070~response to organic cyclic subst	6	0.54%	0.076153
GO:0043627~response to estrogen stimulus	6	0.54%	0.146779
GO:0042493~response to drug	9	0.81%	0.485498

Median: 0.2310913261201935 Geo: 0.17766634724831248

Term	Count	%	PValue
GO:0008270~zinc ion binding	90	8.05%	0.027333
zinc-finger	34	3.04%	0.155905
zinc	45	4.03%	0.306277
metal-binding	60	5.37%	0.763395

Median: 0.21535379537979749 Geo: 0.17768583122794948

Term	Count	%	PValue
GO:0016323~basolateral plasma membrane	13	1.16%	0.010827
GO:0005912~adherens junction	9	0.81%	0.055736
GO:0005925~focal adhesion	5	0.45%	0.158264
GO:0005924~cell-substrate adherens junction	5	0.45%	0.191629
GO:0030055~cell-matrix junction	5	0.45%	0.239078
GO:0005913~cell-cell adherens junction	3	0.27%	0.447158
GO:0030054~cell junction	11	0.98%	0.58202
GO:0005911~intercellular junction	6	0.54%	0.872536

Median: 0.15546695149387 Geo: 0.17829203345072625

Term	Count	%	PValue
GO:0005184~neuropeptide hormone activity	5	0.45%	0.057794
GO:0007218~neuropeptide signaling pathway	9	0.81%	0.155467
GO:0005179~hormone activity	8	0.72%	0.630778

Median: 0.1719070097875019 Geo: 0.17861075696769138

Term	Count	%	PValue
serine/threonine-specific protein kinase	6	0.54%	0.054413
PIRSF000564:kinase-related transforming prot	4	0.36%	0.171907
rno04340:Hedgehog signaling pathway	4	0.36%	0.609158

Median: 0.48112562038876727 Geo: 0.1787057436525346

Term	Count	%	PValue
GO:0044456~synapse part	19	1.70%	2.22E-04
GO:0045202~synapse	27	2.42%	3.62E-04
GO:0045211~postsynaptic membrane	12	1.07%	0.018502
GO:0008144~drug binding	4	0.36%	0.240467
synapse	13	1.16%	0.283011
Postsynaptic cell membrane	8	0.72%	0.384888
GO:0030534~adult behavior	4	0.36%	0.403104
neurotransmitter receptor	5	0.45%	0.462802
GO:0005230~extracellular ligand-gated ion cl	5	0.45%	0.481126
GO:0030594~neurotransmitter receptor activ	7	0.63%	0.565097
cell junction	14	1.25%	0.609355
GO:0042165~neurotransmitter binding	7	0.63%	0.642168
IPR006202:Neurotransmitter-gated ion-chann	3	0.27%	0.748186
IPR006029:Neurotransmitter-gated ion-chann	3	0.27%	0.748186
IPR006201:Neurotransmitter-gated ion-chann	3	0.27%	0.748186
GO:0015276~ligand-gated ion channel activit	5	0.45%	0.772342
GO:0022834~ligand-gated channel activity	5	0.45%	0.772342

Median: 0.1803019793844538 Geo: 0.18582633481789906

Term	Count	%	PValue
GO:0007270~nerve-nerve synaptic transmiss	6	0.54%	0.097158
GO:0007612~learning	5	0.45%	0.107637
GO:0048169~regulation of long-term neurona	3	0.27%	0.147649
GO:0007611~learning and/or memory	8	0.72%	0.180302
GO:0048167~regulation of synaptic plasticity	6	0.54%	0.194764
GO:0050803~regulation of synapse structure	6	0.54%	0.247342
GO:0048168~regulation of neuronal synaptic	3	0.27%	0.57052

Median: 0.21008051367698122 Geo: 0.18658182674770896

Term	Count	%	PValue
GO:0006605~protein targeting	20	1.79%	0.046078
GO:0017038~protein import	9	0.81%	0.142436
GO:0006913~nucleocytoplasmic transport	11	0.98%	0.184392
GO:0051169~nuclear transport	11	0.98%	0.210081
GO:0006606~protein import into nucleus	7	0.63%	0.267542
GO:0051170~nuclear import	7	0.63%	0.287429
GO:0000060~protein import into nucleus, trai	3	0.27%	0.402649

Median: 0.19868478281630433 Geo: 0.1885086380593916

Term	Count	%	PValue
GO:0006665~sphingolipid metabolic process	6	0.54%	0.137856
GO:0006672~ceramide metabolic process	4	0.36%	0.198685
GO:0046519~sphingoid metabolic process	4	0.36%	0.244569

Median: 0.22585340278900806 Geo: 0.18852812202274055

Term	Count	%	PValue
GO:0015833~peptide transport	8	0.72%	0.039498
GO:0046883~regulation of hormone secretior	5	0.45%	0.089544
GO:0002790~peptide secretion	5	0.45%	0.107637
GO:0046888~negative regulation of hormone	3	0.27%	0.189177
GO:0030073~insulin secretion	4	0.36%	0.198685
GO:0046879~hormone secretion	6	0.54%	0.225853
GO:0050796~regulation of insulin secretion	3	0.27%	0.232012
GO:0030072~peptide hormone secretion	4	0.36%	0.260231
GO:0003001~generation of a signal involved	10	0.89%	0.350879
GO:0051046~regulation of secretion	7	0.63%	0.369321
GO:0051048~negative regulation of secretior	3	0.27%	0.422933

Median: 0.20076639204212415 Geo: 0.19510207483180358

Term	Count	%	PValue
IPR001841:Zinc finger, RING-type	11	0.98%	0.152621
SM00184:RING	11	0.98%	0.200766
zinc finger region:RING-type	5	0.45%	0.242372

Median: 0.17259375798038148 Geo: 0.19665995517085125

Term	Count	%	PValue
GO:0040011~locomotion	9	0.81%	0.096192
GO:0030334~regulation of cell migration	7	0.63%	0.1582
GO:0040013~negative regulation of locomoti	4	0.36%	0.16939
GO:0040012~regulation of locomotion	8	0.72%	0.172594
GO:0051270~regulation of cell motility	7	0.63%	0.238419
GO:0030336~negative regulation of cell migr	3	0.27%	0.297004
GO:0051271~negative regulation of cell motil	3	0.27%	0.361117

Median: 0.1927075177175326 Geo: 0.19770529455139862

Term	Count	%	PValue
GO:0009056~catabolic process	46	4.11%	0.060783
GO:0044248~cellular catabolic process	39	3.49%	0.068095
GO:0030163~protein catabolic process	16	1.43%	0.12049
GO:0006511~ubiquitin-dependent protein cat	12	1.07%	0.134647
GO:0043632~modification-dependent macror	12	1.07%	0.144872
GO:0019941~modification-dependent protein	12	1.07%	0.144872
GO:0051603~proteolysis involved in cellular p	12	1.07%	0.166495
GO:0044257~cellular protein catabolic proces	12	1.07%	0.183691
GO:0004843~ubiquitin-specific protease activ	6	0.54%	0.201724
GO:0019783~small conjugating protein-speci	6	0.54%	0.211598
GO:0016790~thiolester hydrolase activity	7	0.63%	0.228439

GO:0004221~ubiquitin thiolesterase activity	5	0.45%	0.318674
GO:0043285~biopolymer catabolic process	16	1.43%	0.421516
GO:0044265~cellular macromolecule cataboli	19	1.70%	0.425693
GO:0009057~macromolecule catabolic proces	23	2.06%	0.437564
GO:0008234~cysteine-type peptidase activity	10	0.89%	0.518148

Median: 0.3402131629373112 Geo: 0.20091348973280643

Term	Count	%	PValue
GO:0009058~biosynthetic process	98	8.77%	0.01976
GO:0044249~cellular biosynthetic process	67	5.99%	0.249013
GO:0009059~macromolecule biosynthetic prc	50	4.47%	0.431413
GO:0006412~translation	31	2.77%	0.767586

Median: 0.15219034199850132 Geo: 0.20388043471441814

Term	Count	%	PValue
GO:0009892~negative regulation of metaboli	28	2.50%	0.082998
GO:0000122~negative regulation of transcrip	12	1.07%	0.106427
GO:0016481~negative regulation of transcrip	20	1.79%	0.108749
GO:0045892~negative regulation of transcrip	15	1.34%	0.145618
GO:0031324~negative regulation of cellular r	24	2.15%	0.15219
GO:0045934~negative regulation of nucleoba	20	1.79%	0.171513
GO:0016564~transcription repressor activity	14	1.25%	0.250078
GO:0006357~regulation of transcription from	23	2.06%	0.714292
GO:0006366~transcription from RNA polymer	25	2.24%	0.933218

Median: 0.20425774245182154 Geo: 0.2051856545880777

Term	Count	%	PValue
GO:0043549~regulation of kinase activity	17	1.52%	0.156675
GO:0051338~regulation of transferase activit	17	1.52%	0.188353
GO:0045859~regulation of protein kinase acti	16	1.43%	0.201339
GO:0043086~negative regulation of catalytic	8	0.72%	0.204258
GO:0006469~negative regulation of protein k	6	0.54%	0.225853
GO:0033673~negative regulation of kinase ac	6	0.54%	0.225853
GO:0051348~negative regulation of transfera	6	0.54%	0.247342

Median: 0.24894442110152143 Geo: 0.21358808151445924

Term	Count	%	PValue
IPR013753:Ras	9	0.81%	0.152954
IPR003578:Ras small GTPase, Rho type	3	0.27%	0.248944
SM00174:RHO	3	0.27%	0.255899

Median: 0.23201158734523689 Geo: 0.21679040728369364

Term	Count	%	PValue
GO:0006665~sphingolipid metabolic process	6	0.54%	0.137856
GO:0006687~glycosphingolipid metabolic pro	3	0.27%	0.232012
GO:0006664~glycolipid metabolic process	3	0.27%	0.318554

Median: 0.2103440663828989 Geo: 0.21685062964976048

Term	Count	%	PValue
GO:0050804~regulation of synaptic transmiss	6	0.54%	0.204959
GO:0031644~regulation of neurological proce	7	0.63%	0.210344
GO:0051969~regulation of transmission of ne	6	0.54%	0.236529

Median: 0.23477854425515898 Geo: 0.22626350310823068

Term	Count	%	PValue
GO:0016477~cell migration	21	1.88%	0.210149
GO:0051674~localization of cell	27	2.42%	0.234779
GO:0006928~cell motility	27	2.42%	0.234779

Median: 0.213759410366611 Geo: 0.22973313871178894

Term	Count	%	PValue
GO:0006099~tricarboxylic acid cycle	4	0.36%	0.155251
GO:0046356~acetyl-CoA catabolic process	4	0.36%	0.16939
GO:0006084~acetyl-CoA metabolic process	5	0.45%	0.193598
GO:0009109~coenzyme catabolic process	4	0.36%	0.213759
GO:0009060~aerobic respiration	4	0.36%	0.260231
GO:0051187~cofactor catabolic process	4	0.36%	0.307828
GO:0045333~cellular respiration	4	0.36%	0.387391

Median: 0.44053360278966125 Geo: 0.22979315384796073

Term	Count	%	PValue
GO:0060090~molecular adaptor activity	6	0.54%	0.04997
GO:0005070~SH3/SH2 adaptor activity	3	0.27%	0.440534
GO:0030674~protein binding, bridging	5	0.45%	0.551213

Median: 0.3185542039557815 Geo: 0.2348694799133672

Term	Count	%	PValue
GO:0045428~regulation of nitric oxide biosyn	3	0.27%	0.127677
GO:0006809~nitric oxide biosynthetic process	3	0.27%	0.318554
GO:0046209~nitric oxide metabolic process	3	0.27%	0.318554

Median: 0.2640537134097305 Geo: 0.24588531886167345

Term	Count	%	PValue
GO:0016868~intramolecular transferase activ	3	0.27%	0.100971
GO:0016866~intramolecular transferase activ	4	0.36%	0.182498
GO:0016853~isomerase activity	9	0.81%	0.345609
Carbohydrate transport and metabolism	7	0.63%	0.573973

Median: 0.21316646918908094 Geo: 0.24597617148673978

Term	Count	%	PValue
GO:0004468~lysine N-acetyltransferase activ	3	0.27%	0.137908
GO:0004402~histone acetyltransferase activi	3	0.27%	0.137908
GO:0016746~transferase activity, transferrin	12	1.07%	0.162026
GO:0008415~acyltransferase activity	11	0.98%	0.206846
GO:0016747~transferase activity, transferrin	11	0.98%	0.213166
GO:0016410~N-acyltransferase activity	5	0.45%	0.244245

GO:0008080~N-acetyltransferase activity	4	0.36%	0.362031
acyltransferase	6	0.54%	0.457428
GO:0016407~acetyltransferase activity	4	0.36%	0.599807

Median: 0.3846404082940681 Geo: 0.24846768359367194

Term	Count	%	PValue
GO:0006643~membrane lipid metabolic proc	14	1.25%	0.099218
GO:0044255~cellular lipid metabolic process	33	2.95%	0.38464
GO:0006629~lipid metabolic process	39	3.49%	0.401942

Median: 0.34773517538872534 Geo: 0.24956754803083364

Term	Count	%	PValue
rno04662:B cell receptor signaling pathway	9	0.81%	0.050096
rno04360:Axon guidance	11	0.98%	0.220624
rno04650:Natural killer cell mediated cytotoxi	9	0.81%	0.288715
rno04210:Apoptosis	7	0.63%	0.406755
rno04660:T cell receptor signaling pathway	8	0.72%	0.42779
rno04370:VEGF signaling pathway	6	0.54%	0.435149

Median: 0.32482771592771276 Geo: 0.24979656724149604

Term	Count	%	PValue
GO:0035258~steroid hormone receptor bindi	5	0.45%	0.025487
GO:0003713~transcription coactivator activit	14	1.25%	0.060449
GO:0048518~positive regulation of biological	70	6.26%	0.121497
activator	14	1.25%	0.123257
GO:0008134~transcription factor binding	26	2.33%	0.124756
GO:0003712~transcription cofactor activity	19	1.70%	0.14302
GO:0009893~positive regulation of metabolic	28	2.50%	0.201809
GO:0016563~transcription activator activity	19	1.70%	0.273589
GO:0031325~positive regulation of cellular m	24	2.15%	0.376066
GO:0048522~positive regulation of cellular pr	58	5.19%	0.380492
GO:0045935~positive regulation of nucleobas	19	1.70%	0.487533
GO:0045893~positive regulation of transcript	15	1.34%	0.509516
GO:0045941~positive regulation of transcript	17	1.52%	0.607714
GO:0045944~positive regulation of transcript	10	0.89%	0.702208
GO:0006357~regulation of transcription from	23	2.06%	0.714292
GO:0006366~transcription from RNA polymer	25	2.24%	0.933218

Median: 0.261572969683048 Geo: 0.2505067926553862

Term	Count	%	PValue
GO:0005778~peroxisomal membrane	4	0.36%	0.187301
GO:0031903~microbody membrane	4	0.36%	0.187301
GO:0044439~peroxisomal part	4	0.36%	0.261573
GO:0044438~microbody part	4	0.36%	0.261573
GO:0005777~peroxisome	7	0.63%	0.28454
GO:0042579~microbody	7	0.63%	0.28454
GO:0007031~peroxisome organization and bi	3	0.27%	0.318554

Median: 0.18151085579993848	Geo: 0.25534676871162876		
Term	Count	%	PValue
GO:0015630~microtubule cytoskeleton	22	1.97%	0.125925
GO:0007017~microtubule-based process	16	1.43%	0.181511
GO:0005874~microtubule	8	0.72%	0.728409

Median: 0.36267773691844213	Geo: 0.25565972459880804		
Term	Count	%	PValue
GO:0006643~membrane lipid metabolic proc	14	1.25%	0.099218
GO:0006644~phospholipid metabolic process	9	0.81%	0.362678
GO:0006650~glycerophospholipid metabolic p	5	0.45%	0.464381

Median: 0.25291158153882726	Geo: 0.2570069622144935		
Term	Count	%	PValue
GO:0001565~phorbol ester receptor activity	3	0.27%	0.137908
IPR002219:Protein kinase C, phorbol ester/dia	5	0.45%	0.237707
zinc finger region:Phorbol-ester/DAG-type	3	0.27%	0.246277
Phorbol-ester binding	4	0.36%	0.259546
SM00109:C1	5	0.45%	0.277455
GO:0019992~diacylglycerol binding	3	0.27%	0.495685

Median: 0.2646648643314025	Geo: 0.257178985179388		
Term	Count	%	PValue
GO:0031981~nuclear lumen	31	2.77%	0.137206
GO:0043233~organelle lumen	38	3.40%	0.201213
GO:0031974~membrane-enclosed lumen	38	3.40%	0.201213
GO:0044428~nuclear part	45	4.03%	0.264665
GO:0005667~transcription factor complex	13	1.16%	0.312198
GO:0005654~nucleoplasm	22	1.97%	0.320442
GO:0044451~nucleoplasm part	17	1.52%	0.505927

Median: 0.23201158734523689	Geo: 0.2583443959260503		
Term	Count	%	PValue
GO:0006903~vesicle targeting	3	0.27%	0.147649
GO:0051650~establishment of vesicle localiza	3	0.27%	0.210485
GO:0051648~vesicle localization	3	0.27%	0.232012
GO:0051640~organelle localization	4	0.36%	0.307828
GO:0051656~establishment of organelle loca	3	0.27%	0.518472

Median: 0.25368116831589566	Geo: 0.25999331869230846		
Term	Count	%	PValue
GO:0042745~circadian sleep/wake cycle	3	0.27%	0.189177
GO:0048512~circadian behavior	3	0.27%	0.210485
GO:0030431~sleep	3	0.27%	0.232012
GO:0007622~rhythmic behavior	3	0.27%	0.275351
GO:0048511~rhythmic process	9	0.81%	0.276768
GO:0007623~circadian rhythm	5	0.45%	0.438703

Median: 0.43323106073215817	Geo: 0.26210156016547564		
Term	Count	%	PValue
GO:0030031~cell projection biogenesis	8	0.72%	0.018509
GO:0048699~generation of neurons	27	2.42%	0.093349
GO:0022008~neurogenesis	29	2.59%	0.105274
GO:0048858~cell projection morphogenesis	20	1.79%	0.129073
GO:0030030~cell projection organization and	20	1.79%	0.129073
GO:0032990~cell part morphogenesis	20	1.79%	0.129073
GO:0030182~neuron differentiation	20	1.79%	0.370018
GO:0048666~neuron development	15	1.34%	0.405516
GO:0031175~neurite development	13	1.16%	0.460946
GO:0000902~cell morphogenesis	28	2.50%	0.512472
GO:0032989~cellular structure morphogenes	28	2.50%	0.512472
GO:0000904~cellular morphogenesis during c	12	1.07%	0.520972
GO:0048812~neurite morphogenesis	11	0.98%	0.525559
GO:0048667~neuron morphogenesis during c	11	0.98%	0.525559
GO:0009653~anatomical structure morphoge	59	5.28%	0.618174
GO:0007409~axonogenesis	8	0.72%	0.784897

Median: 0.2920008222498741	Geo: 0.27413750077368854		
Term	Count	%	PValue
GO:0033014~tetrapyrrole biosynthetic proces	3	0.27%	0.168192
GO:0006779~porphyrin biosynthetic process	3	0.27%	0.168192
GO:0051188~cofactor biosynthetic process	9	0.81%	0.244059
GO:0033013~tetrapyrrole metabolic process	3	0.27%	0.339943
GO:0006778~porphyrin metabolic process	3	0.27%	0.339943
GO:0046483~heterocycle metabolic process	7	0.63%	0.531976

Median: 0.2589829024123425	Geo: 0.2748545576081132		
Term	Count	%	PValue
GO:0009310~amine catabolic process	7	0.63%	0.174917
GO:0044270~nitrogen compound catabolic pr	7	0.63%	0.192318
binding site:Pyridoxal phosphate (covalent)	4	0.36%	0.258983
GO:0009063~amino acid catabolic process	5	0.45%	0.38641
rno00252:Alanine and aspartate metabolism	3	0.27%	0.465956

Median: 0.315135754469714	Geo: 0.27852397661412764		
Term	Count	%	PValue
GO:0005773~vacuole	13	1.16%	0.212609
GO:0005764~lysosome	11	0.98%	0.315136
GO:0000323~lytic vacuole	11	0.98%	0.322484

Median: 0.3837679797429484	Geo: 0.28680725809930663		
Term	Count	%	PValue
GO:0001701~in utero embryonic developmer	8	0.72%	0.09846
GO:0009790~embryonic development	21	1.88%	0.308097
GO:0043009~chordate embryonic developme	9	0.81%	0.459439

GO:0009792~embryonic development ending	9	0.81%	0.485498

Median: 0.43870341527122014	Geo: 0.29161371042556156		
Term	Count	%	PValue
GO:0051241~negative regulation of multicellu	9	0.81%	7.98E-04
GO:0008016~regulation of heart contraction	5	0.45%	0.281088
GO:0003015~heart process	5	0.45%	0.373214
GO:0060047~heart contraction	5	0.45%	0.373214
GO:0035150~regulation of tube size	5	0.45%	0.412681
GO:0050880~regulation of blood vessel size	5	0.45%	0.412681
GO:0003018~vascular process in circulatory s	5	0.45%	0.438703
GO:0008217~regulation of blood pressure	6	0.54%	0.452147
GO:0042311~vasodilation	3	0.27%	0.553633
GO:0035295~tube development	11	0.98%	0.584276
GO:0035239~tube morphogenesis	8	0.72%	0.611771
GO:0008015~blood circulation	10	0.89%	0.726533
GO:0003013~circulatory system process	10	0.89%	0.726533

Median: 0.2779018162955732	Geo: 0.29279001064309174		
Term	Count	%	PValue
GO:0043284~biopolymer biosynthetic process	7	0.63%	0.134529
GO:0033692~cellular polysaccharide biosynth	4	0.36%	0.213759
GO:0000271~polysaccharide biosynthetic pro	4	0.36%	0.229067
GO:0009250~glucan biosynthetic process	3	0.27%	0.232012
GO:0005978~glycogen biosynthetic process	3	0.27%	0.232012
GO:0005977~glycogen metabolic process	4	0.36%	0.323792
GO:0006073~glucan metabolic process	4	0.36%	0.339757
GO:0044264~cellular polysaccharide metabol	5	0.45%	0.412681
GO:0005976~polysaccharide metabolic proce	5	0.45%	0.42573
GO:0016051~carbohydrate biosynthetic proce	6	0.54%	0.675549

Median: 0.2934854081935494	Geo: 0.2955976854979881		
Term	Count	%	PValue
GO:0051093~negative regulation of developm	9	0.81%	0.136133
GO:0050771~negative regulation of axonoger	3	0.27%	0.147649
GO:0050767~regulation of neurogenesis	6	0.54%	0.225853
GO:0050768~negative regulation of neuroger	3	0.27%	0.361117
GO:0050770~regulation of axonogenesis	3	0.27%	0.518472
GO:0007409~axonogenesis	8	0.72%	0.784897

Median: 0.3419389271765082	Geo: 0.3125283750727572		
Term	Count	%	PValue
isomerase	7	0.63%	0.237841
Rotamase	3	0.27%	0.29287
GO:0003755~peptidyl-prolyl cis-trans isomera	3	0.27%	0.341939
GO:0016853~isomerase activity	9	0.81%	0.345609
GO:0016859~cis-trans isomerase activity	3	0.27%	0.362203

Median: 0.3185542039557815 Geo: 0.3141499002215701

Term	Count	%	PValue
GO:0007043~intercellular junction assembly	3	0.27%	0.210485
GO:0045216~intercellular junction assembly a	3	0.27%	0.318554
GO:0007028~cytoplasm organization and bio	3	0.27%	0.462387

Median: 0.4768385859093126 Geo: 0.31852749595736973

Term	Count	%	PValue
GO:0006767~water-soluble vitamin metabolic	9	0.81%	0.007896
GO:0006769~nicotinamide metabolic process	5	0.45%	0.089544
GO:0006766~vitamin metabolic process	9	0.81%	0.106836
GO:0019362~pyridine nucleotide metabolic p	5	0.45%	0.107637
GO:0044262~cellular carbohydrate metabolic	26	2.33%	0.11154
GO:0006098~pentose-phosphate shunt	3	0.27%	0.168192
GO:0006066~alcohol metabolic process	23	2.06%	0.243199
GO:0006739~NADP metabolic process	3	0.27%	0.253662
rno00010:Glycolysis / Gluconeogenesis	5	0.45%	0.360616
GO:0005975~carbohydrate metabolic process	30	2.68%	0.364572
glycolysis	4	0.36%	0.400613
GO:0016616~oxidoreductase activity, acting	7	0.63%	0.471222
GO:0006007~glucose catabolic process	7	0.63%	0.482455
GO:0006006~glucose metabolic process	10	0.89%	0.514797
GO:0046365~monosaccharide catabolic proce	7	0.63%	0.531976
GO:0019320~hexose catabolic process	7	0.63%	0.531976
GO:0046164~alcohol catabolic process	7	0.63%	0.56072
GO:0019318~hexose metabolic process	11	0.98%	0.659195
GO:0005996~monosaccharide metabolic proc	11	0.98%	0.67824
binding site:Substrate	6	0.54%	0.716583
GO:0050662~coenzyme binding	9	0.81%	0.750593
GO:0044275~cellular carbohydrate catabolic	7	0.63%	0.767266
GO:0016052~carbohydrate catabolic process	7	0.63%	0.813781
GO:0006096~glycolysis	4	0.36%	0.861085

Median: 0.32733578102708194 Geo: 0.32861072017313064

Term	Count	%	PValue
GO:0006821~chloride transport	6	0.54%	0.16533
GO:0031404~chloride ion binding	5	0.45%	0.185787
GO:0043168~anion binding	5	0.45%	0.197106
GO:0008509~anion transmembrane transport	10	0.89%	0.276798
GO:0015698~inorganic anion transport	11	0.98%	0.294678
GO:0006820~anion transport	13	1.16%	0.327336
GO:0005253~anion channel activity	5	0.45%	0.331305
chloride channel	4	0.36%	0.400613
GO:0005254~chloride channel activity	4	0.36%	0.480429
chloride	4	0.36%	0.501672
GO:0022836~gated channel activity	11	0.98%	0.933086

Median: 0.38303656691347576 Geo: 0.3286281289729965

Term	Count	%	PValue
GO:0019198~transmembrane receptor protei	3	0.27%	0.177206
GO:0004725~protein tyrosine phosphatase ac	6	0.54%	0.383037
SM00194:PTPc	4	0.36%	0.522872

Median: 0.3137784044701102	Geo: 0.35567081697826736		
Term	Count	%	PValue
GO:0016575~histone deacetylation	3	0.27%	0.210485
GO:0004407~histone deacetylase activity	3	0.27%	0.259361
GO:0033558~protein deacetylase activity	3	0.27%	0.259361
GO:0006476~protein amino acid deacetylatio	3	0.27%	0.275351
GO:0016811~hydrolase activity, acting on ca	5	0.45%	0.306082
GO:0019213~deacetylase activity	3	0.27%	0.321475
GO:0016570~histone modification	4	0.36%	0.464591
GO:0016569~covalent chromatin modificatio	4	0.36%	0.479537
GO:0016568~chromatin modification	7	0.63%	0.570116
GO:0016810~hydrolase activity, acting on ca	6	0.54%	0.664852

Median: 0.37687762029993876	Geo: 0.35655060468790495		
Term	Count	%	PValue
GO:0031300~intrinsic to organelle membrane	9	0.81%	0.177475
GO:0031301~integral to organelle membrane	8	0.72%	0.216569
GO:0030173~integral to Golgi membrane	4	0.36%	0.323272
GO:0031228~intrinsic to Golgi membrane	4	0.36%	0.430483
GO:0030176~integral to endoplasmic reticulu	3	0.27%	0.570731
GO:0031227~intrinsic to endoplasmic reticulu	3	0.27%	0.673033

Median: 0.4075141240870743	Geo: 0.35866557898917073		
Term	Count	%	PValue
IPR000162:GPCR, family 3, metabotropic glut	3	0.27%	0.08858
IPR001828:Extracellular ligand-binding recept	5	0.45%	0.310163
IPR000337:GPCR, family 3	4	0.36%	0.354689
IPR011500:GPCR, family 3, nine cysteines reg	3	0.27%	0.46034
GO:0008066~glutamate receptor activity	7	0.63%	0.608926
GO:0008067~metabotropic glutamate, GABA-	5	0.45%	0.779334

Median: 0.3606164229489288	Geo: 0.36505968111636955		
Term	Count	%	PValue
rno05210:Colorectal cancer	9	0.81%	0.144588
rno05215:Prostate cancer	9	0.81%	0.20278
rno05212:Pancreatic cancer	8	0.72%	0.209256
rno05219:Bladder cancer	5	0.45%	0.22194
rno05213:Endometrial cancer	6	0.54%	0.258366
rno04664:Fc epsilon RI signaling pathway	7	0.63%	0.308233
rno05214:Glioma	6	0.54%	0.35282
rno04930:Type II diabetes mellitus	5	0.45%	0.360616
rno04910:Insulin signaling pathway	10	0.89%	0.420725
rno04012:ErbB signaling pathway	7	0.63%	0.480269

rno05221:Acute myeloid leukemia	5	0.45%	0.499266
rno05220:Chronic myeloid leukemia	6	0.54%	0.601857
rno05223:Non-small cell lung cancer	4	0.36%	0.679289
rno04620:Toll-like receptor signaling pathway	6	0.54%	0.689423
rno05218:Melanoma	5	0.45%	0.694744

Median: 0.6107688362713029 Geo: 0.36786362508084713

Term	Count	%	PValue
GO:0043412~biopolymer modification	104	9.30%	0.010652
GO:0006464~protein modification process	101	9.03%	0.011152
GO:0043687~post-translational protein modif	81	7.25%	0.052405
serine/threonine-specific protein kinase	6	0.54%	0.054413
GO:0006796~phosphate metabolic process	58	5.19%	0.203992
GO:0006793~phosphorus metabolic process	58	5.19%	0.203992
phosphotransferase	9	0.81%	0.270275
IPR008271:Serine/threonine protein kinase, ac	14	1.25%	0.389865
atp-binding	39	3.49%	0.418874
ATP	11	0.98%	0.436506
binding site:ATP	15	1.34%	0.467178
GO:0016740~transferase activity	91	8.14%	0.503766
Serine/threonine-protein kinase	13	1.16%	0.523432
domain:Protein kinase	13	1.16%	0.606836
IPR000719:Protein kinase, core	18	1.61%	0.614701
IPR002290:Serine/threonine protein kinase	9	0.81%	0.665432
GO:0016310~phosphorylation	41	3.67%	0.697755
GO:0004713~protein-tyrosine kinase activity	15	1.34%	0.727277
GO:0004674~protein serine/threonine kinase	26	2.33%	0.739647
GO:0006468~protein amino acid phosphoryla	34	3.04%	0.74446
GO:0016301~kinase activity	43	3.85%	0.775186
GO:0016772~transferase activity, transferring	50	4.47%	0.806929
active site:Proton acceptor	16	1.43%	0.829169
kinase	22	1.97%	0.878988
GO:0016773~phosphotransferase activity, alc	35	3.13%	0.882313
SM00220:S_TKc	16	1.43%	0.886968
nucleotide phosphate-binding region:ATP	15	1.34%	0.911234
GO:0004672~protein kinase activity	29	2.59%	0.915438

Median: 0.36474197152379556 Geo: 0.3745507585387694

Term	Count	%	PValue
domain:Helix-loop-helix motif	6	0.54%	0.103468
SM00353:HLH	7	0.63%	0.226373
DNA-binding region:Basic motif	6	0.54%	0.346947
IPR001092:Basic helix-loop-helix dimerisation	6	0.54%	0.364742
GO:0003705~RNA polymerase II transcription	3	0.27%	0.440534
IPR011598:Helix-loop-helix DNA-binding	3	0.27%	0.803061
GO:0003702~RNA polymerase II transcription	5	0.45%	0.986203

Median: 0.40335407232030307 Geo: 0.3871980889214496

Term	Count	%	PValue
GO:0009719~response to endogenous stimul	28	2.50%	0.170654
GO:0006974~response to DNA damage stimu	15	1.34%	0.366557
GO:0006281~DNA repair	12	1.07%	0.403354
GO:0006260~DNA replication	11	0.98%	0.533056
GO:0006259~DNA metabolic process	32	2.86%	0.647067

Median: 0.3945131660035479 Geo: 0.39080685877127763

Term	Count	%	PValue
IPR013766:Thioredoxin domain	3	0.27%	0.297638
GO:0045454~cell redox homeostasis	5	0.45%	0.373214
IPR006662:Thioredoxin-related	3	0.27%	0.415812
IPR012335:Thioredoxin fold	6	0.54%	0.505016

Median: 0.577788008160159 Geo: 0.40749427993885956

Term	Count	%	PValue
IPR001881:EGF-like calcium-binding	9	0.81%	0.027665
SM00179:EGF_CA	9	0.81%	0.039536
domain:EGF-like 1	6	0.54%	0.132888
IPR000152:Aspartic acid and asparagine hydr	7	0.63%	0.164861
IPR006209:EGF-like	8	0.72%	0.193811
egf-like domain	11	0.98%	0.205223
IPR000742:EGF-like, type 3	10	0.89%	0.212821
domain:EGF-like 3; calcium-binding	3	0.27%	0.220383
IPR013091:EGF calcium-binding	5	0.45%	0.251909
IPR006210:EGF	10	0.89%	0.254032
SM00181:EGF	10	0.89%	0.280013
domain:EGF-like 2	4	0.36%	0.316111
domain:EGF-like 2; calcium-binding	3	0.27%	0.423992
IPR013032:EGF-like region	11	0.98%	0.511755
rno04610:Complement and coagulation casca	5	0.45%	0.528473
serine proteinase	3	0.27%	0.627103
GO:0007596~blood coagulation	5	0.45%	0.688275
GO:0050817~coagulation	5	0.45%	0.697531
GO:0007599~hemostasis	5	0.45%	0.724107
active site:Charge relay system	5	0.45%	0.853922
serine protease	5	0.45%	0.860029
domain:Peptidase S1	3	0.27%	0.864596
IPR001314:Peptidase S1A, chymotrypsin	5	0.45%	0.871184
GO:0050878~regulation of body fluid levels	5	0.45%	0.897558
IPR001254:Peptidase S1 and S6, chymotrypsi	5	0.45%	0.903363
GO:0008236~serine-type peptidase activity	9	0.81%	0.911622
GO:0017171~serine hydrolase activity	9	0.81%	0.911622
SM00020:Tryp_SPc	5	0.45%	0.912062
GO:0004252~serine-type endopeptidase acti	7	0.63%	0.957408
protease	12	1.07%	0.976645

Median: 0.40647718696843593 Geo: 0.41412682105793264

Term	Count	%	PValue
GO:0006323~DNA packaging	16	1.43%	0.206447
IPR002164:Nucleosome assembly protein (NA	3	0.27%	0.273309
GO:0006334~nucleosome assembly	6	0.54%	0.31441
GO:0006325~establishment and/or maintenar	14	1.25%	0.371224
GO:0031497~chromatin assembly	6	0.54%	0.406477
GO:0000786~nucleosome	4	0.36%	0.489057
GO:0065004~protein-DNA complex assembly	7	0.63%	0.570116
GO:0051276~chromosome organization and b	15	1.34%	0.64874
GO:0006333~chromatin assembly or disasser	6	0.54%	0.739903

Median: 0.532431042883997 Geo: 0.4177511484099943

Term	Count	%	PValue
GO:0046851~negative regulation of bone rem	4	0.36%	0.059801
GO:0030279~negative regulation of ossificati	3	0.27%	0.127677
GO:0046850~regulation of bone remodeling	5	0.45%	0.242656
GO:0030278~regulation of ossification	4	0.36%	0.323792
GO:0048771~tissue remodeling	11	0.98%	0.51045
GO:0030282~bone mineralization	3	0.27%	0.518472
GO:0046849~bone remodeling	10	0.89%	0.54639
GO:0001503~ossification	8	0.72%	0.65935
GO:0031214~biomineral formation	8	0.72%	0.65935
GO:0001649~osteoblast differentiation	3	0.27%	0.84536
GO:0001501~skeletal development	11	0.98%	0.892239
GO:0009888~tissue development	12	1.07%	0.993113

Median: 0.4126814261238582 Geo: 0.42114604607328787

Term	Count	%	PValue
GO:0000245~spliceosome assembly	3	0.27%	0.402649
GO:0000377~RNA splicing, via transesterifica	5	0.45%	0.412681
GO:0000398~nuclear mRNA splicing, via splic	5	0.45%	0.412681
GO:0000375~RNA splicing, via transesterifica	5	0.45%	0.412681
GO:0008380~RNA splicing	9	0.81%	0.468157

Median: 0.46654621200982815 Geo: 0.42269776100474143

Term	Count	%	PValue
GO:0046058~cAMP metabolic process	4	0.36%	0.229067
GO:0006171~cAMP biosynthetic process	3	0.27%	0.382033
GO:0009187~cyclic nucleotide metabolic proc	4	0.36%	0.55106
GO:0009190~cyclic nucleotide biosynthetic p	3	0.27%	0.662001

Median: 0.9529865236334002 Geo: 0.42284236028087474

Term	Count	%	PValue
GO:0007187~G-protein signaling, coupled to c	14	1.25%	0.031366
GO:0019932~second-messenger-mediated si	24	2.15%	0.049374
GO:0019935~cyclic-nucleotide-mediated sign	14	1.25%	0.065869
GO:0007188~G-protein signaling, coupled to c	11	0.98%	0.066354
G protein-coupled receptor	11	0.98%	0.068436

GO:0019933~cAMP-mediated signaling	11	0.98%	0.127194
GO:0008227~amine receptor activity	4	0.36%	0.46613
rno04080:Neuroactive ligand-receptor interac	17	1.52%	0.673005
PIRSF002406:vertebrate rhodopsin	4	0.36%	0.841308
transducer	20	1.79%	0.952739
g-protein coupled receptor	18	1.61%	0.952987
IPR000276:Rhodopsin-like GPCR superfamily	12	1.07%	0.999899
receptor	36	3.22%	0.999976
GO:0007166~cell surface receptor linked sigr	76	6.80%	1
GO:0060089~molecular transducer activity	86	7.69%	1
GO:0004871~signal transducer activity	86	7.69%	1
GO:0007186~G-protein coupled receptor prot	44	3.94%	1
GO:0004872~receptor activity	60	5.37%	1
GO:0004888~transmembrane receptor activit	38	3.40%	1
GO:0004930~G-protein coupled receptor acti	27	2.42%	1
GO:0001584~rhodopsin-like receptor activity	16	1.43%	1

Median: 0.7093029113193254 Geo: 0.4274336816670745

Term	Count	%	PValue
IPR013753:Ras	9	0.81%	0.152954
IPR003579:Ras small GTPase, Rab type	3	0.27%	0.709303
SM00175:RAB	3	0.27%	0.719805

Median: 0.3316371203131925 Geo: 0.43087329622529147

Term	Count	%	PValue
GO:0031226~intrinsic to plasma membrane	57	5.10%	0.258809
GO:0044459~plasma membrane part	87	7.78%	0.295558
GO:0005887~integral to plasma membrane	55	4.92%	0.325735
GO:0005886~plasma membrane	106	9.48%	0.337539
topological domain:Cytoplasmic	69	6.17%	0.764369
topological domain:Extracellular	43	3.85%	0.99537

Median: 0.44945922077534506 Geo: 0.43464513235413105

Term	Count	%	PValue
GO:0051094~positive regulation of developm	8	0.72%	0.319107
GO:0048871~multicellular organismal homeo	4	0.36%	0.449459
GO:0001894~tissue homeostasis	4	0.36%	0.449459
GO:0045453~bone resorption	3	0.27%	0.553633

Median: 0.685960066589173 Geo: 0.4362470704578489

Term	Count	%	PValue
GO:0018193~peptidyl-amino acid modificatio	10	0.89%	0.171207
GO:0018108~peptidyl-tyrosine phosphorylatio	4	0.36%	0.68596
GO:0018212~peptidyl-tyrosine modification	4	0.36%	0.706929

Median: 0.40963610445819626 Geo: 0.44729887337555274

Term	Count	%	PValue
repeat:2	6	0.54%	0.388537

repeat:1	6	0.54%	0.388537
repeat:3	5	0.45%	0.430735
repeat:4	4	0.36%	0.615625

Median: 0.4774561483027405	Geo: 0.4484195189382892		
Term	Count	%	PValue
GO:0005739~mitochondrion	48	4.29%	0.270112
GO:0031966~mitochondrial membrane	17	1.52%	0.338256
GO:0019866~organelle inner membrane	15	1.34%	0.439673
GO:0005743~mitochondrial inner membrane	14	1.25%	0.470146
GO:0005740~mitochondrial envelope	17	1.52%	0.484766
GO:0031967~organelle envelope	23	2.06%	0.556712
GO:0031975~envelope	23	2.06%	0.556712
GO:0044429~mitochondrial part	20	1.79%	0.576142

Median: 0.4293023416983348	Geo: 0.4612341902779416		
Term	Count	%	PValue
GO:0015629~actin cytoskeleton	15	1.34%	0.308413
GO:0016459~myosin complex	5	0.45%	0.429302
GO:0003774~motor activity	8	0.72%	0.741087

Median: 0.48151244734237886	Geo: 0.47505105782779394		
Term	Count	%	PValue
GO:0007368~determination of left/right symr	3	0.27%	0.462387
GO:0009855~determination of bilateral symn	3	0.27%	0.481512
GO:0009799~determination of symmetry	3	0.27%	0.481512

Median: 0.4460592396549603	Geo: 0.4885246371145563		
Term	Count	%	PValue
GO:0015291~secondary active transmembrai	13	1.16%	0.32978
GO:0015293~symporter activity	9	0.81%	0.421411
GO:0022804~active transmembrane transpor	22	1.97%	0.446059
GO:0015294~solute:cation symporter activity	5	0.45%	0.584325
GO:0015370~solute:sodium symporter activit	3	0.27%	0.768165

Median: 0.5088209052108131	Geo: 0.4962598576338997		
Term	Count	%	PValue
GO:0042733~embryonic digit morphogenesis	3	0.27%	0.127677
GO:0030326~embryonic limb morphogenesis	4	0.36%	0.494285
GO:0035113~embryonic appendage morphog	4	0.36%	0.494285
GO:0035108~limb morphogenesis	4	0.36%	0.508821
GO:0035107~appendage morphogenesis	4	0.36%	0.508821
GO:0048736~appendage development	4	0.36%	0.508821
GO:0060173~limb development	4	0.36%	0.508821
GO:0007389~pattern specification process	10	0.89%	0.54639
GO:0009952~anterior/posterior pattern forma	5	0.45%	0.58499
GO:0003002~regionalization	6	0.54%	0.754437
GO:0048598~embryonic morphogenesis	6	0.54%	0.89166

Median: 0.6014490269812658	Geo: 0.4973764616269408		
Term	Count	%	PValue
GO:0046466~membrane lipid catabolic proce	3	0.27%	0.297004
GO:0016042~lipid catabolic process	6	0.54%	0.601449
GO:0044242~cellular lipid catabolic process	3	0.27%	0.688803

Median: 0.8277224214823803	Geo: 0.5022243656471842		
Term	Count	%	PValue
propeptide:Removed in mature form	9	0.81%	0.175615
GO:0048503~GPI anchor binding	3	0.27%	0.827722
gpi-anchor	3	0.27%	0.87146

Median: 0.7482201941461548	Geo: 0.5043113219971841		
Term	Count	%	PValue
IPR013089:Kelch related	3	0.27%	0.176793
IPR013069:BTB/POZ	4	0.36%	0.390294
IPR011333:BTB/POZ fold	4	0.36%	0.74822
IPR000210:BTB/POZ-like	4	0.36%	0.767953
SM00225:BTB	4	0.36%	0.82276

Median: 0.6629167877214965	Geo: 0.5180844925108304		
Term	Count	%	PValue
GO:0016891~endoribonuclease activity, prod	3	0.27%	0.280132
GO:0016893~endonuclease activity, active w	3	0.27%	0.300856
GO:0004519~endonuclease activity	5	0.45%	0.636226
GO:0004540~ribonuclease activity	4	0.36%	0.689608
GO:0004521~endoribonuclease activity	3	0.27%	0.715294
GO:0004518~nuclease activity	7	0.63%	0.731114

Median: 0.5088209052108131	Geo: 0.5218217098726277		
Term	Count	%	PValue
GO:0042552~myelination	4	0.36%	0.464591
GO:0007272~ensheathment of neurons	4	0.36%	0.508821
GO:0008366~axon ensheathment	4	0.36%	0.508821
GO:0001508~regulation of action potential	4	0.36%	0.616436

Median: 0.5849051118463864	Geo: 0.5232757559198661		
Term	Count	%	PValue
IPR013087:Zinc finger, C2H2-type/integrase, I	8	0.72%	0.332645
IPR015880:Zinc finger, C2H2-like	9	0.81%	0.543597
SM00355:ZnF_C2H2	9	0.81%	0.584905
IPR007087:Zinc finger, C2H2-type	12	1.07%	0.585557
IPR001909:KRAB box	3	0.27%	0.633491

Median: 0.6445007927650344	Geo: 0.5232873981612741		
Term	Count	%	PValue
GO:0009401~phosphoenolpyruvate-depender	3	0.27%	0.189177

GO:0015293~symporter activity	9	0.81%	0.421411
GO:0005351~sugar:hydrogen ion symporter a	3	0.27%	0.610061
GO:0051119~sugar transmembrane transport	3	0.27%	0.67894
GO:0015144~carbohydrate transmembrane t	3	0.27%	0.748128
GO:0008643~carbohydrate transport	4	0.36%	0.831163

Median: 0.5536331420138444 Geo: 0.5271280856259419

Term	Count	%	PValue
GO:0000096~sulfur amino acid metabolic pro	3	0.27%	0.297004
GO:0044272~sulfur compound biosynthetic p	3	0.27%	0.553633
GO:0006790~sulfur metabolic process	4	0.36%	0.890767

Median: 0.601409864525576 Geo: 0.5314855310753033

Term	Count	%	PValue
GO:0051174~regulation of phosphorus metab	6	0.54%	0.383436
GO:0019220~regulation of phosphate metabo	6	0.54%	0.383436
GO:0042325~regulation of phosphorylation	5	0.45%	0.562107
GO:0001932~regulation of protein amino acic	4	0.36%	0.640713
GO:0033238~regulation of amine metabolic p	4	0.36%	0.652439
GO:0006521~regulation of amino acid metab	4	0.36%	0.652439

Median: 0.6966402340618993 Geo: 0.5381382996526384

Term	Count	%	PValue
GO:0050869~negative regulation of B cell act	3	0.27%	0.072716
GO:0051250~negative regulation of lymphocy	3	0.27%	0.382033
GO:0002521~leukocyte differentiation	8	0.72%	0.385125
GO:0050864~regulation of B cell activation	3	0.27%	0.442854
GO:0030183~B cell differentiation	3	0.27%	0.536282
GO:0051249~regulation of lymphocyte activa	5	0.45%	0.538554
GO:0050865~regulation of cell activation	5	0.45%	0.562107
GO:0042113~B cell activation	4	0.36%	0.628711
GO:0045321~leukocyte activation	11	0.98%	0.69664
GO:0050670~regulation of lymphocyte prolife	3	0.27%	0.701535
GO:0032944~regulation of mononuclear cell p	3	0.27%	0.701535
GO:0032943~mononuclear cell proliferation	4	0.36%	0.706929
GO:0046651~lymphocyte proliferation	4	0.36%	0.706929
GO:0051251~positive regulation of lymphocy	3	0.27%	0.713831
GO:0001775~cell activation	12	1.07%	0.731025
GO:0046649~lymphocyte activation	8	0.72%	0.773536
GO:0030098~lymphocyte differentiation	4	0.36%	0.795871

Median: 0.5464358420691726 Geo: 0.5464881667450793

Term	Count	%	PValue
repeat:LRR 4	4	0.36%	0.296977
repeat:LRR 3	4	0.36%	0.37346
repeat:LRR 2	4	0.36%	0.411215
repeat:LRR 1	4	0.36%	0.411215
leucine-rich repeat	6	0.54%	0.681657

IPR001611:Leucine-rich repeat	6	0.54%	0.823577
IPR003591:Leucine-rich repeat, typical subtyp	3	0.27%	0.862004
SM00369:LRR_TYP	3	0.27%	0.876519

Median: 0.5819051364898118 Geo: 0.5596950020270177

Term	Count	%	PValue
GO:0048646~anatomical structure formation	12	1.07%	0.301312
GO:0048514~blood vessel morphogenesis	10	0.89%	0.54639
GO:0001568~blood vessel development	11	0.98%	0.577108
GO:0001525~angiogenesis	8	0.72%	0.586703
GO:0001944~vasculature development	11	0.98%	0.605447
GO:0009887~organ morphogenesis	19	1.70%	0.910833

Median: 0.7146631166959486 Geo: 0.5600330435588263

Term	Count	%	PValue
GO:0007626~locomotory behavior	13	1.16%	0.260627
rno04620:Toll-like receptor signaling pathway	6	0.54%	0.689423
GO:0042330~taxis	6	0.54%	0.739903
GO:0006935~chemotaxis	6	0.54%	0.739903

Median: 0.595986061875998 Geo: 0.5617526454231732

Term	Count	%	PValue
rno01040:Polyunsaturated fatty acid biosynth	3	0.27%	0.319739
lipid synthesis	5	0.45%	0.462802
fatty acid biosynthesis	3	0.27%	0.516909
GO:0006633~fatty acid biosynthetic process	4	0.36%	0.675063
GO:0046394~carboxylic acid biosynthetic pro	4	0.36%	0.780118
GO:0016053~organic acid biosynthetic proces	4	0.36%	0.780118

Median: 0.6014490269812658 Geo: 0.5625121080973473

Term	Count	%	PValue
GO:0004623~phospholipase A2 activity	3	0.27%	0.42142
GO:0004091~carboxylesterase activity	7	0.63%	0.42217
GO:0016042~lipid catabolic process	6	0.54%	0.601449
GO:0004620~phospholipase activity	4	0.36%	0.668674
GO:0016298~lipase activity	4	0.36%	0.787124

Median: 0.5787287488791943 Geo: 0.5668905827307934

Term	Count	%	PValue
GO:0051128~regulation of cellular componen	6	0.54%	0.33732
GO:0008064~regulation of actin polymerizati	3	0.27%	0.536282
GO:0030832~regulation of actin filament lenç	3	0.27%	0.553633
GO:0032535~regulation of cellular componen	3	0.27%	0.57052
GO:0032956~regulation of actin cytoskeleton	3	0.27%	0.586938
GO:0051493~regulation of cytoskeleton orgai	3	0.27%	0.662001
GO:0033043~regulation of organelle organiza	3	0.27%	0.662001
GO:0008154~actin polymerization and/or dep	3	0.27%	0.7257

Median: 0.5999486537353009	Geo: 0.5669168044626316		
Term	Count	%	PValue
GO:0043123~positive regulation of I-kappaB I	6	0.54%	0.394966
GO:0043122~regulation of I-kappaB kinase/N	6	0.54%	0.452147
GO:0007249~I-kappaB kinase/NF-kappaB cas	7	0.63%	0.74775
GO:0009967~positive regulation of signal tra	8	0.72%	0.773536

Median: 1.0	Geo: 0.5670533146296532		
Term	Count	%	PValue
GO:0009582~detection of abiotic stimulus	5	0.45%	0.170357
GO:0009581~detection of external stimulus	5	0.45%	0.26816
GO:0050954~sensory perception of mechanic	5	0.45%	0.412681
GO:0007608~sensory perception of smell	5	0.45%	1
GO:0007606~sensory perception of chemical	6	0.54%	1
GO:0050906~detection of stimulus during ser	5	0.45%	1
GO:0051606~detection of stimulus	7	0.63%	1

Median: 0.609218857807939	Geo: 0.5802975779525337		
Term	Count	%	PValue
GO:0009890~negative regulation of biosynth	4	0.36%	0.434156
GO:0017148~negative regulation of translatic	3	0.27%	0.57052
GO:0031327~negative regulation of cellular b	3	0.27%	0.647918
GO:0051248~negative regulation of protein n	5	0.45%	0.706588

Median: 0.6213308430829785	Geo: 0.6021493472760941		
Term	Count	%	PValue
IPR001440:Tetratricopeptide TPR-1	4	0.36%	0.509556
IPR011990:Tetratricopeptide-like helical	5	0.45%	0.592901
TPR repeat	4	0.36%	0.621331
IPR013026:Tetratricopeptide region	4	0.36%	0.642694
SM00028:TPR	4	0.36%	0.656174

Median: 0.6928277524443143	Geo: 0.6074386132124397		
Term	Count	%	PValue
GO:0055086~nucleobase, nucleoside and nuc	21	1.88%	0.079074
GO:0009117~nucleotide metabolic process	19	1.70%	0.109776
GO:0015992~proton transport	7	0.63%	0.358964
GO:0006163~purine nucleotide metabolic pro	8	0.72%	0.394626
GO:0006818~hydrogen transport	7	0.63%	0.421131
hydrogen ion transport	4	0.36%	0.435119
GO:0009144~purine nucleoside triphosphate	6	0.54%	0.46343
GO:0009165~nucleotide biosynthetic process	10	0.89%	0.498742
GO:0009141~nucleoside triphosphate metabo	6	0.54%	0.507789
GO:0006164~purine nucleotide biosynthetic p	6	0.54%	0.540067
GO:0015077~monovalent inorganic cation tra	8	0.72%	0.566816
GO:0009108~coenzyme biosynthetic process	6	0.54%	0.601449
GO:0009199~ribonucleoside triphosphate me	5	0.45%	0.6286
GO:0009205~purine ribonucleoside triphosph	5	0.45%	0.6286

GO:0016469~proton-transporting two-sector A	4	0.36%	0.632594
GO:0009150~purine ribonucleotide metabolic	6	0.54%	0.639677
GO:0022890~inorganic cation transmembrane	10	0.89%	0.64058
GO:0009152~purine ribonucleotide biosynthe	5	0.45%	0.669169
GO:0009201~ribonucleoside triphosphate bio	4	0.36%	0.68596
GO:0009206~purine ribonucleoside triphosph	4	0.36%	0.68596
GO:0009145~purine nucleoside triphosphate	4	0.36%	0.68596
GO:0015662~ATPase activity, coupled to tran	4	0.36%	0.699695
GO:0009259~ribonucleotide metabolic proces	6	0.54%	0.700848
GO:0009142~nucleoside triphosphate biosynt	4	0.36%	0.706929
GO:0009260~ribonucleotide biosynthetic proc	5	0.45%	0.715446
GO:0046034~ATP metabolic process	4	0.36%	0.717004
GO:0015405~P-P-bond-hydrolysis-driven trans	9	0.81%	0.733388
GO:0015399~primary active transmembrane	9	0.81%	0.733388
GO:0046961~hydrogen ion transporting ATPa	3	0.27%	0.737562
GO:0015986~ATP synthesis coupled proton tr	3	0.27%	0.748188
GO:0043492~ATPase activity, coupled to mov	8	0.72%	0.775888
GO:0006753~nucleoside phosphate metabolic	3	0.27%	0.788436
GO:0006754~ATP biosynthetic process	3	0.27%	0.788436
GO:0015078~hydrogen ion transmembrane tr	6	0.54%	0.788459
GO:0042625~ATPase activity, coupled to tran	5	0.45%	0.805609
GO:0019829~cation-transporting ATPase activ	3	0.27%	0.848745
GO:0042626~ATPase activity, coupled to tran	7	0.63%	0.871155
GO:0006119~oxidative phosphorylation	4	0.36%	0.876716
GO:0016820~hydrolase activity, acting on aci	7	0.63%	0.888939
GO:0042623~ATPase activity, coupled	12	1.07%	0.897126
rno00190:Oxidative phosphorylation	5	0.45%	0.94111
GO:0016887~ATPase activity	13	1.16%	0.952816

Median: 0.7013168766235801 Geo: 0.6297977628010818

Term	Count	%	PValue
GO:0048511~rhythmic process	9	0.81%	0.276768
GO:0001541~ovarian follicle development	3	0.27%	0.402649
GO:0048608~reproductive structure developm	5	0.45%	0.502071
GO:0008406~gonad development	5	0.45%	0.502071
GO:0045137~development of primary sexual	5	0.45%	0.596168
GO:0022601~menstrual cycle phase	3	0.27%	0.662001
GO:0022602~menstrual cycle process	3	0.27%	0.675627
GO:0008585~female gonad development	3	0.27%	0.688803
GO:0046660~female sex differentiation	3	0.27%	0.713831
GO:0046545~development of primary female	3	0.27%	0.713831
GO:0042698~menstrual cycle	3	0.27%	0.7257
GO:0007548~sex differentiation	5	0.45%	0.756788
GO:0003006~reproductive developmental pro	5	0.45%	0.786396
GO:0032504~multicellular organism reproduc	4	0.36%	0.803388
GO:0048609~reproductive process in a multic	4	0.36%	0.803388
GO:0022414~reproductive process	13	1.16%	0.835992

Median: 0.5984469718441101 Geo: 0.6323830465290332

Term	Count	%	PValue
GO:0007276~gamete generation	15	1.34%	0.566156
GO:0048232~male gamete generation	11	0.98%	0.598447
GO:0007283~spermatogenesis	11	0.98%	0.598447
GO:0019953~sexual reproduction	17	1.52%	0.624193
GO:0000003~reproduction	25	2.24%	0.799089

Median: 0.6418794153582141 Geo: 0.6434810458513377

Term	Count	%	PValue
domain:C2 2	3	0.27%	0.557944
domain:C2 1	3	0.27%	0.557944
IPR000008:C2 calcium-dependent membrane	5	0.45%	0.725815
SM00239:C2	5	0.45%	0.758815

Median: 0.5705196311967391 Geo: 0.6563294464415198

Term	Count	%	PValue
GO:0040014~regulation of multicellular orgar	3	0.27%	0.536282
GO:0035264~multicellular organism growth	3	0.27%	0.57052
GO:0040008~regulation of growth	7	0.63%	0.924063

Median: 0.7446078402660412 Geo: 0.6575850095074384

Term	Count	%	PValue
GO:0000785~chromatin	10	0.89%	0.502927
GO:0005694~chromosome	15	1.34%	0.744608
GO:0044427~chromosomal part	13	1.16%	0.759317

Median: 0.5620393231910041 Geo: 0.6810252212277741

Term	Count	%	PValue
GO:0001608~nucleotide receptor activity, G-p	4	0.36%	0.562039
GO:0016502~nucleotide receptor activity	4	0.36%	0.562039
IPR000276:Rhodopsin-like GPCR superfamily	12	1.07%	0.999899

Median: 0.6427726332095793 Geo: 0.7001764684951715

Term	Count	%	PValue
IPR002126:Cadherin	4	0.36%	0.61543
SM00112:CA	4	0.36%	0.642773
GO:0007156~homophilic cell adhesion	6	0.54%	0.867733

Median: 0.793690309997469 Geo: 0.7071541215403462

Term	Count	%	PValue
GO:0045860~positive regulation of protein ki	9	0.81%	0.459439
GO:0043405~regulation of MAP kinase activit	5	0.45%	0.764474
GO:0000187~activation of MAPK activity	3	0.27%	0.822907
GO:0043406~positive regulation of MAP kinas	3	0.27%	0.865197

Median: 0.779359351302318 Geo: 0.708304923911514

Term	Count	%	PValue

GO:0009605~response to external stimulus	42	3.76%	0.465649
GO:0006954~inflammatory response	17	1.52%	0.676617
GO:0009611~response to wounding	23	2.06%	0.882102
GO:0006952~defense response	22	1.97%	0.905651

Median: 0.8552131779570957 Geo: 0.7091622268294516

Term	Count	%	PValue
GO:0022892~substrate-specific transporter a	65	5.81%	0.10346
GO:0005215~transporter activity	81	7.25%	0.112579
GO:0022891~substrate-specific transmembra	48	4.29%	0.401987
GO:0022857~transmembrane transporter act	52	4.65%	0.446023
transport	57	5.10%	0.500143
GO:0022890~inorganic cation transmembran	10	0.89%	0.64058
GO:0015075~ion transmembrane transporter	37	3.31%	0.666394
GO:0006811~ion transport	40	3.58%	0.704556
GO:0006813~potassium ion transport	8	0.72%	0.743226
potassium	6	0.54%	0.751663
GO:0030955~potassium ion binding	5	0.45%	0.779334
GO:0008324~cation transmembrane transpor	26	2.33%	0.820017
potassium transport	5	0.45%	0.823461
ion transport	22	1.97%	0.854332
GO:0022803~passive transmembrane transp	17	1.52%	0.855213
GO:0015267~channel activity	17	1.52%	0.855213
Ionic channel	13	1.16%	0.888466
voltage-gated channel	6	0.54%	0.896856
GO:0015672~monovalent inorganic cation tra	14	1.25%	0.89821
GO:0006812~cation transport	23	2.06%	0.925976
GO:0030001~metal ion transport	18	1.61%	0.928702
GO:0022836~gated channel activity	11	0.98%	0.933086
GO:0005216~ion channel activity	14	1.25%	0.940465
GO:0031420~alkali metal ion binding	6	0.54%	0.950483
GO:0046873~metal ion transmembrane trans	12	1.07%	0.950868
GO:0022838~substrate specific channel activ	14	1.25%	0.954803
GO:0022832~voltage-gated channel activity	6	0.54%	0.957042
GO:0005244~voltage-gated ion channel activ	6	0.54%	0.957042
GO:0005267~potassium channel activity	4	0.36%	0.973171
GO:0005261~cation channel activity	8	0.72%	0.988112
GO:0022843~voltage-gated cation channel ac	3	0.27%	0.996715

Median: 0.6691685395032168 Geo: 0.7168766539023791

Term	Count	%	PValue
GO:0007179~transforming growth factor beta	4	0.36%	0.616436
GO:0007178~transmembrane receptor protei	5	0.45%	0.669169
GO:0007167~enzyme linked receptor protein	12	1.07%	0.89312

Median: 0.6660073746312482 Geo: 0.7181513531178823

Term	Count	%	PValue
GO:0004601~peroxidase activity	3	0.27%	0.666007

GO:0016684~oxidoreductase activity, acting	3	0.27%	0.666007
GO:0016209~antioxidant activity	3	0.27%	0.835007

Median: 0.7350892210174229 Geo: 0.7192759782543552

Term	Count	%	PValue
GO:0042692~muscle cell differentiation	4	0.36%	0.591074
GO:0014706~striated muscle development	6	0.54%	0.648871
GO:0007517~muscle development	8	0.72%	0.821308
GO:0007519~skeletal muscle development	4	0.36%	0.849722

Median: 0.9984766275751946 Geo: 0.7250339984678495

Term	Count	%	PValue
GO:0048029~monosaccharide binding	3	0.27%	0.382226
GO:0030246~carbohydrate binding	8	0.72%	0.998477
GO:0005529~sugar binding	5	0.45%	0.998658

Median: 0.7884358417100072 Geo: 0.7321529665083604

Term	Count	%	PValue
GO:0006730~one-carbon compound metaboli	6	0.54%	0.571327
GO:0043414~biopolymer methylation	3	0.27%	0.788436
GO:0032259~methylation	3	0.27%	0.871273

Median: 0.6691685395032168 Geo: 0.7523899992015123

Term	Count	%	PValue
GO:0051789~response to protein stimulus	5	0.45%	0.669169
GO:0006986~response to unfolded protein	5	0.45%	0.669169
GO:0009607~response to biotic stimulus	8	0.72%	0.95117

Median: 0.7994349606199997 Geo: 0.7551374387493411

Term	Count	%	PValue
Mitochondrion	23	2.06%	0.658318
transit peptide	12	1.07%	0.799435
transit peptide:Mitochondrion	9	0.81%	0.8182

Median: 0.8350065324763454 Geo: 0.7573085527457687

Term	Count	%	PValue
GO:0030594~neurotransmitter receptor activ	7	0.63%	0.565097
GO:0042165~neurotransmitter binding	7	0.63%	0.642168
GO:0004983~neuropeptide Y receptor activity	3	0.27%	0.652657
GO:0008188~neuropeptide receptor activity	3	0.27%	0.835007
GO:0042923~neuropeptide binding	3	0.27%	0.835007
GO:0042277~peptide binding	9	0.81%	0.886941
GO:0001653~peptide receptor activity	4	0.36%	0.975391

Median: 0.7481884961189249 Geo: 0.7666946662279888

Term	Count	%	PValue
GO:0002009~morphogenesis of an epitheliun	6	0.54%	0.675549
GO:0016331~morphogenesis of embryonic ep	3	0.27%	0.748188

GO:0048598~embryonic morphogenesis	6	0.54%	0.89166

Median: 0.8437420023447036 Geo: 0.7823281244291606

Term	Count	%	PValue
GO:0016064~immunoglobulin mediated immu	4	0.36%	0.523135
GO:0019724~B cell mediated immunity	4	0.36%	0.55106
GO:0002449~lymphocyte mediated immunity	4	0.36%	0.663889
GO:0002460~adaptive immune response bas	4	0.36%	0.706929
GO:0002250~adaptive immune response	4	0.36%	0.706929
GO:0002443~leukocyte mediated immunity	4	0.36%	0.754627
GO:0006959~humoral immune response	4	0.36%	0.843742
GO:0050776~regulation of immune response	5	0.45%	0.847884
GO:0002682~regulation of immune system pr	5	0.45%	0.872566
immune response	3	0.27%	0.955648
GO:0002252~immune effector process	4	0.36%	0.971089
GO:0002526~acute inflammatory response	3	0.27%	0.98349
GO:0006955~immune response	12	1.07%	0.999982

Median: 0.8311187328631705 Geo: 0.7899641669263333

Term	Count	%	PValue
GO:0031965~nuclear membrane	5	0.45%	0.656212
GO:0044453~nuclear membrane part	4	0.36%	0.771129
GO:0005635~nuclear envelope	6	0.54%	0.831119
GO:0005643~nuclear pore	3	0.27%	0.833261
GO:0046930~pore complex	3	0.27%	0.877853

Median: 0.8000862740701508 Geo: 0.7916258582136574

Term	Count	%	PValue
GO:0042107~cytokine metabolic process	4	0.36%	0.706929
GO:0001816~cytokine production	5	0.45%	0.800086
GO:0042089~cytokine biosynthetic process	3	0.27%	0.877096

Median: 0.758827055718108 Geo: 0.7991755408655077

Term	Count	%	PValue
GO:0006497~protein amino acid lipidation	3	0.27%	0.7257
GO:0042158~lipoprotein biosynthetic process	3	0.27%	0.758827
GO:0042157~lipoprotein metabolic process	3	0.27%	0.926887

Median: 0.8160828494606095 Geo: 0.8081255298438712

Term	Count	%	PValue
GO:0016337~cell-cell adhesion	13	1.16%	0.792443
GO:0022610~biological adhesion	30	2.68%	0.816083
GO:0007155~cell adhesion	30	2.68%	0.816083

Median: 0.9232974364126381 Geo: 0.8238423678796846

Term	Count	%	PValue
GO:0016126~sterol biosynthetic process	3	0.27%	0.536282
GO:0016125~sterol metabolic process	4	0.36%	0.831163

GO:0008203~cholesterol metabolic process	3	0.27%	0.923297
GO:0006694~steroid biosynthetic process	3	0.27%	0.936727
GO:0008202~steroid metabolic process	5	0.45%	0.984434

Median: 0.9362941363435059 Geo: 0.8360218956586637

Term	Count	%	PValue
GO:0005843~cytosolic small ribosomal subun	3	0.27%	0.408313
GO:0015935~small ribosomal subunit	3	0.27%	0.733306
ribonucleoprotein	8	0.72%	0.797397
GO:0005830~cytosolic ribosome (sensu Euka	3	0.27%	0.818051
rno03010:Ribosome	4	0.36%	0.848076
GO:0033279~ribosomal subunit	4	0.36%	0.936294
ribosomal protein	5	0.45%	0.941543
ribosome	3	0.27%	0.958014
GO:0030529~ribonucleoprotein complex	17	1.52%	0.996945
GO:0003735~structural constituent of ribosor	7	0.63%	0.99988
GO:0005840~ribosome	7	0.63%	0.999957

Median: 0.8700920567964969 Geo: 0.8419358538470354

Term	Count	%	PValue
GO:0003777~microtubule motor activity	4	0.36%	0.728459
GO:0007018~microtubule-based movement	5	0.45%	0.847884
GO:0005875~microtubule associated complex	5	0.45%	0.8923
GO:0030705~cytoskeleton-dependent intrace	5	0.45%	0.911724

Median: 0.8453599387629621 Geo: 0.8498359253984246

Term	Count	%	PValue
GO:0007126~meiosis	3	0.27%	0.84536
GO:0051327~M phase of meiotic cell cycle	3	0.27%	0.84536
GO:0051321~meiotic cell cycle	3	0.27%	0.858859

Median: 0.8437813998480348 Geo: 0.861373682922828

Term	Count	%	PValue
GO:0005615~extracellular space	88	7.87%	0.774662
GO:0044421~extracellular region part	91	8.14%	0.843781
GO:0005576~extracellular region	94	8.41%	0.97776

Median: 0.8877030784420337 Geo: 0.866705571947067

Term	Count	%	PValue
GO:0015179~L-amino acid transmembrane tr	3	0.27%	0.666007
GO:0006865~amino acid transport	4	0.36%	0.837557
GO:0015171~amino acid transmembrane trar	3	0.27%	0.848745
GO:0046942~carboxylic acid transport	5	0.45%	0.885656
GO:0015849~organic acid transport	5	0.45%	0.889751
GO:0015837~amine transport	4	0.36%	0.924703
GO:0046943~carboxylic acid transmembrane	3	0.27%	0.95976
GO:0005342~organic acid transmembrane tra	3	0.27%	0.961621

Median: 0.9354118585438735	Geo: 0.8733251539872539		
Term	Count	%	PValue
IPR000047:Helix-turn-helix motif, lambda-like	3	0.27%	0.633491
IPR001356:Homeobox	5	0.45%	0.921786
SM00389:HOX	5	0.45%	0.935412
IPR012287:Homeodomain-related	4	0.36%	0.95928
Homeobox	4	0.36%	0.969529

Median: 0.9013406927694274	Geo: 0.8824357299623315		
Term	Count	%	PValue
GO:0045792~negative regulation of cell size	3	0.27%	0.748188
GO:0016049~cell growth	7	0.63%	0.869474
GO:0008361~regulation of cell size	7	0.63%	0.891665
GO:0040007~growth	13	1.16%	0.911017
GO:0040008~regulation of growth	7	0.63%	0.924063
GO:0001558~regulation of cell growth	4	0.36%	0.966943

Median: 0.8924802767520075	Geo: 0.8834873488794694		
Term	Count	%	PValue
GO:0006816~calcium ion transport	6	0.54%	0.823628
calcium transport	3	0.27%	0.889928
GO:0015674~di-, tri-valent inorganic cation tr	7	0.63%	0.895033
GO:0030001~metal ion transport	18	1.61%	0.928702

Median: 0.981699345405155	Geo: 0.8890739249359461		
Term	Count	%	PValue
GO:0009897~external side of plasma membra	6	0.54%	0.404713
IPR003598:Immunoglobulin subtype 2	4	0.36%	0.945364
SM00408:IGc2	4	0.36%	0.963565
IPR013151:Immunoglobulin	4	0.36%	0.971303
SM00409:IG	7	0.63%	0.981699
IPR003599:Immunoglobulin subtype	5	0.45%	0.99162
immunoglobulin domain	6	0.54%	0.996245
IPR007110:Immunoglobulin-like	8	0.72%	0.999529
IPR013783:Immunoglobulin-like fold	7	0.63%	0.999939

Median: 0.8515833251974014	Geo: 0.8906688475145337		
Term	Count	%	PValue
GO:0046906~tetrapyrrole binding	8	0.72%	0.851583
GO:0020037~heme binding	8	0.72%	0.851583
GO:0004497~monooxygenase activity	4	0.36%	0.974304

Median: 0.9962434132282394	Geo: 0.8976463845659044		
Term	Count	%	PValue
membrane	163	14.58%	0.3784
topological domain:Cytoplasmic	69	6.17%	0.764369
transmembrane region	83	7.42%	0.872932
transmembrane	117	10.47%	0.910233

GO:0044425~membrane part	261	23.35%	0.977345
GO:0016020~membrane	313	28.00%	0.991044
topological domain:Extracellular	43	3.85%	0.99537
glycoprotein	85	7.60%	0.997117
glycosylation site:N-linked (GlcNAc...)	68	6.08%	0.998767
signal	60	5.37%	0.999805
GO:0031224~intrinsic to membrane	216	19.32%	0.999831
GO:0016021~integral to membrane	214	19.14%	0.999877
disulfide bond	43	3.85%	0.999931
signal peptide	51	4.56%	0.999983

Median: 0.9770492762883788 Geo: 0.898924811484142

Term	Count	%	PValue
Serine protease inhibitor	3	0.27%	0.659227
protease inhibitor	3	0.27%	0.850183
GO:0004867~serine-type endopeptidase inhil	3	0.27%	0.963399
GO:0004866~endopeptidase inhibitor activity	4	0.36%	0.9907
GO:0030414~protease inhibitor activity	4	0.36%	0.991503
GO:0004857~enzyme inhibitor activity	7	0.63%	0.994834

Median: 0.9024340852042524 Geo: 0.8997282058107576

Term	Count	%	PValue
GO:0042060~wound healing	8	0.72%	0.839849
GO:0042246~tissue regeneration	3	0.27%	0.871273
GO:0031099~regeneration	3	0.27%	0.933595
GO:0048589~developmental growth	3	0.27%	0.959249

Median: 0.9522219354712798 Geo: 0.9072825421771014

Term	Count	%	PValue
GO:0005201~extracellular matrix structural c	4	0.36%	0.771531
GO:0044420~extracellular matrix part	4	0.36%	0.89221
GO:0005578~proteinaceous extracellular mat	9	0.81%	0.952222
GO:0031012~extracellular matrix	9	0.81%	0.963879
extracellular matrix	3	0.27%	0.973042

Median: 0.9356066609929128 Geo: 0.9188051673289847

Term	Count	%	PValue
GO:0050776~regulation of immune response	5	0.45%	0.847884
GO:0002682~regulation of immune system pı	5	0.45%	0.872566
GO:0002253~activation of immune response	3	0.27%	0.926887
GO:0051240~positive regulation of multicellu	5	0.45%	0.944326
GO:0050778~positive regulation of immune r	3	0.27%	0.961214
GO:0002684~positive regulation of immune s	3	0.27%	0.966578

Median: 0.9414350302664871 Geo: 0.9229533304598382

Term	Count	%	PValue
GO:0008201~heparin binding	3	0.27%	0.795615
GO:0005539~glycosaminoglycan binding	3	0.27%	0.929526

GO:0030247~polysaccharide binding	3	0.27%	0.941435
GO:0001871~pattern binding	3	0.27%	0.963399
GO:0030246~carbohydrate binding	8	0.72%	0.998477

Median: 0.9456647511946196 Geo: 0.9352281958909899

Term	Count	%	PValue
GO:0006508~proteolysis	36	3.22%	0.876179
GO:0008233~peptidase activity	28	2.50%	0.945665
GO:0004175~endopeptidase activity	16	1.43%	0.98724

Median: 0.9504320986748979 Geo: 0.9412154711720149

Term	Count	%	PValue
ank repeat	4	0.36%	0.916184
IPR002110:Ankyrin	4	0.36%	0.950432
SM00248:ANK	4	0.36%	0.957554

Median: 0.9658152263361006 Geo: 0.9730433063544447

Term	Count	%	PValue
growth factor	4	0.36%	0.962854
GO:0005125~cytokine activity	8	0.72%	0.965815
GO:0008083~growth factor activity	4	0.36%	0.9907
